# Consecutive waves of NODAL inhibition are required for epiblast patterning

**DOI:** 10.1038/s41467-026-78248-8

**Published:** 2026-10-09

**Authors:** Minming Wang, Tien-Chi Jason Hou, JoAnne Villagrana, Yusuke Kijima, Nicolas Dias, Roxanna Altus, Martina Brueckner, Tuğçe Aktaş, Kaelyn Sumigray, Helene Kretzmer, Zachary D. Smith

**Affiliations:** 1 Department of Genetics, Yale Stem Cell Center, Yale School of Medicine, New Haven, CT USA; 2 Max Planck Institute for Molecular Genetics, Berlin, Germany; 3 Department of Pediatrics, Section of Cardiology, Yale School of Medicine, New Haven, CT USA

**Keywords:** Gastrulation, Gene regulatory networks, Differentiation

## Abstract

The mouse genome encodes two *Lefty* genes that antagonize NODAL signaling to break symmetry during gastrulation. Despite their central regulatory role, accurately describing and comparing their embryonic function remains unclear. Here, we generated high replicate powered single-cell transcriptional atlases of *Lefty1* and *Lefty2* mutant embryogenesis to evaluate the effects of perturbed NODAL signaling during gastrulation. We find that both mutants exhibit phenotypes beginning with the earliest primitive streak derivatives, suggesting a sequential shift from anterior Lefty1 to posterior Lefty2-based control that prevents spurious differentiation into extraembryonic and axial mesoendoderm at the cost of embryonic mesoderm and, in the case of Lefty1, rostral forebrain specification. By phasing bulk SMAD2/3 ChIP-seq data into single-cell-resolved descriptions of signal transduction, we identify distinct NODAL-responsive modules that track the context-specific use of this morphogen over gastrulation time. Collectively, our work highlights the potential of single-cell assays to decode how complex differentiation processes resolve morphogen signals in space and time.

## Abstract (web summary)

Repression of NODAL signaling by Lefty1 and Lefty2 is important for symmetry breaking during mouse gastrulation, but the distinct roles of these factors are not known. Here they show that Lefty1 acts earlier and more anteriorly than Lefty2, but that both are required for correct spatiotemporal patterning.

## Introduction

Gastrulation entails the progressive restriction and allocation of lineage progenitors from epiblast to form the correct body plan, a process that involves precise coordination of a relatively modest repertoire of secreted morphogens^1^. A widely accepted paradigm regarding the multifaceted nature of these morphogens posits that their concentration encodes positional information, which cells perceive and interpret to prime differentiation into distinct states through some fixed, preset threshold^2,3^. Despite their centrality in developmental biology, it remains a difficult problem to explicitly link morphogen signaling gradients with internal cell states, especially in the context of the gastrulating mammalian embryo.

The TGF-β proteins NODAL and LEFTY represent one of the classic activator-inhibitor pairs that shape morphogen gradients across multiple model organisms^4–6^. Biochemically, LEFTY antagonizes NODAL signaling by interfering with binding to the ACTIVIN receptor dimers and the EGF-CFC co-receptor complex^7,8^, which otherwise transduces NODAL signaling through SMAD2/3 effectors that translocate to the nucleus to support gene regulatory networks^9^. Notably, both Nodal and Lefty are often induced as part of the same core pathway, contributing to complex feedback loops that reinforce cell state transitions^10,11^. In the mouse genome, two *Lefty* genes, *Lefty1*, and *Lefty2*, have been identified with distinct expression patterns that often spatially and temporally overlap *Nodal* itself during gastrulation^12^. In particular, *Nodal* is uniformly expressed within the epiblast and visceral endoderm (VE)^13^, and overlaps with *Lefty1* within distal and anterior visceral endoderm (D/AVE) during the early stages of gastrulation^14^. In contrast, *Lefty2* is expressed in the newly-formed primitive streak (PS), extending into the mid-distal realm and disappearing as the PS reaches to the edge of the node, mirroring the temporal decline of *Nodal* expression within the posterior embryo^15^.

Efforts to modify the genetic dosage of Nodal through conditional disruption^16,17^, enhancer deletions^10,18,19^, and/or mosaic embryo analysis^13,16^ have revealed a dosage-sensitive mechanism that governs cell behaviors during gastrulation and confirmed the role of NODAL signaling in the differentiation of multiple cell states, including the PS, DVE and AVE, node, and rostral neural ectoderm. In contrast, NODAL antagonist mutants are expected to enhance NODAL activity, providing a unique perspective for studying spatiotemporal dosage effects. For instance, *Lefty2* mutants exhibit a thicker PS and increased mesoderm-like cell formation^15^, whereas *Cerl1-/-; Lefty1-/-* mutants appear to stochastically form multiple primitive streaks^20^. Although previous research has addressed the static phenotypes associated with mutations in NODAL signaling, general genetic approaches have limited utility to fully link these modulators with the timing and dosage of morphogens to their response in different cell types. As such, a more detailed analysis of the relationship between embryonic patterning and LEFTY-mediated NODAL antagonism could elucidate the specific impacts of NODAL activity dynamics on the progression of critical lineages.

To comprehensively understand the dynamics of NODAL signal transduction in mouse gastrulation, we generated Cas9-based *Lefty1* and *Lefty2* mutant embryo time series that span the early streak to post-gastrulation stages and examined them at single-cell resolution against a deeply sampled wild type (WT) transcriptional reference. Our continuous transcriptional model of mouse gastrulation allows us to refine classical observations for these mutants, pinpointing a collective arrest-like phenotyping where the definitive ectoderm is barely specified and primitive streak products are distorted. In particular, we find that the axial mesendoderm, definitive endoderm and extra-embryonic mesoderm are over-represented, seemingly at the cost of the PS-derived definitive mesoderm. By intersecting transcriptionally responsive SMAD2/3-bound target genes with our reference data, we present a detailed temporal and spatial description of NODAL signal transduction in time and space, as well as how these cues are modulated by LEFTY inhibitors. In summary, our results highlight the importance of antagonists to rapidly toggle morphogen activity to license downstream differentiation and offers a refined perspective to the classic morphogen gradient theory as governed by tightly coordinated bursts of state-specific activity and rapid attenuation.

## Results

### Lefty1 and Lefty2 mutants delay gastrulation

**Fig. 1:**
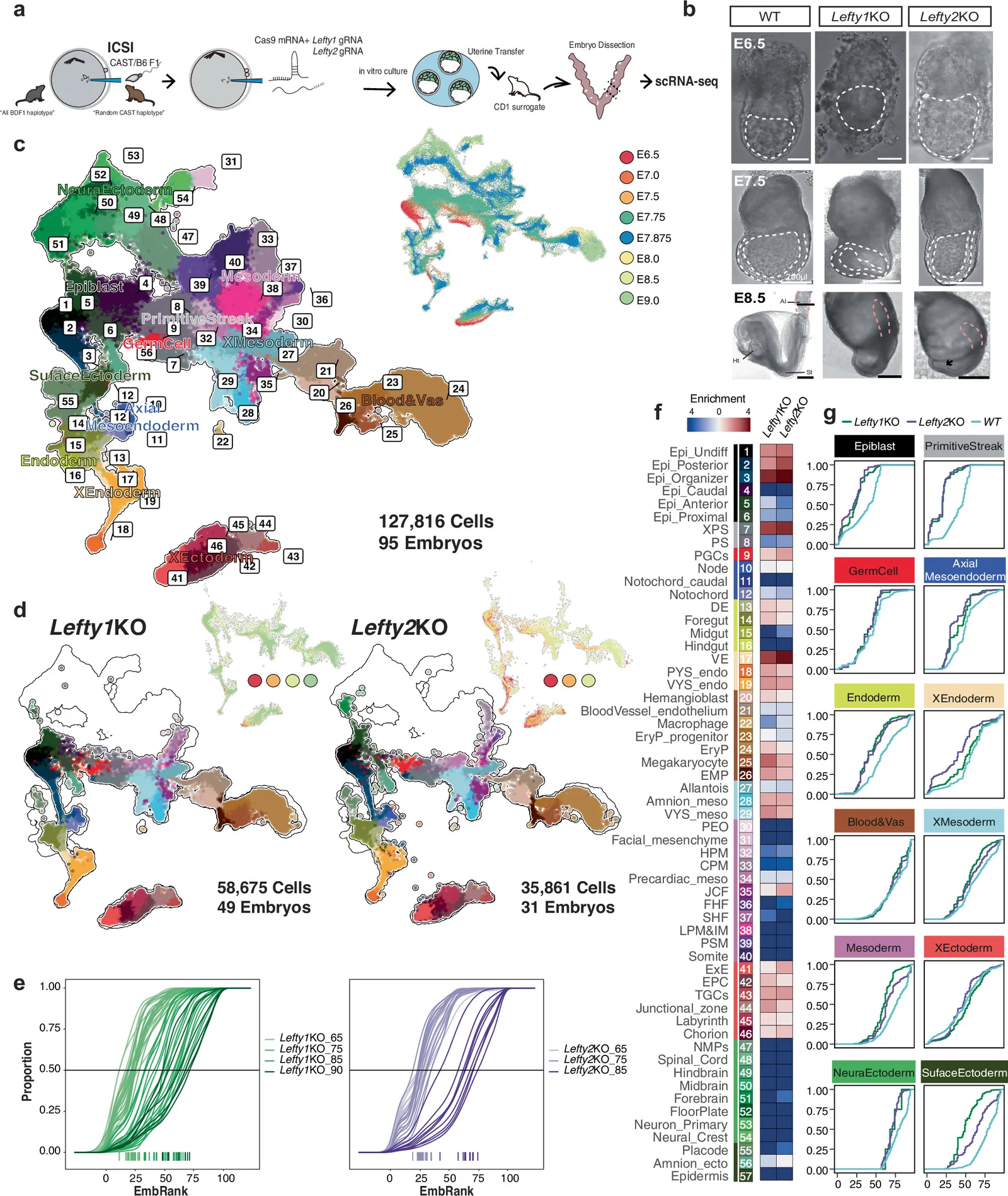
Gastrulating *Lefty* knockout embryos show reduced embryonic mesoderm. **a** Schematic outlining the experimental process for generating demultiplexable *Lefty*KO and WT embryos (“**Methods**”). **b** Representative bright field images of Embryonic day (E)6.5, 7.5 and 8.5 WT and Cas9-generated *Lefty*KO mouse embryos, with prospective epiblast-like cells marked by white dashed lines and enlarged allantois marked by pink dashed lines. Black arrow marks head-fold like structure in *Lefty2* mutants. Scale bar, 50 μm (E6.5), 200 μm (E7.5 and E8.5). Ht, heart; St, somite; Al, allantois. **c** Color-coded uniform manifold approximation and projection (UMAP) of our control reference dataset, comprising 127,816 high-quality single-cell transcriptomes collected from 95 embryos across eight developmental stages. Fate assignment justifications are provided in **Source Data**. **d** Projection of 58,675 *Lefty1*KO (left) and 35,861 *Lefty2*KO (right) single-cell transcriptomes spanning developmental stages E6.5 to E8.5 (and E9.0 for *Lefty1*KO) onto our gastrulation atlas. WT reference cells are shown as background. Both KOs consistently occupy most extra-embryonic states, as well as early definitive endoderm, but show low occupancy of definitive mesoderm or ectoderm. **e** Cumulative distribution functions (CDFs) indicating the relative distribution of mutant cells as they best match the control reference (x-axis, *n* = 95 control embryos, “**Methods”**). The median development rank of each embryo is indicated below the x-axis and shaded by the time of recovery. Overall, *Lefty* mutants exhibit delayed development progression relative to WT. **f** Log2 fold-change in per state proportions between *Lefty* mutants and WT datasets. The color scale is capped to − 4 to 4. **g** Cumulative distribution functions (CDFs) comparing the inferred developmental age of mutant cells across distinct germ layers. Posterior primitive streak products (germ cell, blood and vasculature, and extraembryonic mesoderm) are less affected than the epiblast, primitive streak, axial mesoendoderm, definitive endoderm, mesoderm, and ectoderm lineages, which exhibit substantial delays.

To examine the roles of Lefty1 and Lefty2 during early mouse development, we began by constructing a high-density scRNA-seq atlas of dissociated cells sampled from pools of E6.5 to E9.0 sibling embryos, with dense sampling between E7.5 and E8.0 to capture dynamic transitions in the primitive streak and the induction of neural ectoderm. As described by previous publications from our group^21^, we deployed Intracytoplasmic Sperm Injection (ICSI) of banked CAST/EiJ x C57BL6/J crossed F1 (CF1) sperms into B6D2F1 oocyte, facilitating dense CAST/EiJ polymorphisms-based demultiplexing of individual embryo replicates with our customized computational workflow (Fig. 1a and Supplementary Fig. 1a–d,“**Methods**”). We recovered a total of 127,816 high-quality single-cell transcriptomes from 95 embryos (Fig. 1c, **Source Data**), from which 57 major cell states are identified through iterative clustering and examining stage-specific marker genes (**Source Data**).

We then generated zygotic *Lefty1* and *Lefty2* mutant mouse embryos (Fig. 1b and Supplementary Fig. 2c, d) by introducing Cas9 and three individual single guide RNA (sgRNAs) per target (Fig. 1a, Supplementary Fig. 2a,"**Methods**”). All told, we sampled 49 *Lefty1*KO and 31 *Lefty2*KO embryos by scRNA-seq across developmental stages E6.5 to E8.5 (Fig. 1d), with an additional time point of E9.0 included for *Lefty1*KO (6 embryos) due to their pronounced developmental delays (Fig. 1e). This method enables us to investigate many individually injected embryos to mitigate the effects of rare hypomorphs or stochastic off-target effects, and we confirmed highly efficient target gene disruption by aligning reads from mutant embryos to each target locus (Supplementary Fig. 2a,"**Methods**”)^22^, as well as the reduced *Lefty* transcript counts observed in the *Lefty* mutant datasets (Supplementary Fig. 2b).

Although no discernible morphological differences are observed between mutant and WT embryos at ~ E6.5, disparities between *Lefty*KOs and WT become more apparent after E7.5, including an apparent developmental delay and the retention of epiblast-like cells in regions that should have undergone gastrulation and differentiated into more specialized cell types. Furthermore, in comparison to the embryo proper, the extra-embryonic compartment exhibits pronounced hyperplasia in both mutants, with an enlarged extra-embryonic portion and disproportionately large allantois (Fig. 1b). *Lefty1* mutant embryos appear to display more severe phenotypes than *Lefty2* mutants, including a more pronounced loss of anteroposterior polarity and greater variability in epiblast morphology early during gastrulation, likely reflecting earlier effects on the migration of the AVE/DVE^23^ (Supplementary Fig. 2c, d).

Consistent with our morphological observations, projection of *Lefty1*KO and *Lefty2*KO mutant time series data onto the WT reference revealed similar outcomes. By cell state composition, both mutants initiate gastrulation and produce extraembryonic mesodermal, early endodermal and mesodermal cell states, but otherwise do not efficiently progress into differentiated definitive germ layer derivatives (Fig. 1d, f and Supplementary Figs. 1e, f, 4).

To better leverage our time series data, we timed individual mutant cells by the transcriptional rank (EmbRank) of their nearest WT neighbor, allowing us to examine the relative differentiation of different lineages over the course of embryo patterning (Fig. 1e, “**Methods**”). EmbRank projection revealed substantial heterogeneity in the progression of distinct lineages, with GermCell, XEcto, XMeso and Blood lineages differentiating according to wild-type dynamics. In contrast, products of other germ layers appeared to stall prior to the induction of stable state specific genes, leading to embryos comprised of both appropriately timed and heavily delayed lineages (Fig. 1g). We also observed that the relative severity of this phenotype was greater within the *Lefty1*KO context, including for “E9.0” *Lefty1*KO embryos collected 12 h later than our terminal *Lefty2KO* time point (Fig. 1e). We confirmed this relative ordering of Lefty function by generating a cohort of “DKO” embryos at E7.5 by injecting sgRNAs targeted to both genes, which more closely resembled *Lefty1*KOs in terms of their state composition and gene expression (Supplementary Fig. 2e, f). Taken together, these analyses suggest that Lefty1 and Lefty2 function in similar ways and are both required for proper progression of gastrulation, with Lefty1 acting upstream to license forward differentiation.

### Lefty1 and Lefty2 license early epiblast patterning

**Fig. 2:**
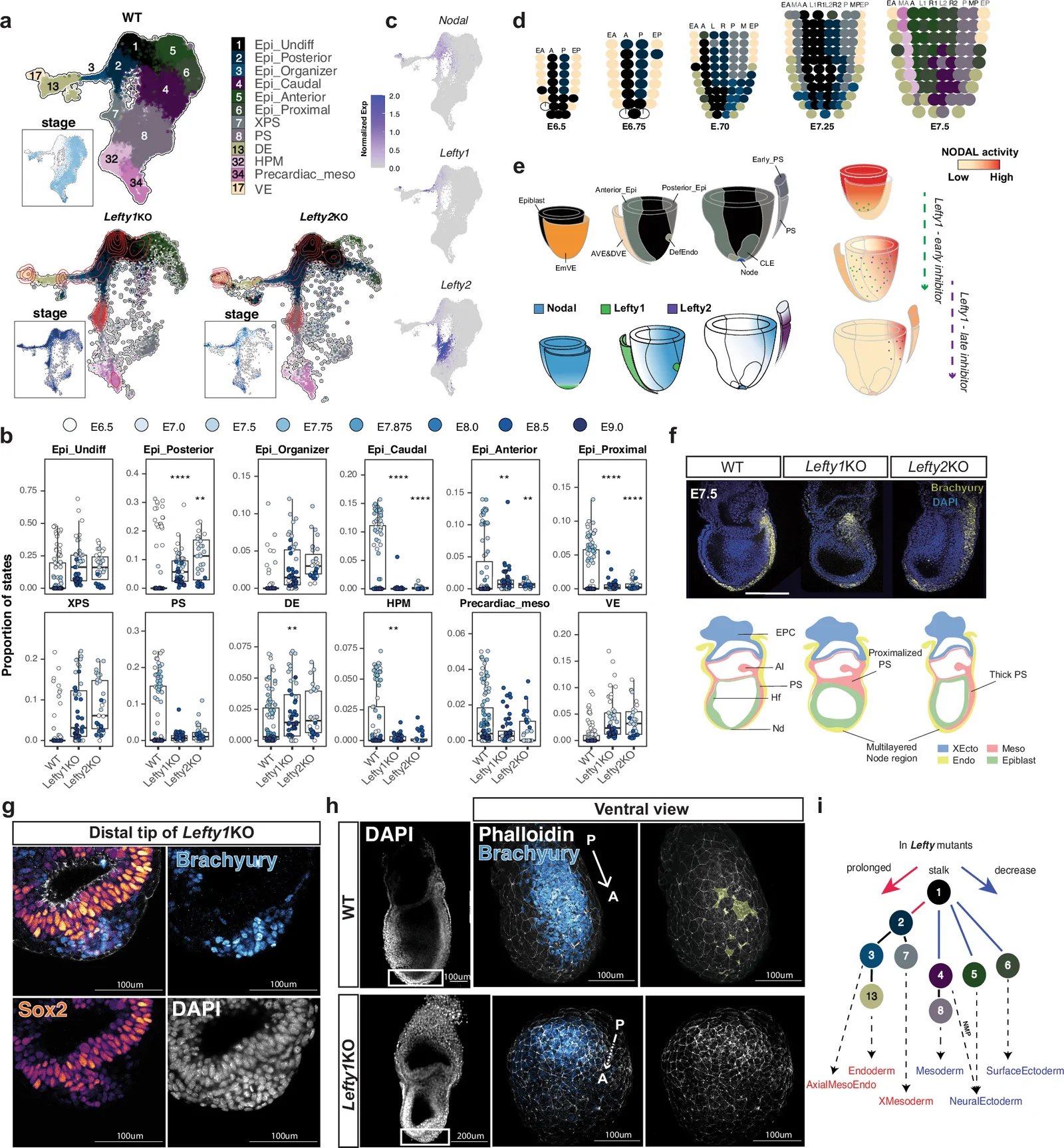
*Lefty* Mutants distort spatiotemporal epiblast patterning. **a** *Top:* Reference UMAP of WT epiblast and early gastrulation-related lineages. *Bottom:* Projection of *Lefty1*KO and *Lefty2*KO lineages onto reference UMAP. Inset density contour overlays show the degree to which mutant datasets reside within earlier WT states, with minimal occupancy of either the caudal or anterior epiblast, nor the embryonic primitive streak (PS). **b** Per-embryo cell states proportions, with each dot representing an individual embryo colored by sampling stage. (* *P* < 0.05, **P < 0.01, ***P < 0.001 ****P < 0.0001, Wilcoxon Rank Sum Test). Center line indicates the median, boundaries the interquartile range (25th–75th percentiles). **c** Normalized expression of *Nodal*, *Lefty1*, and *Lefty2* projected onto the same UMAP as in **Fig. 2a**. **d** Projection of epiblast/PS states onto spatially resolved GEO-seq data (“**Methods**”). A, anterior; L, left lateral; R, right lateral; L1 and R1, left and right anterior lateral; L2 and R2, left and right posterior lateral; M, mesoderm; MA, anterior mesoderm; MP, posterior mesoderm; E, endoderm; EA, anterior endoderm; and EP, posterior endoderm. **e** Summary of *Nodal*, *Lefty1*, and *Lefty2* expression patterns and their roles in regulating NODAL signaling activity. **f** Zoomed-in view of control and *Lefty*KO E7.5 embryos (DAPI, blue; anti-Brachyury, yellow). Scale bar, 200 μm. Bottom panel: schematic summarizing morphological differences at E7.5 (biological replicates, *n* = 3). **g** Magnified lateral and distal view of an E7.5 *Lefty1*KO embryo, showing accumulation of cells between the epiblast and visceral endoderm layers. Sections were stained with DAPI (white), anti-Sox2 (red), and anti-Brachyury (blue). **h** Reconstructed ventral view of WT and *Lefty1*KO embryos at the early head-fold stage. Phalloidin highlights cortical microtubule organization. *Lefty1*KO endodermal cells are markedly smaller, and interaction of node cells (highlighted in yellow in WT) is not observed. **i** Schematic model illustrating impaired epiblast maturation in *Lefty* mutants.

To investigate the spatiotemporal role of NODAL modulators during gastrulation, we identified distinct regional subpopulations within the epiblast lineage, including undifferentiated, posterior, organizer/distal, proximal, anterior and caudal states that emerge over time and show distinct connections to downstream germ layers and cell states. Similarly, we defined temporally distinct primitive streak populations, extraembryonic (XPS) and definitive (PS), based on previously established lineage-specific markers (Source Data). We further verified the regional and temporal distribution of these states were by projecting our scRNAseq-based data onto to spatially resolved Geographical Position Sequencing (Geo-seq) data (Fig. 2a–d; "**Methods”**)^24–26^.

Within this framework, Epi_Posterior represents the earliest epiblast patterning module to stem from “Undifferentiated” (Epi_Undiff), characterized by sustained expression of pluripotency genes together with *Nodal* and *Tdgf1*, which indicate active NODAL signaling^18^. From this early epiblast state, we also identified a distinct population, with sustained epithelial markers, that expresses anterior primitive streak markers such as *Gsc*, *Foxa2*, *Lhx1* and *Chrd*^19,27^. This population closely resembles the organizer population described for the mouse embryo, and we therefore designated it Epi_Organizer. Epi_Organizer shows strong connectivity to axial mesoendoderm and definitive endoderm, consistent with the known fate of the organizer^27^. In parallel, we identified the extraembryonic primitive streak (XPS), marked by high *Kdr* expression in addition to EMT markers. XPS shows strong connectivity to the extraembryonic mesoderm and blood lineages and subsequently merges with the more mature primitive streak state (PS), which contributes to early mesodermal states (Fig. 2a). Thus, as an early differentiation branch of the epiblast, Epi_Posterior appears to give rise to both Epi_Organizer and XPS, while later epiblast patterning leads to the emergence of Epi_Caudal, marked by *Nkx1-2*, *Wnt8a* and *Hoxb1*^28–30^. This population connects to the PS and neuromesodermal progenitors (NMPs), and drives so-called secondary gastrulation of the mouse embryo^31^. All these lineage relationships are also supported by the presence of transitioning cells within our densely sampled reference, and are additionally supported by network flow-based models of the differentiating epiblast^32^.

Projecting *Lefty*KO dataset onto this framework was consistent with the end stage phenotype, including a severe loss of late-timed epiblast branches (Epi_Caudal, Epi_Proximal, and Epi_Anterior) in *Lefty* mutants and marked elongation of Epi_Posterior patterning (Fig. 2a). The extended presence of posteriorized epiblast-like cells also feeds into prolonged differentiation of Epi_Organizer and XPS populations, which likely explains why specification of Node and DE (definitive endoderm) state were relatively preserved (Fig. 2b and Supplementary Fig. 4). In contrast, the severe depletion of later primitive streak (PS) and neuromesodermal progenitor (NMPs) populations corresponded with diminished definitive mesoderm and neural ectoderm products, including limited early mesodermal derivatives such as the *Nkx2-5 *+ precardiac mesoderm (Precardiac_meso) and *Tcf15 *+ head paraxial mesoderm (HPM) (Fig. 2b). In contrast, the early differentiating XPS leads to consistent differentiation of extraembryonic mesoderm and blood states (Supplementary Figs. 3, 4). Interestingly, prolonged specification of the posterior epiblast did not exhaust the undifferentiated epiblast pool, with Epi_Uniff cells persisting into E8.5 and even E9.0 in a manner that likely explains their distorted “shoe-like” morphology (Fig. 1b). Notably, the persistence of extremely early progenitor states is not readily explained by the classic compact gradient-threshold model of NODAL-mediated mesoendodermal fate patterning, where the majority of cells would be consumed according to high signal thresholds.

The striking similarity in epiblast patterning defects between *Lefty1* and *Lefty2* mutants prompted us to ask why two spatially and temporally distinct expression patterns would converge to such similar phenotypes. To explain this, we traced Lefty1 and Lefty2 expression across our dataset. Unexpectedly, we found that *Lefty1* shows low but significant expression within Epi_Organizer cells, in addition to its known expression in a subset of visceral endoderm cells corresponding to the distal and anterior visceral endoderm (D/AVE) (Fig. 2c). This pattern was independently validated using published GEO-seq dataset (Fig. 2d and Supplementary Fig. 3a). Notably, many epiblast cells exhibited concurrent *Nodal* and *Lefty* expression, even though both genes were expressed at low levels (Supplementary Fig. 3b, c). Together, these findings extend our previous understanding of the non-overlapping, distinct spatiotemporal dynamics of *Lefty1* and *Lefty2* during gastrulation. In our view, *Lefty1* exhibits enriched expression in the D/AVE and lower-level expression in the Epi_Organizer, enabling it to exert both anterior and posterior regulatory effects at relatively earlier timepoints. In contrast, *Lefty2* is predominantly induced in XPS and PS populations and acts later during gastrulation (Supplementary Fig. 3b). Although this model requires further validation, NODAL activity in the epiblast appears to be sequentially modulated by Lefty1 and Lefty2 according to space and time, with Lefty1 providing early, broad inhibition from both anterior and posterior regions, and Lefty2 mediating further inhibition from the posterior after gastrulation onset (Fig. 2e). Accordingly, loss of posterior NODAL inhibition may underlie the epiblast’s inability to transition from earlier to later states.

### Incomplete node differentiation distorts the distal epiblast

To gain a clear morphologically understanding of how our single cell data corresponds to mutant phenotypes, we performed a series of immunofluorescence-based analyses on mid-gastrulation stage embryos (Fig. 2f and Supplementary Fig. 5). Brachyury/T staining revealed a striking discontinuous pattern in *Lefty* mutant embryos, with strong signal intensity at the posterior and anterior boundaries but only weak signal between them (Fig. 2f). Notably, the primitive streak in *Lefty1* mutant embryos appeared to align with the short axis rather than the normal long axis of the egg-cylinder, suggesting a failure to transition to the typical longitudinal antero-posterior (A-P) axis^33^. The unstable PS orientation may explain why *Lefty1*KO embryos displayer greater variation in their mid-streak morphologies, which range from a mild, overly symmetrical cylinder to a severe loss of clear anterior-posterior patterning (Supplementary Fig. 2c). This orientation defect is also supported by the inappropriate enrichment of anterior EOMES+ cells and improper localization of Cer1+ cells to the distal region of *Lefty1* mutant embryos (Supplementary Fig. 5a), which recapitulates the DVE migration defects found in *Lefty1* mutants^23^.

Interestingly, when we mapped *Lefty* mutant datasets onto GEO-seq data, we observed that visceral endoderm (VE) and definitive endoderm (DE) populations were less well represented in comparison to Epi_Undiff and Epi_Posterior (Supplementary Fig. 3d), even though they were predicted to be over-represented by our state annotations. This suggests a greater transcriptional divergence of VE and DE between WT and *Lefty* mutants. We confirmed this by calculating PCA centroid distance for each state and performing differential gene expression between the corresponding states (“**Methods**”, Supplementary Fig. 3e, f, **Source Data**). We found that *Lefty1* and *Lefty2* mutants share highly similar misregulated gene sets, with a substantial overlap encompassing approximately one-third of previously identified direct NODAL target genes (**Source Data**, see below). Notably, *Lefty1*KO VE cells showed upregulation of genes associated with cell migration, polarity, and morphology, with significant stronger pathways enrichment in IgSF CAM signaling, Rap1 signaling, actin cytoskeleton organization, and Ras signaling than observed in *Lefty2*KO (Supplementary Fig. 3g). These transcriptional changes may provide a mechanistic explanation for DVE migration defects observed in *Lefty1* mutant embryos^23^.

During normal definitive endoderm (DE) specification, DE cells displace the visceral endoderm (VE) layer through extensive intercalation and mixing^34^. Consistent with intact early DE formation, VE-associated AFP staining was absent from the endodermal layer of E7.5 *Lefty1* mutant embryos, as is expected in WT development (Supplementary Fig. 5b). While this indicates that replacement of the VE by DE proceeds normally in these mutants, we saw a notable distinction for predicted node progenitor cells (dorsal components), which are also expected to intercalate into the visceral endoderm layer (ventral components)^35,36^. Using Foxa2 IF, we confirmed an overabundance of Foxa2+ progenitors in the epiblast of *Lefty1*KO embryos (Supplementary Fig. 5c). At early stages, the relocalization of Foxa2+ organizer cells to the outer layer of the embryo includes shading from the epiblast epithelium, transient residence between the epiblast and visceral endoderm, and fusion with the surface visceral endoderm possibly via a previously reported cyst-like structures^37^. At later stages, these epithelial progenitor cells appear to directly migrate into the surface layer rather than moving as cohesive groups (Supplementary Fig. 5d). Strikingly, we observed an extensive accumulation of cells co-expressing Sox2 and Brachyury/T at the distal tip of E7.5 *Lefty1KO* embryos, a phenomenon not detected in WT embryos and supporting the transcriptional observation that early progenitor states are blocked from complete differentiation (Fig. 2g and Supplementary Fig. 5e). Laminin staining also revealed an extended area of displaced basement membrane, supporting the observation that Lefty1 disruption leads to the accumulation of incompletely differentiated node progenitor cells that are incapable of completing their intercalation into the outermost endodermal layer (Supplementary Fig. 5f). Together, these observations suggest that early organizer cells delaminate from the epiblast layer but remain in an immature state, poised for subsequent intercalation upon NODAL signal modulation (Fig. 2h).

In summary, *Lefty* mutant embryos share a distorted epiblast patterning phenotype, in which the early branches are prolonged without exhausting the pluripotent progenitor pool, while later branches are not robustly induced. Collectively, these results would indicate that Lefty1 and Lefty2 are required to license epiblast progression toward later developmental states (Fig. 2i).

### Ectopic NODAL inhibition partially rescues gastrulation arrest

**Fig. 3:**
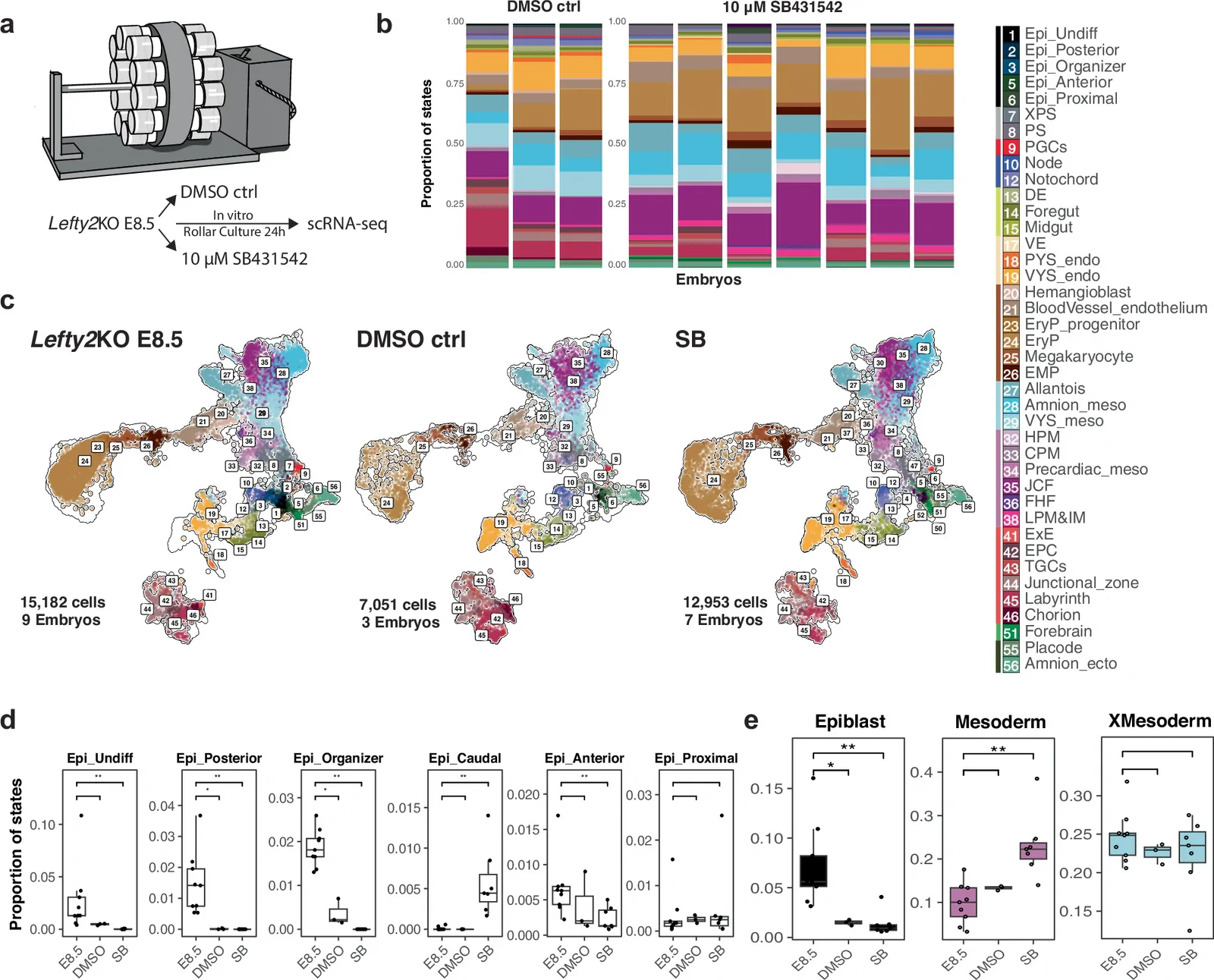
NODAL suppression is required for definitive mesoderm formation. **a** Schematic of the roller culture system and rescue experiment design. E8.5 *Lefty2*KO embryos were cultured in separate flasks in the presence of the NODAL inhibitor SB431542 or a DMSO vehicle control for 24 h, followed by single-cell RNA sequencing (**Methods**). **b** Per-embryo composition of projected cell states following roller culture. *Lefty2*KO embryos treated with SB431542 exhibit a shift toward increased definitive mesoderm differentiation compared with DMSO-treated controls. **c** Color-coded uniform manifold approximation and projection (UMAP) of the *Lefty2*KO E8.5 dataset (left), DMSO control (middle), and SB431542 treatment (right). **d** Per-embryo proportions of epiblast cell states, with each dot representing an individual embryo from E8.5 *Lefty2*KO, DMSO control, or SB431542 treatment groups. *Lefty2*KO embryos cultured with SB431542 show recovery of Epi_Caudal specification, accompanied by reduced proportions of Undiff_Epi and Epi_Posterior states. Statistical significance was evaluated using the Wilcoxon rank-sum test, with *P < 0.05, **P < 0.01, and ***P < 0.001. Sample sizes were WT control, *n* = 10; DMSO group, *n* = 3; and SB group, *n* = 7. The center line indicates the median, the bounds of the box indicate the interquartile range (25th–75th percentiles). **e** Per-embryo proportions of epiblast, mesoderm, and extraembryonic mesoderm (XMesoderm) germ layers, with each dot representing an individual embryo from E8.5 *Lefty2*KO, DMSO control, or SB431542 treatment groups. *Lefty2*KO embryos cultured with SB431542 exhibit increased mesoderm differentiation, while no significant change is observed in extraembryonic mesoderm differentiation. This change is apparent across multiple distinct definitive mesoderm states (see Supplementary Fig. 6). Statistical significance was evaluated using the Wilcoxon rank-sum test, with *P < 0.05, **P < 0.01, and ***P < 0.001. The center line indicates the median, the bounds of the box indicate the interquartile range (25th–75th percentiles).

The presence of residual epiblast and undifferentiated progenitor states in our *Lefty*KO’s prompted us to examine if differentiation could be rescued by the delivery of an ectopic NODAL inhibitor. To do so, we performed an in vitro roller culture-based rescue experiment where *Lefty2*KO mutants were dissected at E8.5 and cultured for 24 hours in either DMSO control or SB431542 (a potent NODAL pathway inhibitor), followed by single-cell sequencing profiling (Fig. 3a–c and Supplementary Fig. 6a). Both culture conditions showed a degree of rescue, including depletion of the Epi_Undiff population compared to the start point (E8.5 *Lefty2*KO) (Fig. 3d, e). In SB-treated embryos, we observed a shutdown of the initial epiblast patterning branches and a switch to later patterning events, including the differentiation of Epi_Caudal and several mesodermal states, while the extraembryonic mesodermal states remained largely unchanged compared to the DMSO control group (Fig. 3e). At the state level, the emergence of neuromesodermal progenitor (NMPs) populations in the SB group also indicated a partial rescue of epiblast caudalization, as did the induction of formal node from immature progenitors and the emergence of early fore- and midbrain states (Supplementary Fig. 6b). Collectively, these data validate the observation that NODAL modulation, rather than thresholding, licenses the forward differentiation of the epiblast and primitive streak.

### D/AVE-expressed Lefty1 protects rostral germ layers

Although depletion of definitive mesoderm and ectoderm is largely shared between *Lefty1* and *Lefty2* mutants, *Lefty2* mutant embryos do weakly produce multiple rostral states compared to *Lefty1* mutants, including forebrain, placode, cranial pharyngeal mesoderm, and juxta-cardiac field (JCF)^38^ (Supplementary Fig. 4). *Lefty2* mutants also exhibit clear morphological features of initial head-fold formation and pharyngeal-arch like ventral structures that extend more distally than WT, consistent with historical in situ staining of forebrain marker *Hesx1*^15^. These findings suggest that anterior patterning is initiated in *Lefty2*KO mutants more efficiently than in *Lefty1*KO mutants, supporting a model in which AVE-driven *Lefty1* expression plays an important role in anterior NODAL inhibition. Given longstanding evidence implicating NODAL signaling in rostral neural patterning, either indirectly through defects in axial mesoderm formation^39^ or more directly via default cell fate decisions^40^, we pursued a focused analysis on this forebrain population.

**Fig. 4:**
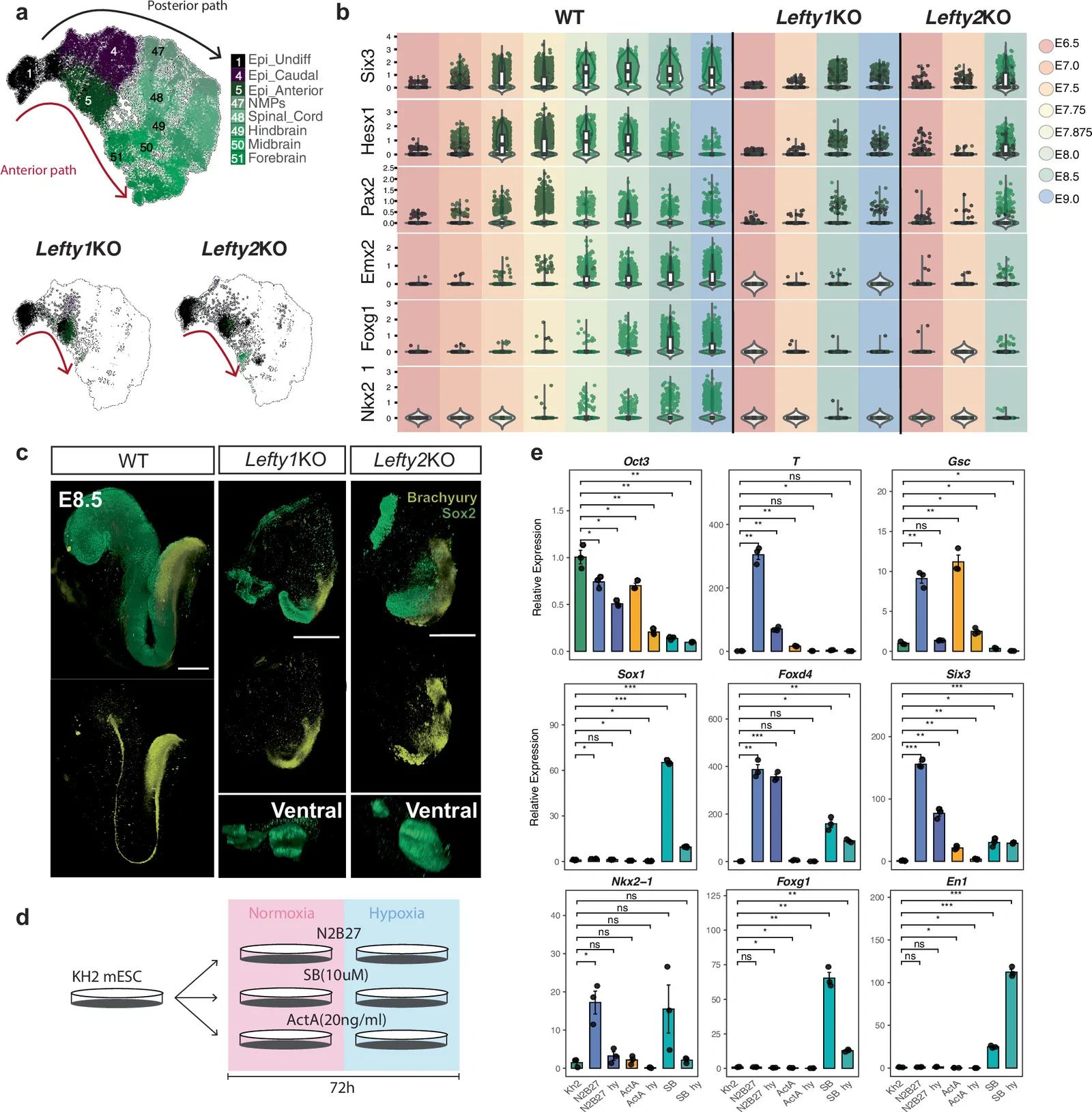
Lefty1 safeguards forebrain fate specification. **a** *Top*: UMAP of epiblast– and neural ectoderm–related lineages. This representation captures anterior–caudal patterning toward the central nervous system, with Epi_Anterior connecting to forebrain and midbrain lineages (Anterior path), and Epi_Caudal contributing to hindbrain, spinal cord, and neuromesodermal progenitors (NMPs) (Posterior path). *Bottom*: Projection of mutant cells corresponding to these lineages onto the control reference subset. Red arrow indicates the differentiation path toward forebrain fates, which progresses further in *Lefty2*KO compared to *Lefty1*KO embryos. **b** Normalized expression levels of forebrain marker genes across developmental stages in WT and *Lefty*KO embryos within the Epi_Undiff-Epi_Anterior-Forebrain trajectory. Early forebrain markers (*Six3, Hesx1, Pax2*), a dorsal forebrain marker (*Emx2*), and ventral forebrain markers (*Foxg1* and *Nkx2-1*) are shown for both WT and *Lefty*KO embryos. Center line indicates the median, the bounds of the box indicate the interquartile range (25th–75th percentiles). **c** 3D reconstruction of E8.5 control, *Lefty1*KO and *Lefty2*KO embryos stained with anti-Sox2 (green) and anti-Brachyury (yellow). Insets show the ventral view of the distal portion of *Lefty*KO embryos to highlight differences in the morphology of SOX2^+^ cells. Scale bar, 200 μm. **d** Schematic of our direct neural differentiation assay designed to test the requirement of NODAL inhibition to support neuralization (“**Methods**”). **e** qRT-PCR analysis of cells cultured as described in Fig. 4d, normalized to an untreated mESC control. SB431542 treatment decreases expression of the pluripotency marker (*Oct3/4*), mesoderm marker (*T*), and NODAL target (*Gsc*) genes, while inducing neural markers (*Sox1, Foxd4*) and forebrain markers (*Six3, Nkx2-1, Foxg1*). Interestingly, Hypoxia and SB specifically promote the expression of the midbrain marker *En1*. Technical replicates (*n* = 3) were performed for each biological replicate, and experiments were independently repeated with three biological replicates (*n* = 3). *P < 0.05, **P < 0.01, and ***P < 0.001, Student’s *t* test.

Lineage-tracing studies of CNS specification in the mouse embryo have revealed a bifurcation-like pattern, supported by both clonal^41^ and *Otx2* mutant analysis^42^. Consistent with these findings, our data clearly reveals distinct anterior and posterior paths from epiblast to neuroepithelium (Fig. 4a). Specifically, most posterior neuroepithelial populations from the hindbrain through the spinal cord appear to be connected to the caudal epiblast, while the forebrain and midbrain lineages are linked to the anterior epiblast. By projecting *Lefty* mutant states onto this framework, we observed a marked depletion of both routes. However, *Lefty2* mutants showed a modest but reproduceable progression in forebrain progression compared to *Lefty1* mutants (Fig. 4a). Consistent with this, transcriptional profiling revealed that the forebrain state in *Lefty2*KO embryos does express moderate levels of early markers, including *Six3*, *Hesx1* and *Pax2*, but shows only weak induction of genes associated with dorsal (i.e., *Emx2*) or ventral (i.e., *Foxg1*, *Nkx2-1*) patterning^43^, indicating that *Lefty2*KO embryos develop only early-stage forebrain-like cells. In contrast, *Lefty1*KO embryos only show the expression of some of these markers within the anterior epiblast with more limited progression along the forebrain differentiation trajectory (Fig. 4b). Together, these results define the anterior-most roles of AVE-based LEFTY1 in protecting the early neural ectoderm.

NODAL inhibition has long been implicated in the classic ‘default model’ of neural induction^44^. To reconfirm that NODAL suppression can directly promote anterior neuroepithelial fate, we performed a simplified neuralization assay in mouse ESCs^45^ (Fig. 4d). To better mimic the early embryonic development environment, we incorporate hypoxic conditions that have recently been reported to favor the generation of anterior tissues with brain-like transcriptional signatures^46^. qRT-PCR results show that SB431542 treatment robustly induced neural genes, with particularly strong upregulation of forebrain markers such as *Foxd4, Six3, Nkx2-1, Foxg1*. Interestingly, the combination of hypoxia and NODAL inhibition specifically induced En1, a midbrain marker, suggesting that hypoxia may synergize with NODAL suppression to fine-tune anterior neural patterning after the early neural ectoderm has been specified (Fig. 4e).

Together, these findings support classic models in which AVE-expressed LEFTY1 safeguards the most anterior ectodermal identities from pre-gastrulation stage, acting as a precisely positioned boundary that extends the functional impact of this transient structure by postponing the posteriorizing effect of NODAL signaling. The increase in forebrain and midbrain cells observed in our roller-culture rescue experiment also supports this interpretation (Supplementary Fig. 6b). However, it cannot fully rule out the possibility that early Lefty1-mediated NODAL inhibition transiently “primes” a subpopulation of epiblast cells, rendering them competent for later anterior neural differentiation.

### Inferring single cell-resolved NODAL signal transduction

**Fig. 5:**
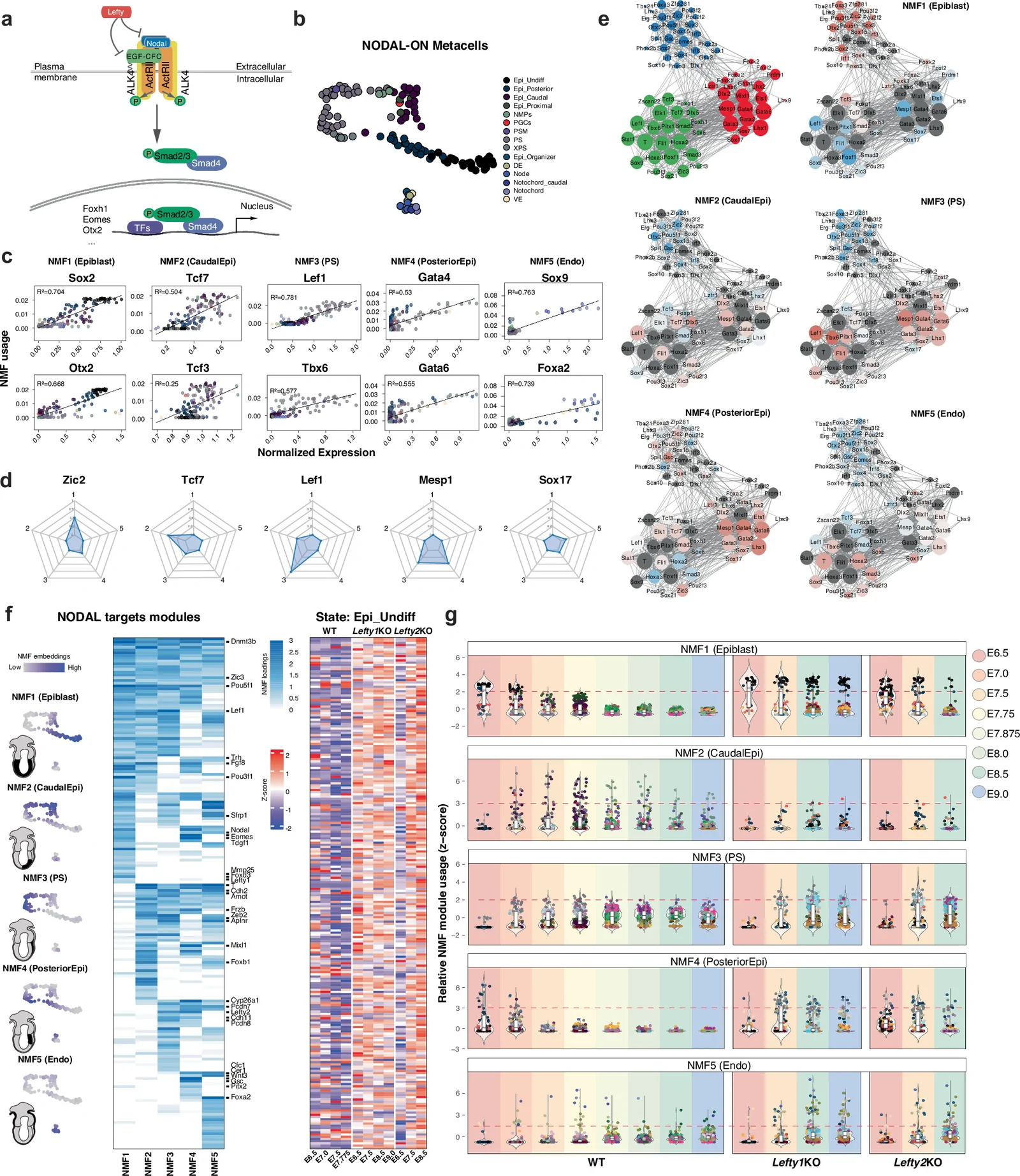
LEFTY1 and 2 license transitions in NODAL signal transduction. **a** Schematic of the NODAL signaling pathway modified from Shen^124^. **b** UMAP of metacells (MCs) exhibiting high NODAL target module scores as inferred by analyzing the transcriptional dynamics of direct SMAD2/3 binding targets. Each MC is colored by its dominant cell state. **c** Correlation between normalized expression of representative transcription factors (as inferred by motif analysis of NODAL targets, see “**Methods**”) and individual NMF embeddings of NODAL modules. **d** Pentagon plot illustrating the relative specificity of individual TFs to one or two distinct NMF modules. **e** WGCNA (Weighted Gene Co-expression Network Analysis)–based transcription factor (TF) co-expression network. 73 TFs inferred from motif enrichment analysis of NODAL target genes were included and grouped into three clusters (“**Methods”**). Node size represents node degree, and edge weight corresponds to the strength of correlation between TFs. TF–NMF module correlations (Spearman correlation) illustrate the relative contribution of different TFs to driving NODAL target gene expression across modules; gray indicates that no enriched motif for the TF within the target genes of a given NMF module. **f** *Left*: NMF embeddings of MCs computed from NODAL target genes, with modules named according to their spatial enrichment. *Middle*: Heatmap showing gene loadings for each NMF module. *Right*: Row-scaled expression of genes, ordered identically to the middle heatmap, in Epi_Undiff from WT and *Lefty*KO datasets. Overall, this analysis highlights the general upregulation and loss of specificity of NODAL target genes in *Lefty* KOs. **g** Scaled NMF module usage between control and mutant cell states. NMF modules 1, 4, and 5 (Epiblast/Posterior Epiblast, Organizer, XPS, VE, DE, and Node) exhibit prolonged usage in *Lefty* mutants, while module 2 (caudal epiblast) is sharply reduced. Center line indicates the median, the bounds of the box indicate the interquartile range (25th–75th percentiles).

To evaluate how LEFTY regulates NODAL activity at the cellular level, we hypothesized the functional output of NODAL signaling activity could be generally captured by examining the integrated expression dynamics of bona-fide SMAD2/3 target genes during early differentiation (Fig. 5a). Conventional approaches such as p-SMAD2 staining^47^ are not feasible for longitudinal tracking and provide limited insight into a cell’s internal state, while current bioinformatic methods that rely on ligand-receptor pair relationships or Gene Ontology only partially address these challenges^48,49^. Here, we estimated NODAL activity by inferring the integrated expression of NODAL target genes within single cells, which we evaluated using SMAD2/3 ChIP-seq data and RNA-seq datasets from *Smad2/3* DKO embryoid bodies (Supplementary Fig. 7a, "**Methods**”)^50,51^. We identified 323 high-confidence target genes that exhibit proximal SMAD2/3 binding and depend on SMAD2/3 for expression, strongly supporting their assignment as direct downstream targets (Supplementary Fig. 7b–d, **Source Data**). Almost all known NODAL targets were included in our list (e.g., *Nodal, Gsc, Eomes, Nanog, Mixl1, Smad7, Lefty1, Lefty2, Cer1, Pitx2*)^10,11,52–60^, and our target genes are well represented in Gene Ontologies related to mesenchyme development and morphogenesis, as well as significantly associated with TGFβ signaling and cell states associated with NODAL sensing (Supplementary Fig. 7e).

We next calculated an integrated module score for NODAL target genes in each Metacell (MC, **“Methods”**), revealing that Epiblast/PS and AME/XEndo lineages display the highest activity (Supplementary Fig. 7f, g). We further grouped these genes using non-negative matrix factorization (NMF), a method that locally decomposes complex data into distinct programs of covarying genes. Applying this method on NODAL-responsive MCs uncovered five spatiotemporally distinct clusters (Fig. 5f and Supplementary Fig. 8a,"**Methods**”): **Module 1** includes pluripotent markers including *Pou5f1*^61^ as well as genes involved in the naïve-to-primed transition such as *Zic2* and *Otx2*^62^; **Module 2** corresponds to a more caudal epiblast identity and predominantly expresses WNT-associate genes, including *Fzd10, Frzb, Wnt5b, Axin2, Wnt8a*^63^; **Module 3** corresponds to a more primitive streak-like identity, but shows the lowest specific usage of module-defining genes (Fig. 5g), suggesting a transient NODAL-responsive state that does not fully commit to formal mesoderm; **Module 4** represents posterior epiblast and organizer-associated states and enriched for genes like *Gsc*^64^, *Cfc1*, *Tdgf1*^65^, *Fgf8*^66^; finally, **Module 5** captures VE and AME identities, marked by enriched expression of *Foxa2*, *Amot*^67^ and *Cer1*^68^. This analysis confirms the general diversity of NODAL signaling responses when confined to single cells, including unique interactions with state-specific signaling axes and transcription factors. Notably, although our analysis showcases the ability to phase distinct transcriptional responses downstream of bulk ChIP-seq data, it remains possible that the use of EB-derived data may lose resolution for the extraembryonic tissues, which are not produced within this system but also respond to NODAL signaling^69^.

We next performed motif enrichment analysis on genes positively related to each module, combined with co-expression analysis, to infer potential TFs that may support the modularized expression of NODAL target genes (“**Methods**”). From 233 modularized NODAL targets, we identified 73 TFs with significantly enriched binding motifs (**Source Data**, Fig. 5e and Supplementary Fig. 8b). Notably, each module was associated with a distinct TF repertoire, with relatively few TFs showing strong correlations across multiple modules (Fig. 5c, d), indicating that NODAL-responsive transcriptional programs interact with highly context-dependent TF combinations. Interestingly, TFs whose motifs were enriched across all five modules—such as Foxh1—exhibited only weak expression correlations with module-specific NODAL targets, suggesting that these broadly acting factors may provide a permissive input, while module specificity is driven by local TF context (Supplementary Fig. 8b). In summary, these analyses suggest that gastrulation-associated NODAL signaling progresses from high to low activity through five major NODAL-responsive modules, each corresponding to distinct developmental stages and spatial domains, and that this progression is likely regulated by combinations of context-dependent transcription factors.

### LEFTY supports transitions in signal transduction

Our ability to infer cell state-specific internal states using our NODAL score further enabled us to examine the effect of Lefty1 or Lefty2 mutation on SMAD2/3 targets. By integrated NODAL score, nearly every embryonic lineage displayed elevated NODAL target expression in *Lefty* mutants compared to WT, and both *Lefty1*KO and *Lefty2*KO showed broadly similar patterns of upregulation, with *Lefty1*KO exhibiting slightly stronger effects in the epiblast, primitive streak, and anterior mesoderm states (Supplementary Fig. 7h, i). Analysis of module-specific usage patterns within our *Lefty*KO atlases revealed sustained usage of modules 1,4 and 5, indicating that unmodulated NODAL activity leads to a largely static transcriptional output (Fig. 5g). Notably, *Lefty1*KO embryos exhibited pronounced up-regulation at earlier developmental stages compared to *Lefty2*KO embryos, again supporting Lefty1’s upstream role in attenuating NODAL signal. The substantial overlap in up-regulated genes between the two mutants further confirm that the overall convergence towards a mesoderm-depleted phenotype arises from the inappropriate maintenance of NODAL target genes in epiblast progenitors (Fig. 5f). Notably, these up-regulated genes displayed prolonged, moderately elevated peak expression levels, suggesting that NODAL antagonism acts to prevent a larger number of cells from responding to this cue more than by driving increased expression levels of genes associated with forward differentiation.

These NODAL target and motif analysis further support the notion that NODAL signaling acts through context specific TF modules to generate spatiotemporally distinct gene expression programs. Neither LEFTY selectively regulates a specific subset of targets, consistent with their general conservation, stability and rapid diffusion. Instead, upregulation of NODAL targets includes many TFs, which may feed into a cell-type-specific gene regulatory network (GRN) after earlier states are properly destabilized.

## Discussion

In this study, we use high-resolution single-cell transcriptional profiling to dissect the spatial and temporal dynamics of NODAL signaling activity during early mouse gastrulation, with a focus on understanding the developmental role of LEFTY-mediated antagonism as a key driver of axis formation. Our study revealed that during gastrulation, high NODAL activity is associated with early differentiation events, whereas lower activity corresponds with secondary waves of anteriorization and caudalization. In both cases, Lefty1 and Lefty2 are temporally and spatially linked to the reduction of NODAL activity as downstream effectors of NODAL signal transduction itself. We confirm a specific role for Lefty1 in protecting rostral cell types, particularly the forebrain, which is more efficiently produced in *Lefty2* mutants. It has been reported that *nodal* null mutants prematurely express markers associated with early forebrain and non-neural ectoderm^40^, and it is well established that A/DVE does not form in these mutants^70^. Therefore, rather than acting as a critical neural-inducing tissue, Lefty1-mediated NODAL antagonism within AVE may primarily function to shield the most anterior ectoderm from NODAL-mediated posterization. It therefore remains an intriguing question whether this posteriorization effect can be reversed by later inhibition of NODAL signaling, or whether early NODAL suppression is instead required to prime the epiblast toward an anterior identity to lock in developmental competence.

Our results clarify a number of classic reports into the function of this essential morphogen and may explain the differences in phenotypes across model organisms. In particular, previous loss-of-function studies on Lefty/Antivin in non-amniote vertebrates have shown a significant expansion of the organizer and mesendoderm progenitors, leading to the enlargement of axial tissues and enhanced formation of mesoderm and endoderm without compromising the ability to generate tailbud-stage embryos^5,71–74^. In mouse embryos, *Lefty2* mutants exhibit an expansion of the primitive streak and excessive mesoderm-like cell formation^15^, which our work clarifies is largely explained by the extended differentiation of XPS-driven extraembryonic mesoderm and primitive blood. Although analysis of *Lefty1* mutants in mice has established its role in left-right axis determination^75^, its early function is considered to be redundant with another NODAL antagonist, Cer1, in the AVE, and the role of LEFTY1 in the differentiation and function of this structure has been less clear^20^. Similar to previous studies^23,76^, we also observed the impaired anterior migration of expanded DVE cells as well as abnormal primitive streak orientation in *Lefty1* mutants, suggesting a specific role of Lefty1 linked to its unique expression in the AVE—an aspect not observed in other model organisms. It has been proposed that rodents evolved a novel function for Lefty1 by acquiring new cis-regulatory elements (CREs) that enable AVE-specific expression, which may explain the adaptations necessary to modulate these conserved signaling cues within the spatially restricted confines of the gastrulating epiblast^77^. Collectively, these results highlight the degree to which early NODAL suppression supports symmetry breaking, as well as multiple potential modes of compensation and feedback that may impact the severity or penetrance of early gastrulation phenotypes within different mutant backgrounds.

Traditionally, morphogen gradients have been thought to induce differential gene expression according to ligand concentration, leading to graded levels of signal activity that direct distinct cell fate changes^2^. However, this basic framework has since been notably expanded and re-contextualized to accommodate many additional features that may affect differentiation, including fold change, duration, accumulation, or rate of change^78^. Key challenges to the traditional gradient model include the ever changing size and shape of the mammalian gastrulating epiblast^79,80^, as well as the unstable short-range nature of the NODAL protein itself, which is more amenable to relay-like propagation to neighboring cells^5,8,47,71,81^. A major refinement of the gradient model has been the integrator model, which proposes that NODAL signaling accumulates over time through autoregulation until sufficient Lefty is translated to inhibit further activation^82^. Since cells are exquisitely sensitive to the low dose of Activin-like signals^83^, the integrator model emphasizes the length of exposure to NODAL signals as crucial in cell fate specification to ensure mesoderm and endoderm formation despite lower ligand concentrations^84^. Despite their differences, both models share the assumption that internal signaling thresholds govern cell fate decisions. However, while cells exhibit varying levels of NODAL signaling activity, there is limited evidence supporting the existence of a strict signaling threshold that dictates distinct cell fates. In a study examining embryoid bodies derived from genetically modified human embryonic stem cells (hESCs) overexpressing *Nodal*, no embryoid body formation was observed over time, nor was there induction of markers for definitive mesoderm or endoderm differentiation^85^. This suggests that an intrinsic threshold for NODAL activity in driving lineage commitment may not exist as currently imagined.

**Fig. 6:**
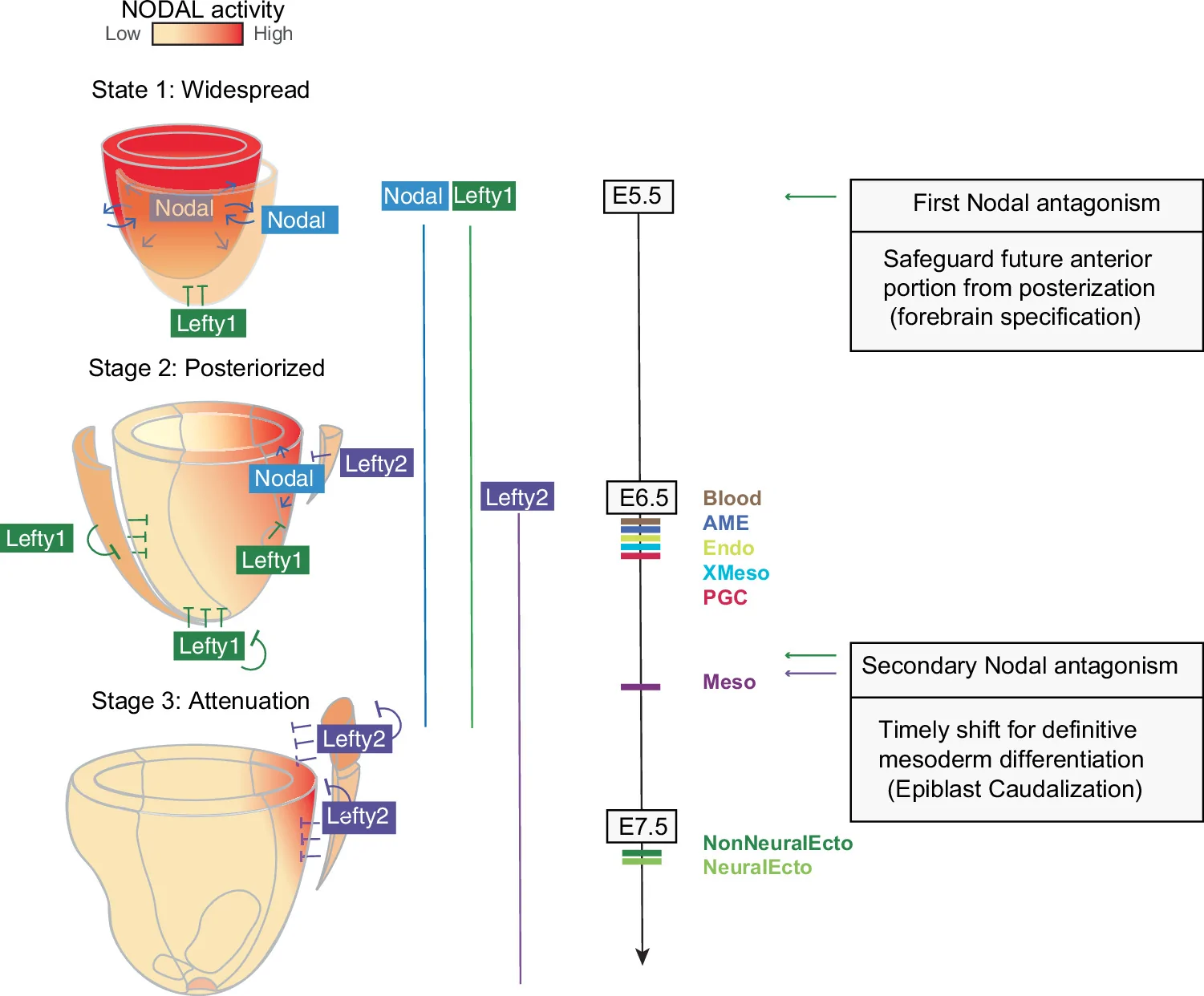
NODAL-based patterning proceeds through multiple phases of LEFTY-based suppression. Functional model of Lefty1 and Lefty2 as they sequentially orient and time individual germ layer derivatives. Initially, Nodal is expressed in the epiblast and induces Lefty1 expression within the VE. Lefty1 expression gradually becomes restricted to the DVE, likely as influenced by the extension of the epiblast and BMP signals from the ExE^125^. As the DVE migrates, Lefty1 protects the adjacent epiblast from posterization, which retains its ability to differentiate into the ectoderm lineage (Fig. 4 and ref. ^40^). By restricting Nodal expression to the posterior epiblast, Lefty1 ensures the correct positioning of the Primitive Streak. The epithelial-mesenchymal transition (EMT) occurs upon receiving the gastrulation signal, possibly downstream of WNT^126^, and primitive streak cells delaminate from the posterior epiblast. During this process, Lefty2 is induced within these delaminating cells to rapidly shut down NODAL activity within the epiblast and license their differentiation towards caudal epiblast, which gives rise to definitive mesoderm.

Based on these studies and our analysis, we propose that a modified “sequential inhibitory wave model” best explains the dynamics of NODAL signaling as inferred from single cell data, as well as the convergent phenotypes of our *Lefty* mutant time series (Fig. 6). In particular, NODAL signal transduction does not appear to encode positional information in a one-to-one manner or to direct pluripotent cells towards multiple fates simultaneously. Instead, it initially drives cells towards a common differentiation competent state, which remains plastic for all epiblast derivatives. Then, NODAL and its induced LEFTY collectively function through a switch-like mechanism to govern distinct transitions. Rather than being determined directly by the duration, level, or integration of NODAL activity, NODAL antagonists instead appear to function by disrupting prior states and licensing new ones. As such, higher levels of NODAL activity either extend the duration prior to differentiation or raise the threshold for antagonists to suppress earlier signals. In this manner, the loss of early or late NODAL antagonists may converge to produce embryos that lack downstream differentiation products. While our model may not be fundamentally different from previous ones, it is modified to align with the biophysical properties of NODAL-LEFTY in the mammalian embryo and incorporate the phenotypic outcomes of persistent NODAL induction. In particular, our data highlight the uniform responses within Lefty *KOs*, where all cell types within the range of NODAL activity exhibit prolonged expression of nearly all NODAL target genes. Such a response strongly indicates that both Lefty1 and Lefty2 constrain the duration and cellular reach of NODAL signaling, rather than impose module-specific transcriptional outputs.

In the context of early mouse embryonic development, the epiblast undergoes a crucial form of symmetry breaking that relies on the convergence of NODAL, BMP and canonical WNT signaling pathways, such that blocking any of these signaling pathways would lead to loss of primitive streak induction or subsequent A-P patterning^86–89^. Notably, our analysis of downstream NODAL target genes includes many genes related to these signaling pathways (e.g., *Wnt3, Wnt5a, Wnt8a, Lef1, Bmpr1b*) that likely interact through complex feedback mechanisms. NODAL is also secreted as a pro-protein that undergoes processing and potentiation by the convertases FURIN and PACE4 within the adjacent extra-embryonic ectoderm, which also senses proNODAL, activates BMP in ExE and participates in DVE patterning^90–92^. BMP signaling in the extra-embryonic ectoderm subsequently activates WNT signaling within the epiblast as well as within the overlying VE^93,94^, which in turn potentiates NODAL signaling by promoting the expression of Nodal and its coreceptor Cripto^90,95^. Although NODAL, WNT, and BMP are all expected to mutually enhance each other’s functions, emerging evidence suggests that they have distinct and modular roles in PS specification. Specifically, BMP signaling is more involved with posterior PS derivatives (extraembryonic mesoderm)^96^, WNT with mid-streak PS derivatives (mesoderm)^97^, and Nodal with anterior PS derivatives (axial mesoendoderm)^98^. The overproduction or lesser impact on extraembryonic mesoderm in our *Lefty* mutants may be partially explained by maintained BMP signaling from the ExE region. Additionally, the loss of definitive mesoderm may stem from delayed or impeded WNT induction. It will therefore be intriguing to further dissect if timely shut down of the NODAL signaling pathway is required to trigger WNT signaling within the mature primitive streak.

In summary, our work demonstrates that Lefty1 and Lefty2 act as consecutive modulators of NODAL signaling, which bookend transitory “competency windows” that permit epiblast patterning and forward differentiation. Each *Lefty* gene appears to play shared and specific roles in this process: Lefty1 orientates PS formation and safeguards anterior structures, while both Lefty1 and Lefty2 aid in consolidating epiblast differentiation and gastrulation completion. Although key predictions of this model have yet to be tested, additional efforts to experimentally modulate NODAL signal behavior will benefit substantially from experimental efforts that examine cellular state in time and space^99^. Beyond our focused investigation, we believe that our study presents an exciting methodology for investigating classical developmental phenotypes using emerging technologies to more broadly probe the internal state of differentiating cells. Moreover, the ability to dissect these processes at the level of gene regulatory networks may open new opportunities to investigate the diversification of evolutionary forms that nonetheless rely on highly conserved signaling pathways to oversee their morphogenesis.

## Methods

### Mice

B6/CAST F1 male mice were generated in-house by breeding C57BL/6 J strain female mice with CAST/EiJ strain males. Swimming sperm were isolated from the caudal epididymis for males ( > 2 months of age) in M2 media (Millipore), decapitated by brief pulse sonication in a Branson Sonifier with a double stepped tip (Branson), and stored at − 40 °C in 25 μl aliquots for use within 6 months to a year of collection. We used 8-week-old male B6/CAST F1 as sperm donors for ICSI and 6–12-week-old B6D2F1/J (BDF1, Jackson Laboratory) female mice to provide oocytes. 22–24 g female CD-1 mice were purchased from Charles River Laboratory and used as pseudopregnant surrogates, and Swiss-Webster vasectomized males (9 weeks old) were purchased from Taconic Biosciences for mating to CD-1 females. All procedures were performed in our specialized, specific-pathogen-free facility, followed all relevant animal welfare guidelines and regulations. Mice were housed at an ambient temperature of 20–26 °C with a relative humidity of 40–60%, maintained on a 12 h light–dark cycle (7 am–7 pm), and provided with a standard diet. Embryos were collected without considering sex, but individual sexes were analyzed after sequencing. Surgical procedures necessary to conduct this work have been approved by the Yale University Committee on the Use of Animals in Research and Teaching (IACUC, Protocol #2020-20357).

### Disrupting lefty1/lefty2 in a hybrid F1 background using zygotic CRISPR-cas9 injection

Embryos were generated as previously described^21,100^. Briefly, BDF1 strain female mice (age 6–8 weeks, Jackson Labs) were superovulated by serial HyperOva (100 μl per mouse, Cosmos Bio USA)^101^ and human chorionic gonadotropin (5 IU per mouse, Millipore) injections ~ 46 h apart. The following day, MII stage oocytes were isolated in M2 media supplemented with hyaluronidase (Millipore) and stored in 25 ml drops of pre-gassed KSOM with a half-strength concentration of amino acids (Millipore) under mineral oil (Irvine Scientific). Zygotes were generated by piezo-actuated intracytoplasmic sperm injection (ICSI^22^,) using thawed sperm in batches of 30–50 oocytes and standard micromanipulation equipment, including a Hamilton Thorne XY Infrared laser, Eppendorf Transferman NK2 and Patchman NP2 micromanipulators, and a Nikon Ti-U inverted microscope.

For zygotic disruption, pronuclear stage 3 (PN3) zygotes were injected with 200 ng/ml Cas9 mRNA and a 100 ng/ml equimolar ratio of 3x sgRNA pools targeted different exons of *lefty1* and *lefty2* (designed using the IDT CRISPR–Cas9 guide RNA checker to select for protospacer sequences with optimal on and off target scores). Cas9 mRNA and sgRNAs were generated via PCR using T7-promoter containing primers and in vitro transcribed using the HiScribe T7 ARCA mRNA kits (with tailing) or HiScribe T7 High Yield RNA Synthesis Kit (NEB), respectively, purified using the Monarch RNA Clean up Kit (New England Biolabs), and resuspended in injection buffer (5 mM Tris buffer, 0.1 mM EDTA, pH 7.4). Injections utilized the same microinjection setup and Piezo = actuated injection of front-filled 6-7 mm injection needles. At around 84 h after fertilization, cavitated blastocysts were transferred into the uterine horns of pseudopregnant CD-1 strain females (25–35 g, Charles River) generated by mating with vasectomized Swiss-Webster strain males (Taconic). Uterine transfer results in a 24 h offset in gestational time to accommodate implantation, after which animals were monitored for embryo isolation. For WT reference, embryos were isolated at 0.5-day intervals from E6.5 to E9.0, with additional sampling at E7.75 and E7.875.

Three lines of evidence support effective disruption of Lefty expression and function by zygotically injecting gRNA and Cas9 mRNA. Firstly, our embryos robustly phenocopy *Lefty* mutants^15,20^. Secondly, we conducted CutSite analysis of the sequencing reads, which reveal high efficiency Indels around the target sites (see Supplementary Fig. 2a)^22^. Finally, the expression of Lefty1 and Lefty2 is significantly reduced across states in scRNA-seq data (Supplementary Fig. 2b).

### Embryo isolation and library preparation for single-cell sequencing

Wild-type and mutant embryos were isolated from surrogate animals after euthanization, and maternal tissues were peeled off by forceps. Embryos were serially washed through several droplets of 1 × PBS and 0.4% BSA, pooled without any morphology-based pre-selection, and subjected to tissue dissociation in 200 μl TrypLE Express (Gibco) for 25 min at 37 °C, with pipetting in 5 min intervals. The cell suspension was filtered using Scienceware Flowmi Cell Strainers, 40 μm. Cells were washed twice with 1 ml 1 × PBS and 0.4% BSA, and centrifuged for 5 min at 1200 rpm. The cell concentration was determined using a hemocytometer, and cells were subjected to single-cell RNA sequencing (10x Genomics, Chromium Single Cell NextGem 3′ v3), aiming for a target cell recovery of up to 13,000 sequenced cells per sequencing library. Single-cell libraries were generated following the manual instructions, except for fewer PCR cycles than recommended during cDNA amplification and library generation/sample indexing to increase library complexity. Libraries were evaluated using a 4200 TapeStation System (Agilent Technologies) and were sequenced with a minimum of 350 million paired-end reads according to parameters as described in the manual.

### Whole-mount immunofluorescence and imaging

The whole-mount immunofluorescence staining of early mouse embryos was done according to the published protocol with minimal modification^102^. Briefly, mouse embryos were recovered at an indicated embryonic stage in PBS and fixed with 4% paraformaldehyde (PFA) at 4 °C for 1-2 h. After being washed three times for 5 minutes with PBS, embryos were permeabilized in PBSTr (sterile PBS with 0.5% Triton X-100) for 20 minutes and followed by Neutralization buffer (sterile PBS with 1 M Glycine and 0.1% PBST) treatment for 15 min at room temperature. After three washes with PBSTw (sterile PBS with 0.01% Tween-20), the embryos were transferred to a blocking buffer (PBSTw with 3% Donkey buffer and 1% BSA, filtered) and incubated at 4 °C overnight. After that, the embryos are stained with primary antibodies at 4 °C for at least two days. After three times of washes with PBSTw, the embryos are stained with secondary antibodies at 4 °C overnight or at room temperature for 2-4 h, followed by DAPI staining and PBST washes before FocusClear™ (CellExplorer) clearing and imaging.

For distal imaging, we adapted a previously described protocol^37^. Intact embryos were positioned in a 35 mm glass-bottom dish (MatTek, P35G-1.5-14-C) containing PBS and immobilized in pre-hardened 1% agarose by creating a small cavity using capillaries. Confocal z-stacks were acquired from the distal side of the embryo.

All bright field images of embryos were acquired using a Leica DMi8 system with a 10x or 20x air objective. All confocal immunofluorescence images were acquired using a Leica STELLARIS 5 microscope or Zeiss LSM880 with a 25x water objective, 20x air objective and 40x oil objective. Images were taken with a z-step of 1–3 μm and appropriate laser and filters for Alexa 405, Alexa 488, Alexa 555, and Alexa 647 or combinations thereof. Images were processed in Fiji ImageJ open access software (1.53t Java 1.8.0_322 (64 bit)) or Leica Application Suite X (3.7.6.25997).

Antibody and reagents used in this study: Sox2 (1:800, rat, #14-9811-82, Invitrogen); Brachyury (1:500, Rabbit, #81694S, CST); Eomes (1:200, Rabbit, #Ab23345, Abcam); Cer1 (1:50, Rat, MAB1986-SP, R&D); Foxa2 (1:500; Rabbit, #8186S, CST); Sox17 (1:400, Goat, #AF1924, R&D); Afp (1:100, Mouse, #MAB1368, R&D); Laminin A (1:500, Rabbit, #L9393, Millipore); Phalloidin-647 (1:40, A22287, Invitrogen); DAPI (1:1000, 1 mg/mL, D1306, Invitrogen); Donkey anti-Rat IgG (1:1000, Alexa Flur 488, #A48269, Invitrogen); Donkey anti-Rabbit IgG (1:1000, Alexa Flur 555, #A31572, Invitrogen); Donkey anti-Goat IgG (1:1000, Alexa Flur 555, #A-21432, Invitrogen); Donkey anti-mouse IgG (1:1000, Alexa Flur 647, #A-31571).

### Culturing of mESCs

The mouse ESCs (mESCs) used in this study is KH2 cell line^103^. The mESCs were cultured on gelatin-coated plates seeded with mitotically inactivated primary CD-1 mouse embryonic fibroblasts (MEFs). Adapted from a previously published protocol^104^, the mESC medium consisted of KnockOut DMEM (Thermo Fisher Scientific, 10829018) supplemented with 15% fetal bovine serum (FBS; Thermo Fisher Scientific, 16140071), 1 × GlutaMAX (Thermo Fisher Scientific, 35050038), 1 × non-essential amino acids (Thero Fisher Scientific, 11140035), 1:1,000 β-mercaptoethanol (Thermo Fisher Scientific, 21985023), 1 × penicillin–streptomycin (Thermo Fisher Scientific, 15140122), and laboratory-purified recombinant LIF. Cells were passaged every 2–3 days by washing twice with Dulbecco’s phosphate-buffered saline (DPBS; Thermo Fisher Scientific, 4190144), dissociating with 1 × TrypLE Express (Thermo Fisher Scientific, 12604021), and replating onto MEFs. Culture medium was replaced daily.

### Direct neural fate specification from mESCs

We adapted and simplified a previously reported serum-free, feeder-free, low-density culture protocol sufficient for neural differentiation of mESCs. To deplete feeder cells, cultures were dissociated and resuspended in mESC medium and incubated for 30 min at 37 °C, allowing MEFs to adhere. The supernatant containing mESCs was collected and used as the KH2 mESC control. Cells were then plated at a density of 100 cells µl⁻¹ (2 × 10⁵ cells per well of a 6-well plate) on plates coated with laminin (1 µg cm⁻²; Sigma-Aldrich, L2020). Cultures were divided into three experimental groups and maintained under either hypoxic (2% O₂) or normoxic (21% O₂) conditions, with three biological replicates per condition. **Group 1 (N2B27):** N2B27 basal medium consisting of 50% DMEM/F12 (Gibco, 31330-038) and 50% Neurobasal medium (Cell Applications, M812-250), supplemented with 0.5 × N2 (Gibco, 17502048), 0.5× B27 (Gibco, 17504044), 1 × GlutaMAX (Thermo Fisher Scientific, 35050038), 1 × non-essential amino acids (Thermo Fisher Scientific, 11140035), 0.1 mM β-mercaptoethanol (Thermo Fisher Scientific, 21985023), and 0.1% BSA (Sigma-Aldrich, A8412). **Group 2 (Activin A):** N2B27 basal medium supplemented with 20 ng ml⁻¹ Activin A (Thermo Fisher Scientific, AF-100-14E). **Group 3 (SB431542):** N2B27 basal medium supplemented with 10 µM SB431542 (Biozol, FBM-10-2443). Cells were cultured for 3 days with daily medium changes and monitored daily for morphological changes.

Gene expression analyses of neural and non-neural lineage markers were performed using quantitative RT–PCR (qRT–PCR). Total RNA was extracted using the Quick-RNA Microprep Kit (Zymo Research, D3020). For each sample, 1 µg of RNA was reverse-transcribed using the RevertAid First Strand cDNA Synthesis Kit (Thermo Fisher Scientific, K1622). The resulting cDNA was diluted 1:200, and 0.5 µl was used per reaction for quantitative PCR with PowerUp SYBR Green Master Mix (Thermo Fisher Scientific, A25777). qRT–PCR was performed on a QuantStudio 7 Real-Time PCR System (Thermo Fisher Scientific). Primer sequences are listed in Supplementary information and were obtained either from PrimerBank^105^ (http://www.pga.mgh.harvard.edu/primerbank/index.html) or from previously published studies.

### Roller culture

Similar to previous reports^99,106^, we used an in-house–built roller culture system equipped with a gas regulation module. Dissected embryos, with the yolk sac gently perforated using forceps, were cultured in rotating bottles containing 75% rat serum in DMEM/F12 medium. The culture medium was equilibrated with the appropriate gas mixture and temperature for at least 1 h before embryos were introduced into the system.

### Pre-processing, cell-to-embryo assignment and sex determination

The scRNA-seq data was primarily processed as previously described^22^. Supplementary Fig. 1a shows a schematic diagram of the general data handling. Briefly, the Cell Ranger pipeline version 3 (10x Genomics Inc., Cell Ranger v6.0.2^107^) was used for each scRNA-seq data set to de-multiplex the raw base call files, generate the fastq files, perform the alignment against the mouse reference genome mm10, filter the alignment and count barcodes and UMIs. Outputs from multiple sequencing runs were also combined using Cell Ranger functions.

For the cell-to-embryo assignment, as described previously^22^, the scRNA-seq data were aligned against an mm10 hybrid mouse genome assembly using STAR v.2.5.3^108^ with default settings and ‘-outSAMattributes NH HI NM MD’. The hybrid genome was prepared using SNPsplit v0.3.4^109^ to mask SNPs between the C57BL/6 J (mouse version mm10, GRCm38) and the CAST/EiJ strain genomes with the ambiguity base (N). Subsequently, SNPsplit was used to sort reads that cover SNPs by origin (reference genome). We then counted the read counts assigned to two genotypes for each chromosome for a single cell, which was transformed into a cell-by-chromosome allelic read fraction matrix. Single cells were assigned to embryos according to the autosomal fraction of CAST SNPs. k-means clustering for multiple k=number of pooled embryos deconvoluted cells into embryo clusters based on the minimum distance in their SNP profile.

For embryo-to-sex assignment, we computed linearly normalized UMI counts for Xist and chromosome Y–linked genes (e.g., Ddx3y, Eif2s3y). The XX rate was defined as the ratio of Xist expression to the sum of Xist expression and the mean expression of chrY genes; embryos with an XX rate greater than 50% were classified as female, and all others as male.

### Quality control and doublets removal

The unfiltered gene expression matrix was passed to CellBender v0.3.0^110^ to remove the ambient RNA counts.

To remove low-quality cells, we calculated the median absolute deviation (MAD) of log-transformed UMI counts across single cells and defined the minimum UMI count threshold as: median(UMI count) – 1.5 x MAD.

If this value was below 2000, a fixed threshold of 2000 UMIs was applied. In cases where inspection of the UMI count histogram indicated abnormal distributions, the threshold was manually adjusted.

For doublet and multiplet removal, we used Scrublet v0.2.3^111^. The expected doublet rate was set to 7.5%, and putative multiplets were identified using the scrub_doublets function with parameters min_gene_variability_pctl = 85 and n_prin_comps = 20. Doublet score distributions were inspected manually; when automatic thresholding failed to separate a bimodal distribution, a fixed doublet score cutoff of 0.25 was applied to retain singlet cells.

### Data processing and clustering of WT scRNA-seq atlas

The filtered gene-barcode matrices generated by Cell Ranger were processed using Seurat v5.1.0^112^ for data normalization, dimensionality reduction, and clustering. Data normalization was performed using the global scaling “LogNormalize”. For dimensionality reduction, Principal Component Analysis (PCA) was applied, followed by the generation of a Uniform Manifold Approximation and Projection (UMAP) using the first 50 principal components, with no obvious batch effects detected.

Cluster determination for the WT reference single-cell atlas was primarily conducted using Seurat with default settings. Initially, a preliminary set of clusters was created by clustering the merged datasets, separating the main germ layers (Blood, XEcto, Endo, XEndo, Meso, Epiblast, Ecto). Subsequently, per-stage clusters were generated according to this preliminary assignment and annotated based on the enrichment of manually curated stage-specific marker genes (**Source Data**). Clusters annotated as representing the same state were then merged to ensure coherent and accurate stage-specific cluster identification.

Shadow clusters were defined as low-density or peripheral cell populations in the embedding space that appear adjacent to major clusters but do not represent distinct, well-separated biological cell types, often exhibiting transcriptional similarity to their corresponding main clusters and reflecting technical noise, incomplete lysis, partial cell states, or doublet-associated signals. Shadow cluster detection was performed separately for each GEM well by identifying outlier clusters characterized by low UMI counts and mixed cell-state identities based on transferred primary annotations. After validation and removal of these shadow clusters, datasets were merged, and the clustering and annotation procedures described above were iteratively repeated.

*Lefty* mutant scRNA-seq data were preprocessed using the same pipeline as the WT reference dataset. For cell state annotation, Seurat’s ‘FindTransferAnchors’ and ‘TransferData’ functions were used to transfer state labels from the WT reference to the *Lefty* mutant dataset. For the UMAP projection, the ‘MapQuery’ function was applied to project the mutant cells onto the WT reference embedding.

### Cut site analysis

As previously published^22^, cut site analysis was conducted on the sorted BAM file to assess the reads spanning the sgRNA protospacer sequences, providing insights into the efficiency of the CRISPR-Cas9-mediated disruption process (Supplementary Fig. 2a). Single reads covering the targeted genes were extracted from the initial alignment and were realigned against the intron-free cDNA sequence of the respective gene using STAR with default settings and ‘–alignEnd- sType EndToEnd–outSAMattributes NH HI NM MD’. These reads were categorized into distinct groups based on their characteristics: 1) ‘mismatched’ encompasses reads where at least one base is a mismatch, insertion, or deletion about the reference sequence, 2) ‘spliced/deleted’ includes split reads that spanned the protospacer sequence non-continuously, 3) ‘insufficient’ are those that did not span the entire cut site, signifying a potential limitation in coverage, and 4) ‘complete’ consists of reads that mapped seamlessly to the cut site without any mismatches.

### Inferring NODAL target genes from re-analyzing ChIP-seq data and RNA-seq data

For ChIP-seq, two independent experiments are included, GSE70486 and GSE125116. For each dataset, downloaded FASTQ reads were mapped to the mouse genome mm10 (GRCm38) with Bowtie2 v2.4.2^113^ with default filtering criteria. The resulting SAM files were converted to BAM files and sorted and indexed through SAMtools v1.16^114^. Peaking calling from ChIP-Seq data was performed with MACS v2.2.7.1^115^. The parameters for peak calling included fold change > 8 and *p*-value < 1e-8 to detect high-confidence binding events. Input samples were used as reference controls for background correction. Peak region filtering was performed with the intersect function from Bedtools v2.30.0^116^ to filter the peaks that overlap in two experiments across different conditions (Activin-treatment and SB431542-treatment). Filtered Peaks are annotated with HOMER v4.11^117^ using annotatePeaks.pl function. Genes are assigned with the “nearest TSS” criteria (**Source Data**). For motif enrichment analysis, we used the findMotifsGenome.pl function in HOMER, applying a *P*-value cutoff of 1 × 10⁻⁵ and a fold-change threshold of 2 to identify significantly enriched motifs among NODAL target genes (**Source Data**).

For RNA-seq data, Fastq files downloaded from GSE125116 were quality assessed by TrimGalore v0.6.7 and mapped to the mouse genome mm39 (GRCm39) with STAR v2.7.7a using standard settings for paired reads. Read counts were normalized by library size through the R package DESeq2 v1.34.0^118^ deposited in Bioconductor. Differential gene expression analysis between any two conditions was performed based on a model utilizing the negative binomial distribution. Genes with FDR < 0.05, fold change > 1.5 or < 0.5, and average normalized read counts > 10 were called differentially expressed genes unless otherwise indicated. Basic statistical calculations were done in R v4.1.2. Potential NODAL target genes were obtained through intersecting significantly downregulated genes with genes derived from activin-treatment ChIP-seq data, then removing genes that exist in genes derived from peaks of SB431542 ChIP-seq data.

NODAL pathway activity scores were calculated using the AddModuleScore function in Seurat, with default parameters except that the number of control genes was set to 10.

For Gene Ontology analysis, genes adjacent to peaks of interest were used as input for clusterProfiler 4.14.6^119^ or the web tool of AmiGO2 (https://amigo.geneontology.org/amigo/landing) and results were plotted as bar graphs with fold change and adjusted *p*-value using R package ggplot2 (v3.4.4).

### Deconvolution of cell states contribution of GEO-seq sample using CIBERSORTx

In preprocessing the GEO-seq data (GSE171588)^24^, the provided log FPKM table was transformed into TPM to satisfy the normalization requirements of CIBERSORTx^26^. Subsequently, the CIBERSORTx web tool (https://cibersortx.stanford.edu/) was utilized following the provided tutorial. For the generation of the single-cell matrix reference sample file, extra-embryonic states (such as Blood and XEcto) were excluded, and samples from the E6.5-E7.5 stage were selected, followed by normalization based on total counts with the cell. During the ‘Create Signature Matrix’ step, parameters were set as ‘Replicates = 20, Fraction = 0.8’, with other settings left at default values. In the subsequent ‘Impute Cell Fractions’ phase, absolute imputation of cell fractions was conducted, with a permutation value of 100 sets for significance analysis. Unless otherwise specified, default parameters were used or set as suggested by CIBERSORTx.

For corn plot visualization, the embedding of GEO-seq RNA-seq datasets was generated following the descriptions in a previous publication^24^.

### Embryo ordering and age group assignment

In this study, we adapted embryo ordering algorithms proposed by Mittnenzweig et al. with modification^32^. Instead of cell-cell similarities, the intrinsic temporal ordering of embryos is inferred using analysis of the embryo-embryo similarities and optimization of the linear ordering of embryos by minimizing the temporal distance between similar embryos. To ensure the integrity of our analysis and to avoid potential biases associated with the sometimes-unstable sampling of extra-embryonic tissues and the hematopoietic lineage, we meticulously removed any residual cells corresponding to the extraembryonic endoderm, extraembryonic ectoderm, and hematopoietic lineage before conducting further analysis. Following filtering, the cosine similarity between embryo-level cell-state composition vectors was computed. Embryos were then ordered using an iterative procedure resembling a bubble-sort algorithm, in which adjacent embryos were swapped if the exchange resulted in a reduction of a predefined goal function. The goal function was defined as the sum of pairwise dissimilarities between adjacent embryos, thereby minimizing discontinuities in cell-state composition along the ordered sequence. This iterative process was continued until a local minimum was reached. The resulting ordering closely matched the actual sampling times and was therefore assigned as the EmbRank, providing a refined developmental ranking for downstream analyses.

### Metacell analysis

In the process of feature gene selection for Metacell (MC) analysis, we initially excluded genes associated with the cell cycle and stress through manual annotation. Metacells were generated separately for each sampling pool. For each pooled GEM well, we initially identified approximately 3000 highly variable genes. MCs were then constructed using the generate_mc function adapted from previous studies^32,120^, with parameters set to *Knn* = 100, *Knn_core* = 30, *min_mc_sz* = 15, and *T_vm* = 0.1. This procedure includes standard bootstrapping, MC splitting, and outlier filtering steps. The resulting reference models comprised 1542, 704, and 399 MCs for the WT, *Lefty1*KO, and *Lefty2*KO datasets, respectively, representing 122,423, 53,183, and 33,083 cells after excluding 4654, 5483, and 2758 outlier cells from downstream analyses. Metacells were annotated according to the dominant single-cell state represented within each MC.

### Differential gene expression analysis (DEG analysis) of scRNA-seq data

To minimize false-positive signals inherent to single-cell RNA-seq–based differential expression analyses, we leveraged the metacell (MC) framework, which yields RNA-seq–like expression profiles by aggregating transcript counts across transcriptionally coherent cells and thereby substantially reduces data sparsity. Differential gene expression analysis was performed on aggregated MC counts using DESeq2 with default parameters. Pseudogenes and blood-related genes were excluded from the analysis. Significantly differentially expressed genes were defined using an adjusted *P* value threshold of 1 × 10⁻³ and an absolute log₂ fold-change cutoff of 2.

### Non-negative matrix factorization (NMF) of NODAL module

To streamline the NMF analysis, we first aggregated counts across metacells (MCs) and selected those exhibiting high NODAL target expression. These MCs were then normalized, and non-negative matrix factorization (NMF) was performed using the runNMF function from the Singlet v0.0.99^121^. We conducted a parameter sweep with ranks *k* = 2 to 10, and the optimal rank *k* = 5 was determined based on the cophenetic correlation coefficient calculated across all 314 NODAL target genes, using three replicate runs for each k.

### Transcription factor (TF) network analysis

Transcription factors (TFs) inferred from motif enrichment analysis of NODAL target genes were subjected to co-expression network analysis. Normalized expression values of these TFs were extracted from the metacell (MC) expression matrix. A weighted gene co-expression network was constructed using the WGCNA framework^122^ with a signed network topology and spearman correlation.

An appropriate soft-thresholding power was determined using the pickSoftThreshold function by assessing scale-free topology criteria across powers ranging from 1 to 20. The resulting adjacency matrix was computed using the selected soft-thresholding power, and self-connections were removed. Weak correlations (adjacency < 0.01) were filtered out to reduce spurious edges.

The filtered adjacency matrix was converted into an undirected, weighted graph using igraph 1.0.1^123^. Community structure was identified using the fast greedy modularity optimization algorithm, which accounts for edge weights. Node degree was calculated and used to scale node size in network visualizations, while edge weights correspond to TF–TF co-expression strength.

To relate TF activity to NODAL transcriptional programs, spearman correlations between TF expression and non-negative matrix factorization (NMF) module embeddings were computed. These TF–NMF correlations were added as node attributes, enabling visualization of TF usage across distinct NODAL-responsive transcriptional modules. Networks were exported in GraphML format for downstream Cytoscape 3.10.4 visualization and analysis.

### Reporting summary

Further information on research design is available in the Nature Portfolio Reporting Summary linked to this article.

## Supplementary information

Supplementary Information

Peer Review file

Reporting Summary

## Source data

Source Data

## Data Availability

The *Lefty* mutant data and wild type data generated in this study have been deposited in the Gene Expression Omnibus (GEO) database under accession codes GSE272574 and GSE266636. The ChIP-seq data and RNA-seq data used in this study are available in the GEO database under accession codes GSE70486 and GSE125116. Source data are provided in this paper.

## Code availability

Codes for generating figures in the manuscript have been deposited at https://github.com/gezi81889/Nodal_antagonism_script.

## References

[1] Bardot, E. S. & Hadjantonakis, A. K. Mouse gastrulation: Coordination of tissue patterning, specification and diversification of cell fate. *Mech. Dev.* **163**, 103617 (2020). 10.1016/j.mod.2020.103617 PMC7534585 32473204

[2] Green, J. B. & Smith, J. C. Growth factors as morphogens: do gradients and thresholds establish body plan? *Trends Genet.* **7**, 245–250 (1991). 10.1016/0168-9525(91)90323-I 1685268

[3] Wolpert, L. Positional information and the spatial pattern of cellular differentiation. *J. Theor. Biol.* **25**, 1–47 (1969). 10.1016/s0022-5193(69)80016-0 4390734

[4] Juan, H. & Hamada, H. Roles of nodal-lefty regulatory loops in embryonic patterning of vertebrates. *Genes Cells* **6**, 923–930 (2001). 10.1046/j.1365-2443.2001.00481.x 11733030

[5] Branford, W. W. & Yost, H. J. Lefty-dependent inhibition of Nodal- and Wnt-responsive organizer gene expression is essential for normal gastrulation. *Curr. Biol.* **12**, 2136–2141 (2002). 10.1016/s0960-9822(02)01360-x 12498689

[6] Meno, C. et al. Diffusion of nodal signaling activity in the absence of the feedback inhibitor Lefty2. *Dev. Cell* **1**, 127–138 (2001). 10.1016/s1534-5807(01)00006-5 11703930

[7] Chen, C. & Shen, M. M. Two modes by which Lefty proteins inhibit nodal signaling. *Curr. Biol.* **14**, 618–624 (2004). 10.1016/j.cub.2004.02.042 15062104

[8] Sakuma, R. et al. Inhibition of Nodal signalling by Lefty mediated through interaction with common receptors and efficient diffusion. *Genes Cells* **7**, 401–412 (2002). 10.1046/j.1365-2443.2002.00528.x 11952836

[9] Nakao, A. et al. TGF-beta receptor-mediated signalling through Smad2, Smad3 and Smad4. *EMBO J.* **16**, 5353–5362 (1997). 10.1093/emboj/16.17.5353 PMC1170167 9311995

[10] Norris, D. P. et al. The Foxh1-dependent autoregulatory enhancer controls the level of Nodal signals in the mouse embryo. *Development* **129**, 3455–3468 (2002). 10.1242/dev.129.14.3455 12091315

[11] Dickmeis, T. et al. Identification of nodal signaling targets by array analysis of induced complex probes. *Dev. Dyn.* **222**, 571–580 (2001). 10.1002/dvdy.1220 11748827

[12] Meno, C. et al. Two closely-related left-right asymmetrically expressed genes, lefty-1 and lefty-2: their distinct expression domains, chromosomal linkage and direct neuralizing activity in Xenopus embryos. *Genes Cells* **2**, 513–524 (1997). 10.1046/j.1365-2443.1997.1400338.x 9348041

[13] Varlet, I., Collignon, J. & Robertson, E. J. nodal expression in the primitive endoderm is required for specification of the anterior axis during mouse gastrulation. *Development* **124**, 1033–1044 (1997). 10.1242/dev.124.5.1033 9056778

[14] Yamamoto, M. et al. Nodal antagonists regulate formation of the anteroposterior axis of the mouse embryo. *Nature* **428**, 387–392 (2004). 10.1038/nature02418 15004567

[15] Meno, C. et al. Mouse Lefty2 and zebrafish antivin are feedback inhibitors of nodal signaling during vertebrate gastrulation. *Mol. Cell* **4**, 287–298 (1999). 10.1016/s1097-2765(00)80331-7 10518210

[16] Lu, C. C. & Robertson, E. J. Multiple roles for Nodal in the epiblast of the mouse embryo in the establishment of anterior-posterior patterning. *Dev. Biol.* **273**, 149–159 (2004). 10.1016/j.ydbio.2004.06.004 15302604

[17] Kumar, A. et al. Nodal signaling from the visceral endoderm is required to maintain Nodal gene expression in the epiblast and drive DVE/AVE migration. *Dev. Biol.* **400**, 1–9 (2015). 10.1016/j.ydbio.2014.12.016 PMC4806383 25536399

[18] Brennan, J. et al. Nodal signalling in the epiblast patterns the early mouse embryo. *Nature* **411**, 965–969 (2001). 10.1038/35082103 11418863

[19] Vincent, S. D. et al. Cell fate decisions within the mouse organizer are governed by graded Nodal signals. *Genes Dev.* **17**, 1646–1662 (2003). 10.1101/gad.1100503 PMC196136 12842913

[20] Perea-Gomez, A. et al. Nodal antagonists in the anterior visceral endoderm prevent the formation of multiple primitive streaks. *Dev. Cell* **3**, 745–756 (2002). 10.1016/s1534-5807(02)00321-0 12431380

[21] Andergassen, D. et al. Diverse epigenetic mechanisms maintain parental imprints within the embryonic and extraembryonic lineages. *Dev. Cell* **56**, 2995–3005 (2021). 10.1016/j.devcel.2021.10.010 PMC9463566 34752748

[22] Grosswendt, S. et al. Epigenetic regulator function through mouse gastrulation. *Nature* **584**, 102–108 (2020). 10.1038/s41586-020-2552-x PMC7415732 32728215

[23] Trichas, G. et al. Nodal dependent differential localisation of dishevelled-2 demarcates regions of differing cell behaviour in the visceral endoderm. *PLoS Biol.* **9**, e1001019 (2011). 10.1371/journal.pbio.1001019 PMC3042994 21364967

[24] Wang, R. et al. Time space and single-cell resolved tissue lineage trajectories and laterality of body plan at gastrulation. *Nat. Commun.* **14**, 5675 (2023). 10.1038/s41467-023-41482-5 PMC10502153 37709743

[25] Peng, G. et al. Molecular architecture of lineage allocation and tissue organization in early mouse embryo. *Nature* **572**, 528–532 (2019). 10.1038/s41586-019-1469-8 31391582

[26] Newman, A. M. et al. Determining cell type abundance and expression from bulk tissues with digital cytometry. *Nat. Biotechnol.* **37**, 773–782 (2019). 10.1038/s41587-019-0114-2 PMC6610714 31061481

[27] Robb, L. & Tam, P. P. Gastrula organiser and embryonic patterning in the mouse. *Semin Cell Dev. Biol.* **15**, 543–554 (2004). 10.1016/j.semcdb.2004.04.005 15271300

[28] Schubert, F. R. et al. Expression of the novel murine homeobox gene Sax-1 in the developing nervous system. *Mech. Dev.* **51**, 99–114 (1995). 10.1016/0925-4773(95)00358-8 7669696

[29] Lewis, S. L. et al. Genetic interaction of Gsc and Dkk1 in head morphogenesis of the mouse. *Mech. Dev.* **124**, 157–165 (2007). 10.1016/j.mod.2006.10.001 17127040

[30] Kimura, C. et al. Complementary functions of Otx2 and Cripto in initial patterning of mouse epiblast. *Dev. Biol.* **235**, 12–32 (2001). 10.1006/dbio.2001.0289 11412024

[31] Tzouanacou, E. et al. Redefining the progression of lineage segregations during mammalian embryogenesis by clonal analysis. *Dev. Cell* **17**, 365–376 (2009). 10.1016/j.devcel.2009.08.002 19758561

[32] Mittnenzweig, M. et al. A single-embryo, single-cell time-resolved model for mouse gastrulation. *Cell* **184**, 2825–2842 (2021). 10.1016/j.cell.2021.04.004 PMC8162424 33932341

[33] Perea-Gomez, A. et al. Initiation of gastrulation in the mouse embryo is preceded by an apparent shift in the orientation of the anterior-posterior axis. *Curr. Biol.* **14**, 197–207 (2004). 10.1016/j.cub.2004.01.030 14761651

[34] Kwon, G. S., Viotti, M. & Hadjantonakis, A. K. The endoderm of the mouse embryo arises by dynamic widespread intercalation of embryonic and extraembryonic lineages. *Dev. Cell* **15**, 509–520 (2008). 10.1016/j.devcel.2008.07.017 PMC2677989 18854136

[35] Bellomo, D. et al. Cell proliferation in mammalian gastrulation: the ventral node and notochord are relatively quiescent. *Dev. Dyn.* **205**, 471–485 (1996). 10.1002/(SICI)1097-0177(199604)205:4<471::AID-AJA10>3.0.CO;2-4 8901057

[36] Yamanaka, Y. et al. Live imaging and genetic analysis of mouse notochord formation reveals regional morphogenetic mechanisms. *Dev. Cell* **13**, 884–896 (2007). 10.1016/j.devcel.2007.10.016 18061569

[37] Gredler, M. L. & Zallen, J. A. Multicellular rosettes link mesenchymal-epithelial transition to radial intercalation in the mouse axial mesoderm. *Dev. Cell* **58**, 933–950 (2023). 10.1016/j.devcel.2023.03.018 PMC10247533 37080203

[38] Tyser, R.C.V. et al. Characterization of a common progenitor pool of the epicardium and myocardium. *Science* **371**, 10.1126/science.abb2986. PMC7615359 33414188

[39] Lowe, L. A., Yamada, S. & Kuehn, M. R. Genetic dissection of nodal function in patterning the mouse embryo. *Development* **128**, 1831–1843 (2001). 10.1242/dev.128.10.1831 11311163

[40] Camus, A. et al. Absence of Nodal signaling promotes precocious neural differentiation in the mouse embryo. *Dev. Biol.* **295**, 743–755 (2006). 10.1016/j.ydbio.2006.03.047 16678814

[41] Mathis, L. & Nicolas, J. F. Different clonal dispersion in the rostral and caudal mouse central nervous system. *Development* **127**, 1277–1290 (2000). 10.1242/dev.127.6.1277 10683180

[42] Acampora, D. et al. Forebrain and midbrain regions are deleted in Otx2-/- mutants due to a defective anterior neuroectoderm specification during gastrulation. *Development* **121**, 3279–3290 (1995). 10.1242/dev.121.10.3279 7588062

[43] Cajal, M. et al. Clonal and molecular analysis of the prospective anterior neural boundary in the mouse embryo. *Development* **139**, 423–436 (2012). 10.1242/dev.075499 PMC3243100 22186731

[44] & Brivanlou, A. H. Neural induction and early patterning in vertebrates. *Wiley Interdiscip. Rev. Dev. Biol.* **2**, 479–498 (2013). 10.1002/wdev.90 PMC6945772 24014419

[45] Tropepe, V. et al. Direct neural fate specification from embryonic stem cells: a primitive mammalian neural stem cell stage acquired through a default mechanism. *Neuron* **30**, 65–78 (2001). 10.1016/s0896-6273(01)00263-x 11343645

[46] Lopez-Anguita, N. et al. Hypoxia induces an early primitive streak signature, enhancing spontaneous elongation and lineage representation in gastruloids. *Development* **149**, 10.1242/dev.200679 (2022). PMC9578691 36102628

[47] Liu, L. et al. Nodal is a short-range morphogen with activity that spreads through a relay mechanism in human gastruloids. *Nat. Commun.* **13**, 497 (2022). 10.1038/s41467-022-28149-3 PMC8789905 35079017

[48] Jin, S. et al. Inference and analysis of cell-cell communication using CellChat. *Nat. Commun.* **12**, 1088 (2021). 10.1038/s41467-021-21246-9 PMC7889871 33597522

[49] Cabello-Aguilar, S. et al. SingleCellSignalR: inference of intercellular networks from single-cell transcriptomics. *Nucleic Acids Res.* **48**, e55 (2020). 10.1093/nar/gkaa183 PMC7261168 32196115

[50] Aragon, E. et al. Structural basis for distinct roles of SMAD2 and SMAD3 in FOXH1 pioneer-directed TGF-beta signaling. *Genes Dev.* **33**, 1506–1524 (2019). 10.1101/gad.330837.119 PMC6824466 31582430

[51] Wang, Q. et al. The p53 Family coordinates Wnt and nodal inputs in mesendodermal differentiation of embryonic stem cells. *Cell Stem Cell* **20**, 70–86 (2017). 10.1016/j.stem.2016.10.002 PMC5218926 27889317

[52] Chen, X. et al. Smad4 and FAST-1 in the assembly of activin-responsive factor. *Nature* **389**, 85–89 (1997). 10.1038/38008 9288972

[53] Labbe, E. et al. Smad2 and Smad3 positively and negatively regulate TGF beta-dependent transcription through the forkhead DNA-binding protein FAST2. *Mol. Cell* **2**, 109–120 (1998). 10.1016/s1097-2765(00)80119-7 9702197

[54] Saijoh, Y. et al. Left-right asymmetric expression of lefty2 and nodal is induced by a signaling pathway that includes the transcription factor FAST2. *Mol. Cell* **5**, 35–47 (2000). 10.1016/s1097-2765(00)80401-3 10678167

[55] Shiratori, H. et al. Two-step regulation of left-right asymmetric expression of Pitx2: initiation by nodal signaling and maintenance by Nkx2. *Mol. Cell* **7**, 137–149 (2001). 10.1016/s1097-2765(01)00162-9 11172719

[56] Kong, X. et al. LncRNA-Smad7 mediates cross-talk between Nodal/TGF-beta and BMP signaling to regulate cell fate determination of pluripotent and multipotent cells. *Nucleic Acids Res.* **50**, 10526–10543 (2022). 10.1093/nar/gkac780 PMC9561265 36134711

[57] Katoh, M. & Katoh, M. CER1 is a common target of WNT and NODAL signaling pathways in human embryonic stem cells. *Int J. Mol. Med.* **17**, 795–799 (2006). 16596263

[58] Simon, C. S. et al. Functional characterisation of cis-regulatory elements governing dynamic Eomes expression in the early mouse embryo. *Development* **144**, 1249–1260 (2017). 10.1242/dev.147322 PMC5399628 28174238

[59] Sun, L. T. et al. Nanog co-regulated by Nodal/Smad2 and Oct4 is required for pluripotency in developing mouse epiblast. *Dev. Biol.* **392**, 182–192 (2014). 10.1016/j.ydbio.2014.06.002 24929238

[60] Hart, A. H. et al. Transcriptional regulation of the homeobox gene Mixl1 by TGF-beta and FoxH1. *Biochem. Biophys. Res. Commun.* **333**, 1361–1369 (2005). 10.1016/j.bbrc.2005.06.044 15982639

[61] Vallier, L., Alexander, M. & Pedersen, R. A. Activin/Nodal and FGF pathways cooperate to maintain pluripotency of human embryonic stem cells. *J. Cell Sci.* **118**, 4495–4509 (2005). 10.1242/jcs.02553 16179608

[62] Osnato, A. et al. TGFbeta signalling is required to maintain pluripotency of human naive pluripotent stem cells. *Elife* **10**, 10.7554/elife.67259 (2021). PMC8410071 34463252

[63] Schnirman, R. E. et al. The role of Wnt signaling in the development of the epiblast and axial progenitors. *Curr. Top. Dev. Biol.* **153**, 145–180 (2023). 10.1016/bs.ctdb.2023.01.010 36967193

[64] Hoodless, P. A. et al. FoxH1 (Fast) functions to specify the anterior primitive streak in the mouse. *Genes Dev.* **15**, 1257–1271 (2001). 10.1101/gad.881501 PMC313796 11358869

[65] Ding, J. et al. Cripto is required for correct orientation of the anterior-posterior axis in the mouse embryo. *Nature* **395**, 702–707 (1998). 10.1038/27215 9790191

[66] Sun, X. et al. Targeted disruption of Fgf8 causes failure of cell migration in the gastrulating mouse embryo. *Genes Dev.* **13**, 1834–1846 (1999). 10.1101/gad.13.14.1834 PMC316887 10421635

[67] Shimono, A. & Behringer, R. R. Angiomotin regulates visceral endoderm movements during mouse embryogenesis. *Curr. Biol.* **13**, 613–617 (2003). 10.1016/s0960-9822(03)00204-5 12676095

[68] Belo, J. A. et al. Cerberus-like is a secreted factor with neutralizing activity expressed in the anterior primitive endoderm of the mouse gastrula. *Mech. Dev.* **68**, 45–57 (1997). 10.1016/s0925-4773(97)00125-1 9431803

[69] Murry, C. E. & Keller, G. Differentiation of embryonic stem cells to clinically relevant populations: lessons from embryonic development. *Cell* **132**, 661–680 (2008). 10.1016/j.cell.2008.02.008 18295582

[70] Mesnard, D., Guzman-Ayala, M. & Constam, D. B. Nodal specifies embryonic visceral endoderm and sustains pluripotent cells in the epiblast before overt axial patterning. *Development* **133**, 2497–2505 (2006). 10.1242/dev.02413 16728477

[71] Cha, Y. R., Takahashi, S. & Wright, C. V. Cooperative non-cell and cell autonomous regulation of Nodal gene expression and signaling by Lefty/Antivin and Brachyury in Xenopus. *Dev. Biol.* **290**, 246–264 (2006). 10.1016/j.ydbio.2005.10.043 16405884

[72] Zhang, H. et al. Interplay between Lefty and Nodal signaling is essential for the organizer and axial formation in amphioxus embryos. *Dev. Biol.* **456**, 63–73 (2019). 10.1016/j.ydbio.2019.08.006 31419410

[73] Agathon, A., Thisse, B. & Thisse, C. Morpholino knock-down of antivin1 and antivin2 upregulates nodal signaling. *Genesis* **30**, 178–182 (2001). 10.1002/gene.1059 11477702

[74] Rogers, K.W. et al. Nodal patterning without Lefty inhibitory feedback is functional but fragile. *Elife* **6**, 10.7554/eLife.28785 (2017). PMC5720593 29215332

[75] Meno, C. et al. lefty-1 is required for left-right determination as a regulator of lefty-2 and nodal. *Cell* **94**, 287–297 (1998). 10.1016/s0092-8674(00)81472-5 9708731

[76] Takaoka, K., Nishimura, H. & Hamada, H. Both Nodal signalling and stochasticity select for prospective distal visceral endoderm in mouse embryos. *Nat. Commun.* **8**, 1492 (2017). 10.1038/s41467-017-01625-x PMC5686177 29138408

[77] Yashiro, K. et al. Distinct transcriptional regulation and phylogenetic divergence of human LEFTY genes. *Genes Cells* **5**, 343–357 (2000). 10.1046/j.1365-2443.2000.00329.x 10886363

[78] Sagner, A. & Briscoe, J. Morphogen interpretation: concentration, time, competence, and signaling dynamics. *Wiley Interdiscip. Rev. Dev. Biol.* **6**, 10.1002/wdev.271 (2017). PMC5516147 28319331

[79] Warga, R. M. & Kimmel, C. B. Cell movements during epiboly and gastrulation in zebrafish. *Development* **108**, 569–580 (1990). 10.1242/dev.108.4.569 2387236

[80] Snow, M. H. L. Gastrulation in the mouse: Growth and regionalization of the epiblast. *Development* **42**, 293–303 (1977).

[81] Chen, Y. & Schier, A. F. Lefty proteins are long-range inhibitors of squint-mediated nodal signaling. *Curr. Biol.* **12**, 2124–2128 (2002). 10.1016/s0960-9822(02)01362-3 12498687

[82] van Boxtel, A. L. et al. A temporal window for signal activation dictates the dimensions of a nodal signaling domain. *Dev. Cell* **35**, 175–185 (2015). 10.1016/j.devcel.2015.09.014 PMC4640439 26506307

[83] Dyson, S. & Gurdon, J. B. The interpretation of position in a morphogen gradient as revealed by occupancy of activin receptors. *Cell* **93**, 557–568 (1998). 10.1016/s0092-8674(00)81185-x 9604931

[84] Hagos, E. G. & Dougan, S. T. Time-dependent patterning of the mesoderm and endoderm by Nodal signals in zebrafish. *BMC Dev. Biol.* **7**, 22 (2007). 10.1186/1471-213X-7-22 PMC1851950 17391517

[85] Vallier, L., Reynolds, D. & Pedersen, R. A. Nodal inhibits differentiation of human embryonic stem cells along the neuroectodermal default pathway. *Dev. Biol.* **275**, 403–421 (2004). 10.1016/j.ydbio.2004.08.031 15501227

[86] Valenta, T. et al. Probing transcription-specific outputs of beta-catenin in vivo. *Genes Dev.* **25**, 2631–2643 (2011). 10.1101/gad.181289.111 PMC3248684 22190459

[87] Huelsken, J. et al. Requirement for beta-catenin in anterior-posterior axis formation in mice. *J. Cell Biol.* **148**, 567–578 (2000). 10.1083/jcb.148.3.567 PMC2174807 10662781

[88] Conlon, F. L. et al. A primary requirement for nodal in the formation and maintenance of the primitive streak in the mouse. *Development* **120**, 1919–1928 (1994). 10.1242/dev.120.7.1919 7924997

[89] Miura, S., Singh, A. P. & Mishina, Y. Bmpr1a is required for proper migration of the AVE through regulation of Dkk1 expression in the pre-streak mouse embryo. *Dev. Biol.* **341**, 246–254 (2010). 10.1016/j.ydbio.2010.02.038 PMC2854289 20211162

[90] Ben-Haim, N. et al. The nodal precursor acting via activin receptors induces mesoderm by maintaining a source of its convertases and BMP4. *Dev. Cell* **11**, 313–323 (2006). 10.1016/j.devcel.2006.07.005 16950123

[91] Rodriguez, T. A. et al. Induction and migration of the anterior visceral endoderm is regulated by the extra-embryonic ectoderm. *Development* **132**, 2513–2520 (2005). 10.1242/dev.01847 15857911

[92] Soares, M. L., Torres-Padilla, M. E. & Zernicka-Goetz, M. Bone morphogenetic protein 4 signaling regulates development of the anterior visceral endoderm in the mouse embryo. *Dev. Growth Differ.* **50**, 615–621 (2008). 10.1111/j.1440-169X.2008.01059.x PMC3342679 18657169

[93] Rivera-Perez, J. A. & Magnuson, T. Primitive streak formation in mice is preceded by localized activation of Brachyury and Wnt3. *Dev. Biol.* **288**, 363–371 (2005). 10.1016/j.ydbio.2005.09.012 16289026

[94] Liu, C., Peng, G. & Jing, N. TGF-beta signaling pathway in early mouse development and embryonic stem cells. *Acta Biochim. Biophys. Sin.* **50**, 68–73 (2018). 10.1093/abbs/gmx120 29190317

[95] Morkel, M. et al. Beta-catenin regulates Cripto- and Wnt3-dependent gene expression programs in mouse axis and mesoderm formation. *Development* **130**, 6283–6294 (2003). 10.1242/dev.00859 14623818

[96] Sozen, B., Demir, N. & Zernicka-Goetz, M. BMP signalling is required for extra-embryonic ectoderm development during pre-to-post-implantation transition of the mouse embryo. *Dev. Biol.* **470**, 84–94 (2021). 10.1016/j.ydbio.2020.11.005 PMC8219371 33217407

[97] Hernandez-Martinez, R. et al. Axin1 and Axin2 regulate the WNT-signaling landscape to promote distinct mesoderm programs. Preprint at 10.1101/2024.09.11.612342 (2024). PMC13399174 42385713

[98] Tremblay, K. D. et al. Formation of the definitive endoderm in mouse is a Smad2-dependent process. *Development* **127**, 3079–3090 (2000). 10.1242/dev.127.14.3079 10862745

[99] Aguilera-Castrejon, A. et al. Ex utero mouse embryogenesis from pre-gastrulation to late organogenesis. *Nature* **593**, 119–124 (2021). 10.1038/s41586-021-03416-3 33731940

[100] Wang, H. et al. One-step generation of mice carrying mutations in multiple genes by CRISPR/Cas-mediated genome engineering. *Cell* **153**, 910–918 (2013). 10.1016/j.cell.2013.04.025 PMC3969854 23643243

[101] Shindo, M. et al. Efficient superovulation and egg collection from mice. *Bio. Protoc.* **12**, 10.21769/bioprotoc.4439 (2022). PMC9244495 35799908

[102] Wong, F. C. K. Whole-mount immunofluorescence staining of early mouse embryos. *Methods Mol. Biol.* **2214**, 143–155 (2021). 10.1007/978-1-0716-0958-3_10 32944908

[103] Beard, C. et al. Efficient method to generate single-copy transgenic mice by site-specific integration in embryonic stem cells. *Genesis* **44**, 23–28 (2006). 10.1002/gene.20180 16400644

[104] Meissner, A., Eminli, S. & Jaenisch, R. Derivation and manipulation of murine embryonic stem cells. *Methods Mol. Biol.* **482**, 3–19 (2009). 10.1007/978-1-59745-060-7_1 19089346

[105] Wang, X. and B. Seed, A PCR primer bank for quantitative gene expression analysis. *Nucleic Acids Res.* **31**, e154 (2003). 10.1093/nar/gng154 PMC291882 14654707

[106] Aguilera-Castrejon, A. & Hanna, J.H. *Highly Conducive Ex utero Mouse Embryogenesis from Pre-Gastrulation to Late Organogenesis*. (2021). 10.1038/s41586-021-03416-3 33731940

[107] Zheng, G. X. et al. Massively parallel digital transcriptional profiling of single cells. *Nat. Commun.* **8**, 14049 (2017). 10.1038/ncomms14049 PMC5241818 28091601

[108] Dobin, A. et al. STAR: ultrafast universal RNA-seq aligner. *Bioinformatics* **29**, 15–21 (2013). 10.1093/bioinformatics/bts635 PMC3530905 23104886

[109] Krueger, F. & Andrews, S. R. SNPsplit: Allele-specific splitting of alignments between genomes with known SNP genotypes. *F1000Res.* **5**, 1479 (2016). 10.12688/f1000research.9037.1 PMC4934512 27429743

[110] Fleming, J. E. et al. From dawn till dusk: Time-adaptive bayesian optimization for neurostimulation. *PLoS Comput. Biol.* **19**, e1011674 (2023). 10.1371/journal.pcbi.1011674 PMC10718444 38091368

[111] Wolock, S. L., Lopez, R. & Klein, A. M. Scrublet: Computational identification of cell doublets in single-cell transcriptomic data. *Cell Syst.* **8**, 281–291 (2019). 10.1016/j.cels.2018.11.005 PMC6625319 30954476

[112] Stuart, T. et al. Comprehensive integration of single-cell data. *Cell* **177**, 1888–1902 (2019). 10.1016/j.cell.2019.05.031 PMC6687398 31178118

[113] Langmead, B. & Salzberg, S. L. Fast gapped-read alignment with Bowtie 2. *Nat. Methods* **9**, 357–359 (2012). 10.1038/nmeth.1923 PMC3322381 22388286

[114] Danecek, P. et al. Twelve years of SAMtools and BCFtools. *Gigascience* **10**, 10.1093/gigascience/giab008 (2021). PMC7931819 33590861

[115] Zhang, Y. et al. Model-based analysis of ChIP-Seq (MACS). *Genome Biol.* **9**, R137 (2008). 10.1186/gb-2008-9-9-r137 PMC2592715 18798982

[116] Quinlan, A. R. & Hall, I. M. BEDTools: a flexible suite of utilities for comparing genomic features. *Bioinformatics* **26**, 841–842 (2010). 10.1093/bioinformatics/btq033 PMC2832824 20110278

[117] Heinz, S. et al. Simple combinations of lineage-determining transcription factors prime cis-regulatory elements required for macrophage and B cell identities. *Mol. Cell* **38**, 576–589 (2010). 10.1016/j.molcel.2010.05.004 PMC2898526 20513432

[118] Love, M. I., Huber, W. & Anders, S. Moderated estimation of fold change and dispersion for RNA-seq data with DESeq2. *Genome Biol.* **15**, 550 (2014). 10.1186/s13059-014-0550-8 PMC4302049 25516281

[119] Xu, S. et al. Using clusterProfiler to characterize multiomics data. *Nat. Protoc.* **19**, 3292–3320 (2024). 10.1038/s41596-024-01020-z 39019974

[120] Baran, Y. et al. MetaCell: analysis of single-cell RNA-seq data using K-nn graph partitions. *Genome Biol.* **20**, 206 (2019). 10.1186/s13059-019-1812-2 PMC6790056 31604482

[121] DeBruine, Z. singlet*:* Non-negative Matrix Factorization for single-cell analysis. https://github.com/zdebruine/singlet (2022).

[122] Langfelder, P. & Horvath, S. WGCNA: an R package for weighted correlation network analysis. *BMC Bioinform.* **9**, 559 (2008). 10.1186/1471-2105-9-559 PMC2631488 19114008

[123] Antonov, M. et al. igraph 1.0 enables fast and robust network analysis across programming languages. *PLoS One* **21**, e0355108 10.1371/journal.pone.0355108 (2026). PMC13436765 42550872

[124] Shen, M. M. Nodal signaling: developmental roles and regulation. *Development* **134**, 1023–1034 (2007). 10.1242/dev.000166 17287255

[125] Yamamoto, M. et al. Antagonism between Smad1 and Smad2 signaling determines the site of distal visceral endoderm formation in the mouse embryo. *J. Cell Biol.* **184**, 323–334 (2009). 10.1083/jcb.200808044 PMC2654303 19153222

[126] Popperl, H. et al. Misexpression of Cwnt8C in the mouse induces an ectopic embryonic axis and causes a truncation of the anterior neuroectoderm. *Development* **124**, 2997–3005 (1997). 10.1242/dev.124.15.2997 9247341

