# Supplementary material for: Consecutive waves of NODAL inhibition are required for epiblast patterning
Source: Nat Commun. 2026 Oct 9;17:11484. doi: 10.1038/s41467-026-78248-8 (PMC13740313; doi:10.1038/s41467-026-78248-8)
Supplement: Supplementary file 1 [file 41467_2026_78248_MOESM1_ESM.pdf]

Supplementary Information

Consecutive Waves of NODAL Inhibition Are Required for Epiblast Patterning

Minming Wang[1], Tien-Chi Jason Hou[1], JoAnne Villagrana[1], Yusuke Kijima[2], Nicolas Dias[1], Roxanna Altus[1], Martina Brueckner[1], Kaelyn Sumigray[1], Helene Kretzmer[2] and Zachary D. Smith[1,2*]

[1]Department of Genetics, Yale Stem Cell Center, Yale School of Medicine; New Haven, CT, USA
[2]Department of Genome Regulation, Max Planck Institute for Molecular Genetics; Berlin, Germany

*Corresponding authors. Email: z.smith@yale.edu

The PDF file includes:
Supplementary Figures 1 to 8
RT-qPCR primers
Personal Notes on cell state annotation

Wang et al., Supplementary Fig 1

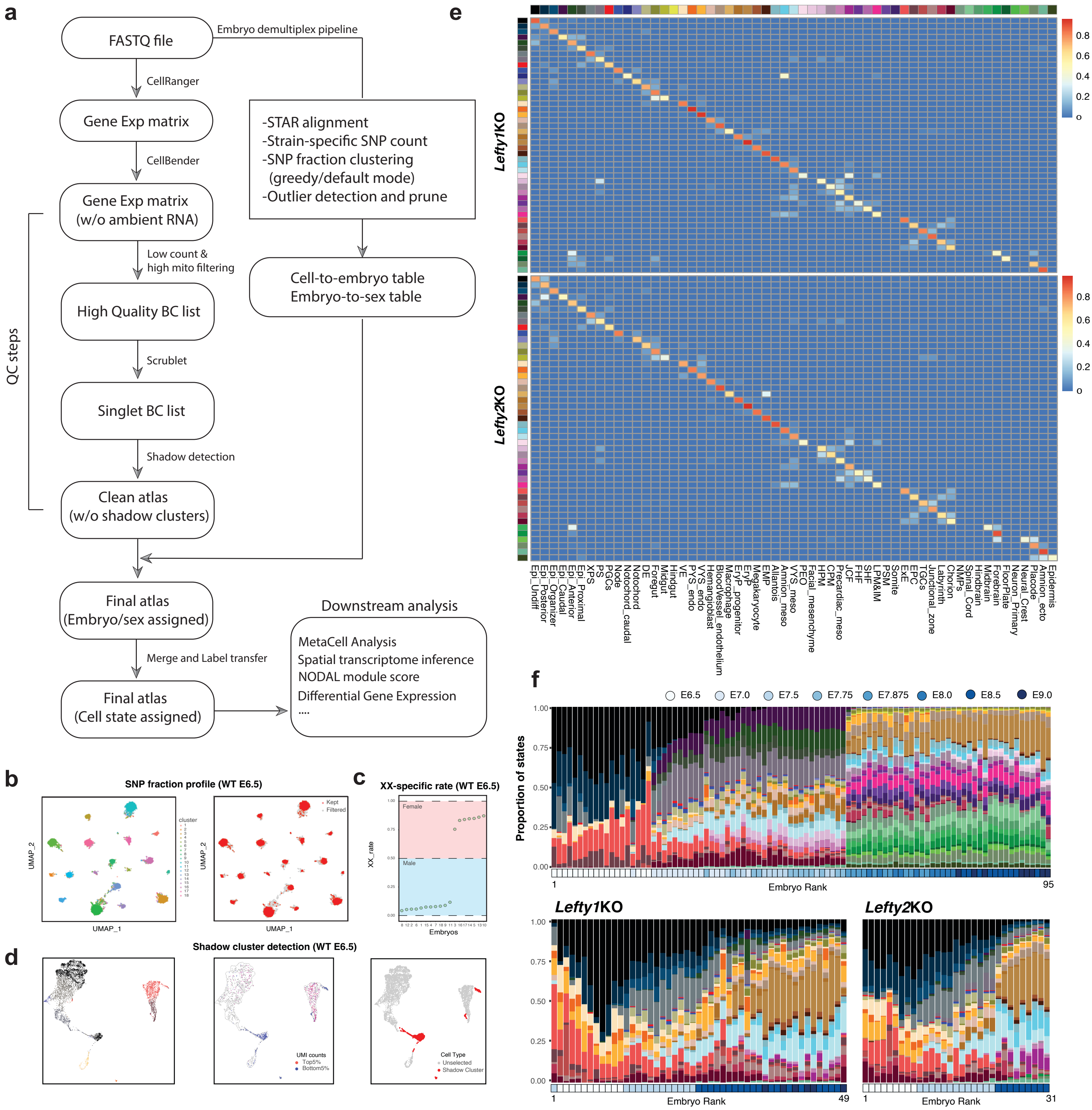

Supplementary Figure 1. Single-cell sequencing dataset process pipeline.

- **a.** Overview of the computational pipeline used in this study, highlighting key steps including embryo demultiplexing by genotype using the CAST SNP profile, correction for ambient RNA contamination, removal of shadow clusters, and cell-state label transfer.

- **b.** *Left:* Representative UMAP of SNP-typable cells from a control E6.5 clutch of 18 pooled embryos. Randomly transmitted CAST/EiJ SNP profiles serve as unique embryo barcodes, reflecting grandparental allele transmission from the male donor. *Right:* Cells retained after quality control are highlighted on the original UMAP.

- **c.** Representative XX-specific rate (**Methods**). Data is shown for our E6.5 clutch of 18 pooled embryos, comprising 11 male and 7 female embryos.

- **d.** Detection and removal of low confidence "shadow clusters" within the same E6.5 dataset, characterized by low UMI counts that confound clear cell state assignment. Cells assigned to these clusters were removed from the reference and used to ensure the recovery of high confidence single cell transcriptomes for the analysis and assembly of our data.

- **e.** Transcriptional similarity (correlation of gene expression) of states recovered between our *Lefty*KO cohorts and our control reference. Generally, well sampled cell states have a high transcriptional correlation between WT and KOs, while later gastrulation states are heavily underproduced and are poorly correlated to their WT counterparts.

- **f.** Per-embryo composition of cell types in WT (top) and *Lefty*KO (bottom) embryos, organized according to inherent transcriptomic similarity ranks (**Methods**). This ranking leverages natural variation across developmental time points to provide a more continuous representation of early gastrulation for both control and mutant conditions.

Wang et al., Supplementary Fig 2

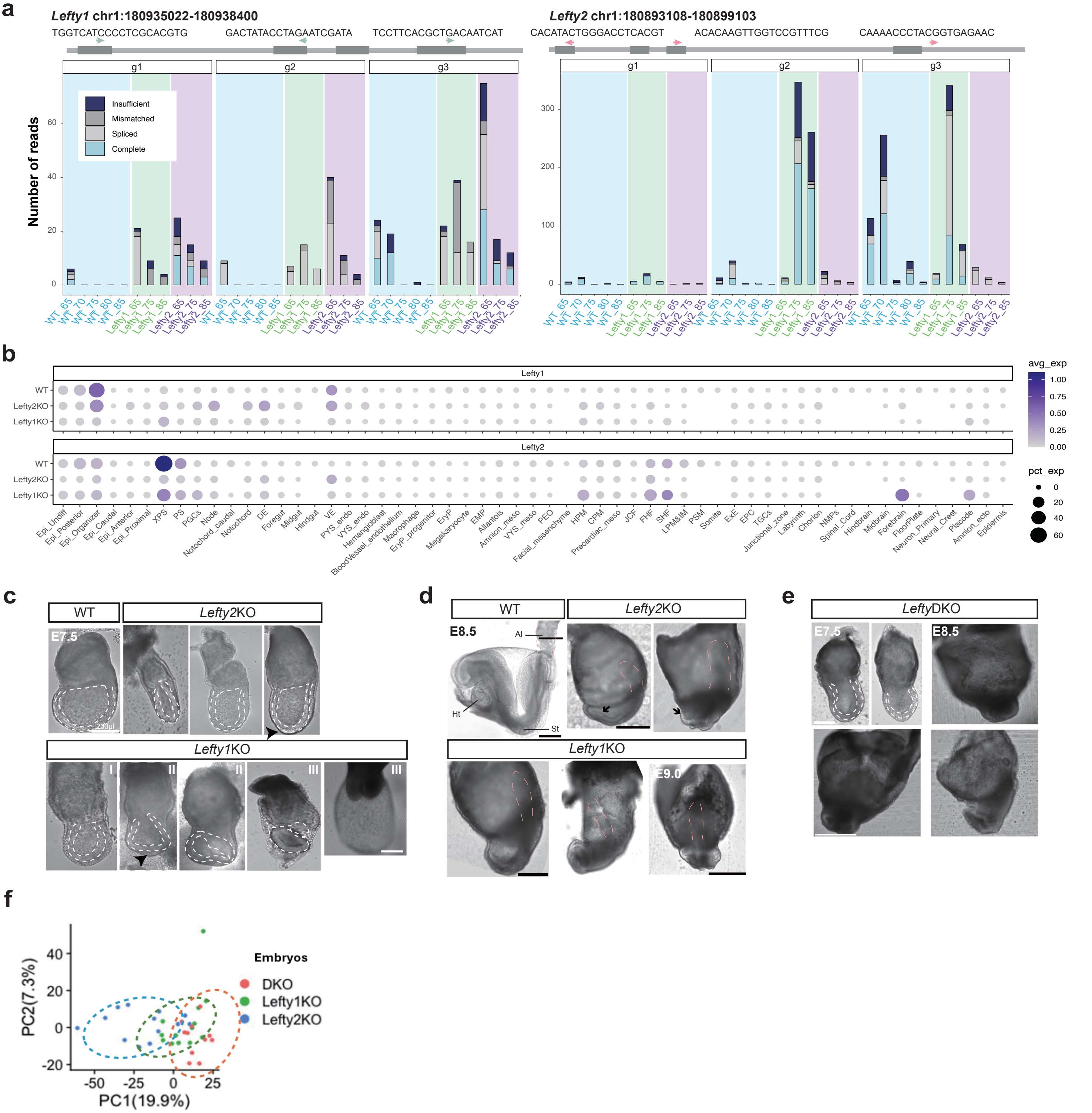

Supplementary Figure 2. Generation of *Lefty1* and *Lefty2* mutant time series data

a. Cut-site analysis confirming high-efficiency disruption of target genes within our *Lefty* mutant data sets. Reads spanning the sgRNA protospacer sequences were used to confirm highly efficient disruption of target loci. Reads are grouped into *mismatched, spliced*, *insufficient,* and *complete* (**Methods**) [22]. Mapping distribution of scRNA-seq reads from WT embryos of stage E6.5 to E8.5 are shown in comparison for each target site of *Lefty1* and *Lefty2*.

b. Dot plots showing the comparable decreased expression of *Lefty1* and *Lefty2* respectively in WT vs *Lefty1*KO and *Lefty2*KO cell states.

c. Bright-field images (epiblast is marked by white dashed lines) of mouse embryos (WT, *Lefty1*KO, and *Lefty2*KO) isolated at E7.5. As previously reported [20], *Lefty1*KO embryos exhibited a variable mid-gastrulation phenotype suggestive of unstable PS initiation, ranging from a mild, overly symmetrical cylinder to a severe loss of clear anterior-posterior (A-P) patterning (I, II, and III, with III accounting for less than 10% of cases). At the same timepoint, *Lefty2*KO embryos were more similar to each other and had comparatively minor phenotypes [15], including a slightly elongated and thicker PS. Scale bar, 50μm.

d. Bright field images of E8.5 WT and *Lefty*KO embryos that converge to a similar macroscopic phenotype, including an expanded extra-embryonic portion and minimized embryonic portion (Allantois is marked by pink dashed lines). The enlarged allantois fails to connect to the placenta, which may contribute to the lethality of *Lefty* mutants past E10.5 [15]. Scale bar, 200 μm. Ht, heart; St, somite; Al, allantois. *Lefty*2KO phenotypes can be distinguished by a headfold-like structure present in the anterior epiblast portion (arrowhead).

e. Generation of *Lefty1/2* double knockouts (DKOs) show phenotypes more consistent with *Lefty1* mutants, confirming the general order of *Lefty* expression during gastrulation.

f. Principal component analysis (PCA) of pseudobulked scRNA-seq data for E7.5 *Lefty1*KO, *Lefty2*KO, and *Lefty* DKO embryos, shown at the per replicate level. DKO embryos are transcriptionally similar to *Lefty1*KO embryos.

Wang et al., Supplementary Fig 3

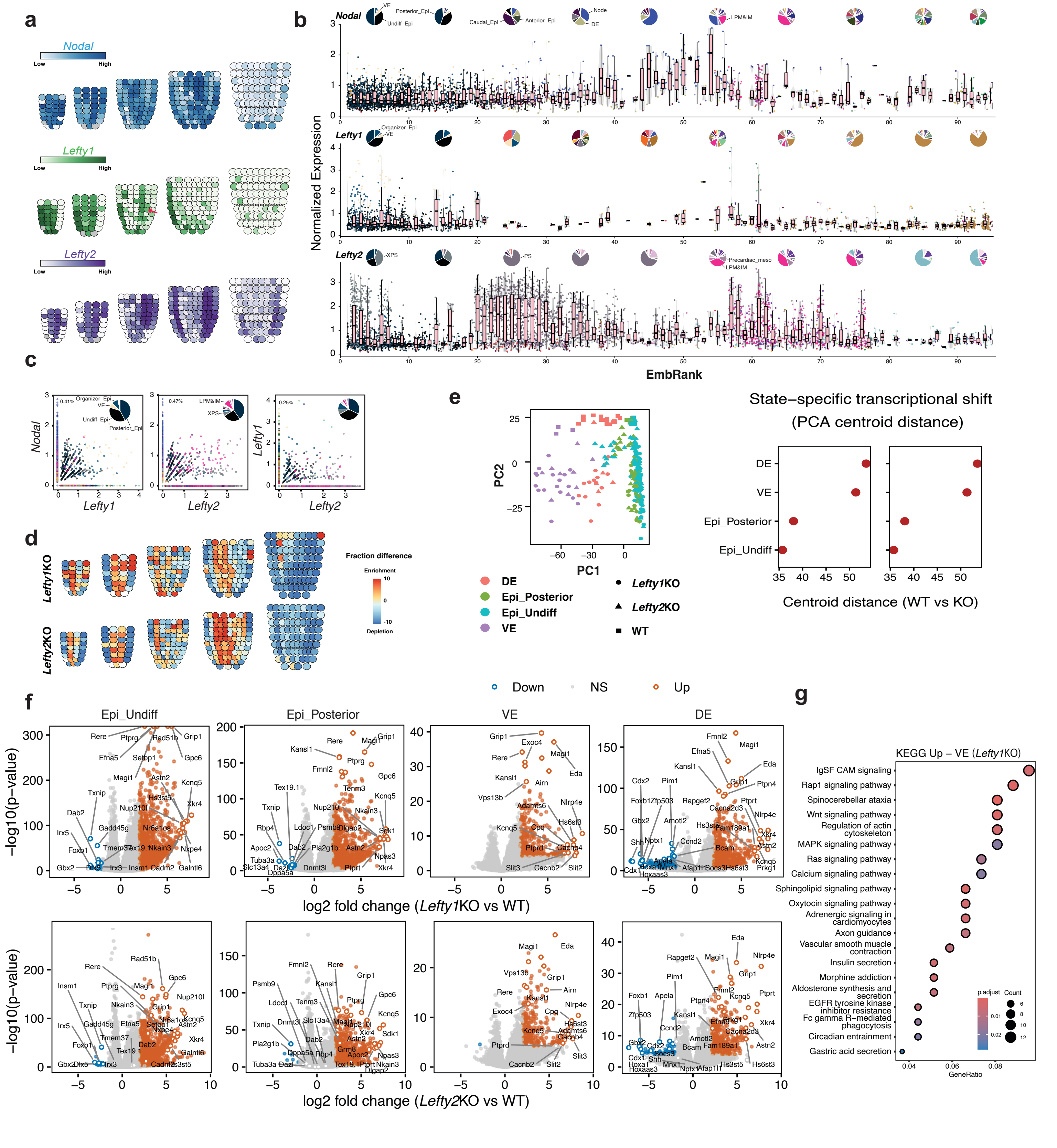

Supplementary Figure 3. *Lefty* mutants display disrupted patterning dynamics consistent with their spatiotemporal expression

a. Corn plots showing expression levels (log10 TPM) of *Nodal*, *Lefty1*, and *Lefty2* in spatially resolved GEO-seq data from E6.5 to E7.5. These data confirm the relative spatiotemporal dynamics of *Lefty* and *Nodal* expression with respect to NODAL signal transduction shown in **Fig. 2d.** This includes a sequential attenuation of NODAL signaling mediated by Lefty1 followed by Lefty2. Our analysis also independently validates *Lefty1* expression in the epiblast organizer region (designated by red arrow).

b. Boxplots showing temporally normalized expression patterns of *Nodal, Lefty1*, and *Lefty2* across cell states (**Methods**). Pie charts above each panel indicate the state proportions of cells expressing each gene across each of ten embryo ranks from E6.5 to E9.0. *Nodal* is initially expressed at low levels in the epiblast and visceral endoderm, then becomes enriched in posterior and caudal epiblast, followed by high expression in the definitive endoderm and node lineages. Later in gastrulation, *Nodal* is subsequently detected at low levels in lateral plate and intermediate mesoderm. *Lefty1* is predominantly expressed in the epiblast and visceral endoderm, with relatively higher expression in the visceral endoderm and Epi_Organizer states. *Lefty2* is initially expressed in the extraembryonic primitive streak, then in the primitive streak, and later in the lateral plate and intermediate mesoderm.

c. Scatter plots of cells showing co-expression of *Nodal* and each *Lefty* gene highlights the degree to which *Lefty1* and *Nodal* are co-expressed in epiblast lineages, while *Lefty2* and *Nodal* are more frequently co-expressed in primitive streak, lateral plate and intermediate mesoderm.

d. Regionalised over/under representation of in cell-state proportions between *Lefty* mutant and WT datasets for the states shown in **Fig. 2a**. Enrichment within the epiblast region reflects epiblast accumulation in *Lefty*KO embryos, consistent with retention of Epi_Undiff states, whereas reduced contributions in visceral endoderm and definitive endoderm regions suggest greater transcriptomic shifts within these lineages. Color intensity reflects the difference in representation between each individual KO and our control reference as projected onto GEO-seq data.

e. PCA analysis of per-replicate pseudo-bulked DE, Epi_Posterior, Epi_Undiff, and VE states, with points colored by cell state. The distances between WT and *Lefty*KO state centroids are shown on the right, revealing greater transcriptomic differences between DE and VE compared to Epi_Posterior and Epi_Undiff in *Lefty* KOs.

f. Differential gene expression analysis (**Methods**) comparing Epi_Undiff, Epi_Posterior, VE, and DE states between WT and *Lefty*KO embryos. The analysis reveals a largely shared set of genes upregulated across these states, with the top increased and decreased genes highlighted. Notably, more than one-third of NODAL target genes are among the significantly upregulated genes, including *Astn2, Fmnl2, Slit3, Xkr4, Cacnb2, Hs6st2, and Ptprd*. This result indicates that, in the absence of Lefty modulators, NODAL-responsive states exhibit a broadly similar response characterized by global upregulation of NODAL target genes.

g. GO analysis of upregulated genes in VE between *Lefty1*KO and WT includes categories related to cell migration, polarity, and morphology, as indicated by enrichment of pathways related to IgSF CAM and Rap1 signaling, actin cytoskeleton organization, and Ras signaling.

Wang et al., Supplementary Fig 4

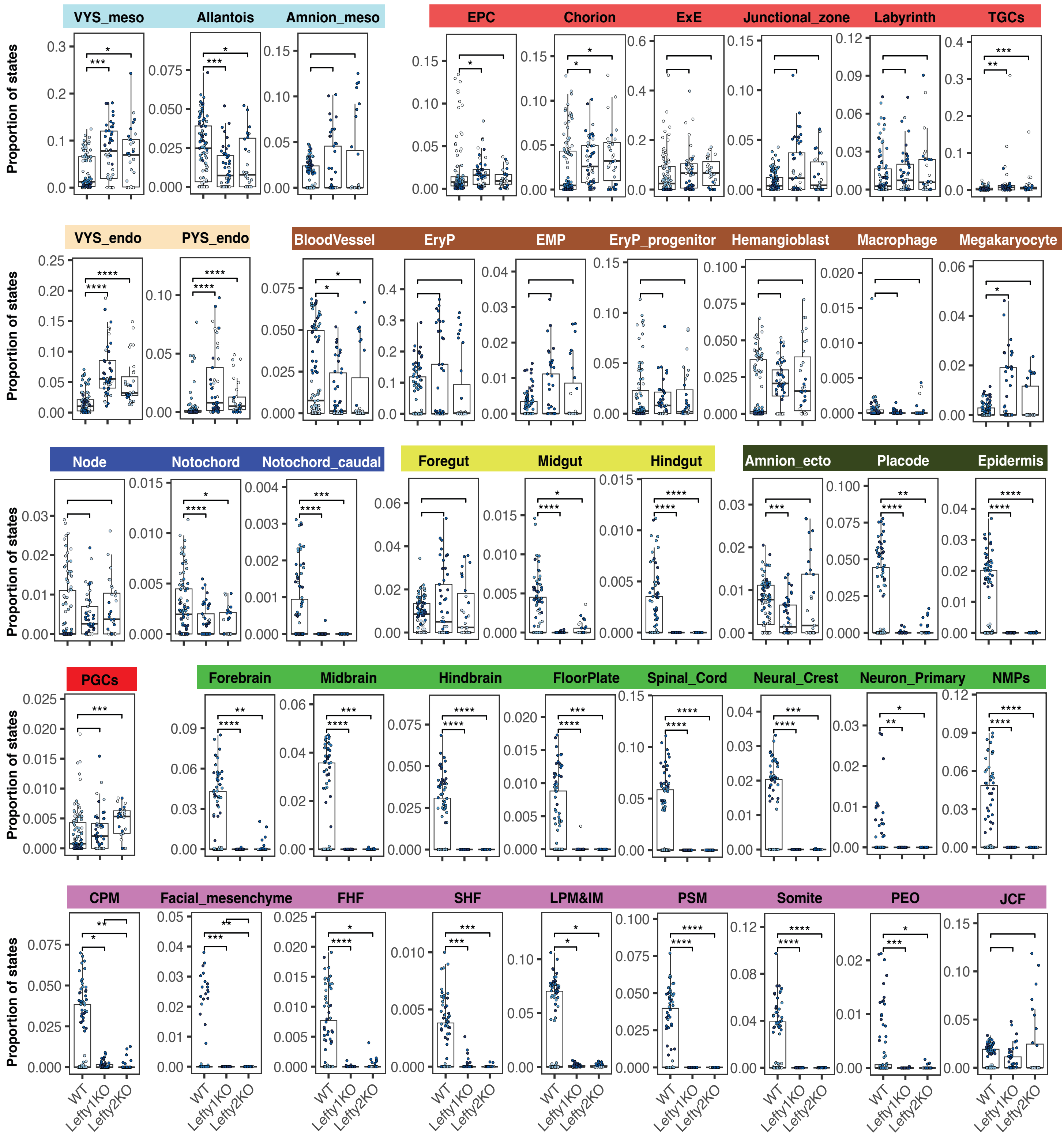

Supplementary Figure 4. Differential cell-state proportion analysis between *Lefty*KO and WT embryos

Per-embryo proportions of each available cell state within our reference, with each dot representing an individual embryo, colored by sampling stage as in **Fig. 2b**. Statistical significance was assessed using the Wilcoxon rank-sum test, with * $P<0.05$, ** $P < 0.01$, *** $P < 0.001$ **** $P<0.0001$.

Wang et al., Supplementary Fig 5

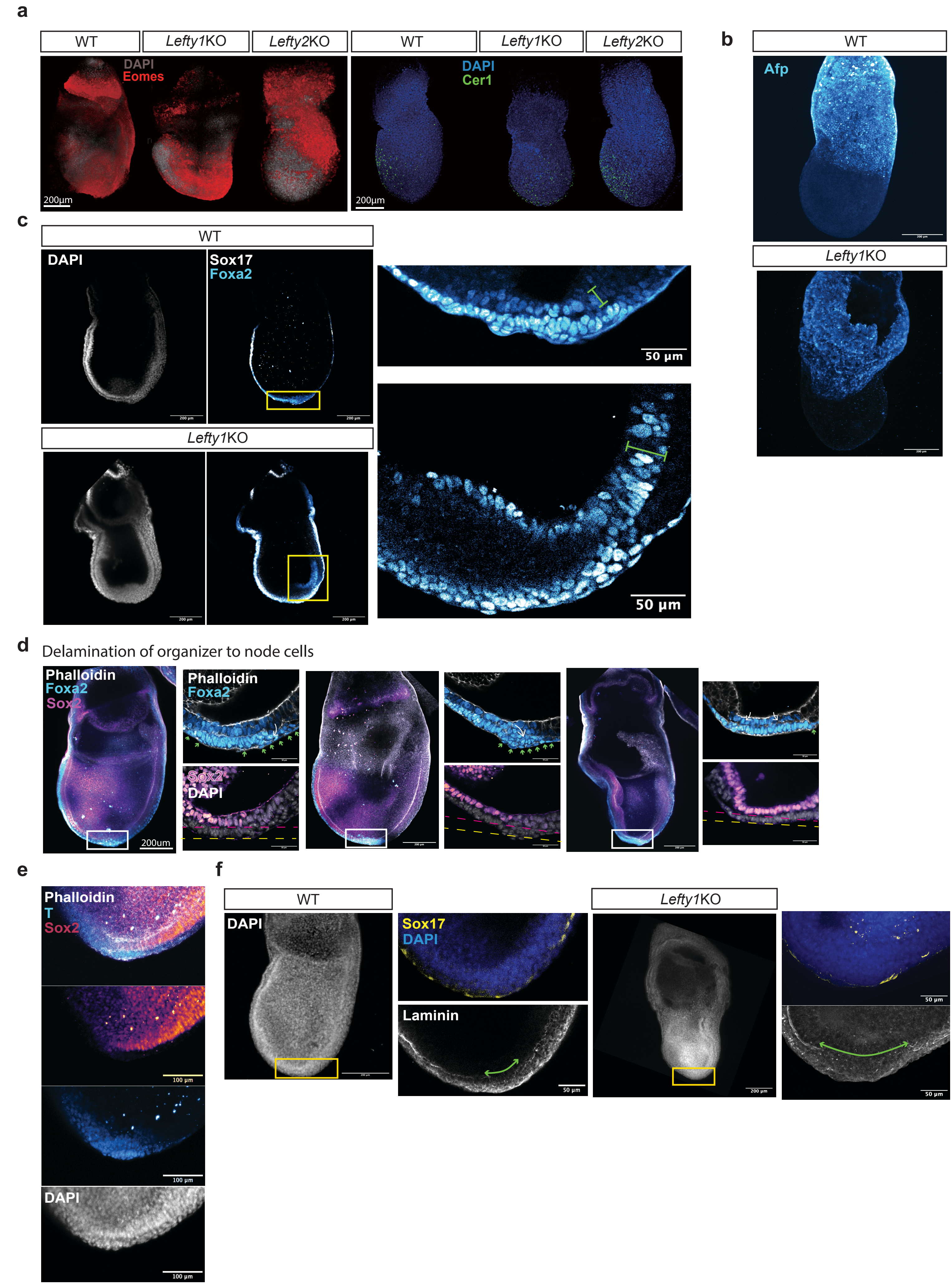

Supplementary Figure 5. Validation of morphological defects as predicted by scRNA-seq analysis

a. Representative 3D reconstructions of WT control, *Lefty1*KO, and *Lefty2*KO E7.5 embryos stained with DAPI (white, top; blue, bottom) and either anti-Eomes (red) or anti-Cer1 (green). *Lefty1*KO and *Lefty2*KO show clear mesodermal cells migrating out of the PS. The *Lefty1*KO embryo shows expanded Cer1 staining in the DVE, indicating an issue with A-P patterning. Scale bar, 200 μm.

b. Whole-mount immunostaining of Afp in WT and *Lefty1*KO embryos at E7.5. Afp, which labels visceral endoderm but not definitive endoderm, shows comparable absence from the embryonic region in both WT and *Lefty1*KO embryos. This indicates that replacement of the embryonic visceral endoderm by definitive endoderm proceeds largely normally in *Lefty1*KO embryos.

c. Magnified views of the regions indicated by yellow rectangles in WT control and *Lefty1*KO embryos at the early head-fold stage, with the epiblast organizer region and endoderm layer distinguished by Foxa2 and Sox17 staining, respectively. The $Foxa2^{+}$/$Sox17^{-}$ organizer domain is markedly expanded in *Lefty1*KO embryos compared with WT controls, as indicated by the green bracket.

d. Immunofluorescence staining of Phalloidin (white), Foxa2 blue), and Sox2 (purple) in WT embryos at E7.25, E7.5, and E7.75, showing that organizer populations within the epiblast contribute to future node lineages. Insets on the right show magnified views of the distal region marked by white rectangles. $Foxa2^{+}$/$Sox2^{+}$ epiblast cells delaminate from the distal epiblast organizer region (white arrows), followed by a transient accumulation of cuboidal $Foxa2^{+}$ cells positioned above the outermost endoderm layer (green arrows). These cells consequently intercalate into the endodermal layer and become node populations.

e. WT control corresponding to **Fig. 2g**, showing that $Sox2^{+}$ cells are confined to the epiblast and do not delaminate into the region between the epiblast and endoderm layers.

f. Immunostaining of Sox17 and Laminin in WT and *Lefty1*KO embryos. $Sox17^{+}$ endodermal cells show comparable staining patterns between WT and *Lefty1*KO embryos and remain confined to the endodermal layer. The lack of Sox17 signal within the distally accumulated region of *Lefty1*KO embryos support the scRNAseq-based prediction that these cells resemble immature node progenitors rather than definitive endoderm progenitors. Laminin staining highlights disruption of the basement membrane between the epiblast and endoderm layers, which is markedly broader in *Lefty1*KO embryos, as indicated by the green bracket. This phenotype is consistent with the aberrant "leakage" of $Sox2^{+}$ cells described in **Fig. 2g**.

Wang et al., Supplementary Fig 6

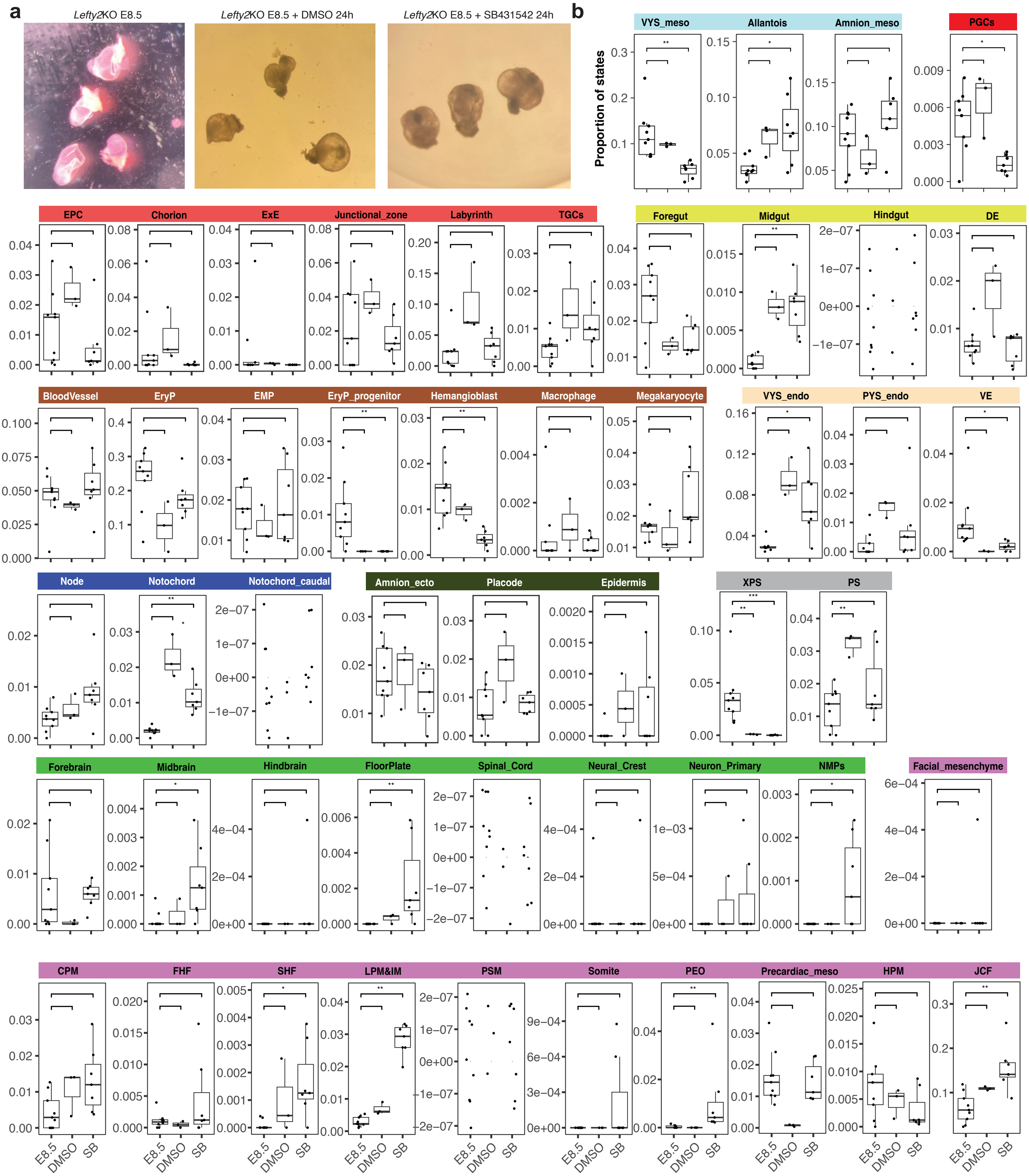

Supplementary Figure 6. Comparison of cell-state proportions between E8.5 *Lefty2*KO embryos and 24-hour roller-cultured *Lefty2*KO embryos

a. Relative morphology of *Lefty2*KO embryos recovered at E8.5 and subjected to 24 hours of either DMSO control or SB-431542-treated roller culture.

b. As in **Fig. 3d**, per-embryo proportions of each cell state within our reference, with each dot representing an individual embryo, colored by sampling stage. Statistical significance was assessed using the Wilcoxon rank-sum test, with * P<0.05, **P < 0.01, ***P < 0.001 ****P<0.0001.

Wang et al., Supplementary Fig 7

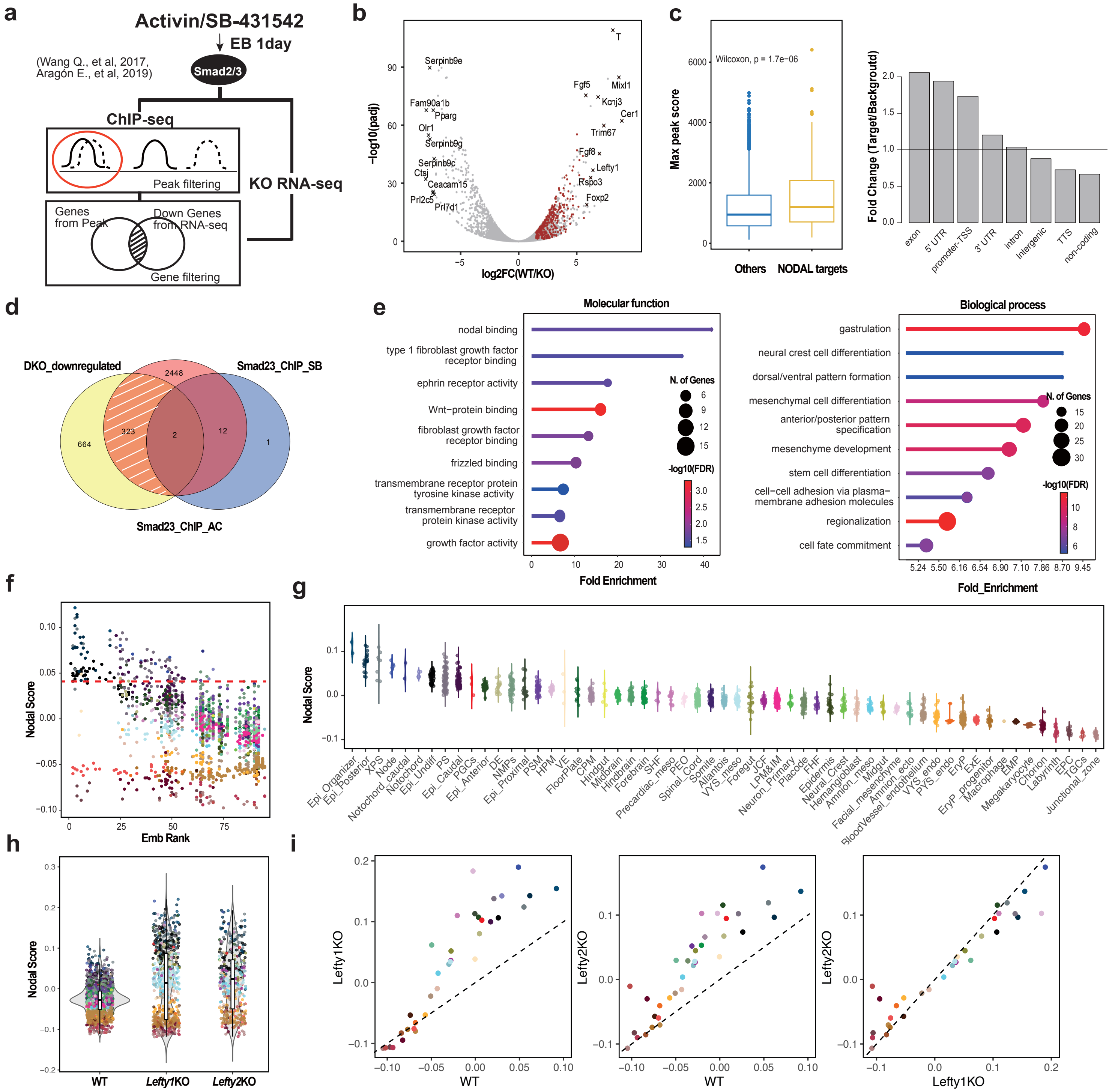

Supplementary Figure 7. Inferring NODAL signal transduction by dynamic gene expression of SMAD2/3 targets

a. Scheme of core NODAL signaling pathway components and experimental scheme used to identify SMAD2/3 targets during EB differentiation, including conditions that use ACTIVIN and SB-431542 as modulators. SMAD2/3 ChIP-seq was done in Day 4 EB after 1-day treatment of ACTIVIN or SB-431542 (GSE70486, GSE125116). The *Smad2/3*DKO RNA-seq was done in day4 EB (GSE125116) [50, 51].

b. Volcano plot depicting differentially expressed genes between WT samples and *Smad2/3*DKO samples. Down-regulated genes (padj<0.01, log2FC>1.5) are highlighted in red, with the top 10 gene candidates labeled with text.

c. *Left*: A boxplot of the maximum peak score for SMAD2/3 ChIP-seq peaks, with those associated with transcriptional induction showing a significantly higher maximum peak score (Wilcoxon test, p=1.7e-6). *Right*: Histogram showing the relative genomic enrichment of transcriptionally associated SMAD2/3 ChIP-seq peaks in different genomic compartments compared to background.

d. Venn diagram showing the intersection between SMAD2/3 ChIP-seq called peaks and significantly down-regulated genes (padj<0.05, log2FC>1.5) in *Smad2/3*DKO RNAseq samples. The 323 peaks that are specific to the SMAD2/3 ChIP and down-regulated in *Smad2/3*DKO vs WT EBs were selected to generate a single cell resolution "NODAL score" for NODAL activity (**Methods**).

e. Gene Ontology (GO) analysis of inferred NODAL target genes, conducted using AmiGO 2 [127]. The results highlight the top significant Molecular Functions and Biological Processes, many of which are associated with NODAL signal transduction or gastrulation.

f. Scatter plots of NODAL target module scores across WT embryonic ranks. Initially, NODAL-responsiveness is bimodal, with early epiblast and extraembryonic endoderm states showing high activity and extraembryonic ectoderm states showing low activity. Over developmental time, the embryo proceeds to lower NODAL activity values. MCs are colored by their dominant single-cell state. The upper plateau of NODAL scores at later stages was used as the threshold to define NODAL-responsive MCs shown in **Fig. 5b**.

g. Dot plot showing NODAL target scores for each recovered metacell (MC) state, ordered from highest to lowest activity in our reference atlas. High NODAL target expression is observed in early epiblast, primitive streak, and node lineages.

h. Violin plot of NODAL target scores calculated from merged WT and *Lefty*KO metacells (MCs), with each MC colored by its dominant single-cell state. The plot demonstrates overall upregulation of NODAL target genes in *Lefty*KO datasets compared to WT.

i. Diagonal plot comparing state-level upregulation of NODAL scores. Except for extraembryonic ectoderm and blood & vessel lineages, both *Lefty1*KO and *Lefty2*KO embryos show elevated NODAL target expression across most states, with NODAL-responsive states such as epiblast, primitive streak, and node exhibiting the greatest increases. Notably, *Lefty1*KO states display a

subtly higher NODAL score than *Lefty2*KO states, consistent with an upstream role for Lefty1 in regulating NODAL signaling activity at the onset of gastrulation.

Wang et al., Supplementary Fig 8

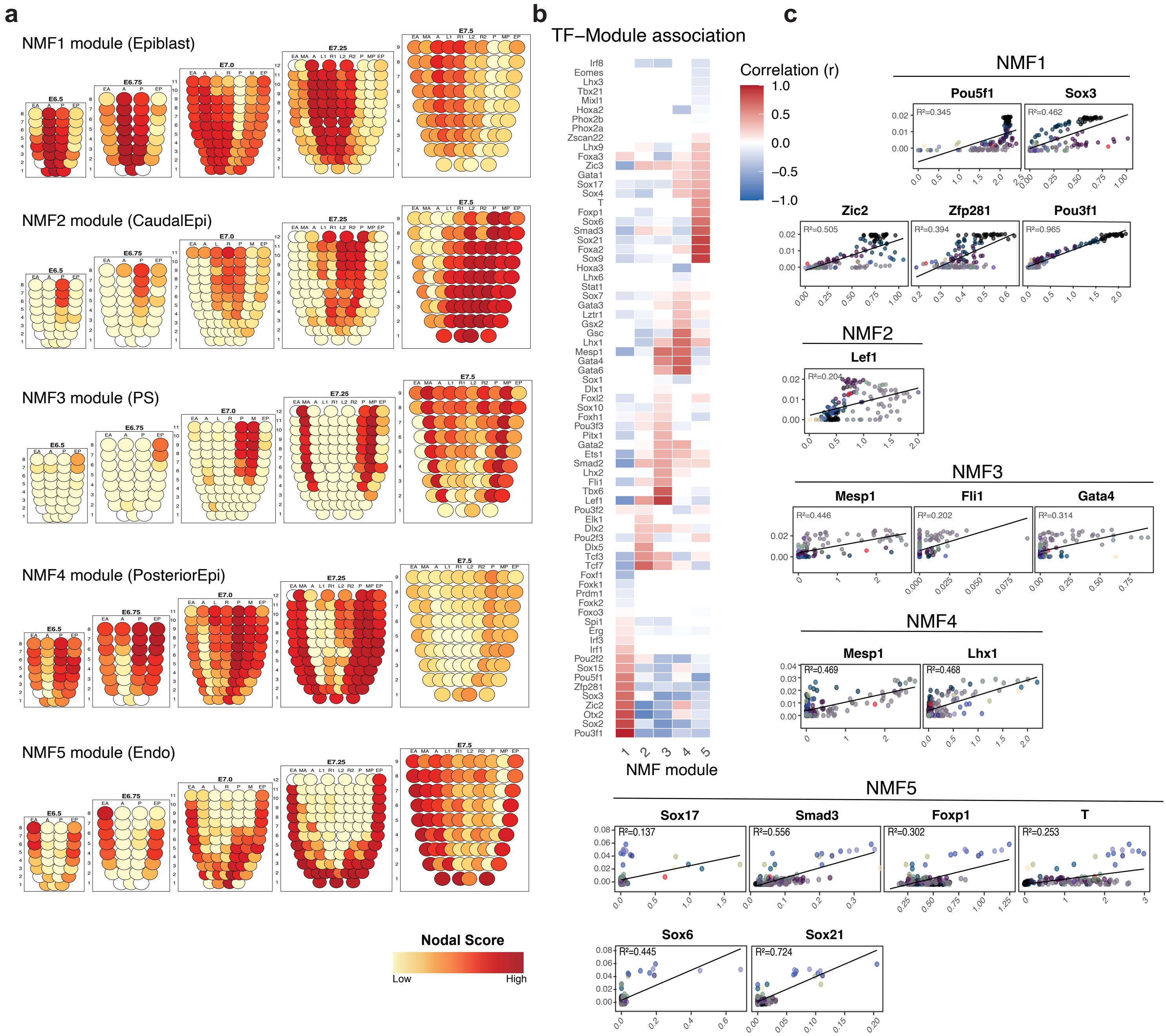

Supplementary Figure 8. NODAL activity modulation and target motif enrichment analysis

a. Corn plots showing aggregated expression levels (log10 TPM) of individual NODAL submodules in spatially resolved GEO-seq data from E6.5 to E7.5. Submodule gene composition and naming are shown in **Fig. 5g**. These data confirm the relative spatiotemporal dynamics of NODAL signal transduction during early gastrulation, including cell state specific differences in signal transduction.

b. Pearson correlation between transcription factor (TF) activity as inferred from motif enrichment analysis of ChIP-seq data (**Method**), and NMF module embeddings. This analysis highlights candidate TFs that may interact with SMAD2/3 signal transduction within specific modules.

c. Extended from **Fig. 5d**, correlation between the inferred activity of individual transcription factors and NODAL submodules for additional TFs, including those associated with Pluripotency (NMF1; Pou5f1, Zic2, and Zfp281), Caudal Epiblast and Primitive Streak differentiation (NMF2 and NMF3; Lef1, Mesp1, Fli1, Gata4), Posteriorization (NMF4; Mesp1, Lhx1), and Definitive Mesoderm differentiation (NMF5; Sox17, Smad3, Foxp1, and T).

qRT-PCR primer list

Sequence	Tm	Source	Length
En1_RT_F:ACACAACCCTGCGATCCTACT<br>En1_RT_R:GGACGGTCCGAATAGCGTG	63	PrimerBank:7106305a2<br>PMID:16678814	120
Foxg1_RT_F:ACAAGAAGAACGGCAAGTACG<br>Foxg1_RT_R:CATAGATGCCATTGAGCGTCA	61	PrimerBank: 6679843a2<br>PMID:16678814	108
Gapdh_RT_F:TTCAACAGCAACTCCCACTCTTC<br>Gapdh_RT_R:CCCTGTTGCTGTAGCCGTATTC	58	PMID:16678814	113
Sox1_RT_F:CAAGATGGCCCAGGAAAA<br>Sox1_RT_R:TCGGACATGACCTTCCACTC	56	PMID:16678814	81
Oct3_RT_F:AGAGGATCACCTTGGGGTACA<br>Oct3_RT_R:CGAAGCGACAGATGGTGGTC	62	PrimerBank:356995852c1	96
Foxd4_RT_F:GAGAGCCATAAGTGCCTGGAG<br>Foxd4_RT_R:GGAGGAGTTACTCAGATCGCC	62	PrimerBank:261824060c1	99
T_RT_F_2: GCTTCAAGGAGCTAACTAACGAG<br>T_RT_R_2: CCAGCAAGAAAGAGTACATGGC	61<br>61	PrimerBank: 6678203a1	117
Six3_RT_F_2:TCCGCGACCTGTACCACAT<br>Six3_RT_R_2:TTGAAGCAATGGGTCTTCTGC	63<br>61	PrimerBank:<br>258613883c3	208
Nkx2-1_RT_F_2: ATGAAGCGCCAGGCTAAGG<br>Nkx2-1_RT_R_2:GGTTTGCCGTCTTTGACTAGG	62<br>61	PrimerBank:<br>226246555c1	164
Gsc_RT_F:CCCCGGTTCTGTACTGGTG<br>Gsc_RT_R:TCTGGGTACTTCGTCTCCTGG	62<br>62	PrimerBank:6754075c1	192

Minming Wang - Personal notes on State annotation
(V1.0 2023-3-1;V2.0 2023-5-25; V3.0 2023-6-18, V4.0 2023-9-04, reviewed 2026-5-27)

Based on "010423_PreliminaryState_Descriptions", I have further annotated some ambiguous early states. Some marker genes may be repeated several times in different lineages, and the expression descriptions of those genes may vary slightly, with a focus on different germ layers. The states listed in this note are not exactly same as in the final paper.

Overview of the annotation

Stage: E6.5-E9.0

My annotations of states are organized by lineage as the primary unit, and I will provide detailed annotations for each lineage. Lineages are organized based on their developmental trajectories, with a focus on continuity, which means that the name of a lineage may not necessarily reflect its full composition.

Lineage:

- Epiblast(Epiblast+PS+PGC)
- Endo(PEpi+Endo+XEndo+Notochord)
- XMeso(PPS+XMeso+Blood)
- NeuralEcto(Ecto+NMP)
- NonNeuralEcto
- Meso
- XEcto

Based on the common definition of germlayer and the separation of clusters, each state within the dataset is assigned to a specific germlayer.

Germlayer:

- Epiblast
- Endo
- XEndo
- XMeso
- Meso
- NeuralEcto
- NonNeuralEcto
- XEcto
- Blood
- PGC

Lineage

① Epiblast

Description:

In mouse embryos, the epiblast is the source of all fetal tissue, both somatic and germline, as well as of the extraembryonic amnion, yolk sac mesoderm and allantois. At E5.5, the epiblast exhibits symmetry and pluripotency. At E6.5, gastrulation is initiated with the formation of **Primitive streak (PS)** on the posterior side of Epiblast **(Posterior_Epi)**, where definitive endoderm and axial mesoderm cell fate are derived. The cells originally in the posterior half of the early streak are replaced in the PS, by continuously expanding lateral epiblast, moving into extraembryonic mesoderm **(Posterior_PS)** and mesoderm (**PS**). As the PS elongates, the **caudal lateral epiblast (CLE)** arises as a distinct population of adjacent epiblast cells. The CLE, housing the **neuromesodermal progenitors (NMPs)**, contribute to posterior neural and paraxial mesodermal lineages, plays a crucial role in the elongation of the embryo, knwon as second gastrulation. It is believed that the restriction of the ectoderm germ layer has not yet occurred until E7.0. The cells that remain on the anterior side of epiblast (**Anterior_Epi**) and do not pass through the PS form Neuroectoderm. In contrast, the cells in the adjacent region anterior/proximal to the Anterior_Epi (**Proximal_Epi**), which are in close proximity to the ExE, contribute to surface ectoderm. The emergence of the murine germline becomes apparent at

around E7.0, with **primodial germ cells (PGCs)** representing the earliest recognizable precursors of gametes. It should be noted that the population of Amnion ectoderm may be too small to be adequately sampled in our dataset.

The states and their future cell fates described above mainly based on early clonal analysis of the epiblast (PMID: 1516475). It is important to note that the position information in the state names primarily describes their current position when specified, rather than their previous position in the embryo, as these cells are not stationary.

**E6.5**	Epiblast_early, Posterior_Epi_early, Posterior_PS
**E7.0**	Epiblast_mid, Posterior_Epi_mid, Caudal_Lateral_Epi, Anterior_Epi, Proximal_Epi, Posterior_PS, PS_mid, PS_late, PGC
**E7.5**	Caudal_Lateral_Epi, Anterior_Epi, Proximal_Epi, PS_late, PGC
**E8.0**	PGC

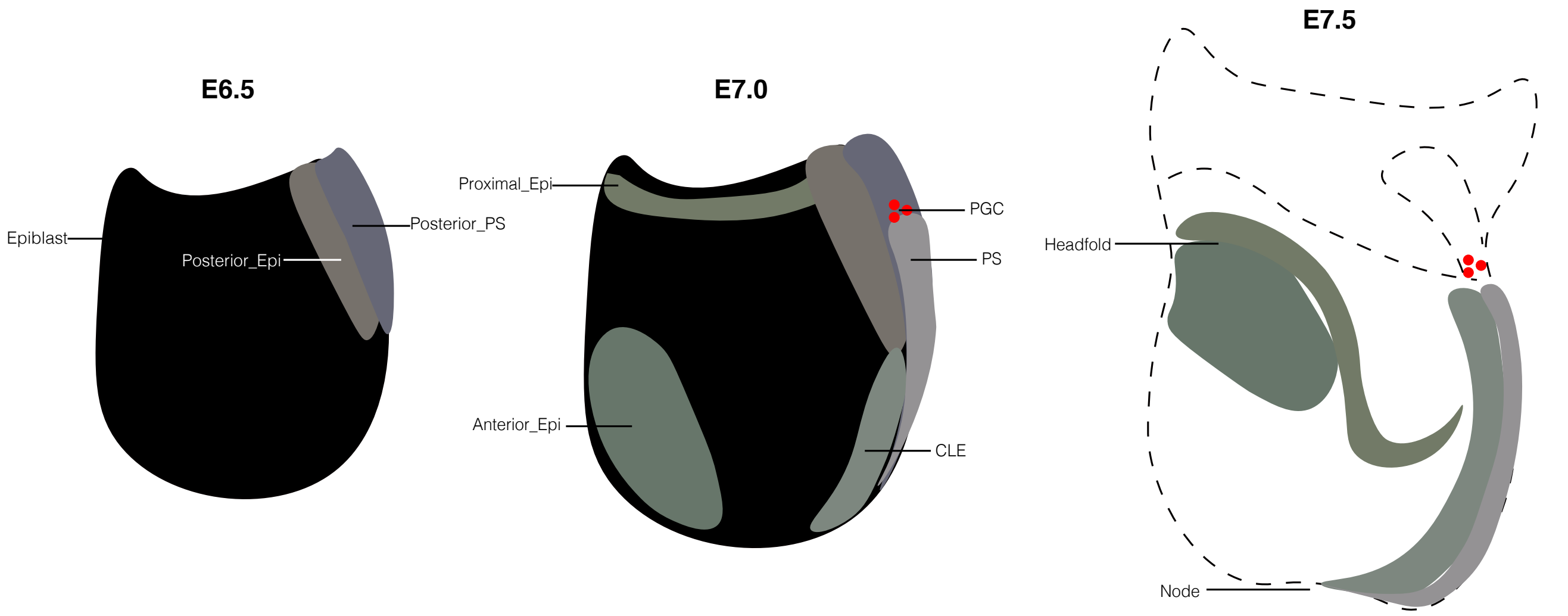

Expression pattern:

————————**Pluripotency marker**————————————————————————-

Sox2: [PMID: 12514105, insitu] E6.5 Epiblast, ExE. E7.5 decrease in poster epi. E8.5 persist in chorion, headfolds and neural tube. E9.5 throughout the nervous system, sensory placodes, branchial arches and gut.

Pou5f1:[PMID: 8631266, lacZ]: E6.5-E7.0 Epiblast. E8.0 decreased in Anterior Epi. Not detected in E9.0, except for PGC.

Tcf7l1: [PMID: 23487311,IF] Pre-streak stage throughout epiblast. Reduce near the PS, and progressively diminished in the posterior epiblast.

————————**Epiblast enriched**—————————————————————————

Sox2, Sox3: [PMID: 10446282, insitu]E6.5 whole Epiblast and a band of ExE. E7.0-E8.0 anterior ectoderm and the extraembryonic expression becomes confined to the chorion ectoderm while Sox3 is rapidly downregulated in chorion. Sox2 is expressed in anterior ectoderm which will give rise to neuroectoderm and anterior surface ectoderm while Sox3 spreads into posterior epiblast. At headfold stage, Sox2 and Sox3 show complementary anteriorposterior gradients of expression, with Sox2 expressed mostly in the anterior ectoderm and Sox3 biased towards the posterior.

Dnmt3a,Dnmt3b: [PMID: 10555141, lacZ] E7.5 Dnmt3a is moderately expressed in the embryonic ectoderm and weakly in mesodermal cells. Dnmt3b is highly expressed in the embryonic ectoderm, neural ectoderm and chorionic ectoderm. E8.5 Dnmt3b is expressed in the forebrain and eyes [dnmt3b-antibody-a371] E4.5 ICM. E5.5 Epiblast. E7.5 Mostly Epiblast.
Pou5f1:[PMID: 1972777, insitu] [PMID: 26534985, insitu] E5.5 whole Epiblast. [PMID: 23487311, insitu] E7.5 Most Epiblast, posterior epiblast show reduced expression. E8.0 Decreased in head fold. E8.5 no expression. [PMID: 29915126, IF] E6.0 Epiblast and ExE. E7.0 Epiblast and PS/nascent mesoderm.
Epcam, Utf1: [PMID: 30787436, scRNA] Epiblast.
Otx2: [PMID: 23487311, insitu] E7.5 Anterior Epiblast. [PMID: 29915126, IF] E7.0 high in anterior Epi and the under VE.
Cdh1:[PMID: 29915126, IF] high in VE and Epiblast, low in meso/PS.
Pouf3f1: E5.5 throughout the epiblast. E6.5-E7.0, limit to the he anterior part of the epiblast. E7.5-E8.0 anterior neuroectoderm. E9.5 forebrain and midbrain.
Fgf5: [PMID: 1794311, insitu] E5.5-E7.5 epiblast, and gradually decrease in proximal, not detect in migrated mesoderm cells (like XMeso). E7.75 no longer detected.

Negative marker: **Cdh2**[PMID: 31371508, IF] **, Mixl1,T, Foxa2, Snai1, Eomes** [PMID: 29915126, IF] E6.75 PS. E7.0 PS/meso elongate to Node. Eomes E6.75 is expressed in PS/meso.

————————Posterior_Epi enriched ————————————————————————-
Fst:[PMID: 10853830, insitu] E7.5 posterior Epiblast. mesoderm wing. E8.5 hindbrain, PYS, paraxial meso. E9.5 somites, mesencephalon, diencephalon
Nanog: [PMID: 23487311, insitu] E6.5-E7.5 posterior epiblast, not in PS. [PMID: 23487311, insitu] E7.5 posterior epiblast. E8.5 no expression.
Tdgf1(Cripto): [PMID: 14623818, insitu] E6.5 Cripto is expressed in posterior Epi and PS nearby.
Nodal: [PMID: 26534985, insitu]
Eomes: [PMID: 10716450, lacZ] E6.5-E7.5 ExE, PS and posterior Epi.
Mixl1: [PMID: 24596343, insitu; 24632399, IF] E5.5 Mixl1 is first expressed in cells of the visceral endoderm. At the time of gastrulation, Mixl1 expression is detected in posterior Epi, PS, and posterior VE overlying the primitive streak. Later it is restricted to the ACD and tailbuds mesoderm by headfolds.
Gsc: [PMID: 1352187,9533949, insitu] Its mRNA accumulates as a patch on the side of the epiblast at the site where the PS is first formed. Gsc expressing cells are then found at the anterior end of developing PS, and finally in the anteriormost mesoderm, a region that give rise to the head process(floorplate).
Foxa2(Hnf3β): [PMID: 8306889, insitu] first express in the anterior portion of the PS (and the underlying epiblast). Its expression persists in axial structures (definitive endoderm and notochord) and in the ventral region of the developing neural tube (floorplate).
Fgf8: [PMID: 7768185, insitu] E6.0-6.5 posterior Epi; E7.5 extends into ACD. E7.75-E8.0 high expression in PS, with a sharp anterior boundary at the node, wall of anterior intraembryonic coelom (PM). E8.5-E9.5 lateral region of the myotome at the anterior and posterior margins of the somites. Forebrain and near the midbrain-hindbrain junction.

————————-**Caudal_lateral_Epi**————————————————-
Nkx1-2: [**https://doi.org/10.1101/045872**, PMID: 7669696, insitu] E6.0 no expression. E7-E7.5 PS near node. ~E8.5 posterior forming neuroepithelium, anteriorly ending at the level of the last formed somite. ~E10.5 during secondary neurulation, it is expressed in the dorsal part of the tail bud, extending into the neural tube.
Sox2,Sox3: [PMID: 24508530, insitu] express low in CLE.

——————-**Anterior_Epi**———————————————
Sox1: [PMID: 29915126,31601548, IF] E7.5 anterior Epi.
Otx2: [PMID: 29915126, IF] E7.75, headfold.
Hesx1, Six3, Otx2: [PMID: 26534985, insitu] E7.5 anterior Epiblast.
Sfrp1,Sfrp2:[PMID: 28292423, insitu] E7.5 Anterior Epiblast, headfold.
Hesx1: [PMID: 8565852, insitu] E7.0-E7.5 Anterior Epi and VE/endoderm, PCP.
Irx3, Six3, Sox2: [PMID: 22186731, insitu] E7-E8.0 anterior epiblast.

————-**Proximal_Epi**———————————————
Wnt6, Lrp2: [PMID: 36252226, scRNA]
Krt18,Krt8: [PMID: 24131634, insitu] E7.0-E7.5 proximal epiblast.
Tfap2c(AP-2),Dlx5: [PMID: 22186731,26534985,, insitu] E7-8, proximal Epiblast.
Hand1: [PMID: 9500550, lacZ]E7.5 EPC, amnion and chorion, allantois, posterior PS.

Active BMP targets: Id1,Id2, Msx1, Msx2 [PMID: 27435625, insitu]

——————-**PS enriched**——————————————————————————-
Hoxb1: [PMID: 26534985, insitu] E7.5 high in PS, low in mesoderm wing.
Snai1: [PMID: 31371508, IF] PS, not in epiblast.
Cdh2: [PMID: 31601548, IF] Meso and PS.
Wnt5a, Wnt5b: [PMID: 28292423, insitu] E7.5 right to node.
Gbx2: [PMID: 12799077] E7.5 PS at anterior border at prospective mid/hindbrain junction. E8.25 PS and throughout the neural tube with rostral border at the mid/hindbrain junction.
Sp5:[PMID: 11231070, insitu] E7.25 PS. The lateral most mesoderm is negative. E8.25 Posterior end of neural plate and the underlying mesoderm. DefEndo as well as notochord become positive. E8.5-10.5, tailbud and mesencephalon and diencephalon.
Mesp1:[PMID: 21148448, insitu] E6.5 early mesodermal cells that ingress into and exit the PS. A weaker expression of Mesp1 is seen as a pair of bands in the presomatic mesoderm. Beyond E8.5 Mesp1 and Mesp2 are expressed in the presomitic mesoderm.

———————-Posterior_PS/XPS————————————————————————
Wnt3: [PMID: 26534985, insitu] posterior PS and Epi.
Lefty2: [PMID: 9348041,10518210, insitu] E6.5-E7.5 PS. E8.0 left LPM
Eomes: [PMID: 10716450, lacZ] E6.5-E7.5 ExE, PS and posterior Epi.
Evx1: [PMID: 1971786,1349539, 7669696, insitu] E7.0-E9, Evx1 emerges at the posterior end of the embryo within the Epiblast and PS, and later in mesoderm and neuroectoderm.
Mesp1:[PMID: 21148448, insitu] E6.5 early mesodermal cells that ingress into and exit the PS. A weaker expression of Mesp1 is seen as a pair of bands in the presomatic mesoderm. Beyond E8.5 Mesp1 and Mesp2 are expressed in the presomitic mesoderm.
Kdr(Flk1):[PMID: 7647376, insitu] E7.0 posterior PS.
Lhx1(Lim1):[PMID: 16728477, insitu] E6.5 Lim1 in AVE and posterior proximal emVE, subsequently, expression is seen in the PS and its derivatives as well as in the mesodermal wings and prechordal plate.

————————PGC——————————————————————————————
Prdm1(Blimp1), Dppa3(Stella), Ifitm3(Fragilis), Alpl: [PMID: 15937476, insitu] E6.25 most proximal layer of the epiblast. ~E7 allantois base, PGC.
Prdm14: [PMID: 18622394, KI]
Tfap2c(Ap2γ): [PMID: 19776388, IF]
Dnd1,Nanog, Kit, Nanos3, Ddx4: [PMID: 19410535, review]

Active BMP targets: [PMID: 31583942, review] Id1,Id2, Msx1, Msx2

Negative marker: **Otx2**

② Endo

Description:

The primitive endoderm gives rise to two extraembryonic cell types: **parietal endoderm (PE)** and **visceral endoderm (VE)**. The parietal endoderm, a single layer of cells that develops in association with the trophectoderm, together later develop into the **parietal yolk sac (PYS)**. The visceral endoderm develops in association with the epiblast and can be further divided into three sublineages based on position and morphology: **Extraembryonic visceral endoderm (ExVE)**, **Embryonic visceral endoderm (EmVE)**, and **Anterior/Distal visceral endoderm (A/DVE)**. The future **visceral yolk sac (VYS)** is thought to form from the VE layer plus the extraembryonic mesoderm layer. It is worth noting that the gene expression pattern of the liver and small intestine, and the placenta may resemble that of the VYS and PYS, respectively. The **definitive endoderm (DefEndo)** and **notochord plate**(and **prechordal plate**) begins to segregate from **anterior primitive streak (APS**, posterior Epi in our annotation) at E6.0-E6.5, gradually replacing EmVE. The DE can be visually identified as an epithelial sheet of approximately 500~1000 cells around the distal cup of an E7.5 mouse embryo. The gut is formed from anterior to posterior, comprising pharyngeal endoderm, foregut, midgut, and hindgut, which gives rise to the major cell types of many internal organs, including the thyroid, thymus, lung, stomach, liver, pancreas, intestine, and bladder.

**E6.5**	Posterior_Epi_early, VYS_endo, PE&PYS_endo, AVE&DVE, DefEndo, EmVE, ExVE
**E7.0**	Posterior_Epi_mid, VYS_endo, PE&PYS_endo, Node&Notochordal_plate, EmVE, AVE&DVE, DefEndo
**E7.5**	VYS_endo, PE&PYS_endo, Node&Notochordal_plate, Hindgut, DefEndo
**E8.0**	VYS_endo, PE&PYS_endo, Node&Notochordal_plate, Hindgut, Midgut, Anterior_foregut, Posterior_foregut, Pharyngeal_endo
**E8.5**	VYS_endo, PE&PYS_endo, Node&Notochordal_plate, Hindgut, Midgut,Anterior_foregut, Anterior_foregut, Posterior_foregut, Pharyngeal_endo

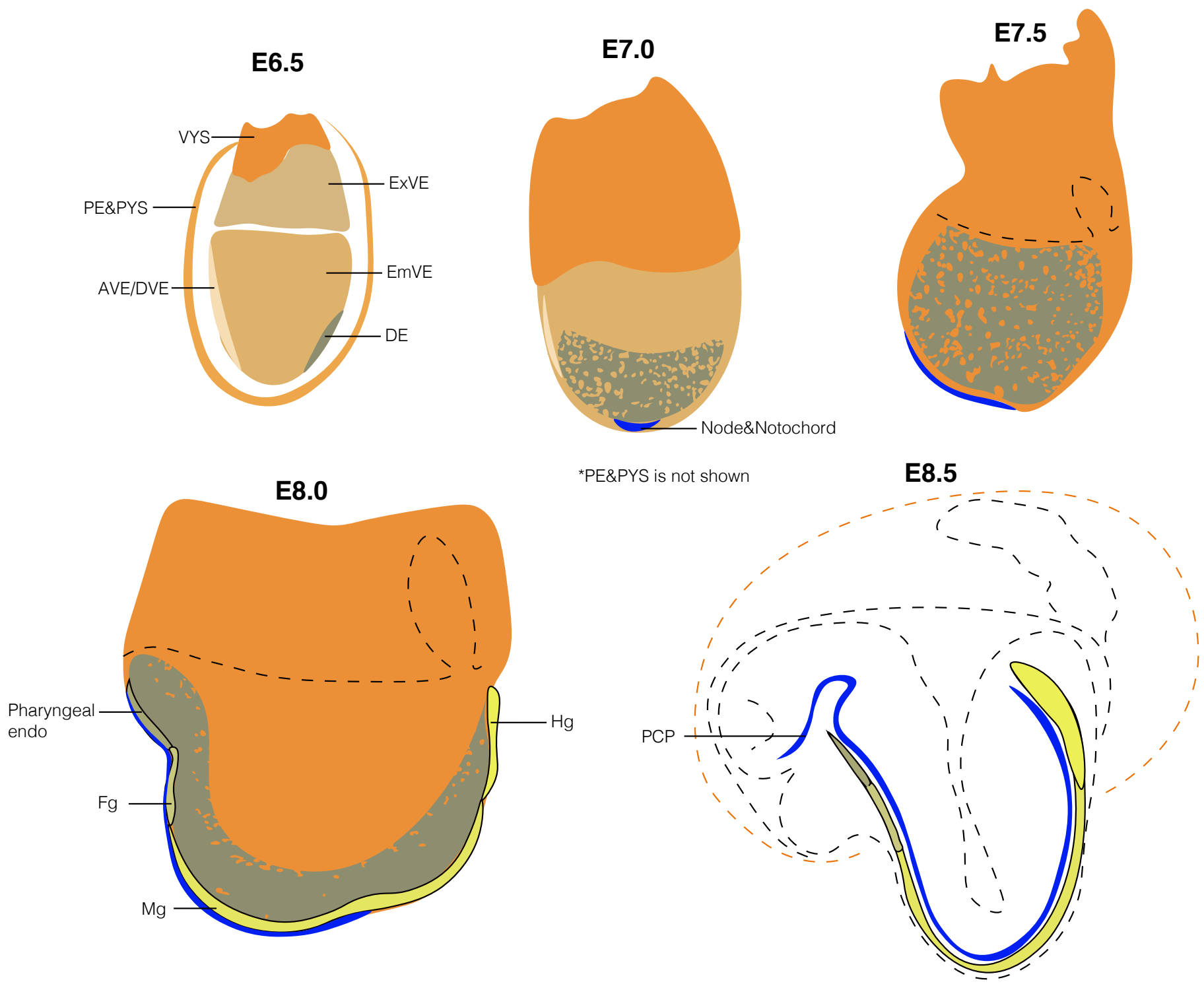

State relationships and assumptions:
- VE possess the ability to transdifferentiate into PE.
- VE participate in the definitive endoderm formation, mainly hindgut.
- After E7.5, VE cells basically drain into VYS_endo.

Important biological processes:
*This session serves to introduce some of the developmental processes of the states present in this lineage.
Hematopoiesis/vasculature: Blood islands in the VYS produce large primitive red cells around E7 and E7.5, with the VYS_endo believed to be a source of the inductive signals necessary for the formation of blood cells and endothelial networks in the yolk sac. By E8.0, the yolk sac vasculature is formed through fusion of these blood islands. This information could be useful if we wish to further classify the VYS_endo.

Morphogenesis of Gut tube: During mouse embryonic development, by E8.0-E9.5, specific domains within the gut endoderm are marked by the local expression of certain transcription factors along the anterior-posterior axis. The anterior gut region, which gives rise to the esophagus and stomach, as well as the lungs, liver, and pancreas, initially expresses Sox2, while the posterior gut expresses Cdx2, creating a clear boundary at the pylorus. Morphogenesis of the primitive gut begins with the formation of two intestinal pockets, one in each region. The foregut pocket starts as a crescent-shaped depression under the neural folds, then deepens and extends rostrally, with the opening moving posteriorly and eventually closing as the foregut develops. Similarly, the hindgut pocket forms 6-8 hours later (equivalent to the time taken for the segmentation of six to eight somites) in the posterior region, with its opening closing anteriorly. As these two gut pockets elongate and close, the lateral walls of the embryonic gut fold ventrally, eventually meeting at the yolk stalk and forming a gut tube by E9.0 (20-25 somites).

Pharyngeal/branchial region: The pharyngeal region is a segmented structure composed of a series of reiterated structures: the **pharyngeal arches** on the exterior surface, the **pharyngeal pouches (Pharyngeal_endo)** on the interior, and a **mesenchymal core** (derived from paraxial mesoderm (**Cranial_meso**) and **neural crest cells**). Mammals have five pairs of pharyngeal arches numbered 1, 2, 3, 4, and 6 (with 5 regressing as soon as it forms) in a rostral to caudal order. In mice, these arches form between day 8 and day 11. Arch 1 has two prominences: the upper (maxillary) prominence forms the maxilla (upper jaw), zygomatic bone, and the squamous part of the temporal bone, while the lower (mandibular) prominence forms the mandible (lower jaw). Arch 2 forms most of the hyoid bone, while arches 3, 4, and 6 form various neck structures, including the cartilage of the larynx. The thyroid forms from the ventral foregut at the level of the second arch, the thymus and parathyroid from the third pouch, and the ultimobranchial bodies from the fourth pouch, collectively known as the pharyngeal organs. The pharyngeal arches also consist of blood vessels, nerves, muscles, and cartilage components. The pharyngeal pouches are transient paired endodermal outpocketings that form on the lateral surfaces of the pharynx and lie in between the exterior pharyngeal arches.

Foregut: The foregut endoderm is categorized into two regions, namely **Anterior_foregut** and **Posterior_foregut**, which eventually develop into lungs, esophagus, trachea, forestomach; antral stomach, liver, and pancreas. Typically, the organ buds are determined around E10. Therefore, in our annotation, we differentiate only the positional aspect of the gut tube. The mesenchymal cells that make up the connective tissue, endothelial cells, cartilage, lymphatics, and the outer surface mesothelial cells of these organs originate from the **Lateral Plate Mesoderm (LPM)**.

Midgut: The midgut region is situated behind the pancreatic domain and is characterized by the presence of Pdx1 expression in the most anterior part, followed by Cdx2. This region encompasses the endoderm that will eventually differentiate into the small intestine and duodenum.

Hindgut: The region furthest towards the posterior end is characterized by the expression of Cdx1 and Cdx2, and it will ultimately give rise to the colon.

- **Anterior/Axial Mesoendoderm (AME):** Around E6.25, the Anterior/Axial Mesendoderm (AME) emerges from the anterior primitive streak and migrates anteriorly to replace AVE. The AME is composed of the **prechordal plate (PCP)** and the anterior **notochord plate (NCP)**. The PCP is positioned beneath the neural plate, which contributes to the midline structures of the foregut endoderm, including the presumptive oral endoderm and ventral cranial mesoderm. While the cells of the NCP invaginate from the ventral surface of the embryo, first in the most anterior region and then progressively in a rostro-caudal direction, remaining connected to the endoderm by their basement membranes until approximately E9.5 (around E8.25, the node disappears). Around E10.5, the basement membranes between the endoderm and notochord separate, and the notochord adheres to the ventral surface of the neural tube. Finally, by E12, the notochord detaches from the neural tube as mesenchymal cells infiltrate between them, enclosing the notochord. In our dataset, the intact PCP is too few to be detected, I only annotate node&notochord plate (may include some PCP cells)**.**

Expression pattern:
————————PE/PYS_endo enriched—————————————————————
Serglycin (Srgn): [PMID: 1136593, insitu] E8.5 Srgn is detected in PE and antimesometrial decidua. E11.5-E14.5 in fetal liver.
Pga5, Scube2: [PMID: 28012457, insitu] E11.5, expressed in PYS endoderm cells. [PMID: 21362171] E8.5 Pga5 is expressed weakly in whole lateral gut epithelium.
Ckap4(P63): [PMID: 9193722, scRNAseq] E3.5 PE
Lama1: [PMID: 9876177, IF] E8.5 PE.
Snai1:[PMID: 10853826, insitu] E5.5 PE, low level in EPC, not in PrE or VE. E6.5,PE, more in EPC, emerging mesoderm. E7.5 mesoderm wings and allantois, delaminating VE.
Thbd:[PMID: 7846065, 3036632, IF] detected in PYS endoderm, aortic arch, heart, blood vessels, lung buds and developing CNS.
Plat(tPA): [PMID: 2516796, WB] PE
Pth1r:[PMID:10330492, insitu] E5.5-E9.5 PE and PYS_endo
Fst:[PMID: 7821227,in situ]The primitive streak region(E6.5-E7.5), specific rhombomeres in the developing hindbrain(E8.5), somites(E8.5), paraxial mesoderm(E8.5) and parietal endoderm cells attached to the Reichert's membrane(E6.0-E8.5) showed strong expression of follistatin.
Sprac:[PMID: 3755680,WB] PE.
Stra6:[PMID: 9203140, insitu] E6.5-E7.5 PE. E8.5-E9.5, PYS, placenta but not trophoblastic giant cells. Not detected in PS, Low in presomatic mesoderm and LPM, IM, basal plate of neural tube.

Negative expression: **Cdh1**(PE cells grow with minimal cell-cell contact as they scattered on the inner surface of the giant cell layer)

————————VYS_endo enriched————————————————————————
Cited1(Msg1):[PMID: 9533950, insitu] E6.5, proximal EmVE and ExVE, low in ExE. E7.5, most proximal PS and nascent mesoderm. E8.0~E8.5, VYS_endo, mesoderm wing. Did not say it isn't express in PYS. *Msg1* is predominantly expressed in nascent mesoderm, the heart tube, limb bud and sclerotome.
Ihh:[PMID: 9242425, insitu] E6.5 VE, EPC, PE. E7.5-E8.5 VYS_endo, low in amnion.
Furin:[PMID: 9811571, insitu] E7.5 VYS(endo and meso), AVE(and underlying mesoderm) and node. E8.0, allantois, anterior intestinal portal(AIP, headfolds). E8.5 heart, AIP, foregut, LPM, tailbud.
Hnf4a:[PMID: 8052626, insitu] E4.5, PrE. E5.5, ExVE and EmVE, more in ExVE[PMID: 16728477 , insitu]. E6.5~E7.5, EmVE. E8.5, VYS_endo, liver diverticulum and hindgut. Later the mesonephric tubules, pancreas, stomach, and intestine and,In the metanephric tubules of the developing kidney.
α-fetoprotein (Afp): [PMID: 75937, IF] AFP is first detectable in E7.0. In 7th and 8th day embryos, AFP is confined to visceral endoderm cells around the embryonic region of the egg cylinder, more proximal EmVE in later stage. By the E9.0, AFP is also present in the extra-embryonic ectoderm, extraembryonic mesoderm and embryonic ectoderm cells around the three cavities of the embryo. On the 12th day of gestation AFP is confined to the endoderm layer of the visceral yolk sac. (the timing in this paper may be a bit late, E8.5 is more like E7.5). [PMID: 16708394, afp-gfp] E5.5, whole EmVE. By E6.5, those EmVE disperse proximally into ExVE (we will still consider

those cells as EmVE). By E7.5, those EmVE already takeover the ExVE space, and will become visceral yolk sac endoderm in the future. E9.5, it is also expressed in embryonic liver.
Ttr(Transthyretin): [PMID: 16728477, insitu] E5.5 VE. [PMID: 19415627, Ttr:RFP, insitu] E6.5 VE, more in ExVE. E7.5-E9.5 VYS_endo.
Apoa4, Lrp2, Fxyd2: [PMID: 28012457, insitu] E11.5, expressed in VYS endoderm cells.
Tdh, Lgals2,Cubn,Pla2g12b,Apoc2:[PMID: 17683524, insitu] E8.5, VYS_endo.
Esx1: [PMID: 10611245, insitu] E8.0-E9.5 chorion, VYS_endo

————————-Primitive endoderm(non VYS, non DE) enriched—————————————
Gata4: [PMID: 30530745, Gata4-GFP, IF] E3.5-E4.5 PrE. E5.5-E6.5 VE, more in ExVE[PMID: 16728477, insitu]. E7.25-7.5 VE residue, located in proximal part. [PMID: 9832509, insitu]E5.5-E6.5, VE and PE. Also abundantly expressed in nascent mesoderm. Precardiac splanchnic mesoderm and adjacent endoderm.
Gata6: [PMID: 9832509, insitu]E5.5-E6.5, VE and PE.

———————XEndo(non DE) enriched——————————————————————————
Rhox5(Pem):[PMID: 9832509, insitu] E6.5 EPC, PE,VE. Yolk sac
Dab2(Disabled-2):[PMID: 12413896, IHC] E4.5, PrE. E5.5-E7.5, VE and PE.

———-ExVE (and PE)enriched————————————————————————
Sox17:[PMID: 11973269, insitu] E6-E6.5 progressive expansion to whole ExVE. E7.0-E7.5, DE and ExVE. E8-E8.5, descending gradient of Sox17 expression from EPC to allantoic region of VE, enhanced expression in the DE(mainly in posterior gut).
Sox7: [PMID: 11973269, insitu] ExVE and PE, similar to Sox17, but not in DE.
Hnf1b(vHnf1):[PMID: 10518496, (heterozygous)Hnf1b-LacZ] E4.5 PrE. E 5.5-E6.25. ExVE, PE, AVE. E7.5 VYS_endo. E7.75-E8.0, few positive cells flank node(maybe DE, week), VYS_endo. E8.5, gut epithelium. E9.5-E10.5, mesonephric duct, pancreatic primordium.
Cited1(Msg1):[PMID: 9533950, insitu] E6.5, proximal EmVE and ExVE, low in ExE. E7.5, most proximal PS and nascent mesoderm. E8.0~E8.5, VYS_endo, mesoderm wing. Did not say it isn't express in PYS. *Msg1* is predominantly expressed in nascent mesoderm, the heart tube, limb bud and sclerotome.
Vegfa:[PMID: 1043382, KI-LacZ] E4.0 PrE. E5.5 ExVE and ExE, PE (lower, mainly in the proximal part). E6.5 ExVE. E7.5-E8.0 VYS_endo, XMeso (around the blood island, not in the allantois), not expressed in ExE and its derived structures (chorion, EPC). E8.0 Vegfa starts expressing in foregut pocket and hindgut. At day E8.5 the primitive gut, heart, and headfolds are all positive for LacZ/ VEGF expression.
Gata4: [PMID: 30530745, Gata4-GFP, IF] E3.5-E4.5 PrE. E5.5-E6.5 VE, more in ExVE[PMID: 16728477, insitu]. E7.25-7.5 VE residue, located in proximal part. [PMID: 9832509, insitu]E5.5-E6.5, VE and PE. Also abundantly expressed in nascent mesoderm. Precardiac splanchnic mesoderm and adjacent endoderm.

—————-EmVE (and DE) enriched——————————————————————————-
α-fetoprotein (Afp): [PMID: 75937, IF] AFP is first detectable in E7.0. In 7th and 8th day embryos, AFP is confined to visceral endoderm cells around the embryonic region of the egg cylinder, more proximal EmVE in later stage. By the E9.0, AFP is also present in the extra-embryonic ectoderm, extraembryonic mesoderm and embryonic ectoderm cells around the three cavities of the embryo. On the 12th day of gestation AFP is confined to the endoderm layer of the visceral yolk sac. (the timing in this paper may be a bit late, E8.5 is more like E7.5). [PMID: 16708394, afp-gfp] E5.5, whole EmVE. By E6.5, those EmVE disperse proximally into ExVE (we will still consider those cells as EmVE). By E7.5, those EmVE already takeover the ExVE space, and will become visceral yolk sac endoderm in the future. E9.5, it is also expressed in embryonic liver.
Foxa2, Lhx1(Lim1): [PMID: 16728477, insitu]E5.5 EmVE. [PMID: 9431803, insitu] E6.5 Lim1 in AVE and posterior proximal emVE, subsequently, expression is seen in the PS and its derivatives as well as in the mesodermal wings and prechordal plate. E7.5 FoxA2 in AVE and EmVE. Later this message is expressed in the node, notochord, floor plate, prechordal mesoderm and DE.
Fgf5, Fgf8: [PMID: 16728477, insitu] E5.5 EmVE.
Eomes: [PMID: 33574614, IF, Eomes-GFP] E6.5, ExE, EmVE and PS. E7.5, XMeso(and amnion), ExE(chorion), Mesoderm, VYS_endo

————-AVE/DVE enriched——————————————————————————————

Cer1: [PMID: 9431803, insitu] DVE, AVE before gastrulation. E7.5 Endoderm overlying the neural plate proximal to the node, include anterior gut endoderm, mesoderm from prechordal plate and notochord, but not in the node or precardiac mesoderm. ~E8.0 Upregulated in forming somites and adjoining presomitic mesoderm. E8.5 *cer-l* expression is restricted to the last two formed somites and presomitic mesoderm.

Cited2(Mrg1): [PMID: 9533950, insitu] E6.5-E7.5, a ring of proximal VE, and more in AVE.

Lefty1: [PMID: 15004567 insitu] DVE, AVE before gastrulation. [PMID: 9348041, insitu]E8.5 prospect floor plate.

Hhex(Hex1):[PMID: 11397001, Hex::GFP] E5.5 DVE. E6.5 AVE, axial mesendoderm. ~E7.5 AVE, AME and Blood island. ~E9, the endothelial cell precursors, notochord, foregut diverticulum.

Hesx1(Rpx): [PMID: 8939602, insitu], E6.5-E7.5, AVE, E7.0-E8.0 Ectoderm under AVE (incipient headfold, neural plate). E8.5, only in headfold. ~E9.0, prosencephalic neurectoderm, oral ectoderm and foregut endoderm. E9.5, ventral diencephalon

Otx2: [PMID: 11171400, insitu] E5.5 Otx2 is expressed diffusely in both the epiblast and the visceral endoderm. From the early primitive streak to headfold stages, the expression of otx2 becomes restricted to the anterior end of embryo proper and the underlying endoderm.

Dkk1:[PMID: 16256739, insitu] E5.5 AVE

Amot: [PMID: 12676095, insitu] E6.5 AVE. E7.5 VE.

Lhx1(Lim1) :[PMID: 16728477, insitu] E6.5 Lim1 in AVE and posterior proximal emVE, subsequently, expression is seen in the PS and its derivatives as well as in the mesodermal wings and prechordal plate.

———DE enriched (and VE/PE)——————————————————————————

Sox17:[PMID: 11973269, insitu] E6-E6.5 progressive expansion to whole ExVE. E7.0-E7.5, DE and ExVE. E8-E8.5, descending gradient of Sox17 expression from EPC to allantoic region of VE, enhanced expression in the DE(mainly in posterior gut: midgut and hindgut).

Foxa2: [PMID: 16728477, insitu]E5.5 EmVE. [PMID: 9431803, insitu] E7.5 FoxA2 in AVE and EmVE. Later this message is expressed in the node, notochord, floor plate, prechordal mesoderm and DE.

Irx1: [PMID: 9486539, insitu] E7.5 mesoderm wing outlying headfold, distal patch of definitive endoderm cells. E8.5-E9.5 Midbrain, hidbrain region, foregut.

Cer1: [PMID: 9431803, insitu] DVE, AVE before gastrulation. E7.5 Endoderm overlying the neural plate proximal to the node, include anterior gut endoderm, mesoderm from prechordal plate and notochord, but not in the node or precardiac mesoderm. ~E8.0 Upregulated in forming somites and adjoining presomitic mesoderm. E8.5 *cer-l* expression is restricted to the last two formed somites and presomitic mesoderm.

———Gut endo enriched——————————————————————————————

Pyy, Nepn(5730521E12Rik): [PMID: 17683524, SAGE, insitu] Somites 0-6 endoderm. From somites 2 to somites 8 stage, Pyy is gradually expanded from small lateral region of the DE to whole ventral foregut region. E8.5-E9 Nepn is highest in the midgut. At E9.5 Nepn is retained but is down-regulated.

Krt19, Foxa1: [PMID: 22236333, insitu, qRT-PCR][PMID: 11062258, Krt19-lacZ] E7.5 Krt19 is expressed in notochord plate, EPC. E8.5 arch pattern in hindgut region, notochord. E9.0 primitive gut epithelium, notochord.

Sox2:[PMID: 10446282, insitu] E8.5-E9.0 Sox2 is expressed throughout the gut endoderm. Sox3 is detected in the posterior region of the foregut.

Negative expression: **H19**([PMID: 1811930, insitu]The expression of this gene in extraembryonic lineage is temporally and spatially restricted in extraembryonic lineages. At E8.5, H19 expression is seen in embryonic organs(heart, intestine and liver)); **Rhox5(Pem)**([PMID: 7958444, insitu], from preimplantation to post-implantation, strongly expressed in extraembryonic tissue). **Cer1** and **Lhx1** also down-regulated during the morphogenesis of embryonic gut.

———Pharyngeal_endo—————————————————————————————-

Isl1: [PMID: 23906961, KI-LacZ, IF]: E8.5, pharyngeal mesoderm(dorsal to the heart tube) and endoderm, foregut. E9.5, add umbilical cord and surround meschenchyme. E10.5-E15.5, central

and peripheral nervous system, neural retina, inner ear, pharyngeal mesoderm and endoderm and their derivatives (craniofacial structures, thymus, thyroid gland and trachea), cardiovascular system (cardiac outflow tract, carotid arteries, umbilical vessels, sinoatrial node and atrial septum), gastrointestinal system (oral epithelium, stomach, pancreas, mesentery) and hindlimb. NOT in Heart tube.
Nkx2-3, Nkx2-1, Nkx2-6, Nkx2-5, Pax9: [PMID: 12141427, insitu] Nkx2-3 was expressed in the pharyngeal floor and pouches, as well as in oral and branchial arch ectoderm. E8.0, expressed in ventral endoderm of foregut pocket. E9.0, pharyngeal endoderm and visceral mesoderm. E8.0-8.25 Nkx2-6 expression in pharyngeal floor endoderm and cranial mesoderm. E9.5 Pax9 expression in dorsal and ventral aspects of the second pair of pharyngeal pouches. E9.0 expression of Nkx2-1 restricted to the midline region of the pharynx.
Gfpt2, Tnnt1,Has2,Kctd12b,Foxe1: [PMID: 30787436, scRNAseq]
Pax1: [PMID: 16752393, insitu] E8.5, anterior most foregut(bifurcate pattern from dorsal). E9.5 branchial pouch.
Pax9, Has2: [PMID: 33106598, insitu] mots anterior foregut.

———Anterior_foregut————————————————————————————————-
Hoxb1,Hoxa1:[PMID: 21385575, insitu] E8.5 Hoxb1 and Hoxa1 are expressed in both the splanchnic mesoderm and ventral and lateral foregut endoderm.
Osr1,Ripply3,Hoxa1,Irx2,Fzd2,Irx1: [PMID: 30787436, scRNAseq]
Tbx1,Prrx2: [PMID: 17683524, insitu]
Rhou: [PMID: 21903671, insitu] E8.0 Rhou is expressed strongly around the rim of the anterior intestinal portal and allantois. E8.5 expressed on the lateral and ventral wall of the foregut but is down-regulated in the epithelium that will contribute to the liver bud(posterior foregut). E9.5 endoderm of pharyngeal pouches and lateral wall of the upper foregut.
Irx1, Irx2: [PMID: 11472847, insitu] E8.5 Irx1 is expressed in midbrain and foregut at level of lung/heart. Irx2 express in hindbrain and week in foregut at the dorsal-caudal side of the heart. Later both Irx1 and Irx2 are detected in the lung development.
Rarb: [PMID: 9097025, insitu]

———Posterior_foregut (level of AIP)—————————————————————————
Hhex(Hex): [PMID: 9389666, insitu] ~E8.0 involuting ventral foregut endoderm (but not in the anterior extremity of the foregut pocket), clusters of angioblasts and in the allantois. E8.5, positive in angioblasts detected throughout the lateral mesoderm and cranial mesenchyme, thyroid and liver primordia progenitors.
Pyy: [PMID: 17683524, insitu] Pyy expression is seen in small lateral region of the DE at as early as 2 somite stage. At the 4 somite stage, the expression domains in the lateral region are expanded. The lateral expression domains expanded and extended anteriorly to the medial ventral foregut. In the early organogenesis stage, the Pyy expression remained in the posterior foregut extending to the midgut junction.
Pdx1:[PMID: 19141476, Pax1Cre] E9.5-E10.5, Pax1 in both ventral and dorsal pancreatic buds.
Myc, Onecut2, Prox1, Tbx20: [PMID: 17683524, scRNA]

————————MidGut———————————————————————————————
Nepn, Nudt11,Pdzk1ip1,Hacd4,Prss8, B4galt6,Kitl: [PMID: 17683524, scRNA]
Nepn: [PMID: 17683524, insitu]
Ihh: [PMID: 11973269, insitu]

———Hindgut——————————————————————————————————-
Tfpi,Trf,Xlr3a,Trap1a,Rhox5,Cited1,Cdx1,Hoxb2,Zfp503,Stra6,Mnx1,Tlx2,Hoxc9,Axin2,Wnt5b, Smim3,Cdx2: [PMID: 17683524, scRNA]
Cdx4: [PMID: 7734393, insitu]

——-Node&Notochordal plate—————————————
Chordin(Chrd):[PMID: 12810603] E7.0 expression extends from the rostral limit of the notochord to the node. After embryo turns, chordin expression are present in the dorsal endoderm adjacent to the notochord. ~E9.5 the axial expression of chordin disappeared from most of the trunk and tail, but it is still strong in the pharynx, chordoneural hinge of the tail bud and postnatal gut.

Shh, Ptch1: [PMID: 16564040,8595881, insitu]
Foxa2: [PMID: 8069910, Foxa2-LacZ]
T: [PMID: 10617567, insitu]
Noto: [PMID: 15231714, insitu] E7.5 Node region. E8.0-E8.5 caudal region of notochord.
Dand5(Cer2):[PMID: 23544137,insitu]
Fgf5:[PMID: 1794310, PMID: 1794311] E5.25 ICM, E7 VE, Epiblast, Mesoderm, node.

③ <u>XMeso</u>

Description:

The extra-embryonic mesoderm will form the mesodermal component of the **visceral yolk sac(VYS_meso)**; it will also generate the mesoderm compartment of the **Amnion, Chorion** and **Allantois**. They contribute to the generation of the primitive vasculature and red blood cells. The allantois in mammals forms the blood vessels of the umbilical cord and provides the embryonic link to the placenta through fusion with the chorion.

- Besides PS, allantois may also have some visceral endoderm derived cells (AX).
- Chorion_meso, Amnion_meso and Allantois_surface are very similar (the lining of exocoelm)
- Hemangioblasts in the posterior streak produce more restricted progenitors, including EryP progenitors, angioblasts, and EMPs.

**E6.5**	Posterior_PS, ACD&VOC
**E7.0**	Posterior_PS, ACD&VOC, VYS_meso_early, Hemogenic_angioblasts, EryP_progenitors, EryP,
**E7.5**	ACD&VOC, VYS_meso_early, Hemogenic_angioblasts, EryP_progenitors, EryP, Amnion_meso, Allantois_surface
**E8.0**	Hemogenic_angioblasts,Hemogenic_endothelium,EryP, EMPs, Chorion_meso, Amnion_meso, Allantois_surface
**E8.5**	Hemogenic_angioblasts,Hemogenic_endothelium,EryP, EMPs, ECs, Chorion_meso, Amnion_meso, Allantois_surface

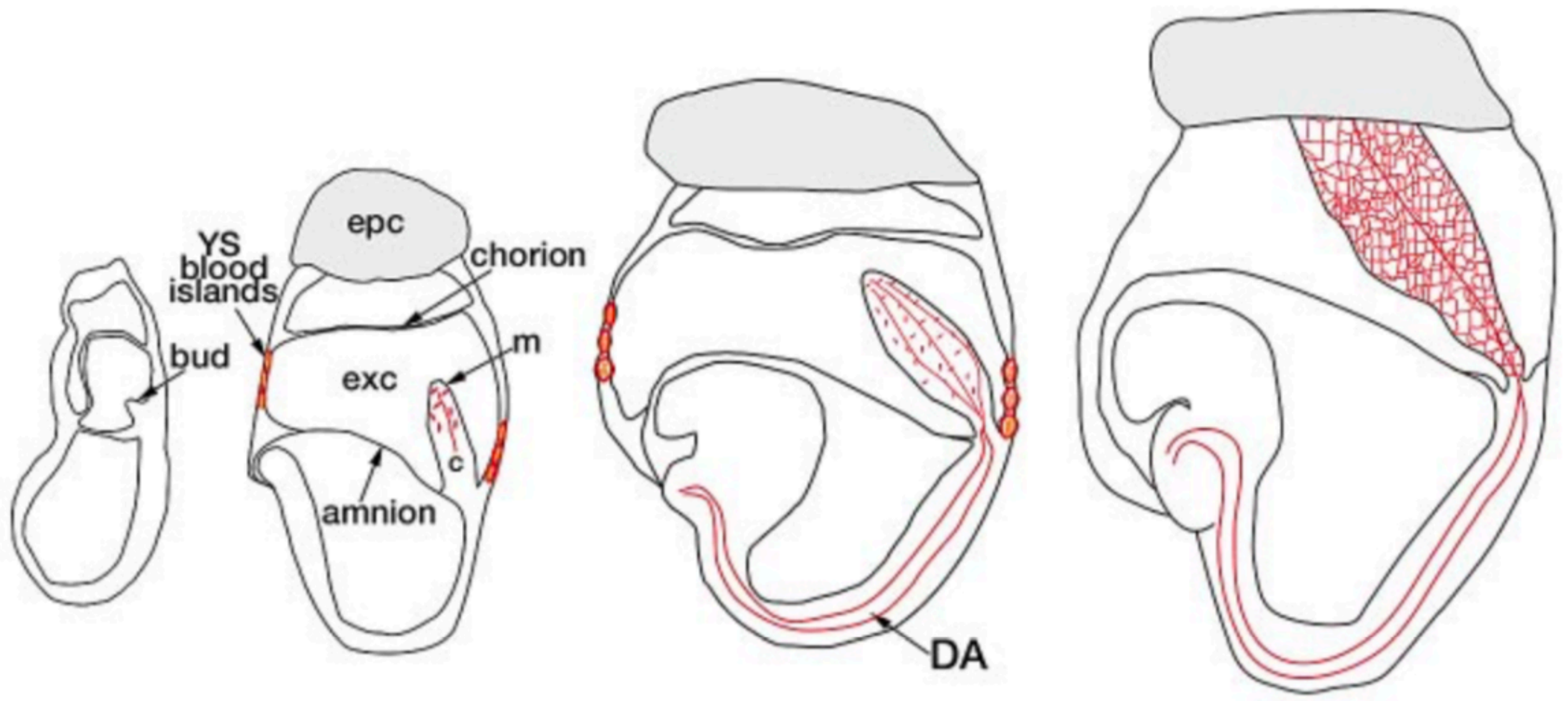

Important biological process:

Yolk-sac Hematopoiesis & Vasculogenesis: The first site of blood cell production in the mouse embryo is the yolksac, where mesodermal cell masses called blood islands arise from <u>VYS_meso</u> between E7.0-E7.5, before the formation of the vasculature. Although those first progenitor cells

express endothelial markers including Tek, Cdh5 and Pecam1, they are not localized within the lining of a blood vessel, but rather within a mass of endothelial-expressing cells, thus in our annotation, I propose those cells should be termed **"Hemogenic_angioblast" (Primitive hemagioblasts/hemagioblasts)**. And those cells expressing some endothelial markers but do not possess the ability to generate primitive erythroblasts are termed **"Angioblasts" (endothelial progenitor cells)**.

By E8.0-E9.0, the outer layer of the blood islands differentiate into mature **endothelial cells (ECs)**, while the inner cells differentiate into Kdr-negative **Erythroid_progenitors**, which give rise to **macrophage, megakaryocyte** lineages and **Primitive_erythroblasts (EryPs)**, which progressively lose their intercellular attachments. Primitive erythroid cells express embryo-specific β-globins, such as (εy, βH1) and an α-globin (ζ), but only low levels of adult β-globins (βmajor, βminor) and α-globins (α1, α2).

The yolk sac vasculature is formed by the fusion of blood islands at E8.0, and at E8.25, a second wave generates **Hemogenic_endothelium**, which give rise to **Definitive_erythroblasts (EryDs)**, E8.25 **Erythroid-myeloid progenitors (EMPs)**, E9.5 T and B progenitors, and E10.5 HSCs. EMPs are the common progenitors for erythro-myeloid lineages in the yolk sac, and they are HSC-independent wave of hematopoietic progenitors. EMPs also contains robust primitive/definitive erythroid fate and potential. EMP emerge in the yolk sac by endothelial-to-haematopoietic transition (EHT). YS-derived primitive macrophages will spread into the embryo proper through the blood after the circulatory system has fully established (from E8.5 to E10), and migrate to various tissues, including brain (microglia). The third wave of hematopoiesis is mainly driven by HSC, which is beyond time of our dataset (schematics are shown below, [PMID: 28043822]). In our dataset, I did not differentiate EryPs and EryDs, together the cluster is called Erys, which may also contain macrophages, megakaryocytes, granulocytes and lymphocytes (the cells may be too few to establish an intact cluster).

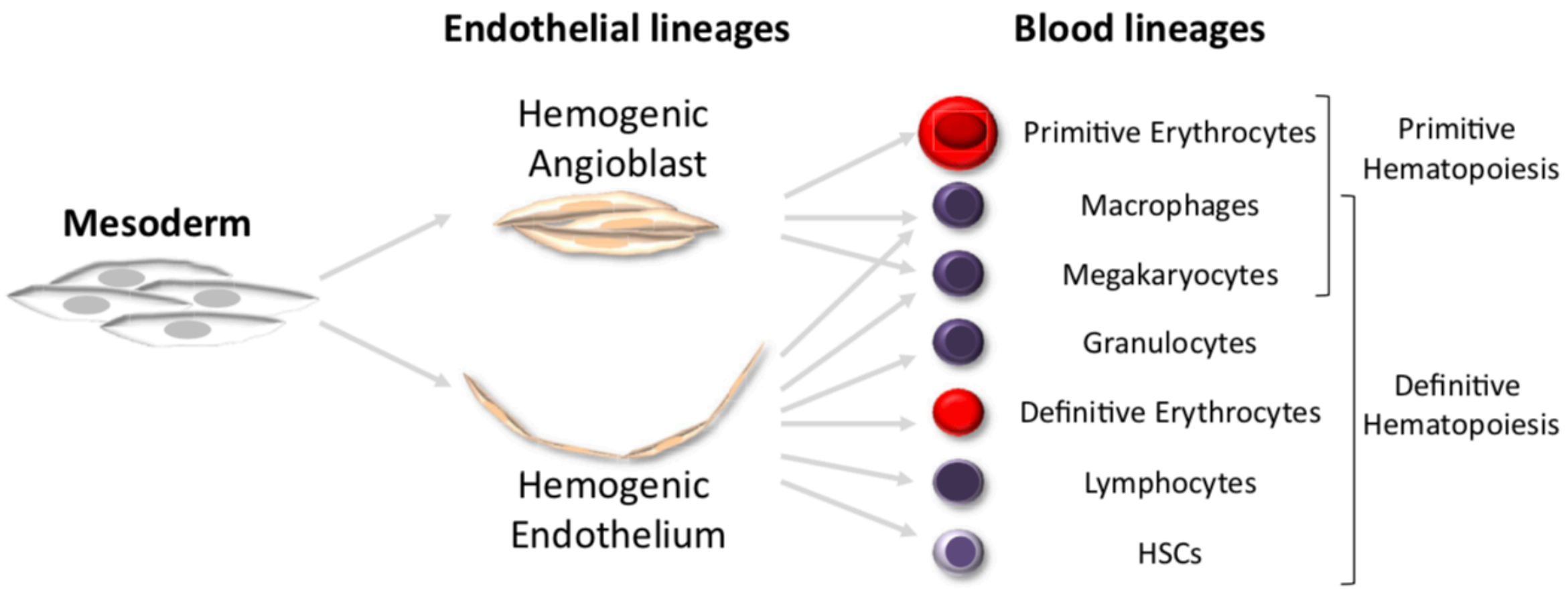

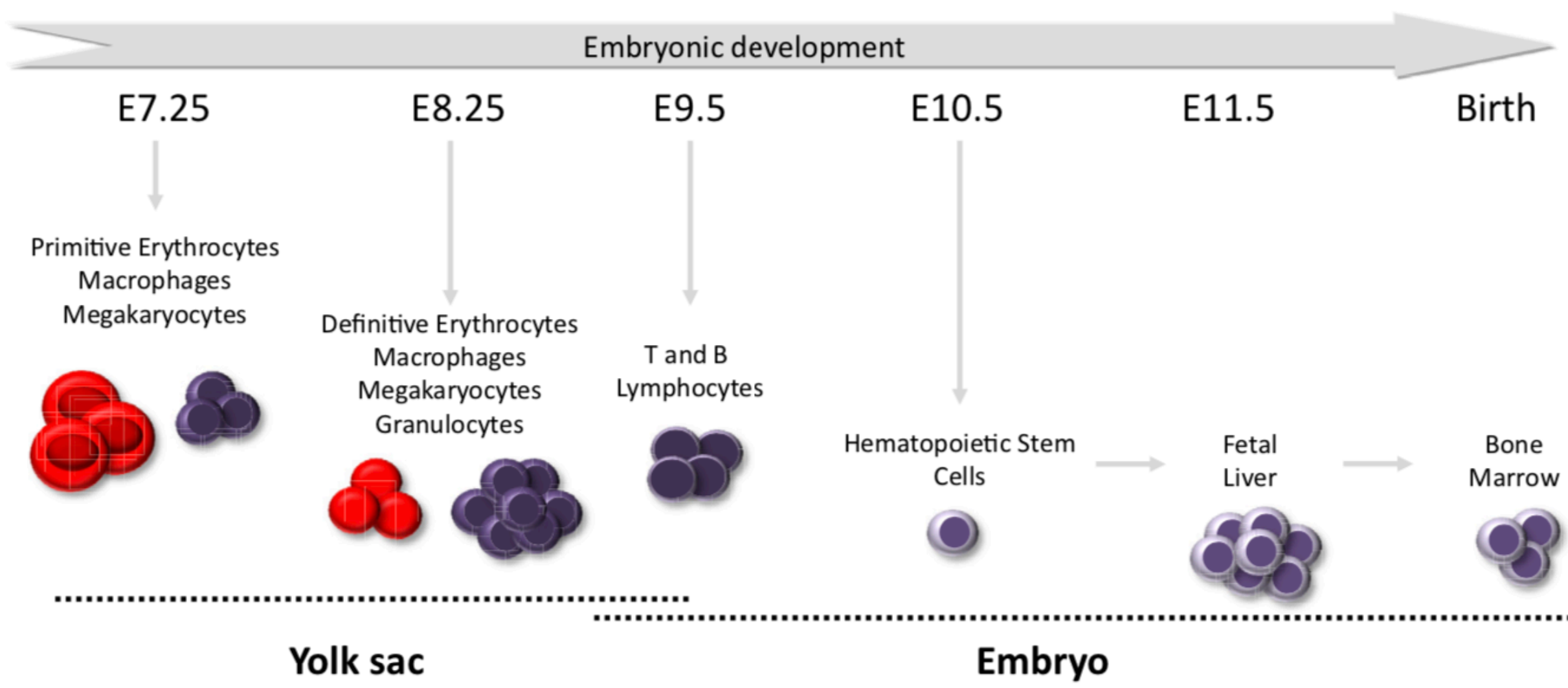

Intraembryonic vasculogenesis: This process is initiated at approximately E7.5, when the primordia of the endocardium begins to form at the cranial region of the embryo. The endocardium is the innermost layer of the heart and blood vessels.

Allantoic Vasculogenesis: The allantois appears during the final stages of exocoelom formation in mice, positioned at the posterior interface between the yolk sac and amnion. At E7.0, The extraembryonic extension of the primitive streak expands into a dense core, **the allantoic core domain (ACD,** also called **extra-embryonic PS, XPS**), under influence of the VYS_endo nearby. It is also said that those VE cells incorporate into ACD by EMT and contribute to the hindgut lip. Allantoic bud formation occurs at E7, followed by the emergence of the allantois at E7.75 and umbilical cords at E8.25. The allantoic vasculatures include a distal branched vascular network, the proximal unbranched umbilical artery, and the vessel of confluence (VOC), which is designated by ACD. The nascent VOC, becomes incorporated into the allantoic bud and exhibits a variety of gene products involved in the formation of hemangioblasts and hemogenic endothelium. By approximately E8.25, the major arterial blood vessels are forming in the proximal region, while chorio-allantoic union begins in the distal region.

*Our hypothesis is that blood cells derived from distinct sources will have a unique transcriptome that distinguishes them from other lineages, leading to their separate convergence.
*Strictly, vasculogenesis here include angiogenesis.

Amnion morphogenesis: At E8.5, the U-shaped embryo turns along its dorsoventral axis and becomes enclosed in the amniotic sac, which is surrounded by the yolk sac.

Allantois morphogenesis: Outer cells of allantois form a simple squamous epithelium, called the mesothelial layer of the allantois (also called proximal flanks or **Allantois_surface**) that overlies inner cells referred to as the core (ACD). In contrast to ACD and allantois vasculogensis, those mesothelial layer is supplied by cells from **IPS (intra-embryonic PS**, **posterior_PS** in our dataset). Densely packed ACD is directly continuous with the PS, while the outer mesothelium is continuous with that of the yolk sac and amnion.

Expression pattern:
—————Allantois_ACD/XPS/VOC————————————-
T: [PMID: 35266581, IF] E7.5, ExE, Notochord, PS, ACD.
Ifitm3:[PMID: 35266581, scRNAseq/IF] E7.5 ACD.
Pou5f1(Oct3/4):[PMID: 18213575, Ihc] EB-5s, express in the allantois and allantois vasculature, associated visceral endoderm, and hindgut endoderm.
Kdr(Flk1): [PMID: 7596435, Kdr-LacZ]

Low marker: **Mki67(Ki67)**[PMID: 28882402, IF]

——————VYS_meso(the lining of exocoelom except blood) enriched——————————
Krt8,Krt18:[PMID: 35266581,transgene] E6.25-E7.75, expressed high in VE, ExE, XMeso.
Cdh11, Nrp1:[PMID: 35266581, IF] E7.5, Amnion_meso, Chorion_meso, XMeso that lining the exoceolm, and allantois surface.
Hand1:[PMID: 9500550, KI-LacZ] E7.5 all lining of exocoelom. E7.75, also in cardiac crescent. E9.5 expression can be seen throughout the LM, as well as within the left ventricle of the heart.
Foxf1: [PMID: 36252226, scRNAseq]
Fgfr2: [PMID: 10330500, insitu]

Negative marker: **Eomes**[PMID: 10330500, insitu]

*Note that mesoderm of Chorion, Amnion and Allantois surface are derived from VYS_meso, so that they all share similar transcription signature.

————-Amnion_meso enriched——————————————————————
Postn: [PMID: 22966238, insitu/qRT-PCR] E8.5 Amnion_meso.

Acta2,Tagln,Col1a1,Dlk1,Nrp1,Vcan: [PMID: 35266581, scRNAseq]
Bmp2,Pitx2,Pitx1,Acta2,Tagln,Bmp4:[PMID: 36252226,scRNAseq]
Tbx20:[PMID: 10940636, insitu] E7.5 both allantois and amnion. E7.75 cardiac crescent and allantois. E8.0 developing heart tube. E8.5-E9.5 looping heart tube, LM, optic vesicle and neural tube.

————Allantois_surface————————————————————————————————————
Vcam1: [PMID: 7539359,7530222,IHC] E8.5 Allantois distal
Vegfa: [PMID: 7647376,insitu] E8.5 VE, DE and mesothelial layer covering the allantois.
Tbx4:[PMID: 21932311, Tbx4-cre] E9.5 Hindlimb field, all cells of the mesothelial layer of allantois. After remodelling of the allantois, Tbx4 expression surrounds both umbilical vessels, but the endothelium is Tbx4 negative. [PMID:8853987, insitu] E8.5-E12.5 Allantois and hindlimb.
Tbx20:[PMID: 10940636, insitu] E7.5 both allantois and amnion. E7.75 cardiac crescent and allantois. E8.0 developing heart tube. E8.5-E9.5 looping heart tube, LM, optic vesicle and neural tube.
Hoxa10,Hoxa11: [PMID: 22219351, insitu] E7.5-8.5 Allantois base
Pitx1,Twist2,Prrx2,Plac1*:* [PMID: 36252226,scRNAseq]
Cdx2:[PMID: 35266581, IF] Amnion, surface AL.

———Chorion_meso————————————————————————————————————
Fn1,Acta2,Dlk1,Tagln: [PMID: 36252226,scRNAseq/IF] low compare to Amnion.
Nr2f2(CoupTFII): [PMID: 20195529] Human CTB differentiation.
Rbpj: [PMID: 31165749, insitu] E8.0-E10.5 chorion plate.

High marker: BMP targets(**Msx1,Msx2, Id1, Id2**)
Negative marker: **Postn**

————- Hemogenic_angioblasts enriched——————————————————————————
Sox7, Rasip1, Dll4, Icam2, Sox18, Exoc3l, Flt4, Gja4, Cldn5, Arap3, Abi3, Rhoj, Prkch, Ctsh, Esam, Hhex [PMID: 35266581, scRNAseq]
Kdr(Flk1):[PMID: 12922023, Kdr-lacZ] E8.5 exclusively in the endothelium. Kdr seems to mark all embryonic endothelial cell progenitors, but only a subset of Kdr-expressing cells can also make hematopoietic cells. [PMID: 7647376, insitu] E7.0 Endothelial cells of VYS.
Etv2, Tal1(Scl),Gata2: [PMID: 24052951,in vitro]
Cd34: [PMID: 7840769, review]
Tek: [PMID: 9846489, insitu]

—————-EryP progenitor enriched(also in primitive erythroblasts)———
Tal1:[PMID: 10688823, IF]
Gata1, Gata2, Tal1, Lmo2, Runx1: [PMID: 20711979, 22337720, insitu] E7.5 expressed in emerging blood islands.
Spi1, Gfi1b: [PMID: 29311656, scRNA]
Negative marker: **Kdr** [PMID: 10688823, IF]

————-Primitive erythroblasts(EryP) enriched——————————————————
Hbb-bh1,Hbb-y: [PMID: 20201948,review]
Hba-x, Lmx1a: [PMID: 29540203, scRNA]
Gata1, Nfe2: [PMID: 29311656, scRNAseq]
Tfrc(Cd71), Cd24a, Cd55, Bsg(Cd147): [PMID: 16940424, cytoflow] Exist on E9.5 EryP surface.

Negative marker: [PMID: 16940424, cytoflow] **Pecam1**(Cd31), **Kit**, **Flt3**(Flk2), **Cxcr4**, **Slamf1**(Cd150), **Kdr, Itga4, Itga5,Itga6, Cd44, Plvap**(Meca32).
Low marker: **Runx1**:[10226014, KI-LacZ]: E8.5 blood island, in both endothelial cells and hematopoietic cells. Relatively low in EryP. Expression also seen in vitelline artery endothelial cells. [PMID: 12049718, PMID: 10226014, transgene] Runx1 protein is first detected in the mesenchymal cells in the yolk sac at E7.5 and in both primitive erythrocytes and endothelial cells in the yolk sac

blood islands at E8.0. Runx1 levels decline in primitive erythrocytes and almost all endothelial cells by E8.5, but remain high in a small number of endothelial cells and blood cells scattered throughout the yolk sac blood islands. Also E8.5, Runx1 expression is detected in three new sites: distal tip mesenchymal cells of allantois, endothelial cells in the vitelline artery, and in endothelial cells in the ventral portion of the paired dorsal aortae within what will become the AGM region. Between E9.5 and E11.5, Runx1 is expressed in endothelial cells in the V?U arteries, in both endothelial cells and mesenchymal cells in the ventral portion of the AGM region, and in a small number of endothelial cells in the yolk sac, but in no other endothelial cells in the embryo.

———-**Hemogenic endothelium enriched**————————————————————————
Lyve1: [PMID: 27880904, IF/cre] E8.5 Yolk sac vasculature. E9.5 blood island endothelium, co-express with Pecam1. E10.5 Cardinal vein, viteline vasculature.
Cdh5,Pecam1, Cd34, Tek(Tie2): [PMID: 10688823, IF] E7.8 aortic primordia; E8.3 vessel primordia and vascular networks in the allantois.
Aplnr, Flt4, Ephb4:[PMID: 21793101, insitu]

Negative marker: **Etv2** [PMID: 29753018] more involved in lymphagiogenesis.

————-EC enriched——————————————————
Cdh5, Pecam1, Cd34, Tek(Tie2): [PMID: 10688823, IF] E7.8 aortic primordia; E8.3 vessel primordia and vascular networks in the allantois.
Dll4, Unc5b, Hey1, Efnb2, Gja4(Cx37), Gja5: [PMID: 35079138, scRNA]
Kdr: [PMID: 10688823, IF]
Efnb2, Nrp1, Hey2, Notch1, Notch4, Jag1, Ephb4:[PMID: 21793101, insitu] an arterial and venous signature consistent with their endothelial identity.

Low marker: **Tal1**[PMID: 10688823, IF]

————- **EMP enriched**——————————————————————————
Runx1, Spi1(PU.1), Gfi1b, Tspan32, Sla2, Alox5ap, Fcer1g, Itgb3, Itga2b, Tmem163, Ptgs1, Rab27b, Nfe2, F10, Alox5, Csf1r:[PMID: 29311656, scRNA]
Fcgr3: Ptprc(Cd45), Kit [PMID: 30787436, scRNA]
Adgrg1, Cx3cr1: [PMID: 29540203, scRNA] they state Csf1r and Cx3cr1 as primitive macrophage marker, and Itga2b and Adgrg1 as myeloid progenitor marker, kit as EMP marker
Itgam(CD11b),Cd68: [PMID: 27036090, review] macrophage population marker

④ <u>Neural_Ecto</u>

Description:

At ~E7.5, The **neural plate** develops as a thickened region of embryonic ectoderm overlying the notochord and prechordal plate. Closure of the neural tube is initiated in the cervical region at the hindbrain-cervical boundary and the proceeds cranially and caudally. Different regions of the neural tube form by **primary and secondary neurulation**. In primary neurulation, the flat neural plate proliferates and invaginates, creasing inward until the elevating edges come in contact and fuse to form a cylindrical, hollow neural tube. Secondary neurulation occurs in the caudal section where the solid cord of cells sinks into the embryo and cavitates to form a separate hollow tube in the tail bud. The tubes formed by the primary and secondary neurulation eventually connect. The cephalic end of the neural tube has three primary brain vesicles: **prosencephalon (forebrain)**, **mesencephalon (midbrain)** and **rhombencephalon (hindbrain)**. The prosencephalon subdivides into the **telecephalon (cerebral hemispheres)** and **diencephalon** (consisting chiefly of the **thalamus** and **hypothalamus**). The mesencephalon is the most rostral brain domain in which the neural boundaries for organizing **brain nuclei** (i.e., the alar and basal plates, the sulcus limitans) remain distinct. The rhombencephalon subdivides into the **metencephalon (cerebellum and pons)** and the **myelencephalon (medulla oblongata)**. The post-cephalic region of the neural tube remains a hollow cylinder and develops into the **spinal cord.**

The analysis of clones from single epiblast cells directly labelled at later time points reveals that the anterior and posterior CNS lineages become separated at ~E7.5 in mouse embryo, with epiblast cells located rostral to the node generated neural-only clones that contributed to the more anterior hindbrain; epiblast cells from anterior two-thirds of the epiblast at this start age map to forebrain and hindbrain; by contrast, clones descended from epiblast cells closer to the node contributed to regions posterior to the hindbrain and included **neuralmesodermal progenitor cells (NMPs)**, which drives secondary neurulation by contributing to both spinal cord and paraxial mesoderm. The extent to which NMPs contribute to the CNS remains controversial. Moreover, it has not been determined whether the trunk neural tissue that is generated transit through an initial anterior neural state or arise independently by a process involving the formation of NMPs. Personally my view over this question is that anterior CNS and posterior CNS are specified separately but the maintaining and further development of anterior CNS need the signal released from posterior CNS.

All states: NMPs, NeuroEpithelium_posterior, NeuroEpithelium_anterior, Neural_crest, Hindbrain, Midbrain, Forebrain, Floor_plate, Neurons

State relationships and assumptions:
- NMP contributes to neuroepithelium_posterior and neuroepithelium_anterior, which also participates in the floorplate.
- In our annptation, the neuroepithelium_anterior and neuroepithelium_posterior are more like subdivision of future spinal cord population.

**E8.0**	NMPs, NeuroEpithelium_posterior, NeuroEpithelium_anterior, Neural_crest, Hindbrain, Midbrain, Forebrain, Floor_plate
**E8.5**	NMPs, NeuroEpithelium_anterior, Neural_crest, Hindbrain, Midbrain, Forebrain, Floor_plate, Neurons

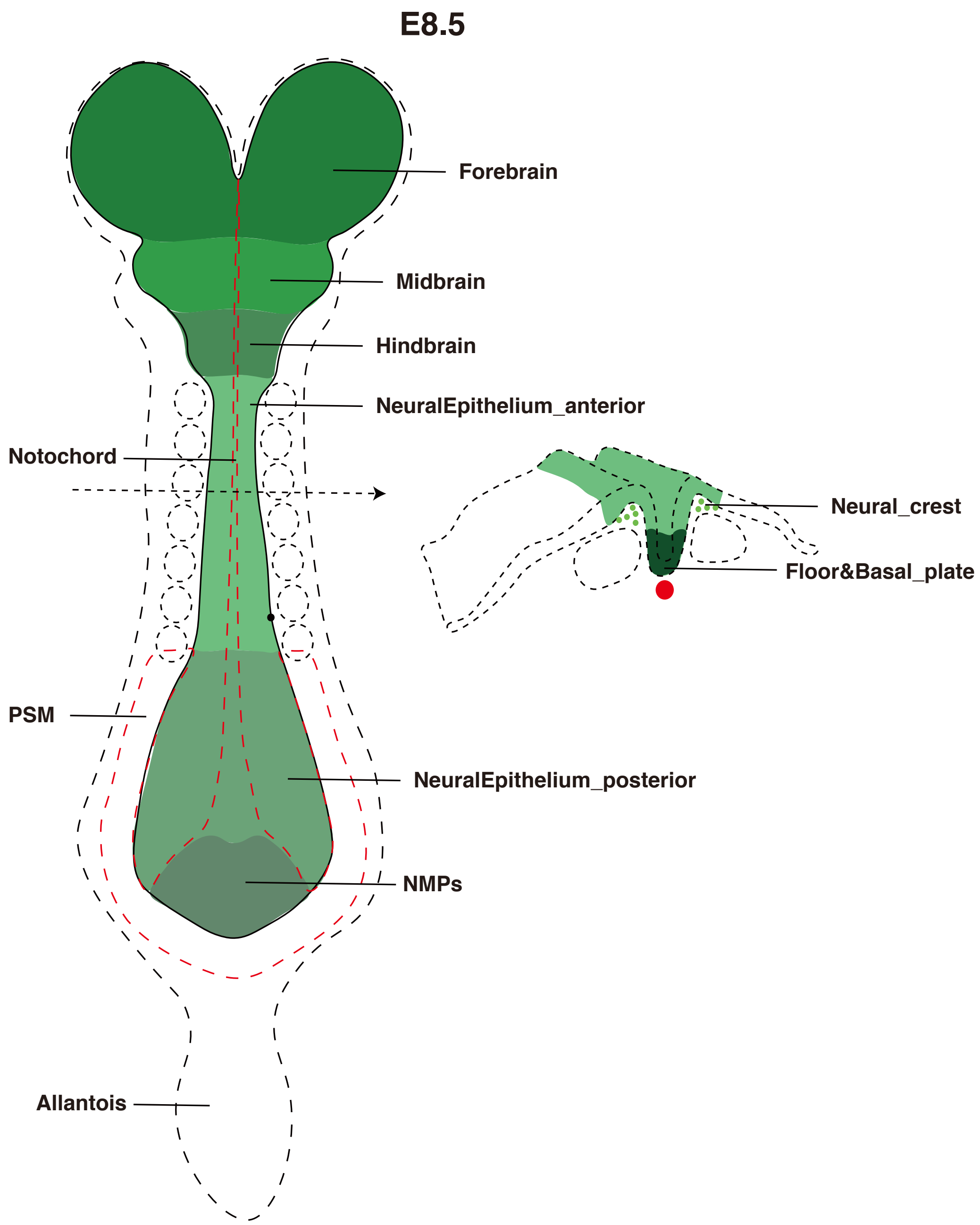

Important biological processes:

Neural tube closure: At ~E7.5 signals from notochord induce the overlying embryonic ectoderm to thicken and form the neural plate, which morphs through directed cellular migration to form a structure that is narrow mediolaterally and elongated cranio-caudally (but notochord is not required for the formation of neural tube). The cranial and lateral edges of the neural plate are clearly defined by raised borders, but caudally the neural plate merges into the tailbud. Toward the end of this stage, the lateral edges of the neural plate begin to elevate while a shallow neural groove forms in the midline of the embryonic axis. This elevation takes place all along the central axis, but the prominence of the tube walls at the cephalic end of the plate results in the production of enlarged **head folds** as the first structural landmark of 'cephalization'. At ~E8.0 the neural groove is deepened and head folds are prominent. The apices of the cephalic neural fold remodel to create two epithelial layers: the outer **surface ectoderm** and inner **neuroectoderm**. The fusion of neural folds occurs at the level of the fourth and fifth somites. Neural fold fusion begins at two additional sites in the cephalic region: By E8.5, Closure 2, at the forebrain-midbrain boundary and which progresses bidirectionally, and Closure 3, at the rostral extremity of the forebrain and which progresses caudally. By E9.0, the caudal progression of Closure 3 meets the rostral progression of Closure 2, completing the formation of rostral neural tube. Caudal

neuropore is sealed by E9.5~10. From ventral to dorsal, the neural tube can be divided into four

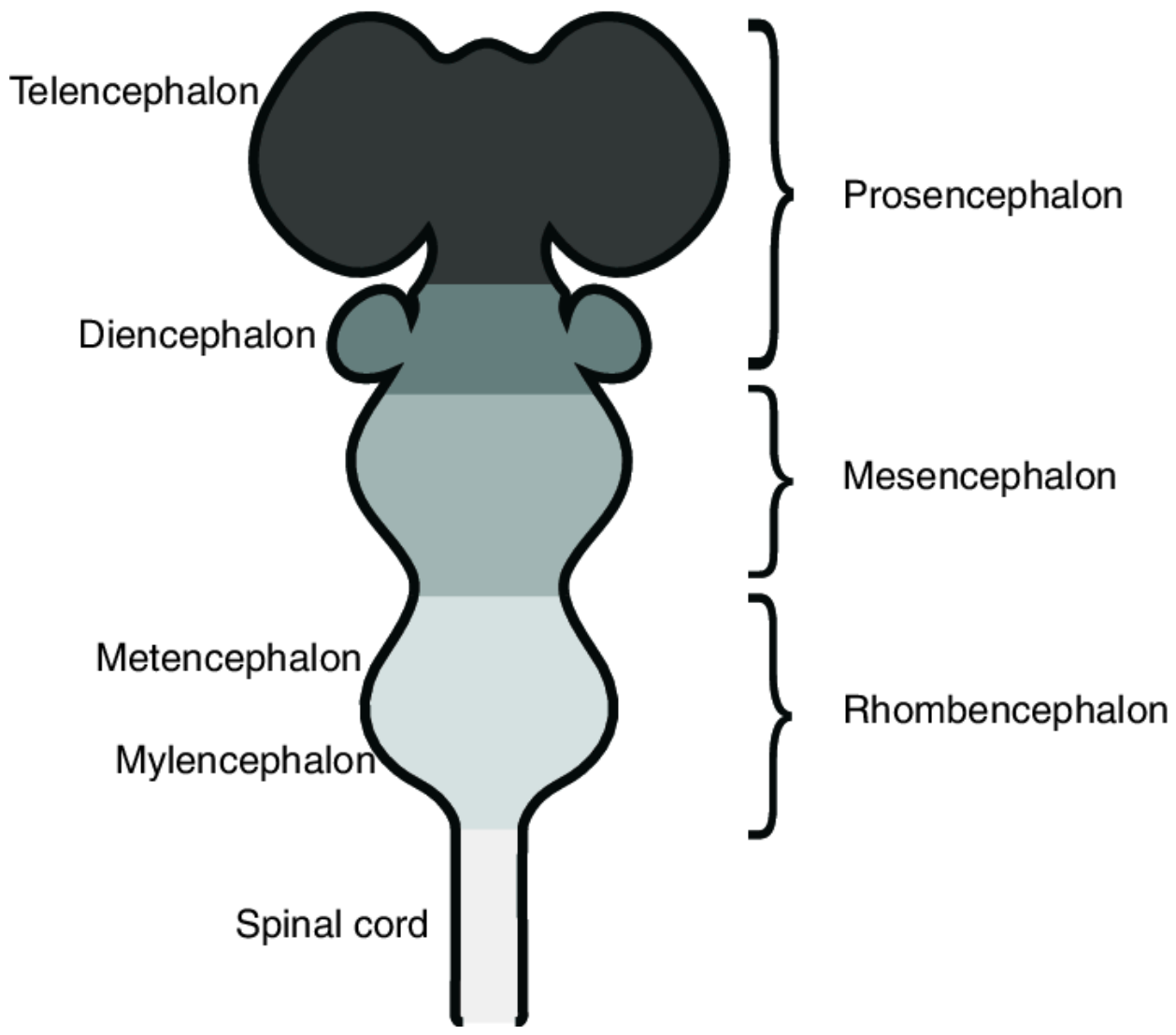

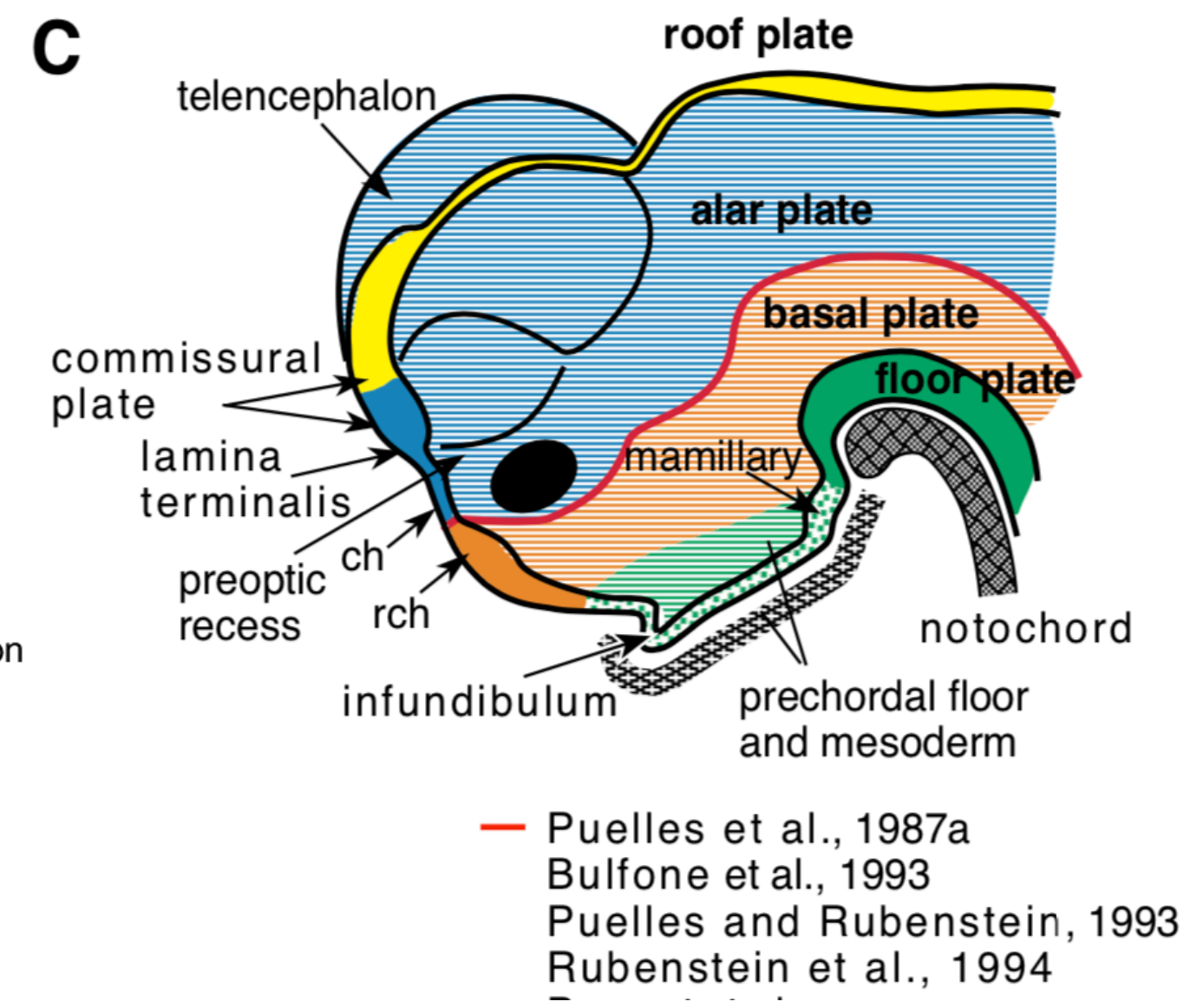

zones: **floorplate**, **basalplate**, **alarplate** and **roofplate** (PMID: 8575293).

Neural crest cells: The neural crest cells (NC cells) arise from the apices of the elevating neural folds along the neuroectodermal/surface ectodermal junction. These cells delaminate from the neuroepithelium, undergo EMT, and migrate in streaming columns to colonize throughout the anterior-posterior body axis to generate numerous derivatives. NC cells originate from four major segments of the neural tube to form **cranial (or cephalic**), **vagal, trunk,** and **sacral neural crest** cells. Distinct populations of cranial NC cells originate from the diencephalon, midbrain, and hindbrain and have the capability to differentiate into craniofacial bone, cartilage, and connective tissue. The vegal NC cells form various cell types in thymus, thyroid, parathyroid and heart, and also forms ganglia in lung, pancreas, and the gut. Cardiac NC cells (a subset of Vegal NC cells) from the dorsal neural tube between the otic vesicle and the third somite. These cells contribute to conotruncal cushions and the aorticopulmonary septum during overt septation of the outflow tract and envelop both the thymus and thyroid as these organs form. Trunk NC cells arise in the posterior part of the embryo and migrate along three distinct pathways: a dorsal-lateral pathway between the ectoderm and the somites, a ventral-lateral pathway between somites, and a ventral-medial pathway between the neural tube and the posterior sclerotome. These cells generate melanocytes, thatch he peripheral nervous system, and secretory cells of the endocrine system. The sacral neural crest contributes neurons and glia to the post-umbilical gut.

Neuralmesodermal progenitors (NMPs): NMPs are bipotent cells that can contribute to both the elongating spinal cord and the adjacent paraxial mesoderm. Fate-mapping studies have localized NMP cell population to the **caudal lateral epiblast (CLE)** and adjacent **node-streak border (NSB)**.

————————NMP enriched——————————————————-
T, Sox2, Nkx1-2: from the definition and source of NMP
Fgf8, Wnt3a: from the signaling requirements of NMP
Epha1:[PMID: 35299237,scRNA/insitu] this paper also suggest: **Wnt5a, Cdx4, Fgf17, Cdx2, Hoxb11, Gdf11, Sp5, Evx1, Efna1, Spry4, Gad1**.
Sp5: [PMID: 11231070, insitu] E7.25-E7.5 along PS; E8.0-E8.5 posterior end of neural plate and the underlying mesoderm(tailbud region). Faint in mesencephalon and diencephalon, gradually become strong. With the formation of the gut, the definitive endoderm also becomes positive, as well as notochord.
Nkx1-2: [https://doi.org/10.1101/045872, PMID: 7669696, insitu] E6.0 no expression. E7-E7.5 PS near node. ~E8.5 posterior forming neuroepithelium, anteriorly ending at the level of the last

formed somite. ~E10.5 during secondary neurulation, it is expressed in the dorsal part of the tail bud, extending into the neural tube.
Fgf8: [PMID: 23874217, insitu] E8.0-E8.5 tailbud region.
Hoxa7, Hoxb4, Hoxb3, Hoxb1: [PMID: 2571936, insitu] E9.5 enriched in tailbud region.

Low marker: **Tbx6**(tailbud mesoderm/presomitic mesoderm marker)

——Neuroepithelium (posterior and anterior)—————————————-
Sox2, Sox3: [PMID: 10446282, insitu] E8.0-E9.0 Sox1, Sox2 and Sox3 are expressed in the neuroectoderm. At 6 somites stage, Sox2 and Sox3 transcripts are extended along the A-P, while Sox1 is more anterior.
Gemin2(Sip1): [PMID: 16127714, insitu] E8.5 whole neural plate and paraxial mesoderm.

————————-**NeuralEpithelium_posterior enriched**——————————————
Nkx1-2: [https://doi.org/10.1101/045872, PMID: 7669696, insitu] E6.0 no expression. E7-E7.5 PS near node. ~E8.5 posterior forming neuroepithelium, anteriorly ending at the level of the last formed somite. ~E10.5 during secondary neurulation, it is expressed in the dorsal part of the tail bud, extending into the neural tube.
Gbx2: [PMID: 12799077, insitu] E8.25 expression was detected within the PS and throughout the neural tube with a rostral border and mid/hindbrain junction. E9.0 enriched in tailbud/posterior region. While anterior expression is gradually fainted, only persist in midbrain-hindbrain junction and the level of branchial arch.

Low marker: **Fgf8** [PMID: 23874217, insitu]

———————-NeuralEpithelium_anterior enriched————————————————————
Pax6:[PMID: 23874217, insitu] E8.0-E8.5, Anterior neuroplate (except tailbud region), forebrain. Pou3f2
Sox1: [PMID: 10446282, insitu] E8.0-E9.0 Sox1, Sox2 and Sox3 are expressed in the neuroectoderm. At 6 somites stage, Sox2 and Sox3 transcripts are extended along the A-P, while Sox1 is more anterior.
Irx3: [PMID: 9427753] Irx3 in the mouse are expressed early in the prospective neural plate in a subset of neural precursor cells. E7.5 with *Irx3* expression in the embryonic ectoderm (eme) and extra-embryonic ectoderm (exe). E8, *Irx3* expression is seen in the posterior head fold (hf) portion giving rise to the hindbrain. E8.5 embryo showing *Irx3* transcripts concentrated to the ventricular zone of the developing spinal cord. 8.5 embyo. *Irx3* expression is confined to the midbrain (MB), hindbrain (HB) and the rostral part of the spinal cord (SP). cephalic mesodermal cells (cem). NO heart expression.

———Forebrain enriched————————————————
Otx2: [PMID: 7588062, Otx2-lacZ/insitu] E9.0-10.5 cover anterior most forebrain and midbrain region.
Hesx1:[PMID: 8565852, insitu] ~E8 expression is restricted to the cephalic neuroectoderm.
Foxg1(BF1,forebrain-restricted transcription factor): [] At E8.5, foxg1 expression is present in the most rostral region of neural tube. E9, Foxg1 expression is contained in the telencephalic neuroepithelium, including the progenitor cells of the cerebral cortex, the basal ganglia and the olfactory bulb.
Six3: [PMID: 22186731, insitu] forebrain.
Nkx2-1: [PMID: 8575293, review/in situ] Nkx2-1 is first detected at about 1-somite stage in a median strip of the anterior head and ends caudally near the cephalic flexure. At the beginning of anterior neuropore closure, two discontinuous domains (ventral and dorsal) of Nkx2-1 expression are apparent. Ventral one covers the postoptic hypothalamic area; the dorsal domain of Nkx2-1 is found in the rostroventral telencephalon and includes the preoptic area, medial ganglionic eminence and anterior entopeduncular area.

———Midbrain enriched————————————————
En1, En2: [PMID: 17537797, lacZ] E8.5-E11.5 midbrain.

En2, Wnt1, Pax5, Pax2: [PMID: 9405645, insitu]

———————-Hindbrain enriched—————————
Irx3: [PMID: 9427753] Irx3 in the mouse are expressed early in the prospective neural plate in a subset of neural precursor cells. E7.5 with *Irx3* expression in the embryonic ectoderm (eme) and extra-embryonic ectoderm (exe). E8, *Irx3* expression is seen in the posterior head fold (hf) portion giving rise to the hindbrain. E8.5 embryo showing *Irx3* transcripts concentrated to the ventricular zone of the developing spinal cord. 8.5 embyo. *Irx3* expression is confined to the midbrain (MB), hindbrain (HB) and the rostral part of the spinal cord (SP). cephalic mesodermal cells (cem). NO heart expression.
Egr2(Krox20), Mafb(Kreisier): [PMID: 21930923, insitu] E8.5 Rhombomere 3 and 5.
Egr2, Cyp26b1, Tmem35a: [PMID: 19208226,insitu] E9.5 Rhombomere 3 and 5.

———————-Neural crest cells enriched—————————
Cdh6, Cdh11, Itga4, Itga5,Itgb1, Adam12, Mmp2: [PMID: 23123967, review] Cranial NC markers.
Sox10, Dlx2, Foxd3, Tfap2a, Pax3, Sox9: [PMID: 30787436, scRNAseq]
Pax3: [PMID: 23397132, insitu] posterior-lateral domain of forebrain region that give rise to neural crest cells in an older embryo.

———————-Floor&Basal_plate enriched—————————
Nkx2-2, Shh: [PMID: 8575293, review/in situ] express in different domain of floorplate (here include ventral midline cells(real floor plate) and adjacent paramedian plate cells).
Foxa1,Foxa2: [PMID: 8069910, insitu] E9.5 notochord and floorplate.
Ntn1(Netrin1): [PMID: 28445456, insitu/IF] E9.5 expressed in basal plate ventricular zone and floor plate.
Bmp7,Foxa2, Shh, Nkx2-1: [PMID: 9244300,IF]
Nkx6-1: [PMID: 11567614,IF] E8.5 basal plate.

Negative marker: **Nkx6-2, Dbx2, Pax7, Pax6** (dorsal neural tube marker) [PMID: 11567614, IF]

———————-Neurons enriched—————————
Pou3f1(Oct6),Pou3f2(Brn2):[PMID: 12198163, insitu] embryonic cortex development
Isl1: [PMID: 8069910, 8565076, 21569850, insitu] E9.5 express in the nuclei of developing motor neurons in the ventral spinal cord adjacent to the floorplate.
Gadd45g: [PMID: 21843656, insitu] primary neuron precursors.
Uchl1: [PMID: 26222784, Uchl1-GFP] sensory neuron marker.

⑤ <u>NonNeural_Ecto/General_Ecto</u>

Description:

The folding neural plate is bordered by non-neural ectoderm cells, which include **surface ectoderm (epidermis)** and later specified **preplacodal ectoderm.** The preplacodal ectoderm is divided into **posterior preplacodal ectoderm (E8.0 otic and epibranchial placodes)** and **anterior preplacodal ectoderm (E8.0 lens, E8.5 olfactory and adenohypophyseal placode)**. In our annotation, I did not found a proper cluster for preplacodal ectoderm, which may be due to the small cell population size or transient emergence.

Important biological processes:

Cranial placodes specification: Develop exclusively from cranial non-neural ectoderm, placodes give rise to various sensory ganglia and contributes to the pituitary gland and sensory organs of the vertebrate head. They include the adenohypophyseal, olfactory, lens, trigeminal, and profundal placodes, a series of epibranchial placodes, and an otic placode. All placodes originate from a common **preplacodal primordium** around the neural plate. Placodes are regions of increased cell proliferation compared to the epidermis and development of placodal derivatives often involves cell shape changes (columnar epithelia) and morphogenic movements (invagination). All placode except adenohypophyseal and lens placode are neurogenic.

Anterior cranial placodes include the **adenohypophyseal, olfactory and lens placodes**. The adenohypophyseal placode invaginates from the roof of the mouth to form Rathke's pouch which differentiates into the anterior pituitary and give rise to five types of endocrine hormone-secreting cells. The olfactory placode invaginates to form the olfactory sensory epithelium containing various types of secretory cells and olfactory sensory neurons, while the lens placode invaginates to give rise to the lens vesicle.

The posterior placodes comprise the **otic** and **epibranchial placodes** that give rise respectively to the inner ear and sensory neurons of the geniculate, petrosal and nodose ganglia. The otic placode invaginates and pinches off from surface ectoderm to form the otic vesicle which then differentiates to generate the cochlear and vestibular systems of the inner ear, and the sensory neurons of its associated vestibulocochlear (VIIIth) ganglion.

Finally, the **trigeminal placode** develops between the anterior and posterior placodes, giving rise to the sensory neurons of the ophthalmic and maxilla-mandibular divisions of the trigeminal ganglion.

Surface ectoderm/Periderm specification: After neural tube closure, the **surface ectoderm** subsequently differentiates into **epidermis**. It is also predicted that neural ectoderm and surface ectoderm must be coordinated for closure to succeed. Surface ectoderm generates a transient protective layer called **periderm**, which is shed off once the epidermis has differentiated and stratified. Before epidermis stratification, around E9.5 in mouse embryo, Krt5/Krt14 express replace Krt8/Krt18 expression, which marks the event called **epidermal commitment**. Then surface ectoderm becomes the embryonic epidermal basal layer that gives rise to all the structures of the future epidermis and epidermal appendages.

All states: Posterior_Epi, Surface_ecto, Epidermis, Placode_anterior, Placode_posterior, Placode_trigeminal

**E7.0**	Proximal_Epi
**E7.5**	Proximal_Epi
**E8.0**	Surface_ecto, Placode_posterior, Placode_anterior, Epidermis
**E8.5**	Surface_ecto, Placode_posterior, Placode_anterior, Placode_trigeminal, Epidermis

Expression pattern:

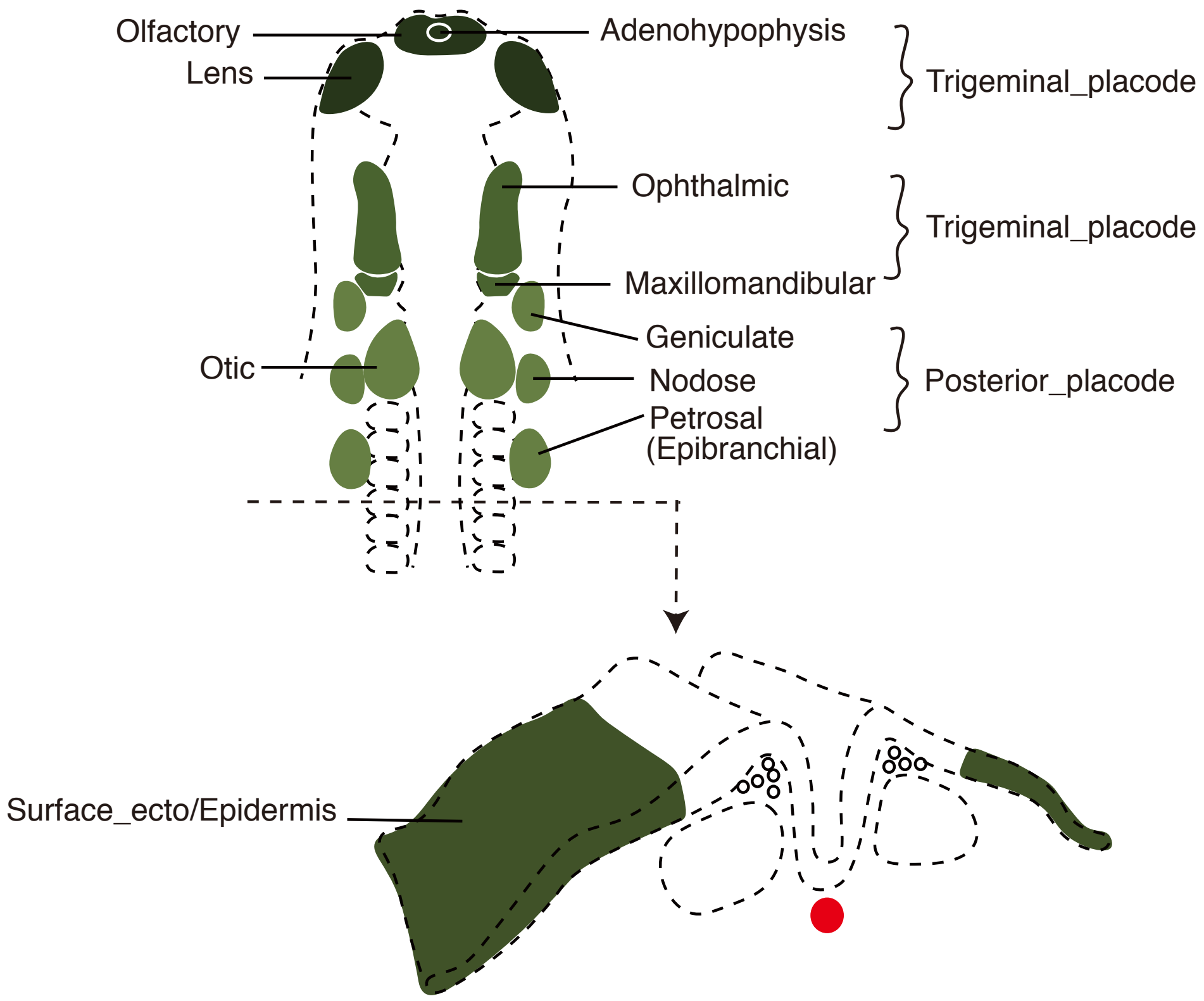

——-Placode_anterior———————————-
Otx2:[PMID: 15790971, insitu] E9.5 forebrain and lens placode.
Pax6, Sox2, Pitx2: [PMID: 23426374, insitu] E9.5-E10.5 lens placode.
Sox2: [PMID: 9609835, insitu] Sox2 is not expressed in the early preplacodal region; it is expressed in the distinct lens, olfactory and otic placode
Six1, Six4: [PMID: 19027001, insitu] E8.0-E8.5 express in preplacodal region. E9.0 -14.5 olfactory ectoderm.
Eya1, Eya2: [PMID: 19027001, lacZ] E8.25-8.5, Eya1 is not expressed in preplacodal region. Its expression became detectable at around E8.75. E10.5 in the center of olfactory pit; E8.0-E8.5 Eya2 is expressed in preplacodal ectoderm and in olfactory ectoderm at E9.0.
Dlx5, Otx1, Ebf1, Lhx2: [PMID: 19027001, insitu] E9.5-E10.5 olfactory placode.

——-Placode_posterior———————————-
Gbx2:[PMID: 15790971,insitu] E9.5 otic placode.
Pax2, Pax8, Jag1: [PMID: 20727173, insitu] E8.0 otic placode.

——Placode_trigeminal———————————-
Pax3, Sostdc1(wise): [PMID: 18538759, insitu] trigeminal region.

Negative marker: **Pax6**

———Surface_ecto enriched—————————————
Krt8, Krt18, Tfap2a: [PMID: 36007540, scRNA]
Ckap4(p63): [PMID: 26690418, IF/insitu] First appears near the newly formed somites and the posterior part of the embryo (E8.5), further expanding to the whole embryonic surface with particular enrichment in the first branchial arches and the limb buds and tailbud. (E9.5-E10.5).
Dkk1, Krt19, Amot :[PMID: 30787436, scRNAseq]

Krt19:[PMID: 31393940, lacZ] E9.5 skin.

Highest BMP activity: **Msx1, Msx2, id1, id2**

——Epidermis/Periderm enriched—————————-

Krt5:[PMID: 7525178, lacZ/insitu] E9.5 Dorsolateral ectoderm over posterior somites. By E10.5, expression was intense in the first branchial arch.

Krt19:[PMID: 31393940, lacZ] E9.5 skin.

Krt17,Irf6,Sfn, Fut4(SSEA1):[PMID: 25133425, insitu] Periderm markers.

*__Epicardium (not exist in our annotation)__: **Wt1, Tcf21, Sema3d, Tbx18, Scx** [PMID: 32359445, scRNA]

⑥ **Meso**

Description:

From E6.25 in mice, mesoderm continuously emerges from a transient and dynamic zone known as the primitive streak (PS), which forms in the posterior epiblast. During EMT, cells lose pluripotency and acquire distinct mesodermal lineage identities while spreading over the embryo between the visceral endoderm and epiblast. The first progenitors are specified at the posterior PS and generate extra-embryonic structures such as the amnion, allantois, and yolk sac. More anterior **PS** generate **cardiac and cranial** lineages, followed by **intermediate, lateral and paraxial mesoderm**. The lateral plate further subdivides into **somatopleure** and **splanchnopleure** by a split in the mesoderm, creating the coelom or body cavity. After this initial induction, progenitors differentiate and migrate towards their final destination while new progenitors continue to emerge.

Paraxial mesoderm (PM) development is composed of several stages: **presomatic mesoderm specification, somitogenesis** and **somite specification.** Mature somites contain two major populations: the **sclerotome** and **dermomyotome**, the space between is coelom, or body cavity. The sclerotome gives rise to the **vertebrae and associated ribs**, **tendons**, and other tissues, such as **vascular cells of the dorsal aorta**, **intervertebral blood vessels**, and **meninges**. The **dermomyotome** produces two components: the **myotome** and the **dermatome**. The myotome gives rise to the **musculature of the back, rib cage, ventral body wall**, and **limbs**. The dermatome gives rise to the **dermis of the back**.

The **lateral mesoderm (LPM)** forms the **splanchnic mesoderm** and **somatic mesoderm.** The splanchnic mesoderm gives rise to components of the circulatory system, such as the **heart**, **blood vessels**, and **blood cells,** whereas the somatic mesoderm forms the **pelvic skeleton** and **mesodermal components of the limbs**, with the exception of the muscles, which are derived from the dermomyotome.

The **intermediate mesoderm (IM)** forms the **urogenital system**, including the **kidneys** and **gonads.**

Important biological process:

Heart Development: The first wave of cardiac mesoderm are generated by **precardic mesoderm** cells migrating to the anterior lateral region of the embryo (**first heart field, FHF**), which forms **cardiac crescent**. At E8.0, Fusion of cardiac crescent at midline at the time of foregut invagination generates a **heart tube** with a caudal venous pole and rostral arterial pole. Cells of **second heart field (SHF)** are specified from **pharyngeal mesoderm** and added to the developing heart tube at E9.0. FHF mainly contributes to the **left ventricle of the heart**, while the SHF give rise to **right ventricle** and **outflow tract (OFT)**. An additional, non-mesodermal cell population migrates from the neural crest into the heart via the pharyngeal arches and the OFT. These are named cardiac neural crest cells, and participate mainly to the septation of the heart and formation of the cardiac valves.

Craniopharyngeal development: In head region, the unsegmented **cranial paraxial mesoderm (Head_paraxial_meso)** contributes to broad regions of the heart and head/neck musculature that surrounds the developing pharynx and is situated within the core of the pharyngeal arches. The pharyngeal mesoderm can be divided into two subdomains: the loosely connected, **mesenchymal paraxial mesoderm (Dorsomedial_cranial_PM)**, located on both sides of the neural tube and notochord; and the **medial splanchnic mesoderm** which is maintained as epithelial tissue, containing SHF cells, connecting to FHF cells. This lateral splanchnic mesoderm is located on the ventral side, beneath the floor of the pharynx.

Somitogenesis: Paraxial_meso is segmented into sequentially ordered blocks of cells referred to as somites. On formation, the paraxial mesoderm has a mesenchymal appearance and is called **presomitic mesoderm (PSM)**. Presomitic mesoderm is derived from the primitive streak or neuromesodermal progenitors in the tail bud. After maturation, the PSM becomes segmented,

resulting in two rows of epithelial spheres, the somites, along side the neural tube. Later in development, the somites undergo further differentiation along the dorsal–ventral axis into **dermomyotome** and **sklerotome**, which give rise to dermis and skeletal muscles, and to the axial skeleton, respectively.

Kidney development: the kidney develops in a distinct temporal sequence via three different stages: **pronephros** (E8.0), **mesonephros** (E9.5) and **metanephros** (E10.5-adult kidney). The kidney primordia emerge as bilateral fields early somitogenesis. In amniotes, kidney progenitors form directly adjacent to the paraxial somites as the most medially located LPM stripe (also referred to as **intermediate mesoderm**).

Limb formation: Limb emerge from the trunk at E8.5, as bilateral buds of mesenchymal cells from the LPM covered by an ectodermal epithelium.

All states: PS_mid, PS_late, Nascent_Paraxial_meso, Nascent_LateralPlate_meso, Nascent_Intermediate_meso, Head_paraxial_meso, Precardiac_meso, Tailbud_meso, Somites, Somites_sclerotome, Somites_dermomyotome, SHF, FHF, LateralPlate_meso, LPM_hindlimb, LPM_forelimb, Intermediate_meso, IM_Pronephros, CranialPharyngeal_meso

**E7.0**	PS_mid, PS_late, Nascent_Paraxial_meso, Nascent_LateralPlate_meso, Head_paraxial_meso, Precardiac_meso
**E7.5**	PS_late, Nascent_Paraxial_meso, Nascent_LateralPlate_meso, Nascent_Intermediate_meso, Head_paraxial_meso, Precardiac_meso
**E8.0**	Tailbud_meso, Somites, SHF, FHF, LateralPlate_meso, Intermediate_meso, IM_Pronephros, CranialPharyngeal_meso
**E8.5**	Tailbud_meso, Somites, Somites_sclerotome, Somites_dermomyotome, SHF, FHF, LPM_hindlimb, LPM_forelimb, LateralPlate_meso, IM_Pronephros, CranialPharyngeal_meso

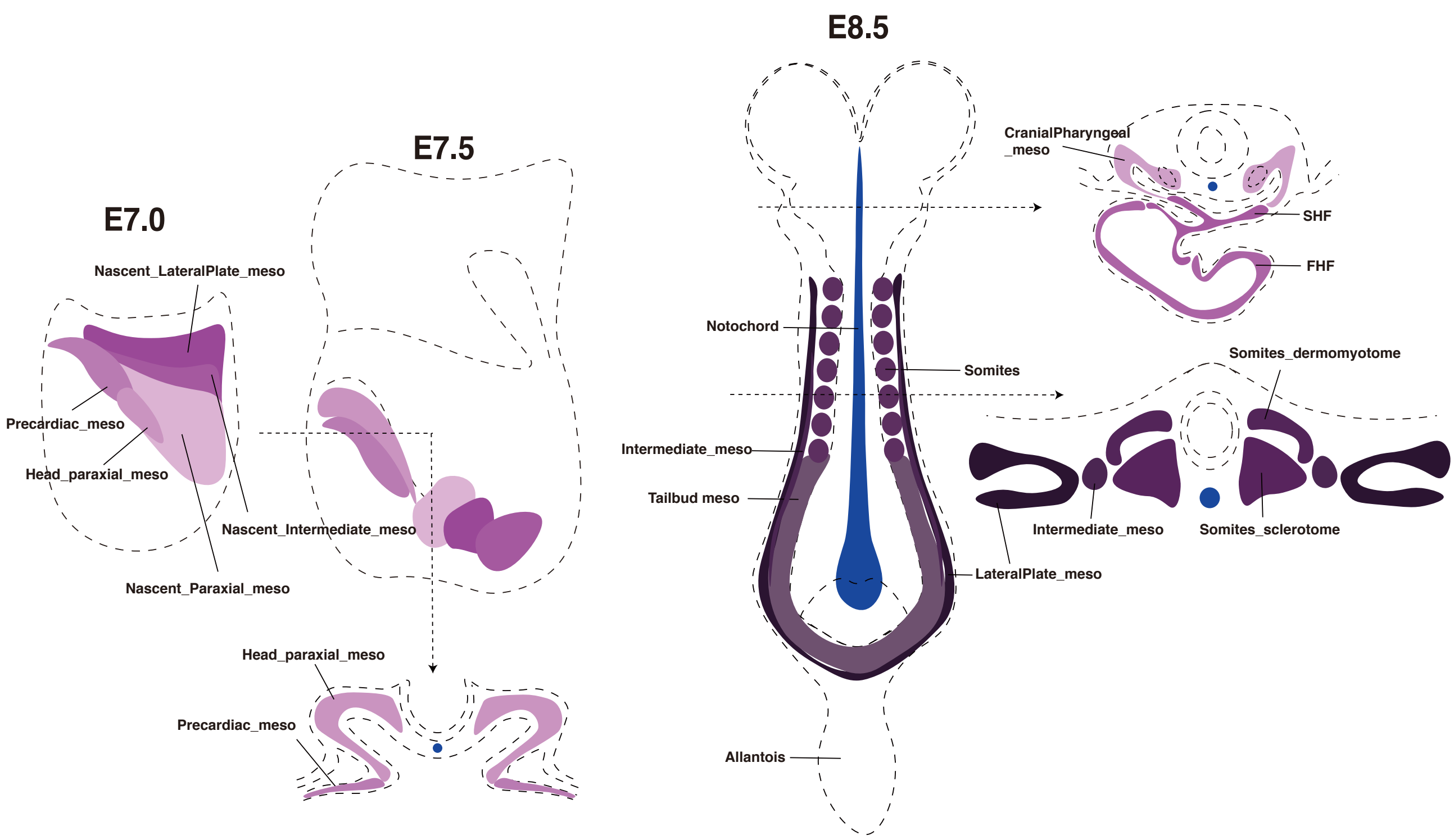

Expression pattern:

———————-Nascent_paraxial_meso cells enriched———————————

Mesp1:[PMID: 8787751, insitu] E6.5-E6.75 Mesoderm wing. E7.5-E8.0 Base of allantois, LPM.
Snai1:[PMID: 9671584, insitu] E7.5 PS and migrating mesoderm cells. E8.5 Snail1/2 express in neural crest cells. E9.5 Snai1 is in tailbud. Snai2 is in IM.
Lefty2, Tdgf1(Cripto): [PMID: 14668413, insitu] E7.0 posterior view show that Lefty2 and Tdgf1 is in the mesoderm wings.
Eomes: [PMID: 10716450, insitu] subset of nascent mesoderm.
Lhx1: [PMID: 10328927, insitu] E7.0 Expression in mesodermal wings.
Fgfr1:[PMID: 1321062, insitu] E6.5-E8.5 Initially in primitive ectoderm and then in migrating mesoderm and embryonic ectoderm; later in neural ectoderm and paraxial mesoderm.
Ripply2: [PMID: 17937396, insitu] E7.75 Expression is as a single bilateral stripe located anterior and perpendicular to the node. E8.5 Expression is restricted to the anterior presomitic mesoderm (newly formed somites.)
Lfng: [PMID: 12441287, insitu] E7.0 express in the posterior mesoderm. E7.5 Expression in a distinct domain lateral to the node.

Low marker: **T**
Negative marker: **Cdh1**

——————————-Nascent_Intermediate_meso——————————————————-

Osr1: [PMID: 16223478, lacZ] E7.5 Expression in intermediate mesoderm at E7.5, shortly after they migrate away form primitive streak. E8.5 Expression throughout intermediate mesoderm caudal to the cardiac crescent. E9.5 Strong expression in dorsal wall of common atrial chamber, caudal somites.
Lhx1: [PMID: 10864462, insitu] Late streak stage, show some expression in early intermediate mesoderm.
Cfc1(Cripto, Cryptic):[PMID: 9053319, insitu] E6.5 Patchy expression in the mesodermal wings and localized expression at the anterior end of the primitive streak E7.5 Expression is found in the head process (arrow) and medial region of the egg cylinder. The staining in the head process is more intense at its caudal end, where the node is forming. E8.0-E8.5 LPM and notochord plate.

High BMP targets: **Id1, Id2, Msx1, Msx2**

——————————-Nascent_LateralPlate_meso——————————————————-

Hand1[PMID: 9500550, lacZ] E7.75 cardiac crescent and lateral mesoderm.
Foxf1[PMID: 14745957, insitu]: E8.0-E8.5 posterior primitive streak and LPM, restricted to splanchnic mesoderm.
Cfc1(Cripto, Cryptic):[PMID: 9053319, insitu] E6.5 Patchy expression in the mesodermal wings and localized expression at the anterior end of the primitive streak E7.5 Expression is found in the head process (arrow) and medial region of the egg cylinder. The staining in the head process is more intense at its caudal end, where the node is forming. E8.0-E8.5 LPM and notochord plate.

High BMP targets: **Id1, Id2, Msx1, Msx2**

——————————-Precardiac_meso——————————————————————-

Tbx5, Nkx2-5:[PMID: 26469858, insitu] E7.5-E8.5 anterior embryo, cardiac crescent.
Smarcd3, Mef2c, Isl1, Tbx5: [PMID: 25296024, insitu] E7.0-E7.5 Smarcd3 expression precedes Isl1 and Tbx5 in a domain that lies at the anterior-proximal region of the embryo and extends into the extraembryonic tissues. E8.5 Cardiac crescent and looping heart.
Isl1: [PMID: 14667410, insitu] E7.0-8.0 pharyngeal meso (mainly in anterior LPM splanchnic meschenchyme, include SHF) and overlap with FHF. E8.5 outflow tract (SHF).
Tbx20:[PMID: 10940636, insitu] E7.5 both allantois and amnion, LPM and cardiac crescent. E7.75 cardiac crescent and allantois. E8.0 developing heart tube. E8.5-E9.5 looping heart tube, LM, optic vesicle and neural tube.
Sfrp5:[PMID: 28287088, IF] E7.5, cardiac crescent but different from Nkx2-5.
Hand2: [PMID: 9500550, insitu] E7.75 cardiac crescent

Hand2 expression marks the cardiac crescent and liner heart tube and is downregulated within the left ventricle at the onset of cardiac looping. At comparable developmental stages, the left ventricle and myocardial cuff express Hand1.
Gata4, Gata5: [PMID: 8045339, 9119112, insitu] E7.0 Expression exclusively in embryonic mesoderm subjacent to future headfold region which contains the cardiogenic plate.
Bmp2:[PMID: 7657163, insitu] E7.5 Bmp-2 RNA is localized in the anterior neural folds and presumed precardiac mesoderm.

————————————Head_paraxial_meso enriched———————————————
Tcf15 (paraxis/meso1): [PMID: 7597044, insitu] E7.5-E7.75 bilaterally head paraxial mesoderm under the neural fold of hindbrain regain. E8.25 first several somites and show a sharp anterior boundary of expression in the unsegmented paraxial mesoderm of the head. E8.75-E9.0 somites.
Tbx1: [PMID: 19247433, 8853987, insitu] E8.0-E8.5 pharyngeal region. E7.5 Expression is present in the distal, anterior region of the egg cylinder, rostral to the node.
Twist1: [PMID: 8573722, insitu] E7.0 Expression is lateral to the primitive streak. Gradually enriched in anterior part. E8.0 Expression is in the primordium of the thoracic body wall at the level of the foregut and heart. Expression is also in somites (sclerotome) and in the forming somite. E8.5 first branchial arch.
Nbl1(Dan):[PMID: 10208746, insitu] E7.5 Expression is apparent in the mesodermal wings that underlie the anterior neural plate. E8.0 Expression was seen in the paraxial mesoderm both within and rostral to the somitic column. E8.5 strongest throughout the two most caudal somites but appeared to be rapidly downregulated as these somites matured.

———————————Tailbud_meso/presomitic meso enriched————————————-
Fgf8: [PMID: 15196959,7768185, insitu] E8.5 Taibud after somites. Fgf8 RNA is detected at a uniformly high level throughout the primitive streak(unsegmented region), with a sharp anterior boundary at the node. RNA is also detected in cells located in the wall of the intraembryonic coelom
Fgf3(int2): [PMID: 3293998,14623822, insitu]E7.5 newly emerged mesoderm. E8.0 newly emerged mesoderm and XMeso. E8.5-9.5 tailbud, hindbrain, PE. E9.5 pharyngeal pouch.
Cyp26a1:
Hes7,Notch1: [PMID: 18708576, insitu] E9.0 Notch1 is expressed in both Tailbud and latest somites. Notch2 only in latest somites.
Tbx6, Dll1: [PMID: 18708576, insitu]
Tbx6:[PMID: 8954725, insitu] Tbx6 transcripts are not detected at E6.5, but are first detected attached E7.0, in a stripe which corresponds to the early primitive streak. At E7.5, Tbx6 expression in PS extends laterally to paraxial mesoderm. At E8.5-12.5, Tbx6 is detected in the unsegmented, presomatic mesoderm of the tail region, surrounding the caudal end of the neural plate.
Dll1: [PMID: 7671806] E7.5 PS and some nascent mesoderm. E8.0 posterior mesoderm extend to around the node. E8.5 “U" shape presomitic mesoderm.
Cdx4: [PMID: 19539783, insitu] E8.0-8.5 Expression in the caudal region was extended anteriorly in the lateral plate mesoderm.

Low/negative marker: **Sox2**, BMP targets(**Id1, Id2, Msx1, Msx2**)

———————————Somites enriched—————————————————————-
Tcf15 (paraxis/meso1): [PMID: 7597044, insitu] E7.5-E7.75 bilaterally head paraxial mesoderm under the neural fold of hindbrain regain. E8.25 first several somites and show a sharp anterior boundary of expression in the unsegmented paraxial mesoderm of the head. E8.75-E9.0 expression is mantained more posterior somites.
Pax3: [PMID: 33214242, insitu] E8.5 Anterior neural folds and somites.
Meox1, Fst: [PMID: 11562355, insitu] E8.5 Meox1 is normally strongly expressed in somites, including the forming somite.
Meox1: [PMID: 15196959,insitu] E8.5 all somites, low in PSM.

Meox2: [PMID: 1363541, insitu] E8.5 Expression is present in the entire somite. No expression in neural tissue, presomitic mesoderm and cardiac mesoderm. E9.5 enrich in trunk sclerotome.
Foxc1, Foxc2: [PMID: 8375339, insitu] E6.5 PS and nascent mesoderm. E7.5-E8.5 Expression is detected from the anterior paraxial mesoderm to the middle of the primitive streak.
Aldh1a2(Raldh2):[PMID: 21385575, insitu] E7.25-E7.5 expansion of expression in nascent mesoderm and PS. E8.5 somites.
Tbx18,Uncx(Uncx4.1):[PMID: 18708576, insitu] E8.5-E9.0 somites.

——————————Somites_Sclerotome enriched———————————————————-
Pax1, Pax9, Sox9: [PMID: 17557304, review]

——————————-Somites_Dermomyotome_ enriched————————————————-
Myf5: [PMID: 15196959, insitu] E8.75 Strongest expression in medial lip of differentiating dermomyotome.
Dmrt2, Pax3, Myf5: [PMID: 20368965, insitu]
Pax3: [PMID: 10545229, insitu] E9.0 Expression is detected in the dorsal somitic region that gives rise to the dermomyotome.

——————————-FHF(cardiac_crescent/heart tube)——————————————————
Nkx2-5: [PMID: 24613616, insitu] E7.5-E8.0 cardiac crescent, Pharyngeal mesoderm(SHF), FHF. E8.5-E9.0 whole heart.
Tbx5, Irx4: [PMID: 24123507, IF] E8.75 Irx4 expression was detected in the majority of the cells of the cardiac crescent. A subset of these cells in the anterior and lateral regions were also positive for Tbx5.
Myl7:[PMID: 14667410, insitu] E7.0-8.0 FHF(ventral most cardiac crescent). E8.5 heart tube
Hcn4:[PMID: 14643687, insitu] E7.5 Expression is in the cardiac mesoderm. E8.0 sinus venosus. E8.5 left atrium.
Mef2c: [PMID: 15937931, insitu] E8.5 Expression is in the cardiac mesoderm.
Tbx20: [PMID: 10940636, insitu] E7.5 heart precursor and allantois. E7.75 cardiac crescent, allantois. E8.0-E10.5 Myocardium of looping heart tube. LPM immediately below the heart.
Myl1: [PMID: 18973680, insitu] E7.75 heart crescent.
Acta1: [PMID: 18400219, IHC] E8.5 Expression in nascent cardiac myocytes.

——————————-**SHF**——————————————————
Isl1: [PMID: 14667410, insitu] E7.0-8.0 pharyngeal meso (mainly in anterior LPM splanchnic meschenchyme, include SHF) and overlap with FHF E8.5 outflow tract(SHF).
Fgf10, Fgf8: [PMID: 11702954, insitu] E8.0 SHF dorsal to the heart tube. E9.5 Right ventricle and OFT.
Tbx1: [PMID: 14975726, insitu] E7.5 -E8.5 CPM and SHF.

——————————————-CranialPharyngeal_meso enriched————————————————-
Six1, Eya1, Tbx1:[PMID: 21364285, insitu] E8.5, CPM, somitic mesoderm (Six1, Eya1)
Six1: [PMID: 20471971, insitu] E8.0 CPM, pharnygeal endoderm.
Snai2: [PMID: 9671584, insitu]

——————————-LPM enriched———————————————-
Foxf1, Bmp4, Hoxb6, Hand1, Hand2, Prrx1: [PMID: 32561665, review]
Foxf1: [PMID: 11124112,14745957,9486531, 15196959, insitu] E8.5 allantois, bilateral stripe of LPM. In both splanchnic and somatic mesoderm at the hindgut level, but restricted to the splanchnic mesoderm at midgut and foregut levels. E9.5 Splanchnic mesoderm. vasculature of E9.5 yolk sac.
Cdx4: [PMID: 19539783, insitu] E8.0-8.5 Expression in the caudal region was extended anteriorly in the lateral plate mesoderm.
——————————-LPM_forelimb enriched——————————————-
Tbx5, Hoxc4, Hoxc5: [PMID: 15621531, insitu]
——————————-LPM_hindlimb enriched——————————————-

Tbx4, Pitx1, Hoxc10: [PMID: 15621531, insitu]
Cdx4: [PMID: 19539783, insitu] E8.0-8.5 Expression in the caudal region was extended anteriorly in the lateral plate mesoderm.
Hoxd9: [PMID: 22819676, insitu] E10.5 tail and hind limb.

——————————————**IM enriched**———————————————-
Osr1,Lhx1: [PMID: 15196959, insitu]~E8.5 Osr1 is expressed in bilateral stripe outside the somites, extended from tailbud. Lhx1 is also expressed in bilateral stripe, but not extend to tailbud.
Osr1: [PMID: 16223478, insitu/lacZ] E8.5 throughout the IM caudal to the cardiac crescent and is activated in many other tissues, including the branchial arches and limb buds in later stages. E7.5 intermediate region of mesoderm wings, no staining detected in PS. E9.5 IM and lung bud mesenchyme, fore- and hindgut mesoderm and in the dorsal atrial wall.
Grem1(Drm): [PMID: 10208746, insitu] E8.5 intermediate mesoderm and Pronephros.

—————————**IM_Pronephros_meso**——————————————————————
Pax2, Pax8, Ret(c-ret): [PMID: 12435636, insitu] E8.0-8.5 pronephric anlage (before MET), midbrain-hindbrain boundary, otic placode.
Lhx1, Wt1: [PMID: 12435636, IF] E9.5 Genital ridge and pronephros.
Gdnf: [PMID: 16018995, insitu] E8.5-11.5 nephrogenic mesenchyme.
Pax2, Pax3, Gata3: [PMID: 10631184,16319112, insitu]

⑦ XEcto

Description:

The TE cells of the mouse blastocyst that overlie the ICM continue to proliferate after implantation and form the **Extraembryonic Ectoderm (ExE)** and the **Ectoplacental Cone (EPC)**. With gastrulation starting at around E6.5, the extraembryonic mesoderm lineage is generated, an essential layer that originates from the epiblast and forms the allantois and the extraembryonic mesodermal layers of the amnion and chorion. Fusion of the chorion and allantois at ~E8.5 enables the invagination of extra-embryonic mesoderm-derived blood vessels into the chorionic trophoblast layer. The interaction of fetal blood vessels with trophoblast-lined maternal blood sinuses generates the basic structure of the mouse **placental labyrinth**. At E9.5, the multipotent progenitor cells differentiate into **syncytiotrophoblast (STB)** and **sinusoidal trophoblast giant cells (TGCs)**. It should be noted that the **chorion_ecto** in our annotation is more advanced chorion which may already fuse with EPC (after E7.5, **chorionic plate**). The early chorion is more like to be clustered with ExE. **DiffExE** is likely to be the transition state between **EPC** and **ExE**.

Important biological process:

Lybyrinth formation: In the mouse, the entire exchange barrier, from the maternal to the fetal side, is made up of a total of three continuous cell layers, two layers of **syncytiotrophoblast**

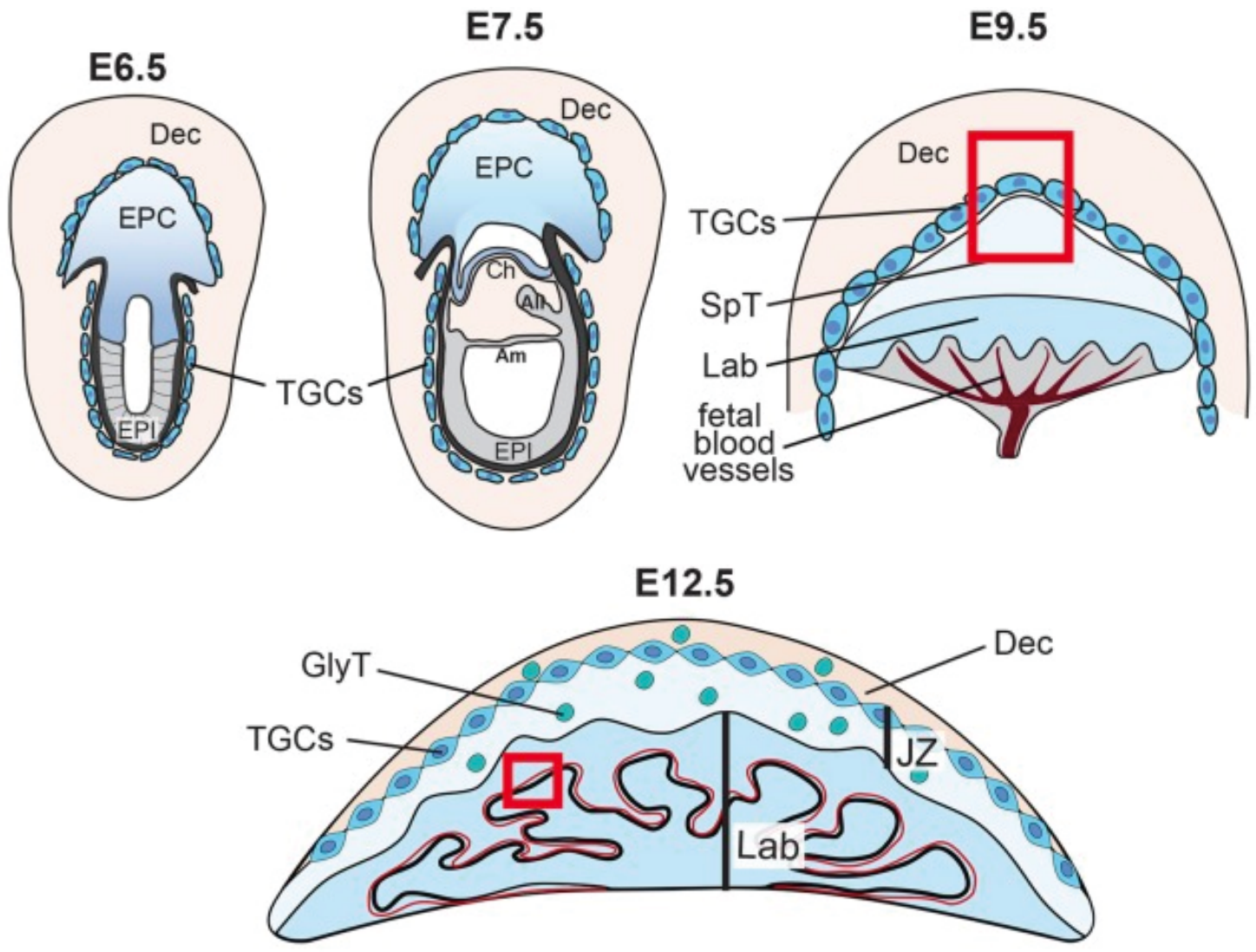

(**SynT-I** and **SynT-II**) and the extra-embryonic mesoderm-derived fetal endothelial cells. **Sinusoidal TGCs** that are likely of chorionic trophoblast origin are also present at the maternal side. These intricate developmental steps start to occur from around mid-gestation in the mouse (E9.5–10.5) and lead to the formation of the so-called labyrinth.

Junctional Zone formation: The **junctional zone (JZ)** is positioned between the labyrinth and the maternal decidua. The JZ originates mainly from cells of the core of the EPC. It contains three main cell types: **spongiotrophoblast cells (SpT)**, **glycogen cells (GCs)** and a layer of **TGCs** that directly border the decidua.

All states: ExE, DiffExE, EPC, Chorion_ecto, Labyrinth_zone, Junctional_zone

**E6.5**	ExE, DiffExE, EPC, Junctional_zone
**E7.0**	ExE, DiffExE, EPC, Junctional_zone
**E7.5**	ExE, DiffExE, EPC, Junctional_zone, Chorion_ecto

**E8.0**	EPC, Junctional_zone, Labyrinthine_zone, Chorion_ecto
**E8.5**	EPC, Junctional_zone, Labyrinthine_zone, Chorion_ecto

Expression pattern:
—————————-ExE enriched———————————————————
Pou3f1: [PMID: 8652411, insitu] E7.0 anterior Epiblast and ExE.
Sox2: [PMID: 12514105, insitu] E6.5 ExE and Epiblast. E7.5 anterior Epiblast, expression in ExE persists. E8.5 Expression is present in the extraembryonic ectoderm portion.
Akap17b(Talia): [PMID: 17302988, insitu] E5.5-7.5 ExE.
Bmp4: [PMID: 10049358, insitu] E5.5-E7.5 ExE.
Cdx2: [PMID: 18781635, insitu] E6.5 ExE.
Eomes,Fgfr2: [PMID: 10330500, insitu] E5.0-E7.0 ExE.
Esrrb(Err2): [PMID: 15003627, IHC] E7.5 ExE.
Tead3: [PMID: 9671946, insitu] E7.5 ExE.
Elf5: [PMID: 23552073, insitu] E6.7 ExE.

—————————-EPC enriched———————————————————
Gjb5: [PMID: 17961533, insitu] E6.5-E8.5, ExE, high in EPC. E9.5 labyrinthine zone, spongiotrophoblast. E15.5 placental glycogen cells.
Ndrg1: [PMID: 17302988, insitu] E6.5 low in ExE, high in EPC. E7.5 EPC, also exist in node.
Ascl2(Mash2): [PMID: 10495277, insitu] E6.5-E7.5 EPC.
Tfap2c: [PMID: 9765260, insitu] E6.5-E7.5 EPC.
Tpbpa: [PMID: 17576768, insitu] E8.5 EPC.
Dlx3: [PMID: 18448564, insitu] E7.5-E9.0 EPC.

—————————-Chorion_ecto enriched———————————————————
Hand1,Prl3d1(Pl1): [PMID: 18448564, insitu] E9.0 chorion.
Prl4a1: [PMID: 18662396, insitu] E8.5 fetal periphery of the ectoplacental cone.
Sdc1: [PMID: 1765004, IF] E7.5 EPC and chorion.
Tcf12: [PMID: 15018808, insitu] E7.0 chorion and amnion.
Map2k3, Xbp1: [PMID: 35764880, scRNA]
Grb2:[PMID: 33141023, scRNA]

—————————-Labyrinth_zone/SynT-II enriched—————————————————
Esx1, Cebpb, Gcm1: [PMID: 18448564, insitu] E8.5-14.5 labyrinth zone.
Tfrc, Ror2, Lgr5, Tcf7l1, Gata2: [PMID: 33141023, scRNA]
Igf2: [PMID: 16338473, insitu] labyrinthine trophoblasts.

—————————-Junctional_zone/Glacogen cells enriched———————————————
Igfbp2,Igfbp3: [PMID: 16338473, insitu] glacogen cells.
Etv6, Meis1: [PMID: 33141023, scRNA]
