# Supplementary material for: Consecutive waves of NODAL inhibition are required for epiblast patterning
Source: Nat Commun. 2026 Oct 9;17:11484. doi: 10.1038/s41467-026-78248-8 (PMC13740313; doi:10.1038/s41467-026-78248-8)
Supplement: Supplementary file 2 [file 41467_2026_78248_MOESM2_ESM.pdf]

nature portfolio

Peer Review File

Consecutive Waves of NODAL Inhibition Are Required for Epiblast Patterning

Corresponding Author: Dr Zachary Smith

This file contains all reviewer reports in order by version, followed by all author rebuttals in order by version.

Version 0:

Reviewer comments:

Reviewer #1

(Remarks to the Author)
The manuscript details an extensive scRNASeq study and elaborate bioinformatic analysis across gastrulation comparing wild type embryos with Lefty 1 and 2 mutant embryos obtained by injection of zygotes with Cas9 and guide RNAs. The results go beyond the traditional view of Nodal signalling as gradients created by the expression patterns of Nodal and its inhibitors, and offer a more dynamic view in which temporally and regionally regulated regions of Nodal high and low states create differentiation waves.

The work is not at odds with existing literature, and adequately refers relevant works. It adds a significant level of complexity and finesse to what was previously known in a very elegant manner, and offers a blueprint for performing similar analysis for the other signalling pathways active at those stages of development. The zygotic injection is a powerful tool in this type of work in which a limited number of embryos suffices to obtain a huge amount of data. It diminishes the risk of biases related to the genetic background of mutant lines, and is a great way to reduce the number of animals required to obtain meaningful results.

The analysis of the mutants at the morphological and patterning levels is thorough and well executed.

The interpretation of the bioinformatic data seems precise and nuanced, and the conclusions are well justified.

Reviewer #2

(Remarks to the Author)
Summary:

Wang and colleagues present a single cell transcriptomic study that aims to elucidate the regulatory consequences of Lefty-mediated antagonism of Nodal signaling in early mouse development. The authors characterize the transcriptomic cell states present in wild-type, LEFTY1 mutant, LEFTY2 mutant and LEFTY1/LEFTY2 double mutant embryos, as well as construct models to explain how these cells progress through these states during the development of each genotype. By constructing a transcriptomically-based Nodal signaling ‘score’, the authors infer spatially-resolved Nodal signaling activity and suggest a modified model for how Nodal signaling intensity and dynamics are shaped by LEFTY expression and interpreted by embryonic cells.

I believe that the transcriptomic characterization presented complements classical studies on this topic and will be of interest to members of the field. I think that the study is suitable for publication in Nature Communications provided that the revisions outlined below are addressed. In short, I believe that (1) the proposed model needs to be clarified, (2) the authors need to do a better job of discussing their findings in the context of well-known studies on this system, and (3) the computational procedures of the paper would benefit from more explanation to make it accessible to traditional embryologists. I also suggest—though not as a precondition for publication— that the authors perform empirical measurements of Nodal signaling activity to test the most interesting predictions of their computational analysis.

Major comments:

1. Clarification of the model

The clearest articulation of the authors' model of Nodal patterning and interpretation appears on page 8 (in the discussion, 'Based on our analyses…shifts cell fates posteriorly'). I found it challenging to understand what was being claimed. The authors present the model as a significant revision of the current status quo—presumably the classical concentration threshold model— however it is not clear to me that what they propose differs from previously proposed models. The discussion of the model needs to be clarified. Specifically, I would raise the following issues:

a. I am unclear on what is meant by a 'differentiation wave'. My best guess is that this means that fates associated with high- and low-Nodal signaling states are not specified simultaneously, but rather separated in time (with high Nodal states adopted first, and low Nodal states adopted later). If this is the intent, I believe it could be stated more clearly. I think this language issue is of particular importance to resolve given the recent claim from the Warmflash lab that Nodal signaling propagates as a wave in an in vitro model of human gastrulation.

b. In this paragraph and in the abstract, the authors claim that Nodal signaling 'toggles' in a 'switch-like' manner between discrete signaling levels. I don't see anything in the data to support this claim; indeed, the authors' own Nodal signaling scores seem to vary along a continuum. If this is to be incorporated as a key part of the model, stronger support is needed.

c. Given the above two points, it seems to me that the major supposition of the model is that Nodal-dependent fate specification occurs in two distinct temporal 'waves'. I am not convinced this represents a substantial change from models that have been proposed previously.

For example, this could be viewed as a twist on the classical threshold model. In that model, cells adopt distinct fates based on Nodal signaling intensity. In the authors data, the same thing happens—high and low Nodal signaling lead to distinct fates— but with a time delay between the two waves. I don't think that the classical articulation of the threshold model makes strong claims about the timing of fate acquisition and could readily accommodate the idea that adoption of 'low threshold' fates takes a bit longer.

Similarly, I think the authors data could be consistent with an 'integrator' model of interpretation in which the duration of signaling plays a role in allocating cells to distinct fates. This has been proposed, for example, in PMID: 17391517. Could the data be consistent with a model in which the 'late fates' just require longer integration of Nodal signaling?

The key point here is that, with the current articulation of the model, it is hard for me to understand precisely what is being claimed and how it relates to what has been proposed before.

d. I think that the Fig. 6 is a great addition to the paper and some sort of graphical illustration is helpful in understanding what the authors mean by their model. However, the presented illustration doesn't clarify what the authors believe to be new. Indeed, from the diagrams of Nodal signaling activity are very similar to what you would find in a review from 15 years ago (e.g. Fig. 4 from PMID 17287255). Could the authors elaborate on what their model and data add to this picture?

e. It is not clear to me— from the current description of the model— if the authors have a view of what feature of Nodal signaling cells read when making decisions. Does their 'wave' model imply that cells read concentrations, duration, integrated intensity, peak intensity, rate of change or something else? How does LEFTY expression alter this quality to organize cell fate acquisition?

2. Integration with prior literature

Throughout the paper, discussion of previous models of Nodal-Lefty patterning in mouse and other systems is inadequate. As noted above, this makes it challenging to understand precisely what change to our understanding the authors are claiming to make. Specifically, points that should be more completely addressed are:

a. Addition of a thorough discussion of the authors proposed model in comparison to the Robertson lab's work on this topic (e.g. PMID: 12842913). This study concluded that Nodal patterning relied on interpretation of graded signals; do the authors wish to refute this model and establish a new one? If they reject those findings, what is the basis? Do they view the data in that paper consistent or inconsistent with the findings here?

b. Several studies in mouse and other organisms have explored how loss of Lefty feedback impacts Nodal pattern formation and downstream cell fate acquisition. I think the paper would benefit from a dedicated section in the discussion that covers Lefty's role in zebrafish (PMID: 29215332, PMID: 12498687), xenopus (PMID: 16405884) mouse (PMID: 10518210) and gastruloid (PMID: 35079017) models, and explains how the findings of this paper confirm or revise the lessons and models generated by those studies. Does this study reveal something new about the effect of Lefty expression on Nodal signaling, or is it consistent with what we see in other organisms?

3. Computational pipeline

I believe that this study would be of interest to traditional embryologists, however the level of technical description is likely to reduce accessibility to this audience. I would regard myself as a quantitatively literate developmental biologist— but not a transcriptomics expert— and I found the main flow of the technical arguments hard to follow. I would suggest the following:

a. It would be helpful to add a user-friendly cartoon of the computational analysis pipeline that helps non-experts understand what is going on. For example, could the concept of metacells be intuitively illustrated? What, at a 'gut level' is GeoSeq doing? I leave this issue to the authors discretion, however I do believe that the reach of the paper could be substantially increased by making the technical side more accessible.

b. Inferring the Nodal signaling activity through the Nodal score is an interesting concept. However, I don't think the procedure was well explained given the centrality to the paper's argument. I was unable to locate a description of its calculation in the Methods section; as far as I can tell it only appears in the legend of Extended Data Figure 3. An extended discussion of how it was calculated would be helpful.

c. If I understand correctly from Extended Data Figure 3: the Nodal score for each cell is simply the weight of PC1 from the PCA? If this is true, I think the rationale needs to be extended and defended in the methods. I see that several Nodal targets are loaded significantly in PC1, but presumably it could also represent the action of other biological processes? Why use PC1 rather than a weighted sum of known Nodal target genes? Given that PC1 explains the most variance in the data of any PC, I am concerned that this measure represents some other large and complex trend in the data (for example, developmental time or a combination of signaling pathways?).

4. Optional: supporting measurements of Nodal signaling activity

To my eye, the most surprising output of the computational measurements was the transient cessation of Nodal signaling activity in E 7.0 embryos (e.g. Fig. 2a). From measurements of the expression of NODAL and LEFTY genes in the literature, one would expect signaling activity to begin throughout the entire embryo (as shown in Fig. 2a and f) and monotonically become restricted to the posterior side and primitive streak. Instead, the authors seem to observe a transient shut down in Nodal signaling before a second wave of activity. This would be a really neat prediction to test experimentally; I think it would be as simple as a short timecourse of a-pSmad2 immunostainings over the relevant stages. If the transient 'stop' in Nodal signaling activity were observed, I think it would demonstrate the power of the transcriptomic measurements over the traditional approach of in situ visualization of gene expression.

Reviewer #3

(Remarks to the Author)
In this study Wang et al. dissect the role of Nodal signalling in patterning the mouse primitive streak. Using single-cell RNAseq of wild-type, and Nodal antagonist Lefty1 and Lefty2 CRISPR knockouts they track the signalling output of Nodal signalling at different stages in different cells fates during mouse gastrulation and the consequence of Nodal signalling perturbation. The scale of work in terms of the spatial and temporal resolution is impressive, providing novel insights into Lefty-controlled waves of Nodal activity in the streak.

However, my major concern is that this study overlooks the primary AVE migration phenotype of Lefty1 mutants. This is in part due to neglecting previous papers on Lefty1 knockouts, and in part due to the loss of spatial information in the single-cell RNAseq methodology used to analyse these mutants. As a consequence, the analysis and conclusions of the Lefty1 mutant phenotype are oversimplified.

Major points:

Nodal score
- The Nodal score / responsive gene list was derived from ChIP-Seq/RNAseq done in embryoid bodies, containing only epiblast derived lineages. Does this neglect Nodal responsive genes that may be specific to the extra-embryonic lineages? What about the Nodal response in the extra-embryonic ectoderm, and visceral endoderm, is this fully captured in the model?
- Can the authors validate their computational Nodal response output with pSmad2 staining to see the degree of overlap? I would recommend Brumm et al 2025 Developmental Cell https://doi.org/10.1016/j.devcel.2024.10.020 protocol for the best pSmad2 staining I've seen in gastrulating mouse embryos.

Lefty1 knock-out
- "Although no discernible morphological differences are observed between mutant and WT embryos at ~E6.5, disparities between LeftyKOs become more apparent after E7.5". The authors conclude from their Lefty1 knockout-results that Lefty1 antagonism of Nodal is the main driver of the phenotype A-P patterning and differentiation defects seen in mutant embryos at E7.5. However, the discussion and analysis overlook the primary phenotype of Lefty1 mutants: defects in AVE migration (Trichas et. al., 2011 PLOS Biology https://doi.org/10.1371/journal.pbio.1001019). Lefty1 mutants can be classified at E6.25

as having AVE over-migration, or DVE/AVE migration arrest. The work of Trichas et al. should be referred to in the paper.
- It appears from the images in the extended data figures (Ext. data 4e II and Ext data 7b) that Lefty1KO E7.5 embryos exhibit thickened distal regions, and distally localised Cer1, indicative of a DVE/AVE migration arrest. This is further supported by failure in A-P rotation and proximally localised primitive streak, marked by Brachyury (Fig. 5b). Therefore, this study's Lefty1KO phenotype recapitulates the one described in Trichas et. al., and warrants further analysis at earlier stages to identify the onset and mechanism of this mutant phenotype.
- Given that there are Lefty1KO AVE defects at E6.5 it raises the question of whether the defects in epiblast patterning seen at E7.5 are caused by
(i) only the loss of Lefty1 antagonism of Nodal as concluded in this paper
or
(ii) failure of proper AVE migration, influencing antagonism of multiple signalling pathways (e.g. AVE-secreted Cer1 on BMP, and DKK1 on WNT) in addition to the loss of antagonism of Lefty1 on Nodal
- The cause of AVE migration defects in Lefty1KO is unclear. The single-cell data set in this study offers a great opportunity address this question and understand the driving cause of the phenotypes seen in Lefty1 mutants. Can differences in the visceral endoderm of WT vs Lefty1KO at E6.5 be detected? A pre-print (Stower et al. 2023 https://doi.org/10.1101/2023.03.31.534937) suggests that Lefty1-dependent mechanical heterogeneity in the EmVE and ExVE is the primary cause for AVE migration defects in Lefty1KO embryos, originating as early as E5.5. It would be interesting to know if this can be seen at a transcript level.

Version 1:

Reviewer comments:

Reviewer #2

(Remarks to the Author)
I thank the authors' for the attentive responses to my comments. I feel the revised manuscript more clearly presents the authors' conception of how the NODAL and LEFTY are working together to pattern the embryo. I support publication of the manuscript.

Congratulations to the authors on a nice piece of work!

Reviewer #4

(Remarks to the Author)
This manuscript describes analysis of an extensive single-cell RNA-seq (scRNA-seq) time series dataset over murine embryonic development in Lefty1 and Lefty2 KO settings and a comparable WT control condition. With these data, the main goal is to learn where and how each KO impacts progression of transcriptomic states, especially in light of NODAL signaling which is expected to mediate the impacts of these perturbations. To accomplish this, the authors perform relatively standard scRNA-seq processing and embedding, which reveals (1) the absence of later gastrulation states which do appear in the control, indicating an aberrant arrest of development in mutant conditions, (2) a map of Lefty1/2 transcription which generates hypotheses on why this happens, (3) a map of downstream NODAL signaling (via integration with ChIP-seq data and unbiased transcriptomic analysis).

Overall, the scRNA-seq analysis is sound, following widely accepted methods for data cleaning (CellBender), filtering (Scrublet), and embedding (Seurat). Moreover, the conclusions drawn from the scRNA-seq data in this study are largely derived from extremely strong signals (e.g. the absence of entire large clusters in some datasets versus others). Even with slight variations in analysis, I imagine the major conclusions would hold. As it currently stands, I believe the analysis itself is acceptable for publication.

That said, I have some comments on the Methods, the most major of which surround its clarity and the justification for various decisions. I detail these below:

1. Data Integration: Integration of mutant with WT datasets is arguably the most important step of the analysis for this paper, as it drives most of the conclusions regarding the effects of mutations.
a. The language used throughout the Results (e.g. "project mutant onto WT reference atlas") seemed to imply that the embedding was first generated on WT cells, and mutant cells are somehow embedded on top of that space. However, the Methods don't describe such a procedure, leading me to believe that these are all just joint embeddings without additional harmonization. This deserves clarity, as the most important result of the paper hinges on how this was done.
b. To justify and understand the embedding in either case, I would suggest adding some metric to visualize the mixing of Lefty1/2 and WT states within the spaces that they overlap. An example might be the entropy of the distribution of conditions for each cell's neighbors, such that high entropy suggests strong mixing.
c. Relatedly, I am curious whether there is any differential expression between cells which are well-matched at the integration level, but come from different conditions. The Milo tool (Dann et al 2022) would be suitable to check for these more subtle differences which can often be missed by focusing on coarse compositions.

2. Data processing: I believe the data processing steps are all sensible, but could be further justified. In particular:
a. Was ambient RNA correction with CellBender a necessary step to obtain the results? These sort of large-scale changes to the data are sometimes important, but can make major changes to the underlying data structure. This should be justified

with an explanation or, ideally, with a comparison to un-corrected data embeddings.
b. Can the authors please clarify the motivation for their UMI threshold selection for filtering (as opposed to more standard approaches, e.g. taking the lower mode in the UMI count distribution)? Was this threshold defined per independent sample?
c. Do the Scrublet identified doublets have a higher proportion of cells mixed across assigned embryos? This would nicely validate the doublet detection.
d. What is meant by “shadow cluster” in this case – please define more clearly.
e. Please justify why metacell-level aggregation becomes necessary for NODAL score computation. As this is typically done to circumvent sparsity, I assume some issues might have been encountered in attempting this at the single-cell level.
f. Relatedly, why are cell cycle and other confounding signals removed only at this stage of the analysis? Were highly variable genes not used earlier for PCA computation and if not, then why?

Minor Points:
The authors spend a lot of effort developing a signature for NODAL output, but then rely on a fully-unbiased method for module detection (NMF). A better harmonization of these could be produced by using a method like Spectra (Kunes et al 2024). This isn’t a necessary addition – just a suggestion.

Finally, I would suggest clarifying in each UMAP precisely which cell subsets are being plotted. For example, it took me a while to understand which cells are WT/Lefty1/2 mutants in Fig 2a,c.

Open Access This Peer Review File is licensed under a Creative Commons Attribution 4.0 International License, which permits use, sharing, adaptation, distribution and reproduction in any medium or format, as long as you give appropriate credit to the original author(s) and the source, provide a link to the Creative Commons license, and indicate if changes were made.
In cases where reviewers are anonymous, credit should be given to 'Anonymous Referee' and the source.
The images or other third party material in this Peer Review File are included in the article’s Creative Commons license, unless indicated otherwise in a credit line to the material. If material is not included in the article’s Creative Commons license and your intended use is not permitted by statutory regulation or exceeds the permitted use, you will need to obtain permission directly from the copyright holder.
To view a copy of this license, visit https://creativecommons.org/licenses/by/4.0/

We thank all reviewers for their constructive comments, which we have diligently worked to incorporate into our revised manuscript over the past year. In particular, we have worked to make the following improvements:

1. **Clearer presentation of the data from major findings to their implications,** including improved figure representations for major transitions in early differentiation and primitive streak establishment. These include refined analyses of the early patterned epiblast as it matures into spatially localized regions (**Fig. 2**) and the distinct anterior and posterior trajectories in neural ectoderm formation (**Fig. 4**). This should clarify the resolution with which we can characterize the transcriptional dynamics of early gastrulation, as well as the robustness of our conclusion that NODAL-based patterning requires transient waves of inhibition.
2. **Morphological and functional validation of scRNA-seq based conclusions**, including careful imaging-based dissection of delaminating axial precursors within the distal tip (**Fig. 2g, h**) and validation of the differentiation wave model by ex vivo culture with the NODAL inhibitor SB431542 (**Fig. 3**). These validate the finding that the gastrulation embryo requires NODAL signal suppression to license downstream differentiation.
3. **Detailed dissection of the interaction between ChIP-seq validated SMAD2/3 targets with cell state-specific transcriptional programs (Fig. 5).** This highlights the degree to which SMAD2/3 signal transduction supports state specific programs, and highlights the failure of Lefty1 or Lefty2 mutants to appropriate modulate these transitions.

Alongside more specific requests and corrections, we hope that these additions have improved the manuscript in the reviewer's eyes, and that you now consider it sufficient for publication in *Nature Communications*.

Point by point responses:

Reviewer #1 (Remarks to the Author):

The manuscript details an extensive scRNASeq study and elaborate bioinformatic analysis across gastrulation comparing wild type embryos with Lefty 1 and 2 mutant embryos obtained by injection of zygotes with Cas9 and guide RNAs. The results go beyond the traditional view of Nodal signalling as gradients created by the expression patterns of Nodal and its inhibitors, and offer a more dynamic view in which temporally and regionally regulated regions of Nodal high and low states create differentiation waves.

The work is not at odds with existing literature, and adequately refers relevant works. It adds a significant level of complexity and finesse to what was previously known in a very elegant manner, and offers a blueprint for performing similar analysis for the other signalling pathways active at those stages of development. The zygotic injection is a powerful tool in this type of work in which a limited number of embryos suffices to obtain a huge amount of data. It diminishes the risk of biases related to the genetic background of mutant lines, and is a great way to reduce the number of animals required to obtain meaningful results.

The analysis of the mutants at the morphological and patterning levels is thorough and well

executed.

The interpretation of the bioinformatic data seems precise and nuanced, and the conclusions are well justified.

We thank the reviewer for their positive comments, including our revised "differentiation wave" model for NODAL-based epiblast patterning, our experimental and analytical pipelines, as well our validation. We hope you find this revised manuscript to be further improved at the level of interpretation and clarity.

Reviewer2:

Summary: Wang and colleagues present a single cell transcriptomic study that aims to elucidate the regulatory consequences of Lefty-mediated antagonism of Nodal signaling in early mouse development. The authors characterize the transcriptomic cell states present in wild-type, LEFTY1 mutant, LEFTY2 mutant and LEFTY1/LEFTY2 double mutant embryos, as well as construct models to explain how these cells progress through these states during the development of each genotype. By constructing a transcriptomically-based Nodal signaling 'score', the authors infer spatially-resolved Nodal signaling activity and suggest a modified model for how Nodal signaling intensity and dynamics are shaped by LEFTY expression and interpreted by embryonic cells.

I believe that the transcriptomic characterization presented complements classical studies on this topic and will be of interest to members of the field. I think that the study is suitable for publication in Nature Communications provided that the revisions outlined below are addressed. In short, I believe that (1) the proposed model needs to be clarified, (2) the authors need to do a better job of discussing their findings in the context of well-known studies on this system, and (3) the computational procedures of the paper would benefit from more explanation to make it accessible to traditional embryologists. I also suggest—though not as a precondition for publication— that the authors perform empirical measurements of Nodal signaling activity to test the most interesting predictions of their computational analysis.

We thank the reviewer for their constructive comments and expertise, and have worked to both clarify the model and contextualize our findings with additional validation and analysis. Regarding the application of empirical read outs, which we discuss below, a component of our argument is the degree to which single cell technologies can be combined with in vitro experiments such as ChIP-seq to provide higher resolution, data-based descriptions of signal transduction and internal state. We have worked hard to improve these visualizations and descriptions in **Figure 5** and hope these will be of value to you and the field.

Major comments:

1. Clarification of the model:

The clearest articulation of the authors' model of Nodal patterning and interpretation appears on page 8 (in the discussion, 'Based on our analyses…shifts cell fates posteriorly'). I found it challenging to understand what was being claimed. The authors present the model as a significant revision of the current status quo—presumably the classical concentration threshold model—however it is not clear to me that what they propose differs from previously proposed models. The discussion of the model needs to be clarified. Specifically, I would raise the following issues:

a. I am unclear on what is meant by a 'differentiation wave'. My best guess is that this means that

fates associated with high- and low-Nodal signaling states are not specified simultaneously, but rather separated in time (with high Nodal states adopted first, and low Nodal states adopted later). If this is the intent, I believe it could be stated more clearly. I think this language issue is of particular importance to resolve given the recent claim from the Warmflash lab that Nodal signaling propagates as a wave in an in vitro model of human gastrulation.

We thank the reviewer for this comment and have worked to improve the description of our model in a revised **Figure 6**. Briefly, the distinction we aim to highlight is that cell fates arising from progenitor states with high and low Nodal signaling are not specified simultaneously, but rather sequentially over time and through periods where NODAL activity is distinguished as part of Lefty's inclusion as an immediate early target. Furthermore, when high Nodal progenitors transition to a different cell fate, the timely removal of Nodal activity appears to be essential to license proper differentiation.

To clarify this concept, we have revised the description of the differentiation wave in the discussion:

"Based on these studies and our analysis, we propose that a modified "sequential inhibitory wave model" best explains the dynamics of NODAL signaling as inferred from single cell data, as well as the highly convergent phenotypes of our Lefty mutant time series. In particular, Nodal signal transduction does not appear to encode positional information in a one-to-one manner or to direct pluripotent cells towards multiple fates. Instead, it initially drives cells towards a common differentiation competent state, which remains plastic for all epiblast derivatives (except for forebrain and midbrain) but more efficiently toward extraembryonic mesoderm, axial mesoderm or endoderm…"

We appreciate your valuable suggestion and have incorporated the findings from Liu et al., 2022, which describe how Nodal activity propagates via a relay mechanism, exhibiting wave-like behavior by inducing neighboring cells to transcribe Nodal, into our discussion section. Please see the added text below:

"Key challenges to the traditional gradient model include the ever changing size and shape of the gastrulating epiblast (Snow 1977, Warga and Kimmel 1990), as well as the unstable short-range nature of the NODAL protein itself, which is more amenable to relay-like propagation to neighboring cells (Branford and Yost 2002, Chen and Schier 2002, Sakuma, Ohnishi Yi et al. 2002, Cha, Takahashi et al. 2006, Liu, Nemashkalo et al. 2022)."

b. In this paragraph and in the abstract, the authors claim that Nodal signaling 'toggles' in a 'switch-like' manner between discrete signaling levels. I don't see anything in the data to support this claim; indeed, the authors' own Nodal signaling scores seem to vary along a continuum. If this is to be incorporated as a key part of the model, stronger support is needed.

We apologize for any confusion caused by our previous wording and have worked to make our language more precise. As our Nodal signaling scores indeed exhibit a continuous range, our

intention was not to suggest a strict binary switch in signaling levels. Rather, we aimed to convey that Nodal signaling regulates discrete cell fate choices during sequential differentiation events.

To clarify this, we have revised the relevant expressions to emphasize that Nodal activity "modulates" cell fate decisions rather than toggling between high and low signaling states. These changes ensure that our description more accurately reflects the data presented.

c. Given the above two points, it seems to me that the major supposition of the model is that Nodal-dependent fate specification occurs in two distinct temporal 'waves'. I am not convinced this represents a substantial change from models that have been proposed previously. For example, this could be viewed as a twist on the classical threshold model. In that model, cells adopt distinct fates based on Nodal signaling intensity. In the authors data, the same thing happens—high and low Nodal signaling lead to distinct fates— but with a time delay between the two waves. I don't think that the classical articulation of the threshold model makes strong claims about the timing of fate acquisition and could readily accommodate the idea that adoption of 'low threshold' fates takes a bit longer.

Similarly, I think the authors data could be consistent with an 'integrator' model of interpretation in which the duration of signaling plays a role in allocating cells to distinct fates. This has been proposed, for example, in PMID: 17391517. Could the data be consistent with a model in which the 'late fates' just require longer integration of Nodal signaling?

The key point here is that, with the current articulation of the model, it is hard for me to understand precisely what is being claimed and how it relates to what has been proposed before.

We agree that, at a superficial level, our observations might appear compatible with either a classical threshold model or an integrator model. However, we do not believe that either framework adequately captures the key features of NODAL-dependent fate specification revealed by our data.

With respect to the classical threshold model, our results do not support the existence of multiple stable NODAL thresholds that directly instruct distinct fates. Instead, we observe that cells remain in a common NODAL-responsive progenitor state until suppressed by Lefty1/2 induction. In this view, NODAL does not actively drive cells toward progressively "higher" or "lower" threshold fates. Rather, persistent NODAL signaling inhibits full differentiation. Fate transitions occur only once NODAL activity is effectively shut down, primarily through the action of LEFTY and other antagonists. Thus, what are often referred to as "low-threshold" fates are not specified by reduced NODAL signaling per se, but by the *termination* of NODAL signaling altogether. This conceptual distinction is not explicitly addressed in classical threshold models.

For example, our model shows that extended NODAL signaling leads to the production of organizer cell states, but that these cells only partially differentiate further into bona fide node or notochord in the absence of either *Lefty* gene (**Fig. 2g, h, see below**). Such a morphological

phenotype is well explained by a differentiation wave where NODAL first specifies "anterior primitive streak" but that these cells cannot differentiate further until after LEFTY-based suppression.

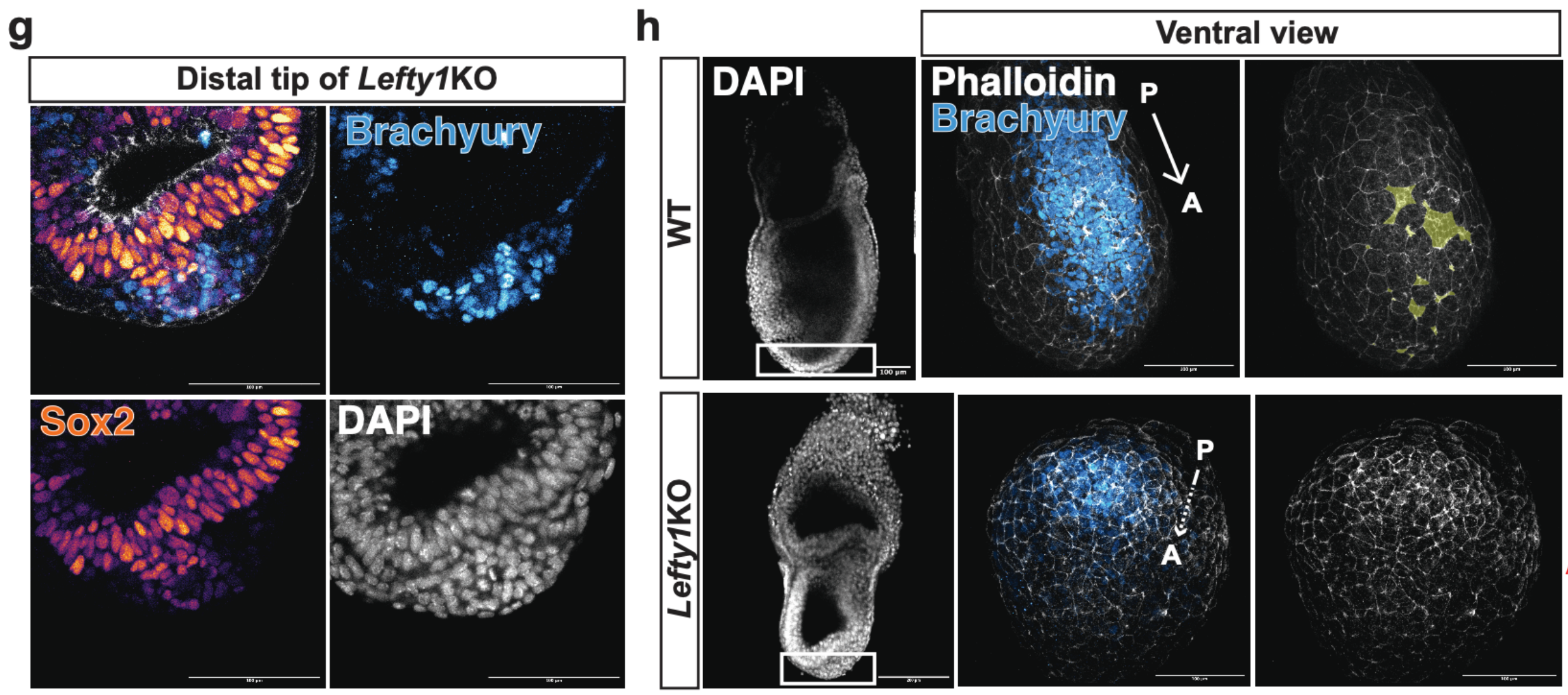

Revised Figure 2g. Magnified lateral view of the distal region of a representative optical section from a *Lefty1*KO embryo at E7.5, showing accumulation of cells between the epiblast and visceral endoderm layer. Sections were stained with DAPI (white), anti-Sox2 (red), and anti-Brachyury (blue). Within the mutant, a subset of the accumulated cells are SOX2^{+}/Brachyury^{+} and remain contiguous with the epiblast layer, suggesting that these cells were recently and rapidly delaminated from the epiblast but fail to properly differentiate into formal axial mesoderm.

Revised Figure 2h. Magnified ventral view of the distal region reconstructed from image stacks of WT and *Lefty1*KO embryos at the early head-fold stage. Phalloidin staining highlights cortical microtubule organization, revealing cell shape and size. Endodermal cells in *Lefty1*KO embryos are markedly smaller than those in WT embryos, and intercalation of differentiating node cells (highlighted in yellow) is not observed. Brachyury (T) staining marks the underlying primitive streak and node cells; notably, in *Lefty1*KO embryos, Brachyury^{+} cells remain clustered at the distal region and fail to extend anteriorly to the extent observed in control embryos.

Second, our model also differs from an integrator-based interpretation in which late fates arise through prolonged accumulation of NODAL signaling. We instead propose that the emergence of later fates requires suppression of NODAL activity, rather than continued signal integration. This distinction is supported by several observations. Notably, *Lefty* mutant embryos retain undifferentiated epiblast cells for extended periods and fail to progress toward definitive mesoderm and neural ectoderm, despite prolonged and elevated NODAL target gene expression. If late fates were specified by longer integration of NODAL signaling, one would predict enhanced or accelerated production of definitive mesoderm in these mutants, which is contrary to our findings (we found the highest proportion of DE over our time course is not significantly higher than that of WT). We have now added pharmacological NODAL inhibition in roller culture (**Fig. 3e**, see

relevant screenshots below) which we find rescues epiblast caudalization and mesodermal differentiation, indicating that removal—rather than accumulation—of NODAL signaling is the critical step enabling lineage progression.

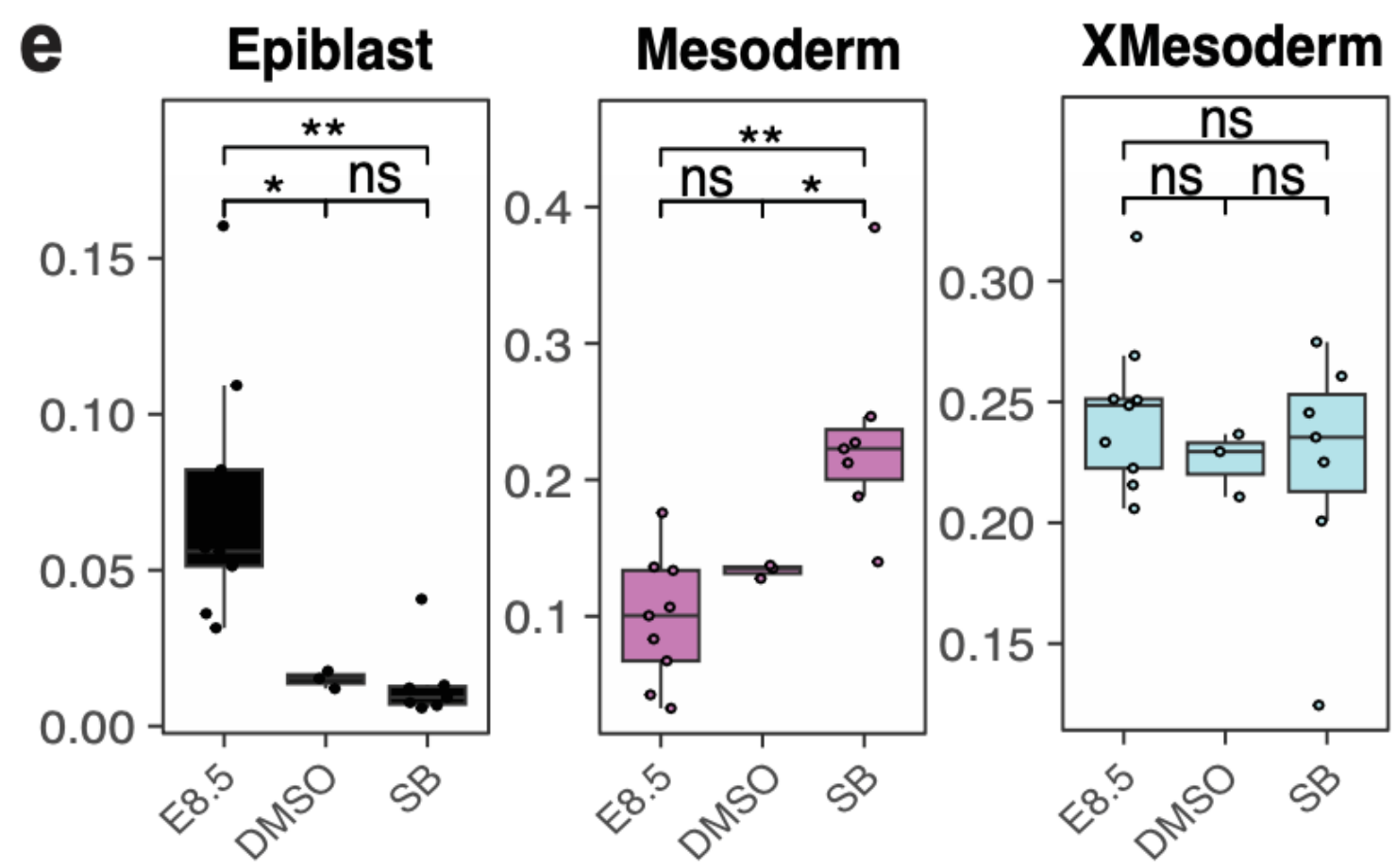

Revised Figure 3e. Per-embryo proportions of epiblast, mesoderm, and extraembryonic mesoderm (XMesoderm) germ layers, with each dot representing an individual embryo from E8.5 *Lefty2*KO, DMSO control, or SB431542 treatment groups. *Lefty2*KO embryos cultured with SB431542 exhibit increased mesoderm differentiation, while no significant change is observed in extraembryonic mesoderm differentiation. This change is apparent across multiple distinct definitive mesoderm states (see **Extended Data Fig. 6**). Statistical significance was evaluated using the Wilcoxon rank-sum test, with *P < 0.05, **P < 0.01, and ***P < 0.001.

Taken together, we propose that NODAL activity propagates across the epiblast in a wave-like manner, with cells transition through early NODAL "on" states during specification but require this signal to be extinguished to license further differentiation. Furthermore, variations in apparent signaling intensity primarily reflect differences in activation timing, rather than stable graded states encoding fate identity. Cell fate decisions are then gated by the timing of NODAL signal termination, which defines discrete competency windows for other morphogens signals. This interpretation is further consistent with prior observations showing that even minimal NODAL signaling, or Nodal expression lacking positive feedback, is sufficient to rescue gastrulation defects in *nodal* mutants (Vincent, Dunn et al. 2003).

We acknowledge that it is difficult to fully exclude threshold- or integrator-based interpretations, particularly given the extensive feedback regulation within the NODAL pathway. However, we believe that the sequential inhibitory wave model provides a more parsimonious and intuitive explanation for the prolonged progenitor states, the convergence of *Lefty* mutant phenotypes, and the requirement for precise NODAL shutdown in enabling orderly fate allocation during gastrulation. Please see below a revised paragraph in our discussion that discusses the nuances between these classic models and the implications from our own work:

Nuances between classic models: "morphogen gradients have been thought to induce differential gene expression according to ligand concentration, leading to graded levels of signal activity that direct distinct cell fate changes (Green and Smith 1991)….. A major refinement of gradient model has been the integrator model, which proposes that Nodal signaling accumulates overtime through autoregulation until sufficient Lefty is translated to inhibit further activation (van Boxtel, Chesebro et al. 2015)… the integrator model emphasizes the length of exposure to Nodal signals plays a crucial role in cell fate specification to ensure mesoderm and endoderm formation despite lower ligand concentrations (Hagos and Dougan 2007)… Despite their differences, both models share the assumption that internal signaling thresholds govern cell fate decisions. However, while cells exhibit varying levels of Nodal signaling activity, there is limited evidence supporting the existence of a strict signaling threshold that dictates distinct cell fates."

Implications from our work: "While our model is not fundamentally different from previous ones, it is modified to better align with the biophysical properties of Nodal-Lefty in mammalian embryo system and incorporates the phenotypic outcomes of persistent NODAL induction. In particular, our data highlight the uniform phenotypes of Lefty *KOs*, where all cell types within the range of NODAL activity exhibit prolonged expression of nearly all NODAL target genes. Such a response strongly indicates that both Lefty1 and Lefty2 are required to constrain the duration and cellular reach of NODAL signaling, rather than to impose module-specific transcriptional outputs."

d. I think that the Fig. 6 is a great addition to the paper and some sort of graphical illustration is helpful in understanding what the authors mean by their model. However, the presented illustration doesn't clarify what the authors believe to be new. Indeed, from the diagrams of Nodal signaling activity are very similar to what you would find in a review from 15 years ago (e.g. Fig. 4 from PMID 17287255). Could the authors elaborate on what their model and data add to this picture?

Thank you for your valuable feedback. We have revised **Figure 6** by expanding the single schematic into three panels to provide a clearer and more detailed illustration of our model. Please see the revised figure below:

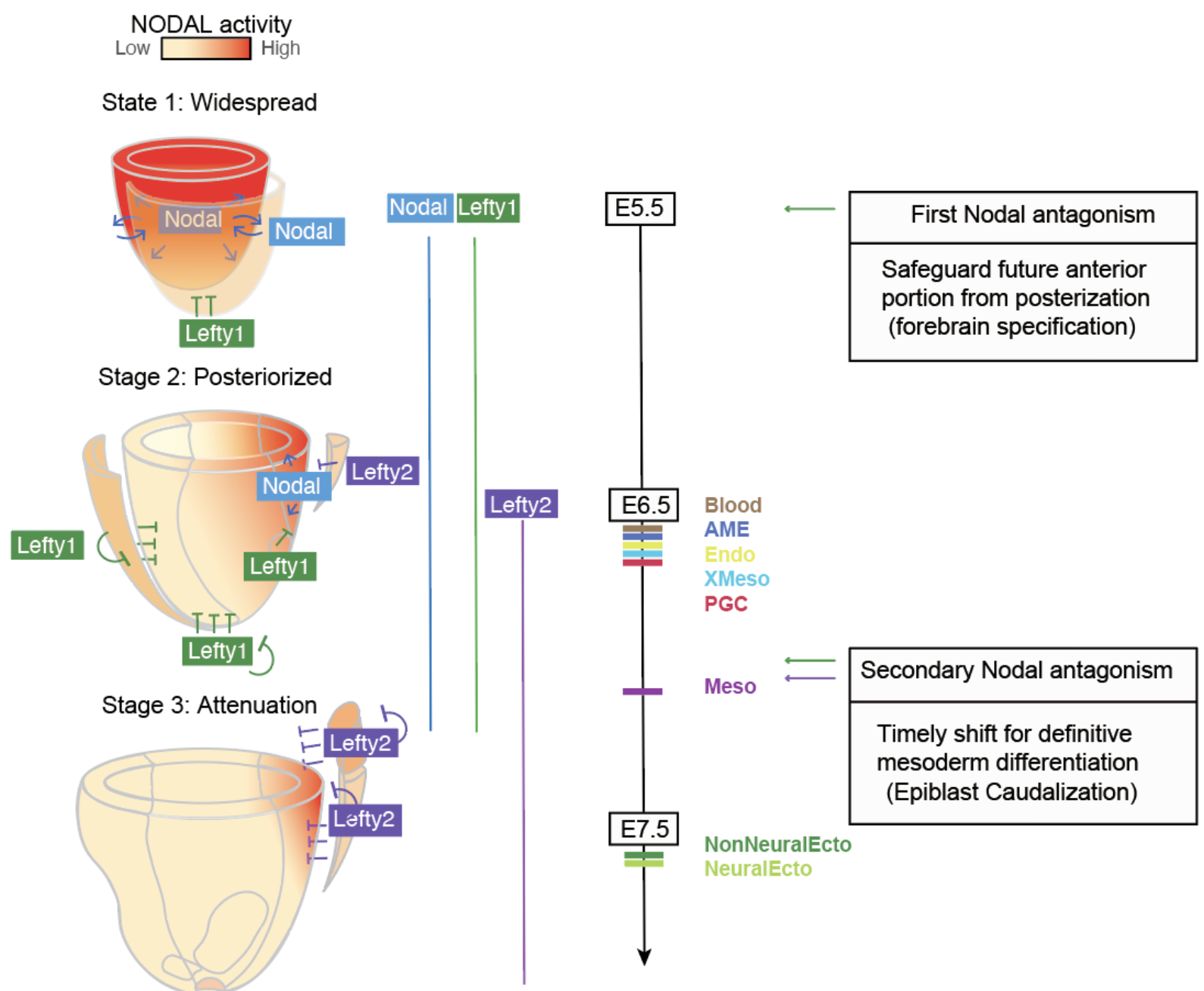

Revised Figure 6. NODAL-based patterning proceeds through multiple phases of LEFTY-based suppression.

On the left, we depict the expression patterns of Nodal, Lefty1, and Lefty2, illustrating how Nodal activity is regulated during gastrulation by these three key modulators. While this schematic bears similarity to the 2007 review (Shen 2007), where Lefty1 is primarily expressed in the anterior visceral endoderm (AVE) and contributes to the downregulation of Nodal activity in the anterior epiblast, our model provides additional insights. Specifically, we highlight that Lefty1, like Lefty2, also expressed in the posterior region, where they play a crucial role in terminating Nodal signaling in the posterior embryo.

On the right, we introduce a refined temporal framework for Nodal antagonism, distinguishing between primary and secondary phases. Primary Nodal antagonism is predominantly mediated by Lefty1, whereas secondary Nodal antagonism involves both Lefty1 and Lefty2. We also illustrate the functional consequences of impairments in these phases: Lefty1 KO embryos exhibit defects in both phases, whereas Lefty2 KO embryos primarily display defects in the secondary antagonism, with only minor disruption to the primary phase. These observations align with the proposed upstream regulatory role of Lefty1 in modulating Nodal activity.

We hope these revisions clarify our model and highlight its novel contributions beyond previous studies.

e. It is not clear to me— from the current description of the model— if the authors have a view of what feature of Nodal signaling cells read when making decisions. Does their 'wave' model imply

that cells read concentrations, duration, integrated intensity, peak intensity, rate of change or something else? How does LEFTY expression alter this quality to organize cell fate acquisition?

Our data do not support a model in which cells interpret NODAL signaling through a fixed internal threshold. Instead, our results suggest that NODAL signaling may function primarily to maintain cells in a permissive, plastic progenitor state, rather than to directly instruct distinct fate choices. In this framework, most NODAL responding states experience a common NODAL-responsive transcriptional program, with fate diversification occurring only after primary NODAL activity is effectively terminated.

We have now updated a **revised Figure** 5 to showcase these effects. Namely, we see that the specific SMAD2/3 targets detected in differentiating EBs can be "phased" into one of five distinct developmental programs, each of which emerges within a specific cell type at a specific time (see Fig. 5g). However, upon Lefty knockout, we observe an overall upregulation of most NODAL target genes, without specific tuning of individual NODAL target modules (see **Fig. 5f**).

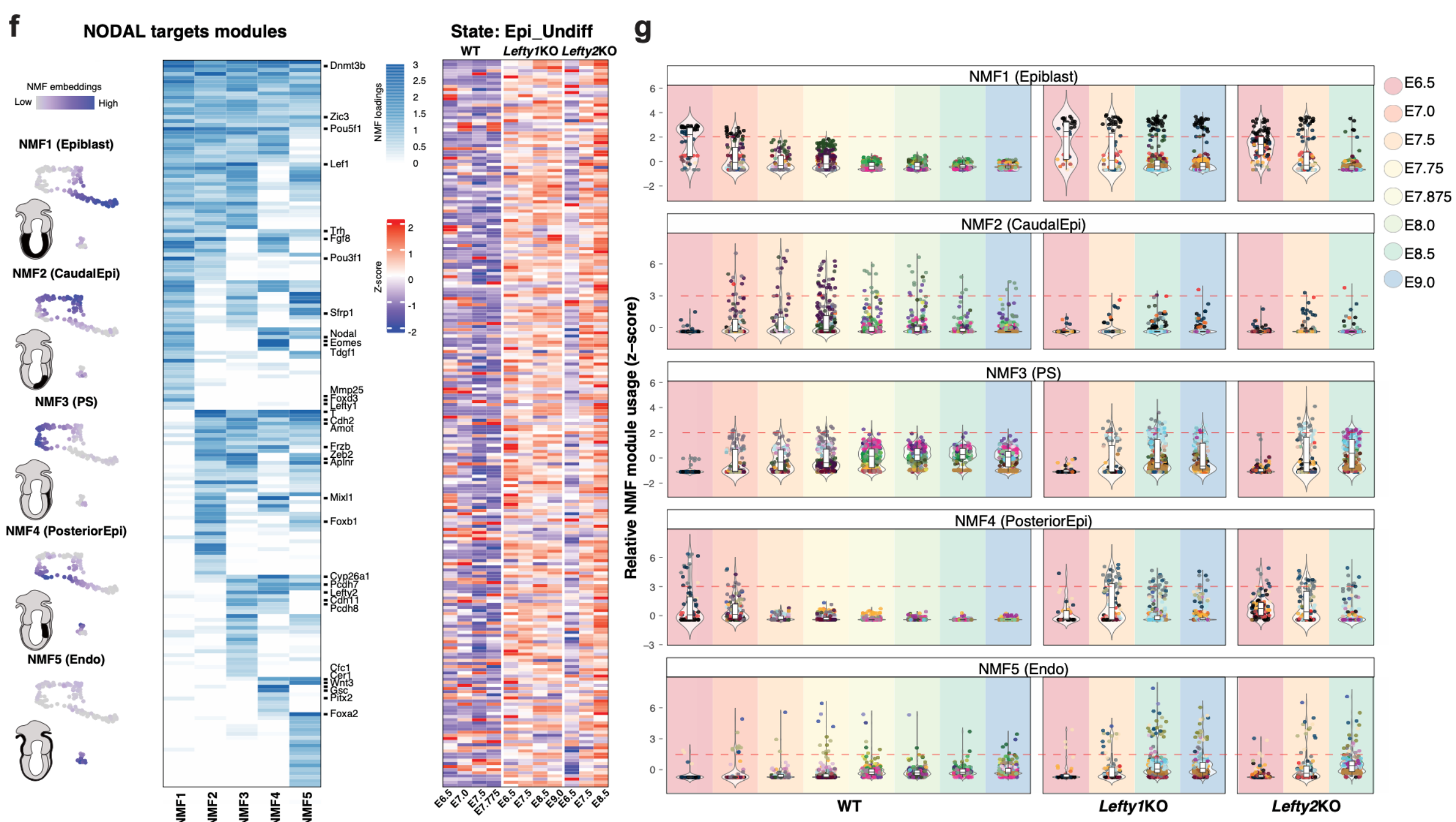

Revised Figure 5f. *Left*: Five non-negative matrix factorization (NMF) embeddings of MCs computed from NODAL target genes, with modules named according to their spatial enrichment in Epi_Undiff (Epiblast), Epi_Caudal (CaudalEpi), primitive streak (PS), Epi_Posterior (PosteriorEpi), and definitive endoderm, visceral endoderm, and node lineages (Endo). *Middle*: Heatmap showing gene loadings for each NMF module. *Right*: Row-scaled expression of genes, ordered identically to the middle heatmap, in epiblast states from WT and *Lefty*KO datasets. Overall, this analysis highlights the overall upregulation of NODAL target genes. Notably, *Lefty1*KO embryos show higher expression of Nodal target genes at E6.5 compared with *Lefty2*KO, indicating an earlier role for Lefty1 in restraining NODAL signaling activity.

Revised Figure 5g. Scaled NMF module usage across developmental stages calculated from merged MCs of WT and *Lefty*KO datasets, with MCs colored by their dominant cell state. NMF modules 1, 4, and 5 exhibit prolonged usage within similar cell states in *Lefty*KO embryos, with Module 1 associated with Epi_Undiff and Epi_Posterior, Module 4 with Epi_Organizer and XPS, and Module 5 with VE, DE, and Node lineages. In contrast, Module 2, representing a caudal epiblast enriched subset of Nodal target genes, is reduced in *Lefty*KO embryos, consistent with the failure to stably induce these states within the mutants. Module 3 displays comparatively modest changes.

According to these data, our "sequential inhibitory wave" model does not imply that cells explicitly read a single quantitative feature of the signal—such as absolute concentration, peak amplitude, or integrated signal duration—to select between multiple fates. Rather, we propose that cells respond to the presence versus effective absence of NODAL signaling to transition between nearby states. Without NODAL modulation, cells remain trapped in their undifferentiated or early progenitor transcriptional state and cannot proceed to subsequent stages of differentiation. Only when NODAL signaling is shut down—through the combined action of LEFTY and other antagonists—can cells exit this state and initiate other downstream differentiation programs.

Within this view, parameters such as signal concentration, duration, or rate of change may influence the *timing* of this shutdown by modulating how rapidly LEFTY-mediated antagonism accumulates and suppresses NODAL signaling. However, these parameters do not appear to encode fate identity directly. Instead, LEFTY expression alters the qualitative outcome of NODAL signaling by enforcing a transition from an "on" to an "off" state, which can then become permissive to a subsequent NODAL-dependent push. In this manner, NODAL instead reinforces discrete competency windows during which specific fate programs can be unlocked.

Thus, LEFTY does not refine a graded NODAL signal into multiple positional outputs, but rather acts as a critical regulator of signal termination, organizing cell fate acquisition by controlling when progenitor cells are released from NODAL-dependent plasticity.

2. Integration with prior literature:

Throughout the paper, discussion of previous models of Nodal-Lefty patterning in mouse and other systems is inadequate. As noted above, this makes it challenging to understand precisely what change to our understanding the authors are claiming to make. Specifically, points that should be more completely addressed are:

a. Addition of a thorough discussion of the authors proposed model in comparison to the Robertson lab's work on this topic (e.g. PMID: 12842913). This study concluded that Nodal patterning relied on interpretation of graded signals; do the authors wish to refute this model and establish a new

one? If they reject those findings, what is the basis? Do they view the data in that paper consistent or inconsistent with the findings here?

Thank you for the suggestion. We acknowledge the need for a more comprehensive discussion of previous Nodal patterning models in mice and the specific contexts in which these models were generated. Note that we do not intend to negate these models, merely to present continuously modeled scRNA-seq data to provide an explanation for the appearance of graded signal behavior. We also hope that the reviewer appreciates the challenges in addressing such a deeply studied signaling axis within the framework of a modern scientific report.

The study by Robertson's lab (Vincent, Dunn et al. 2003) compared cell fate decisions in the organizer among three different Nodal-related mutants. In epiblast-specific *Smad2* mutants and Nodal PEE enhancer mutants, anterior derivatives such as ADE (anterior definitive endoderm) and PCP (prechordal plate) were lost, while notochord formation remained unaffected. Furthermore, introducing a single *Smad3* mutant allele into a Smad2-deficient epiblast resulted in a complete loss of organizer derivatives. Based on these observations, the authors proposed a graded Nodal activity model, suggesting that different organizer derivatives require distinct levels of Nodal signaling—higher levels for anterior axial structures (ADE and PCP) and lower levels for the posterior axial structure (notochord).

However, this interpretation raises certain conceptual challenges. If the organizer represents a high-threshold state of Nodal signaling, it is unclear why it can still be specified—albeit at reduced levels—even in severe Nodal-decreased mutants. This suggests that an alternative model may better explain the data. Although our study does not specifically focus on organizer fate decisions, we propose that the organizer itself is initially in a Nodal-ON state, and its differentiation into PCP and ADE also occurs in a Nodal-ON context. In contrast, notochord formation requires a Nodal-OFF state, which could explain its later specification. This requirement for Nodal inhibition in notochord formation has also been supported by recent in vitro studies (Rito, Libby et al. 2025). In the *Smad2-/-; Smad3+/-* mutant, we propose that differentiation into the organizer is shortened because Nodal activity is rapidly downregulated, promoting caudalization. As a result, significantly fewer axial progenitors are generated. However, further investigation is needed to clarify how organizer specification is affected in this mutant, as this aspect was not directly addressed in the original study.

b. Several studies in mouse and other organisms have explored how loss of Lefty feedback impacts Nodal pattern formation and downstream cell fate acquisition. I think the paper would benefit from a dedicated section in the discussion that covers Lefty's role in zebrafish (PMID: 29215332, PMID: 12498687), xenopus (PMID: 16405884) mouse (PMID: 10518210) and gastruloid (PMID: 35079017) models, and explains how the findings of this paper confirm or revise the lessons and models generated by those studies. Does this study reveal something new about the effect of Lefty expression on Nodal signaling, or is it consistent with what we see in other organisms?

Thank you for your insightful suggestion. In response, we have added a dedicated section to the Discussion that comprehensively reviews *Lefty* mutant phenotypes and functions in zebrafish, *Xenopus*, mouse, and gastruloids. We have also explicitly outlined how our findings in the mouse model confirm or diverge from previous studies.

Specifically, we highlight that the overgeneration of organizer and mesodermal/primitive streak cells in mouse *Lefty* mutants is consistent with phenotypes observed in zebrafish, and *Xenopus* following *Lefty* loss. However, our study also reveals a key difference: in mice, these progenitor cells fail to further differentiate and embryos arrested before reaching the tailbud stage. Instead of contributing to definitive mesoderm and endoderm, as seen in other vertebrates, the excess *PS-like* cells in mouse embryos preferentially give rise to extraembryonic mesoderm. This suggests a species-specific role of *Lefty* in regulating cell fate potential and axial elongation, which may be related to differences in embryonic architecture among vertebrates. Additionally, we discuss a novel function of *Lefty1* in the mouse embryo. While *Lefty* proteins are generally considered *Nodal* antagonists that regulate signaling range and duration, our findings indicate that *Lefty1* also plays a role in AVE migration and primitive streak orientation, an aspect not previously described in other vertebrate models.

Please see below the included paragraph within our discussion:

"Our results clarify a number of classic reports into the function of this essential morphogen, and may explain the differences in phenotypes across model organisms. In particular, previous loss-of-function studies on Lefty/Antivin in non-amniote vertebrates have shown a significant expansion of the organizer and mesendoderm progenitors, leading to the enlargement of axial tissues and enhanced formation all mesoderm and endoderm without compromising the ability to generate tailbud-stage embryos (Cha, Takahashi, and Wright 2006; Zhang et al. 2019; Branford and Yost 2002; Agathon, Thisse, and Thisse 2001; Rogers et al. 2017). In mouse embryos, Lefty2 mutants exhibit an expansion of primitive streak and excessive mesoderm-like cell formation (Meno et al. 1999), which our work clarifies is largely explained by the extended differentiation of XPS-driven extraembryonic mesoderm and blood. Furthermore, although analysis of Lefty1 mutant in mice has established its role in left-right axis determination (Meno et al. 1998), its early function is considered to be redundant with another Nodal antagonist, Cer1, in AVE, and the role of LEFTY1 in the differentiation and function of this structure has been less clear (Perea-Gomez et al. 2002). Similar to previous studies (Trichas et al. 2011) (Takaoka, Nishimura, and Hamada 2017), we also observed the impaired anterior migration of expanded DVE cells as well as abnormal primitive streak orientation in Lefty1 mutants, suggesting a specific role of Lefty1 linked to its unique expression in the AVE—an aspect not observed in other model organisms. It has been proposed that rodents evolved a novel function for Lefty1 by acquiring new cis-regulatory elements (CREs) that enable AVE-specific expression, which may explain the adaptations necessary to modulate these conserved signaling cues within the spatially restricted confines of the gastrulating epiblast (Yashiro et al. 2000)."

In response to this comment and question (a), we have also expanded our discussion of dominant *Nodal-Lefty* regulation models, including the gradient model, integrator model, and our proposed sequential inhibitory wave model. We specifically address how the high diffusivity of *Lefty* challenges the current integrator model by showing that *Nodal* does not necessarily need to accumulate within a *Lefty*-defined time window to induce different cell fates. Instead, we propose that *Nodal* and *Lefty* function as toggles to rapidly promote distinct state transitions. Only when *Lefty* or other *Nodal* antagonists completely shut down *Nodal* activity in a progenitor cell can a fate switch occur, rather than it being determined solely by the duration of *Nodal* signaling or the level of *Nodal* activity within a given time window. Our model better aligns with the biophysical properties of the *Nodal-Lefty* system and helps explain why, in mice, the loss of *Nodal* antagonists results in a severe depletion of downstream differentiation products rather than a distorted gradient that shifts all cell fates posteriorly. Our model is not a complete departure from previous frameworks but rather an evolution that incorporates new experimental insights and reconciles discrepancies between vertebrate systems.

We believe these additions strengthen our manuscript and provide a clearer perspective on how our study builds upon and refines previous findings. Thank you for requesting them.

3. Computational pipeline:

I believe that this study would be of interest to traditional embryologists, however the level of technical description is likely to reduce accessibility to this audience. I would regard myself as a quantitatively literate developmental biologist— but not a transcriptomics expert— and I found the main flow of the technical arguments hard to follow. I would suggest the following:

a. It would be helpful to add a user-friendly cartoon of the computational analysis pipeline that helps non-experts understand what is going on. For example, could the concept of metacells be intuitively illustrated? What, at a 'gut level' is GeoSeq doing? I leave this issue to the authors discretion, however I do believe that the reach of the paper could be substantially increased by making the technical side more accessible.

Thank you for your valuable suggestion. We have added a user-friendly workflow of the computational analysis pipeline below to improve accessibility for non-experts and are including this as a supplementary figure (**Extended Data Fig. 1a**). See below:

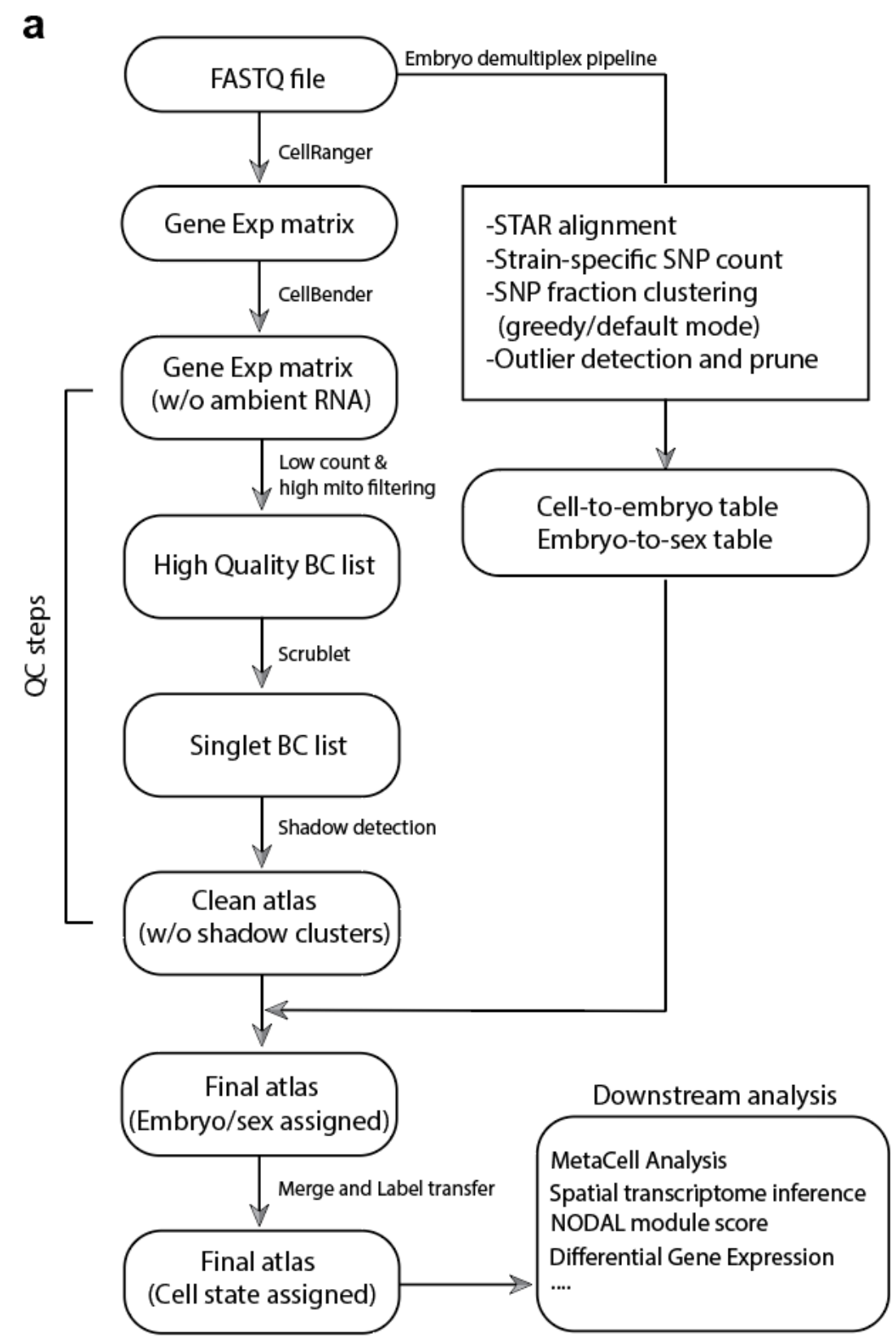

Revised Extended Data Figure 1a. Overview of the computational pipeline used in this study, highlighting key steps including embryo demultiplexing by genotype using the CAST SNP profile, correction for ambient RNA contamination, removal of shadow clusters, and cell-state label transfer.

In our study, we performed single-cell RNA sequencing on dissociated cells from 95 embryos spanning embryonic days (E) 6.5 to 9.0, which are sequenced in batches and resolved by haplotype information. We used haplotype information detected in the sequencing reads to demultiplex the data at the embryo level, allowing us to obtain sufficient biological replicates and capture natural variations among litters. We then generated a combined WT atlas and annotated 57 distinct cell states. To externally validate the spatial localization of these states, we projected them onto published Geo-seq data (Wang, Yang et al. 2023) using CIBERSORTx (Newman, Steen et al. 2019). Geo-seq is derived from low-input RNA sequencing of laser dissected regions of mouse embryos, enabling spatially resolved transcriptomic analysis (Peng, Suo et al. 2016, Peng, Suo et al. 2019, Wang, Yang et al. 2023). This dataset employs a specialized “corn plot” to visualize gene expression patterns across different embryonic regions. CIBERSORTx is a machine-learning-based method that infers cell-type composition from bulk RNA-seq data using a reference scRNA-seq dataset. By integrating our single-cell data with the Geo-seq dataset via CIBERSORTx, we

could "project" our identified cell states onto the spatial embeddings provided by Geo-seq, enhancing spatial interpretation.

To construct a continuous differentiation map, we performed metacell analysis per batch on the entire dataset. This method aggregates transcriptionally similar single cells into more coherent and distinct transcriptional states, termed metacells (MCs). Using a graph-based algorithm, metacells partition single-cell profiles into statistically equivalent groups that, in theory, represent cells derived from the same RNA pool. This approach effectively minimizes the intrinsic sampling noise of single-cell data while preserving biological variance within each cell state.

Finally, to resolve the single cell level activity of SMAD2/3 signaling, we leverage bulk SMAD2/3 ChIP-seq and RNA-seq data from SB-431542 or ACTIVN-treated mouse embryoid bodies to identify an experimentally validated set of NODAL-responsive genes, after which we examined the relative activity and dynamics within single cells across our gastrulation. We inferred a single NODAL score by calculating average expression of the same set of SMAD2/3 targets against background features to infer the relative variation within single cells. We further partitioned these target genes into distinct "modules" using Nonnegative Matrix Factorization (NMF), a method for decomposing complex signals into distinct local "programs." This method allows us to describe the specific genes that are active in different patterns throughout our gastrulation reference."

We believe that these additions and clarifications will make the technical aspects of our study more accessible to a broader developmental biology audience.

b. Inferring the Nodal signaling activity through the Nodal score is an interesting concept. However, I don't think the procedure was well explained given the centrality to the paper's argument. I was unable to locate a description of its calculation in the Methods section; as far as I can tell it only appears in the legend of Extended Data Figure 3. An extended discussion of how it was calculated would be helpful.

Thank you for the suggestion. The steps used to calculate the Nodal score have been updated and added to the Methods section "**Inferring NODAL target genes from re-analyzing ChIP-seq data and RNA-seq data**" and "**Non-negative Matrix Factorization (NMF) of NODAL module**".

c. If I understand correctly from Extended Data Figure 3: the Nodal score for each cell is simply the weight of PC1 from the PCA? If this is true, I think the rationale needs to be extended and defended in the methods. I see that several Nodal targets are loaded significantly in PC1, but presumably it could also represent the action of other biological processes? Why use PC1 rather than a weighted sum of known Nodal target genes? Given that PC1 explains the most variance in

the data of any PC, I am concerned that this measure represents some other large and complex trend in the data (for example, developmental time or a combination of signaling pathways?).

We thank the reviewer for this question and have revised our analysis accordingly. Within the first submission, the NODAL score was derived from the embeddings along the first principal component (PC1), rather than from gene loadings or weights. We acknowledge that the contribution of other signaling pathways cannot be entirely excluded, as a subset of target genes is shared across pathways. Importantly, our goal was not to demonstrate complete pathway segregation, but rather to capture relative enrichment of NODAL signaling activity across cell populations.

To further refine NODAL-responsive states and reduce potential bias introduced by reliance on a single dominant variance direction, we subsequently employed non-negative matrix factorization (NMF) in our latest analysis. NMF decomposes gene expression data into a set of additive, non-negative components, enabling identification of coherent gene modules and their corresponding cell-level embeddings. We used these embeddings as module activity scores, which better capture complex, multi-modal patterns of NODAL target gene expression beyond what is accessible through PC1 alone. Please see **Figure 5** for this refined analysis, which we further leverage to identify cell state specific transcriptional interactions between SMAD2/3 targets and lineage specific transcription factors.

Although it is no longer in the text, our rationale for using PC1 was that it represents the direction of maximal coordinated variance among the selected NODAL target genes. This enables identification of cell populations exhibiting excess coordinated variability—i.e., overdispersion—within this gene set, a property expected for cells actively responding to NODAL signaling. Thus, PC1 provides an effective summary of collective NODAL target gene behavior at the single-cell level. To assess pathway specificity, we applied the same strategy to BMP and WNT target genes (see below **Reviewer Figure**). Although substantial overlap exists between NODAL and WNT targets in epiblast cells, the resulting PC1 score distributions were distinct, indicating divergent activity patterns and supporting the utility of PC1 embeddings for capturing pathway-specific signaling dynamics.

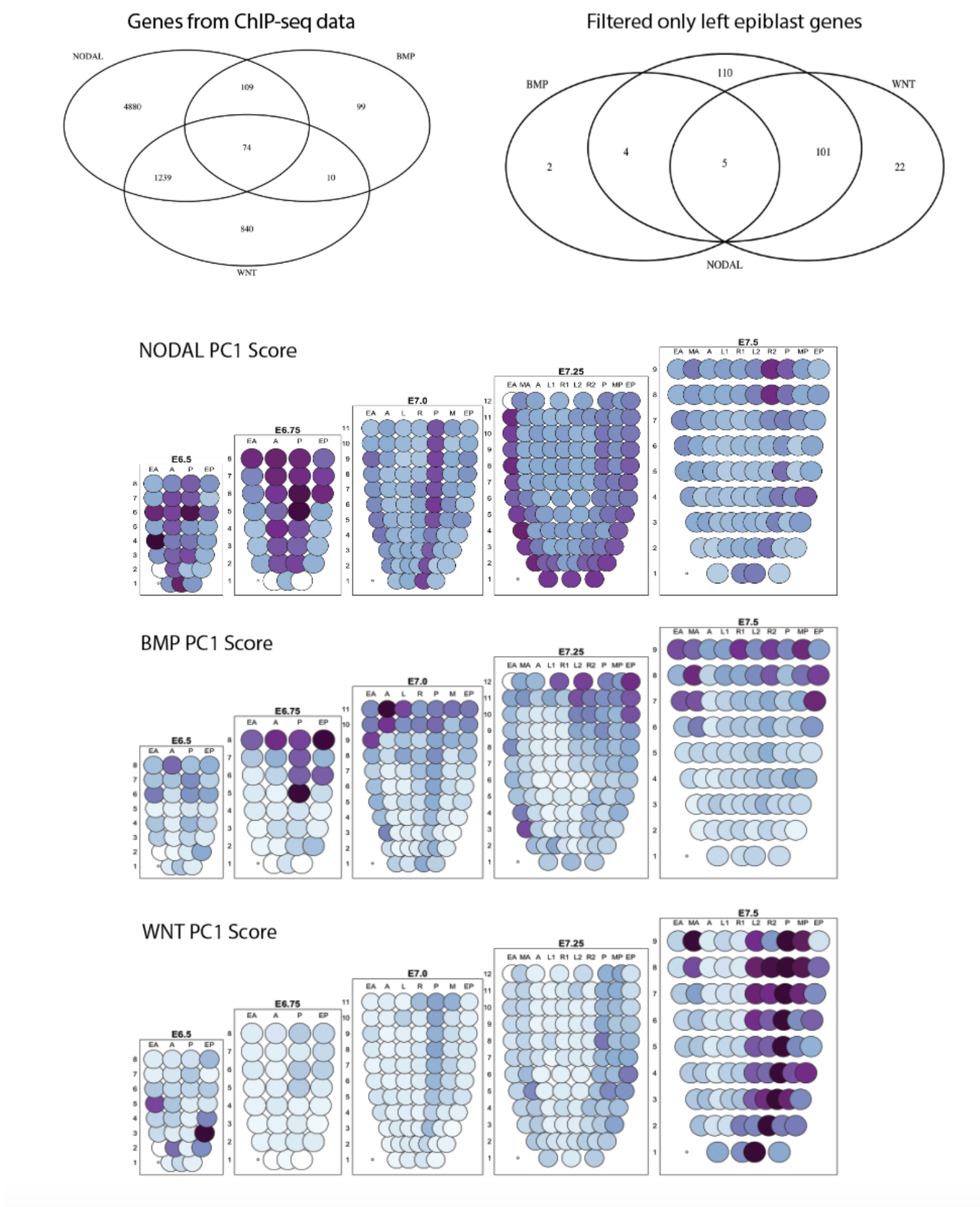

Reviewer Figure 1. Inference of additional signal transduction pathway timing and localization by phasing ChIP-seq information in single cells. Briefly, ChIP-seq data for BMP and WNT were taken from GSE70581 and GSE162774 respectively and analyzed as for SMAD2/3 binding as conducted before. Then, the relative PC1 score of these targets were projected onto the GEO-seq data as conducted for distinct NMF modules as shown in Extended Data Fig. 8a. Notably, the dynamics of WNT signal transduction are notably delayed in comparison to NODAL and localized within the PS proper. These data would support the notion that NODAL signaling modulation precedes WNT-based licensing of definitive mesoderm forming primitive streak. Moreover, it highlights the reasons why these cell states are not induced in Lefty KO cells, as PS differentiation requires LEFTY-based attenuation of earlier NODAL waves.

4. Optional: supporting measurements of Nodal signaling activity

To my eye, the most surprising output of the computational measurements was the transient cessation of Nodal signaling activity in E 7.0 embryos (e.g. Fig. 2a). From measurements of the expression of NODAL and LEFTY genes in the literature, one would expect signaling activity to begin throughout the entire embryo (as shown in Fig. 2a and f) and monotonically become

restricted to the posterior side and primitive streak. Instead, the authors seem to observe a transient shut down in Nodal signaling before a second wave of activity. This would be a really neat prediction to test experimentally; I think it would be as simple as a short time course of a-pSmad2 immunostainings over the relevant stages. If the transient 'stop' in Nodal signaling activity were observed, I think it would demonstrate the power of the transcriptomic measurements over the traditional approach of in situ visualization of gene expression.

We were very enthusiastic about testing this hypothesis but faced limitations in optimizing a reliable pSMAD2 protocol using published reagents and methods. Specifically, we observed uniformly weak nuclear staining across conditions, making it difficult to interpret dynamic changes in signaling activity. While we cannot exclude technical issues such as antibody performance—even though we used the same reagent as reported—this limitation prevented us from confidently resolving transient NODAL signaling dynamics using imaging.

Notably, this apparent "system-wide" modulation in NODAL activity is less apparent when we apply our distinct NMF modules to the same spatial data (see Extended Data Fig. 8a and below). There, we see what instead appears to be a transition in the activity of specific SMAD2/3 targets from those associated with the Epiblast to those associated with the Caudal Epiblast and Primitive streak. As these represent distinct targets, it is likely that this attenuation reflected a major transition in how NODAL signal transduction *interacts* with cell state, which our results still support does not happen in the absence of LEFTY Modulation. Notably, changes in SMAD2/3 binding or synergy would not be visibly apparent by IF for pSMADs, highlighting the value of single cell resolved transcriptional analysis.

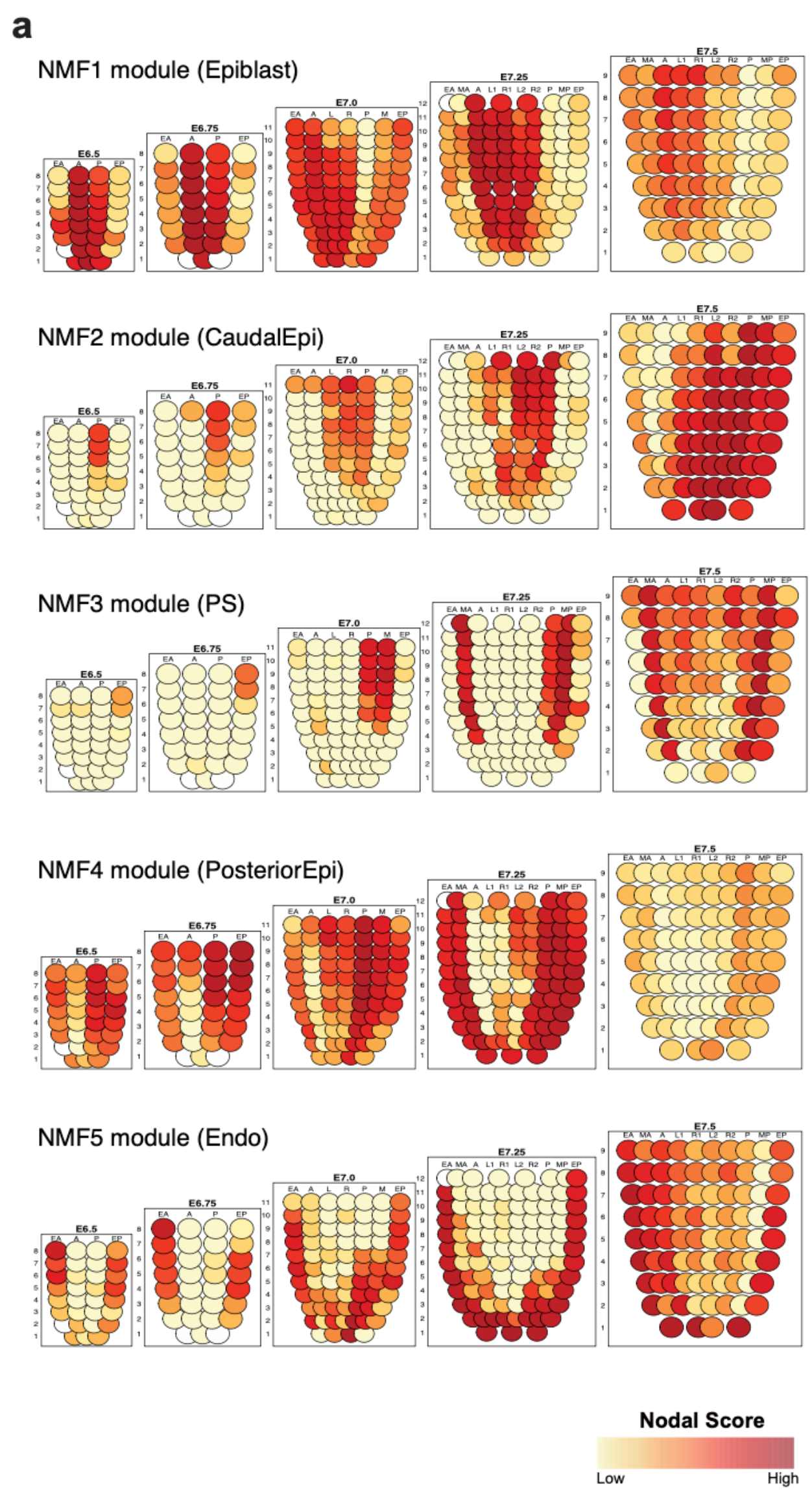

Revised Extended Data Fig. 8a. Corn plots showing aggregated expression levels (log10 TPM) of individual NODAL submodules in spatially resolved GEO-seq data from E6.5 to E7.5. Submodule gene composition and naming are shown in **Fig. 5g**. These data confirm the relative spatiotemporal dynamics of NODAL signal transduction during early gastrulation, including cell state specific differences in signal transduction.

We hope these comments and changes to our analysis and text satisfy the reviewers curiosity and very much appreciate their time and enthusiasm for the project and its findings. We believe it has substantially improved our work.

Reviewer3:

In this study Wang et al. dissect the role of Nodal signalling in patterning the mouse primitive streak. Using single-cell RNAseq of wild-type, and Nodal antagonist Lefty1 and Lefty2 CRISPR knockouts they track the signalling output of Nodal signalling at different stages in different cells fates during mouse gastrulation and the consequence of Nodal signalling perturbation. The scale of work in terms of the spatial and temporal resolution is impressive, providing novel insights into Lefty-controlled waves of Nodal activity in the streak.

However, my major concern is that this study overlooks the primary AVE migration phenotype of Lefty1 mutants. This is in part due to neglecting previous papers on Lefty1 knockouts, and in part due to the loss of spatial information in the single-cell RNAseq methodology used to analyse these mutants. As a consequence, the analysis and conclusions of the Lefty1 mutant phenotype are oversimplified.

We thank the reviewer for their positive comments about the scale and scope of our work and have done our best to address their specific comments below, including a description of molecular signatures that distinguish control from KO AVE/DVE as well as spatial validation of phenotypes at the distal tip of the embryo.

Major Points:

Nodal score: The Nodal score / responsive gene list was derived from ChIP-Seq/RNAseq done in embryoid bodies, containing only epiblast derived lineages. Does this neglect Nodal responsive genes that may be specific to the extra-embryonic lineages? What about the Nodal response in the extra-embryonic ectoderm, and visceral endoderm, is this fully captured in the model?

Yes, the dataset we used is derived from embryoid bodies and therefore may not fully capture NODAL activity within extraembryonic lineages, such as the extraembryonic endoderm. However, it is important to note that many genes enriched in the extraembryonic endoderm, including *Amot*, *Foxa2*, and *Cer1*, are represented among the inferred NODAL target genes due to the high degree of transcriptional similarity with the early DE. Consistent with this, visceral endoderm states also display high inferred NODAL activity as inferred by our analysis. We have also added the following statement to the text to clarify this possible limitation:

"Notably, although our analysis showcases the ability to phase distinct transcriptional responses downstream of bulk ChIP-seq data, it remains possible that the use of EB-derived data may lose resolution for the extraembryonic tissues, which are not produced within this system but also respond to NODAL signaling (Murry and Keller 2008)."

For the extraembryonic ectoderm, our analysis revealed low NODAL activity, which may be attributable to limitations of the EB-based NODAL target assessment, and the loss of *Lefty* also appears to have little effect on its differentiation. Therefore, we tend to propose that

extraembryonic ectoderm is robust to prolonged NODAL activity and/or alternatively requires NODAL signal at the onset of gastrulation. Notably, in *Nodal* null mutants the ExE-Epiblast boundary is lost, and ExE seems collapsed (Conlon, Lyons et al. 1994, Ben-Haim, Lu et al. 2006). It has been reported that the unprocessed pro-NODAL is necessary to maintain BMP expression in the ExE via Activin receptors (Ben-Haim, Lu et al. 2006), a mechanism that may explain how Nodal regulates extraembryonic ectoderm specification.

For the extraembryonic endoderm, we captured a substantial set of NODAL target genes that are also expressed in the visceral endoderm (VE), and accordingly the VE displays a highly NODAL-responsive state in our analysis. We further examined transcriptional differences within the VE between control and KO embryos, finding that, compared with early epiblast states, the VE and DE exhibit greater transcriptome divergence (see Extended Data Fig. 3e). In this comparsion we observed a substantial overlap of differentiaonally up-regulated genes between epiblast and VE, most of them are NODAL targets. Nevertheless, deeper analysis into the VE conforms with the reviewers comments and we have highlighted it specifically below:

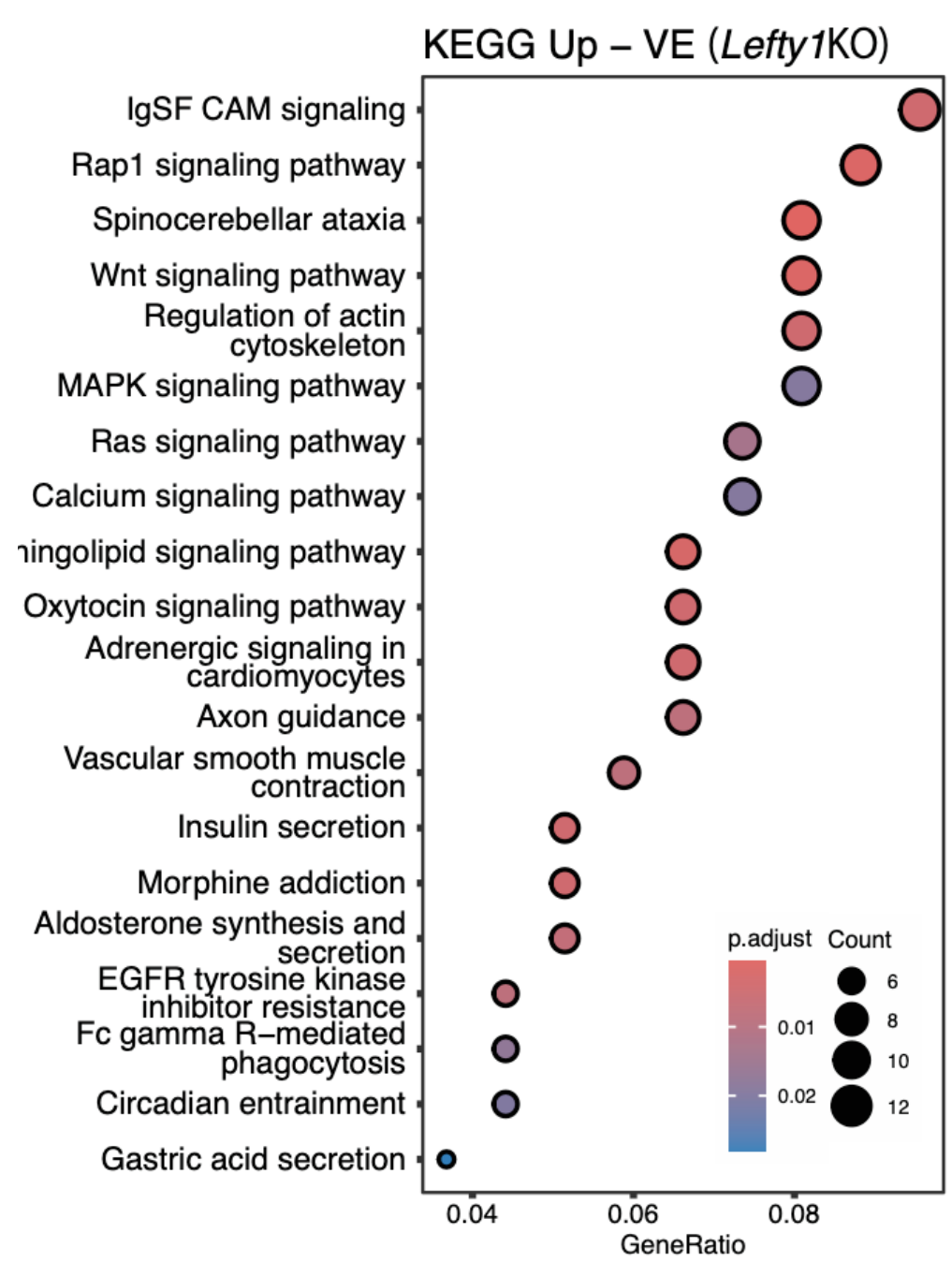

Revised Extended Data Fig. 3g. GO analysis of upregulated genes in VE between *Lefty1*KO and WT includes categories related to cell migration, polarity, and morphology, as indicated by enrichment of pathways related to IgSF CAM and Rap1 signaling, actin cytoskeleton organization, and Ras signaling.

Revised Text: "Notably, Lefty1KO VE cells showed upregulation of genes associated with cell migration, polarity, and morphology, with significant stronger pathways enrichment in IgSF CAM signaling, Rap1 signaling, actin cytoskeleton organization, and Ras signaling than observed in

Lefty2KO (**Extended Data Fig. 3g**). These transcriptional changes may provide a mechanistic explanation for the DVE migration observed in Lefty1 mutant embryos (Trichas et al. 2011)."

- Can the authors validate their computational Nodal response output with pSmad2 staining to see the degree of overlap? I would recommend Brumm et al 2025 Developmental Cell https://doi.org/10.1016/j.devcel.2024.10.020 protocol for the best pSmad2 staining I've seen in gastrulating mouse embryos.

We were very interested in performing this experiment. One of the main challenges lies in defining the appropriate temporal resolution, as any transient change of NODAL signaling is likely to be brief and confined to a narrow developmental window. In addition, quantitatively assessing signaling activity using imaging-based approaches remains technically challenging and does not capture changes in NODAL signaling that reflect a repurposing of targets versus complete cessation.

We indeed have attempted to reproduce the same protocol using the reported antibody. However, in our hands, the staining quality was suboptimal, with nuclear signals appearing uniformly dim and lacking sufficient contrast for quantitative interpretation. While we cannot exclude the possibility that further optimization is required, including antibody performance or protocol modifications, these technical limitations currently prevent us from drawing reliable conclusions from this approach.

However, as discussed for Reviewer 2, we have refined our NODAL score analysis to reflect distinct expression modules that act downstream of SMAD2/3 signaling as predicted from EB-derived ChIP-seq. There we see a pattern more consistent with a re-distribution of SMAD2/3 target gene expression, that nonetheless requires LEFTY1/2 activity to support. In this manner, our transcriptional read out is more sensitive that pSMAD2 IF, in that it directly addresses the role of LEFTY1/2 in supporting transient signal suppression to support the induction of downstream genes.

Lefty1 knock-out:

- "Although no discernible morphological differences are observed between mutant and WT embryos at ~E6.5, disparities between LeftyKOs become more apparent after E7.5". The authors conclude from their Lefty1 knockout-results that Lefty1 antagonism of Nodal is the main driver of the phenotype A-P patterning and differentiation defects seen in mutant embryos at E7.5. However, the discussion and analysis overlook the primary phenotype of Lefty1 mutants: defects in AVE migration (Trichas et. al., 2011 PLOS Biology https://doi.org/10.1371/journal.pbio.1001019). Lefty1 mutants can be classified at E6.25 as having AVE over-migration, or DVE/AVE migration arrest. The work of Trichas et al. should be referred to in the paper.

We apologize for this omission, Trichas et al. is now discussed and cited several times throughout the text (see below).

"Lefty1 mutant embryos appear to display more severe phenotypes than Lefty2 mutants, including a more pronounced loss of anteroposterior polarity and greater variability in epiblast morphology early during gastrulation, a phenotype that may reflect earlier effects on the migration of the AVE/DVE (Trichas, Joyce et al. 2011) **(Extended Data Fig. 2c, d)**."

"This orientation defect is also supported by the inappropriate enrichment of anterior EOMES+ cells and improper localization of Cer1+ cells to the distal region of Lefty1 mutant embryos (**Extended Data Fig. 5a**), which recapitulates the DVE migration defects found in Lefty1 mutants (Trichas, Joyce et al. 2011)"

" These transcriptional changes may provide a mechanistic explanation for the DVE migration observed in Lefty1 mutant embryos (Trichas, Joyce et al. 2011)."

" Similar to previous studies (Trichas, Joyce et al. 2011) (Takaoka, Nishimura et al. 2017), we also observed the impaired anterior migration of expanded DVE cells as well as abnormal primitive streak orientation in Lefty1 mutants, suggesting a specific role of Lefty1 linked to its unique expression in the AVE—an aspect not observed in other model organisms."

- It appears from the images in the extended data figures (Ext. data 4e II and Ext data 7b) that Lefty1KO E7.5 embryos exhibit thickened distal regions, and distally localised Cer1, indicative of a DVE/AVE migration arrest. This is further supported by failure in A-P rotation and proximally localised primitive streak, marked by Brachyury (Fig. 5b). Therefore, this study's Lefty1KO phenotype recapitulates the one described in Trichas et. al., and warrants further analysis at earlier stages to identify the onset and mechanism of this mutant phenotype.

Thank you for this suggestion, we have now clarified the differences in primitive streak phenotype more carefully and highlighted the extent to which *Lefty1* vs *Lefty2* KOs display A/P patterning defects. In particular, we highlight the severity of this phenotype in three places:

"*Lefty1* mutant embryos appear to display more severe phenotypes than *Lefty2* mutants, including a more pronounced loss of anteroposterior polarity and greater variability in epiblast morphology early during gastrulation, a phenotype that may reflect earlier effects on the migration of the AVE/DVE (Trichas, Joyce et al. 2011) **(Extended Data Fig. 2c,d)**."

"Notably, Lefty1KO VE cells showed upregulation of genes associated with cell migration, polarity, and morphology, with significant stronger pathways enrichment in IgSF CAM signaling, Rap1 signaling, actin cytoskeleton organization, and Ras signaling than observed in Lefty2KO

(**Extended Data Fig. 3g**). These transcriptional changes may provide a mechanistic explanation for the DVE migration observed in Lefty1 mutant embryos (Trichas et al. 2011)."

"It has been proposed that rodents evolved a novel function for Lefty1 by acquiring new cis-regulatory elements (CREs) that enable AVE-specific expression, which may explain the adaptations necessary to modulate these conserved signaling cues within the spatially restricted confines of the gastrulating epiblast (Yashiro, Saijoh et al. 2000)."

- Given that there are Lefty1KO AVE defects at E6.5 it raises the question of whether the defects in epiblast patterning seen at E7.5 are caused by (i) only the loss of Lefty1 antagonism of Nodal as concluded in this paper or (ii) failure of proper AVE migration, influencing antagonism of multiple signalling pathways (e.g. AVE-secreted Cer1 on BMP, and DKK1 on WNT) in addition to the loss of antagonism of Lefty1 on Nodal

The orientation and formation of primitive streak seem to accompany the migration defects of AVE and are closely related to the regulators of WNT, NODAL and BMP signaling pathways. For instance, In *Wnt3* or *β-catenin* mutants, although the AVE is correctly formed and migrated, the primitive streak is not formed (Liu, Wakamiya et al. 1999, Huelsken, Vogel et al. 2000), which indicates that the mechanism that necessary for AVE migration is not sufficient for PS formation. In *Cripto*, *Otx2* or *Bmpr1a* mutants, AVE migration is defective, and markers of the primitive streak are either absent or localized to the proximal side (Ding, Yang et al. 1998, Perea-Gomez, Lawson et al. 2001, Miura, Singh et al. 2010), suggesting that migration of AVE may direct the orientation and maintenance of primitive streak. However, how the failure of AVE migration contributes to the defect of mesoderm specification remains unclear. In some cases, such as *Foxa2* mutant, although AVE migration is defective, some embryos recover well with somite formation, though they are fused due to the lost the notochord (Ang and Rossant 1994, Yang and Klingensmith 2006). Given the high similarity in state outcomes between *Lefty1* and *Lefty2* mutants observed in our manuscript, we therefore favor interpretation (i), although we cannot fully rule out the possibility of (ii).

With respect to the loss of definitive mesoderm differentiation, we propose that this phenotype is primarily driven by elevated and persistent NODAL activity, rather than by defects in AVE-derived patterning cues. This interpretation is supported by the observation that *Lefty2* mutants—despite lacking overt AVE migration defects—still exhibit a pronounced loss of definitive mesoderm.

To directly test whether excessive NODAL signaling underlies this differentiation failure, we performed roller-culture rescue experiments using *Lefty2* mutant embryos. E8.5 *Lefty2* mutants consistently displayed epiblast arrest prior to culture. Embryos were then roller-cultured for 24 hours in 50% rat serum, either in the presence of the NODAL inhibitor SB431542 (10 μM) or under control conditions. Single-cell transcriptomic profiling revealed that global inhibition of

NODAL signaling—rather than suppression of a specific AVE-derived source—promotes differentiation toward definitive mesoderm derivatives.

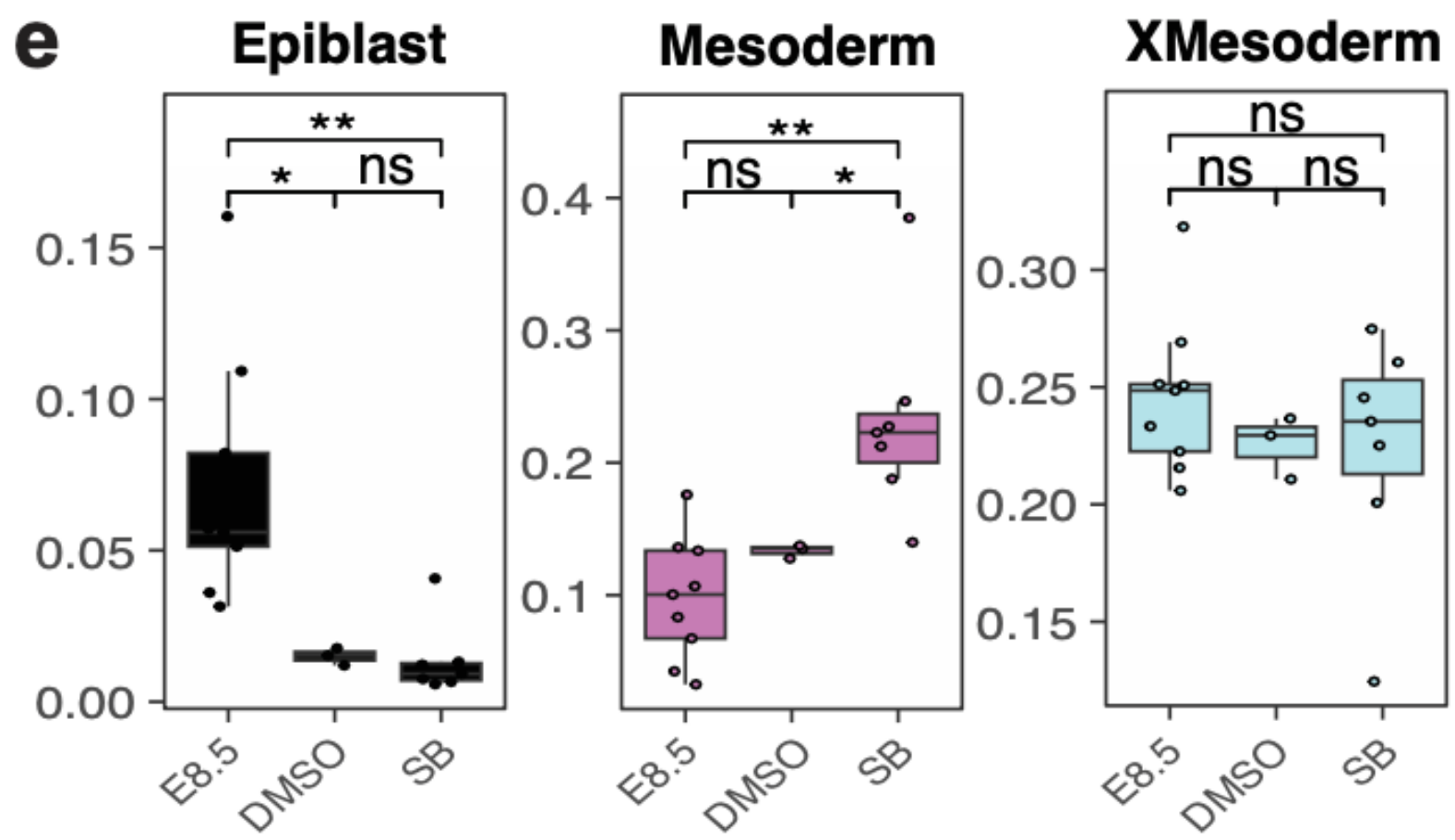

Revised Figure 3e. Per-embryo proportions of epiblast, mesoderm, and extraembryonic mesoderm (XMesoderm) germ layers, with each dot representing an individual embryo from E8.5 *Lefty2*KO, DMSO control, or SB431542 treatment groups. *Lefty2*KO embryos cultured with SB431542 exhibit increased mesoderm differentiation, while no significant change is observed in extraembryonic mesoderm differentiation. This change is apparent across multiple distinct definitive mesoderm states (see **Extended Data Fig. 6**). Statistical significance was evaluated using the Wilcoxon rank-sum test, with *P < 0.05, **P < 0.01, and ***P < 0.001.

Together, these results indicate that sustained NODAL activity is sufficient to block definitive mesoderm differentiation, providing functional support for interpretation (i), while leaving open the possibility that additional early patterning effects may also contribute to the overall phenotype.

- The cause of AVE migration defects in Lefty1KO is unclear. The single-cell data set in this study offers a great opportunity to address this question and understand the driving cause of the phenotypes seen in Lefty1 mutants. Can differences in the visceral endoderm of WT vs Lefty1KO at E6.5 be detected? A pre-print (Stower et al. 2023 https://doi.org/10.1101/2023.03.31.534937) suggests that Lefty1-dependent mechanical heterogeneity in the EmVE and ExVE is the primary cause for AVE migration defects in Lefty1KO embryos, originating as early as E5.5. It would be interesting to know if this can be seen at a transcript level.

In the revised manuscript, we performed differential gene expression analysis on all visceral endoderm (VE) states combined, including EmVE, ExVE, and AVE. This analysis identified gene sets with significantly stronger enrichment for pathways related to IgSF CAM signaling, Rap1 signaling, actin cytoskeleton organization, and Ras signaling, which may partly explain the mechanical heterogeneity in visceral endoderm, as discussed prior to the reviewer's question. Given that our single-cell dataset previously defined AVE&DVE state, we also assessed differential gene expression within the AVE&DVE state in WT and *Lefty1*KO embryos, and the

results are attached here. Our analysis did not reveal significant transcriptomic changes (defined as log(fold change) > 2) between these conditions. However, we observed a slight upregulation (log(fold change)>0.5) of genes associated with oxidation and energy metabolism, suggesting that cellular dynamics and energy regulation may contribute to the proper migration of the AVE population.

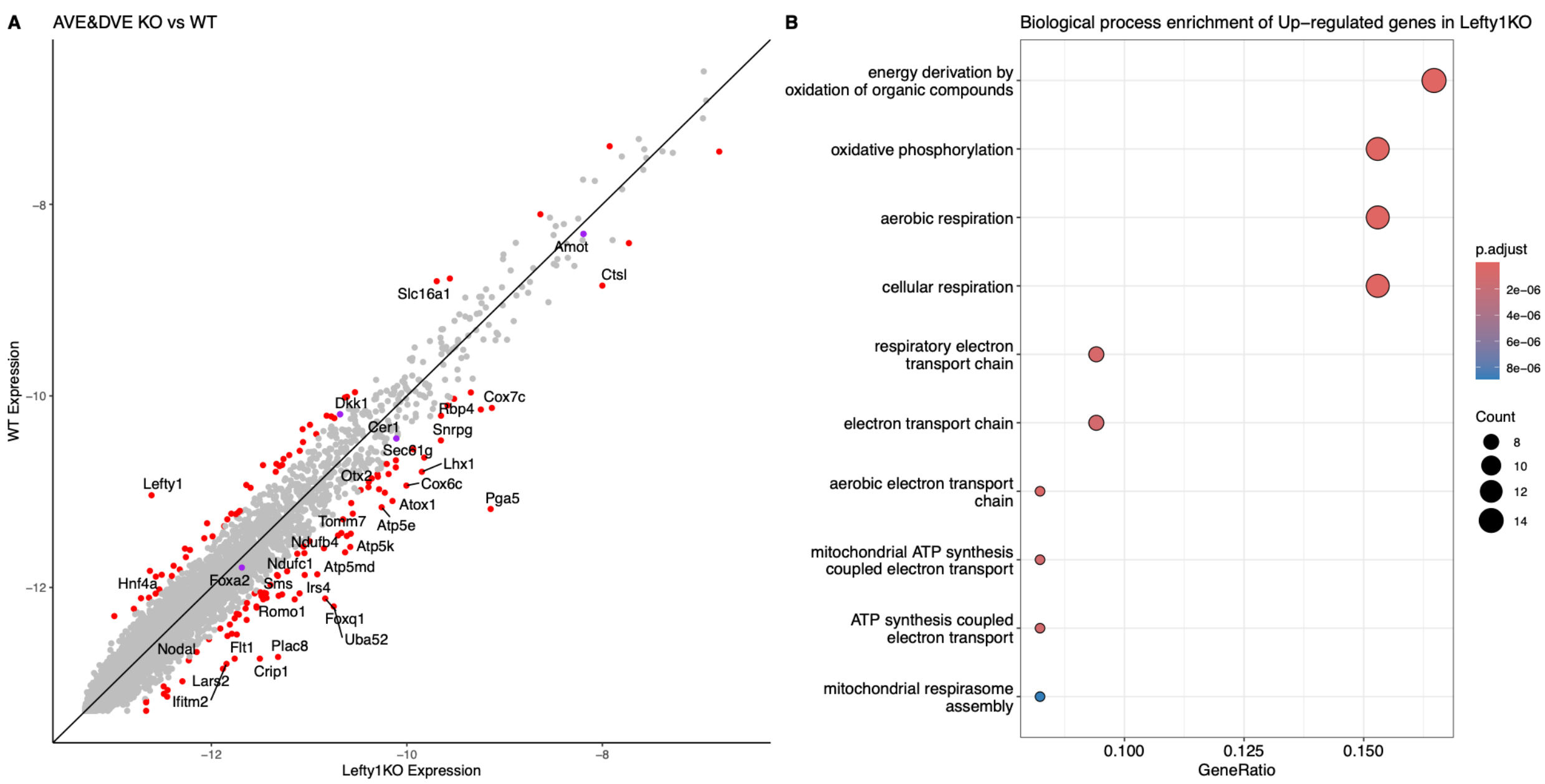

Reviewer Figure 2. Gene expression differences associated with AVE/DVE in Lefty1 KO compared to Control. Although here transcriptional differences are mostly associated with changes in metabolic state, full analysis of the entire VE (Extended Data **Fig 3f and g**) show downregulation of a greater repertoire of genes associated with cell migration, polarity, and morphology, with significant stronger pathways enrichment in IgSF CAM signaling, Rap1 signaling, actin cytoskeleton organization, and Ras signaling in *Lefty1*KO vs *Lefty2*KO.

We hope this satisfies the Reviewers curiosity about the VE in Lefty mutant embryos and thank them again for their comments, which we believe have significantly improved our manuscript.

References:

Agathon, A., B. Thisse and C. Thisse (2001). "Morpholino knock-down of antivin1 and antivin2 upregulates nodal signaling." Genesis **30**(3): 178-182.

Ang, S. L. and J. Rossant (1994). "HNF-3 beta is essential for node and notochord formation in mouse development." Cell **78**(4): 561-574.

Ben-Haim, N., C. Lu, M. Guzman-Ayala, L. Pescatore, D. Mesnard, M. Bischofberger, F. Naef, E. J. Robertson and D. B. Constam (2006). "The nodal precursor acting via activin receptors induces mesoderm by maintaining a source of its convertases and BMP4." Dev Cell **11**(3): 313-323.

Branford, W. W. and H. J. Yost (2002). "Lefty-dependent inhibition of Nodal- and Wnt-responsive organizer gene expression is essential for normal gastrulation." Curr Biol **12**(24): 2136-2141.

Cha, Y. R., S. Takahashi and C. V. Wright (2006). "Cooperative non-cell and cell autonomous regulation of Nodal gene expression and signaling by Lefty/Antivin and Brachyury in Xenopus." Dev Biol **290**(2): 246-264.

Chen, Y. and A. F. Schier (2002). "Lefty proteins are long-range inhibitors of squint-mediated nodal signaling." Curr Biol **12**(24): 2124-2128.

Conlon, F. L., K. M. Lyons, N. Takaesu, K. S. Barth, A. Kispert, B. Herrmann and E. J. Robertson (1994). "A primary requirement for nodal in the formation and maintenance of the primitive streak in the mouse." Development **120**(7): 1919-1928.

Ding, J., L. Yang, Y. T. Yan, A. Chen, N. Desai, A. Wynshaw-Boris and M. M. Shen (1998). "Cripto is required for correct orientation of the anterior-posterior axis in the mouse embryo." Nature **395**(6703): 702-707.

Green, J. B. and J. C. Smith (1991). "Growth factors as morphogens: do gradients and thresholds establish body plan?" Trends Genet **7**(8): 245-250.

Hagos, E. G. and S. T. Dougan (2007). "Time-dependent patterning of the mesoderm and endoderm by Nodal signals in zebrafish." BMC Dev Biol **7**: 22.

Huelsken, J., R. Vogel, V. Brinkmann, B. Erdmann, C. Birchmeier and W. Birchmeier (2000). "Requirement for beta-catenin in anterior-posterior axis formation in mice." J Cell Biol **148**(3): 567-578.

Liu, L., A. Nemashkalo, L. Rezende, J. Y. Jung, S. Chhabra, M. C. Guerra, I. Heemskerk and A. Warmflash (2022). "Nodal is a short-range morphogen with activity that spreads through a relay mechanism in human gastruloids." Nat Commun **13**(1): 497.

Liu, P., M. Wakamiya, M. J. Shea, U. Albrecht, R. R. Behringer and A. Bradley (1999). "Requirement for Wnt3 in vertebrate axis formation." Nat Genet **22**(4): 361-365.

Meno, C., K. Gritsman, S. Ohishi, Y. Ohfuji, E. Heckscher, K. Mochida, A. Shimono, H. Kondoh, W. S. Talbot, E. J. Robertson, A. F. Schier and H. Hamada (1999). "Mouse Lefty2 and zebrafish antivin are feedback inhibitors of nodal signaling during vertebrate gastrulation." Mol Cell **4**(3): 287-298.

Meno, C., A. Shimono, Y. Saijoh, K. Yashiro, K. Mochida, S. Ohishi, S. Noji, H. Kondoh and H. Hamada (1998). "lefty-1 is required for left-right determination as a regulator of lefty-2 and nodal." Cell **94**(3): 287-297.

Miura, S., A. P. Singh and Y. Mishina (2010). "Bmpr1a is required for proper migration of the AVE through regulation of Dkk1 expression in the pre-streak mouse embryo." Dev Biol **341**(1): 246-254.

Murry, C. E. and G. Keller (2008). "Differentiation of embryonic stem cells to clinically relevant populations: lessons from embryonic development." Cell **132**(4): 661-680.

Newman, A. M., C. B. Steen, C. L. Liu, A. J. Gentles, A. A. Chaudhuri, F. Scherer, M. S. Khodadoust, M. S. Esfahani, B. A. Luca, D. Steiner, M. Diehn and A. A. Alizadeh (2019). "Determining cell type abundance and expression from bulk tissues with digital cytometry." Nat Biotechnol **37**(7): 773-782.
Peng, G., S. Suo, J. Chen, W. Chen, C. Liu, F. Yu, R. Wang, S. Chen, N. Sun, G. Cui, L. Song, P. P. Tam, J. D. Han and N. Jing (2016). "Spatial Transcriptome for the Molecular Annotation of Lineage Fates and Cell Identity in Mid-gastrula Mouse Embryo." Dev Cell **36**(6): 681-697.
Peng, G., S. Suo, G. Cui, F. Yu, R. Wang, J. Chen, S. Chen, Z. Liu, G. Chen, Y. Qian, P. P. L. Tam, J. J. Han and N. Jing (2019). "Molecular architecture of lineage allocation and tissue organization in early mouse embryo." Nature **572**(7770): 528-532.
Perea-Gomez, A., K. A. Lawson, M. Rhinn, L. Zakin, P. Brulet, S. Mazan and S. L. Ang (2001). "Otx2 is required for visceral endoderm movement and for the restriction of posterior signals in the epiblast of the mouse embryo." Development **128**(5): 753-765.
Perea-Gomez, A., F. D. Vella, W. Shawlot, M. Oulad-Abdelghani, C. Chazaud, C. Meno, V. Pfister, L. Chen, E. Robertson, H. Hamada, R. R. Behringer and S. L. Ang (2002). "Nodal antagonists in the anterior visceral endoderm prevent the formation of multiple primitive streaks." Dev Cell **3**(5): 745-756.
Rito, T., A. R. G. Libby, M. Demuth, M. C. Domart, J. Cornwall-Scoones and J. Briscoe (2025). "Timely TGFbeta signalling inhibition induces notochord." Nature **637**(8046): 673-682.
Rogers, K. W., N. D. Lord, J. A. Gagnon, A. Pauli, S. Zimmerman, D. C. Aksel, D. Reyon, S. Q. Tsai, J. K. Joung and A. F. Schier (2017). "Nodal patterning without Lefty inhibitory feedback is functional but fragile." Elife **6**.
Sakuma, R., Y. Ohnishi Yi, C. Meno, H. Fujii, H. Juan, J. Takeuchi, T. Ogura, E. Li, K. Miyazono and H. Hamada (2002). "Inhibition of Nodal signalling by Lefty mediated through interaction with common receptors and efficient diffusion." Genes Cells **7**(4): 401-412.
Shen, M. M. (2007). "Nodal signaling: developmental roles and regulation." Development **134**(6): 1023-1034.
Snow, M. H. L. (1977). "Gastrulation in the mouse : Growth and regionalization of the epiblast." Development **42**(1): 293-303.
Takaoka, K., H. Nishimura and H. Hamada (2017). "Both Nodal signalling and stochasticity select for prospective distal visceral endoderm in mouse embryos." Nat Commun **8**(1): 1492.
Trichas, G., B. Joyce, L. A. Crompton, V. Wilkins, M. Clements, M. Tada, T. A. Rodriguez and S. Srinivas (2011). "Nodal dependent differential localisation of dishevelled-2 demarcates regions of differing cell behaviour in the visceral endoderm." PLoS Biol **9**(2): e1001019.
van Boxtel, A. L., J. E. Chesebro, C. Heliot, M. C. Ramel, R. K. Stone and C. S. Hill (2015). "A Temporal Window for Signal Activation Dictates the Dimensions of a Nodal Signaling Domain." Dev Cell **35**(2): 175-185.
Vincent, S. D., N. R. Dunn, S. Hayashi, D. P. Norris and E. J. Robertson (2003). "Cell fate decisions within the mouse organizer are governed by graded Nodal signals." Genes Dev **17**(13): 1646-1662.
Wang, R., X. Yang, J. Chen, L. Zhang, J. A. Griffiths, G. Cui, Y. Chen, Y. Qian, G. Peng, J. Li, L. Wang, J. C. Marioni, P. P. L. Tam and N. Jing (2023). "Time space and single-cell resolved tissue lineage trajectories and laterality of body plan at gastrulation." Nat Commun **14**(1): 5675.
Warga, R. M. and C. B. Kimmel (1990). "Cell movements during epiboly and gastrulation in zebrafish." Development **108**(4): 569-580.
Yang, Y. P. and J. Klingensmith (2006). "Roles of organizer factors and BMP antagonism in mammalian forebrain establishment." Dev Biol **296**(2): 458-475.

Yashiro, K., Y. Saijoh, R. Sakuma, M. Tada, N. Tomita, K. Amano, Y. Matsuda, M. Monden, S. Okada and H. Hamada (2000). "Distinct transcriptional regulation and phylogenetic divergence of human LEFTY genes." Genes Cells **5**(5): 343-357.
Zhang, H., S. Chen, C. Shang, X. Wu, Y. Wang and G. Li (2019). "Interplay between Lefty and Nodal signaling is essential for the organizer and axial formation in amphioxus embryos." Dev Biol.

Reviewer 4 (technical report):
This manuscript describes analysis of an extensive single-cell RNA-seq (scRNA-seq) time series dataset over murine embryonic development in Lefty1 and Lefty2 KO settings and a comparable WT control condition. With these data, the main goal is to learn where and how each KO impacts progression of transcriptomic states, especially in light of NODAL signaling which is expected to mediate the impacts of these perturbations. To accomplish this, the authors perform relatively standard scRNA-seq processing and embedding, which reveals (1) the absence of later gastrulation states which do appear in the control, indicating an aberrant arrest of development in mutant conditions, (2) a map of Lefty1/2 transcription which generates hypotheses on why this happens, (3) a map of downstream NODAL signaling (via integration with ChIP-seq data and unbiased transcriptomic analysis).

Overall, the scRNA-seq analysis is sound, following widely accepted methods for data cleaning (CellBender), filtering (Scrublet), and embedding (Seurat). Moreover, the conclusions drawn from the scRNA-seq data in this study are largely derived from extremely strong signals (e.g. the absence of entire large clusters in some datasets versus others). Even with slight variations in analysis, I imagine the major conclusions would hold. As it currently stands, I believe the analysis itself is acceptable for publication.

We thank the reviewer for their assessment of our computational analysis, below are point by point comments and corrections related to their assessment.

That said, I have some comments on the Methods, the most major of which surround its clarity and the justification for various decisions. I detail these below:

1. Data Integration: Integration of mutant with WT datasets is arguably the most important step of the analysis for this paper, as it drives most of the conclusions regarding the effects of mutations.
a. The language used throughout the Results (e.g. “project mutant onto WT reference atlas”) seemed to imply that the embedding was first generated on WT cells, and mutant cells are somehow embedded on top of that space. However, the Methods don’t describe such a procedure, leading me to believe that these are all just joint embeddings without additional harmonization. This deserves clarity, as the most important result of the paper hinges on how this was done.

We thank the reviewer for raising this important point. We did not perform a joint integration of the mutant and WT datasets. Instead, the WT reference atlas was kept separate and used as a reference for label transfer and embedding projection. Specifically, cell state labels were assigned to mutant cells using Seurat’s ‘FindTransferAnchors’ and ‘TransferData’ functions, and mutant cells were projected onto the WT UMAP embedding using ‘MapQuery.’ Therefore, the mutant cells were not included in the construction of the WT reference embedding.

To clarify this point, we have revised the Methods section (“Data Processing and Clustering of WT scRNA-seq Atlas”) to explicitly describe the reference mapping procedure：

“*Lefty* mutant scRNA-seq data were preprocessed using the same pipeline as the WT reference dataset. For cell state annotation, Seurat's ‘FindTransferAnchors’ and ‘TransferData’ functions were used to transfer state labels from the WT reference to the *Lefty* mutant dataset. For UMAP projection, the ‘MapQuery’ function was applied to project the mutant cells onto the WT reference embedding.”

b. To justify and understand the embedding in either case, I would suggest adding some metric to visualize the mixing of Lefty1/2 and WT states within the spaces that they overlap. An example might be the entropy of the distribution of conditions for each cell's neighbors, such that high entropy suggests strong mixing.

We thank the reviewer for this helpful suggestion. To quantify the local mixing between WT and Lefty1/2 cells in the shared embedding, we calculated the Shannon entropy of condition labels among the 30 nearest neighbors of each cell. High entropy values indicate strong local mixing between WT and Lefty cells, whereas low entropy values reflect condition-specific segregation.

As shown below in the included reviewr figure (left: *Lefty1* KO vs. WT; right: *Lefty2* KO vs. WT), cell types exhibiting high entropy values include the extraembryonic ectoderm, blood, and extraembryonic endoderm, suggesting that these populations are relatively unaffected in *Lefty* mutants, consistent with the results of the cell proportion analysis. In contrast, more differentiated mesodermal derivatives display lower entropy values, indicating increased separation between WT and *Lefty* mutants and suggesting that these lineages are more strongly affected by the loss of Lefty.

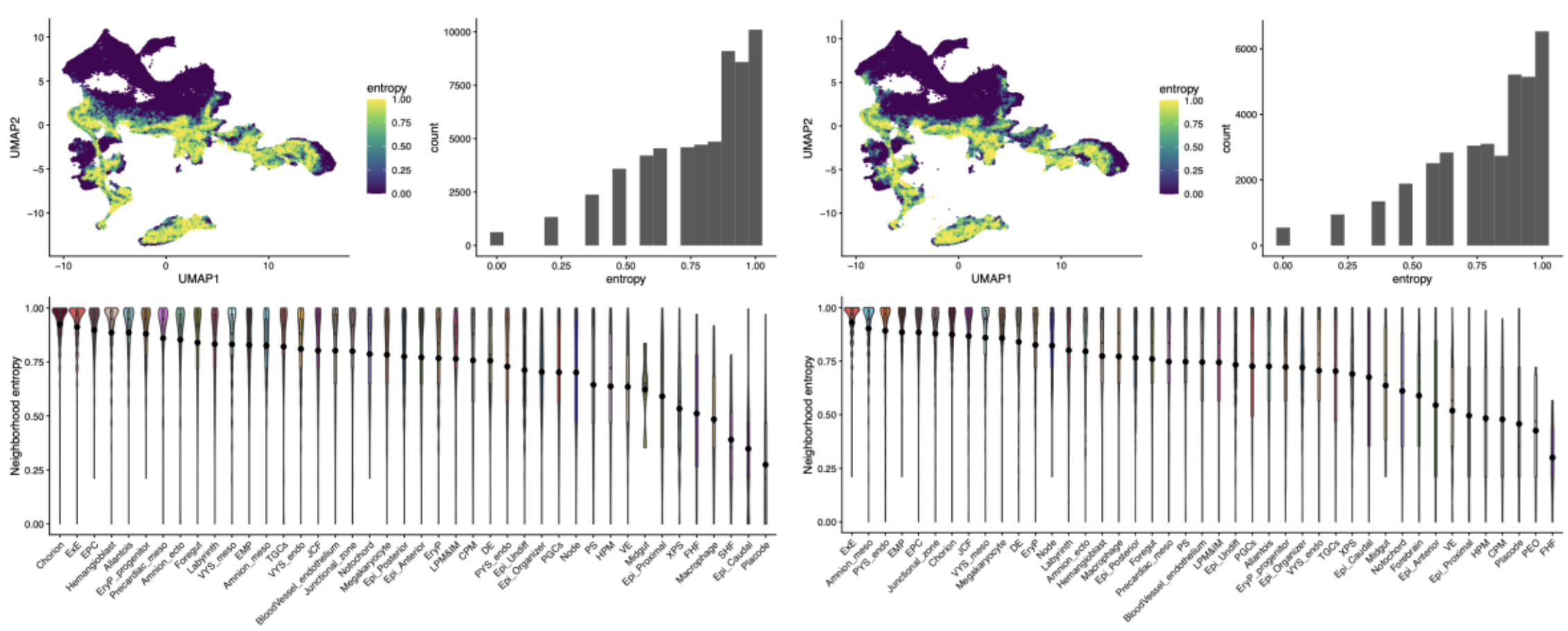

Reviewer Figure 1. Computed entropy analysis for mutant Lefty 1 (left) and Lefty 2 (right) mutant cells conform with the conclusions of our manuscript, whereby extraembryonic cells are largely unaffected, while embryonic mesodermal cell types are.

c. Relatedly, I am curious whether there is any differential expression between cells which are well-matched at the integration level, but come from different conditions. The Milo tool (Dann et al 2022) would be suitable to check for these more subtle differences which can often be missed by focusing on coarse compositions.

We thank the reviewer for this suggestion. We agree that it is important to determine whether transcriptional differences persist between cells occupying similar transcriptional states. We explored this question by comparing WT and Lefty mutant cells within each annotated state. We did not observe evidence for the emergence of novel transcriptional states or widespread

transcriptional remodeling in mutant cells. Instead, the observed differences were largely restricted to increased expression of established Nodal target genes, consistent with enhanced Nodal signaling rather than a global shift in cell identity. Therefore, we conclude that the principal effect of Lefty loss is modulation of signaling activity within existing cell states, rather than the generation of new transcriptional programs. Because our analyses did not reveal substantial transcriptional divergence beyond pathway-level changes within matched states, we did not pursue neighborhood-based differential abundance analysis (e.g. Milo), which is primarily designed to detect local compositional differences.

2. Data processing: I believe the data processing steps are all sensible, but could be further justified. In particular:

a. Was ambient RNA correction with CellBender a necessary step to obtain the results? These sort of large-scale changes to the data are sometimes important, but can make major changes to the underlying data structure. This should be justified with an explanation or, ideally, with a comparison to un-corrected data embeddings.

We thank the reviewer for this important comment. Ambient RNA correction using CellBender was not required to obtain the major conclusions of this study. Our initial analysis pipeline did not include this step, and the overall results and biological conclusions remained consistent. CellBender was subsequently incorporated as part of a unified and more stringent preprocessing pipeline applied consistently across datasets to minimize potential ambient RNA contamination.

We agree that ambient RNA correction can influence data structure in some contexts; however, in our datasets, the major cell states and overall embeddings were largely unchanged after correction, indicating that the conclusions presented in this study are not dependent on the use of CellBender.

b. Can the authors please clarify the motivation for their UMI threshold selection for filtering (as opposed to more standard approaches, e.g. taking the lower mode in the UMI count distribution)? Was this threshold defined per independent sample?

We thank the reviewer for this question. The UMI count distributions in our datasets were generally bimodal, with the lower-count population likely representing low-quality or incomplete cells. Rather than selecting the lower mode or the local minimum between the two modes, we used a robust, sample-specific filtering strategy based on the median absolute deviation (MAD). Cells with low UMI counts were identified using a cutoff defined as the larger of (i) a median − 1.5 × MAD–based threshold and (ii) a minimum UMI threshold of 2,000 UMIs.

This minimum threshold was introduced to prevent overly permissive filtering in samples with low sequencing depth or narrow UMI distributions. Importantly, all thresholds were determined independently for each GEM well, allowing the filtering procedure to adapt to sample-specific characteristics.

Finally, we visually inspected the UMI count distributions for all samples and confirmed that the resulting cutoffs consistently fell near the boundary between the low- and high-UMI populations, in agreement with the expected separation of low-quality and high-quality cells.

c. Do the Scrublet identified doublets have a higher proportion of cells mixed across assigned embryos? This would nicely validate the doublet detection.

We thank the reviewer for this insightful suggestion. In our analysis, embryo identity was inferred using a k-means clustering–based approach applied to SNP profiles, where each cell was assigned to the most likely embryo of origin. As a result, each cell receives a single embryo label, and therefore Scrublet-identified doublets do not explicitly exhibit "mixed embryo assignments" in the downstream labeling step.

However, we do observe that cells flagged as doublets by Scrublet are enriched at the periphery of clusters in the embedding space. Consistently, these cells show more ambiguous SNP profiles compared to high-confidence singlets, suggesting increased likelihood of mixed genetic signals. This observation agrees with the expectation that doublets tend to represent transcriptional and/or genotypic mixtures of distinct cell states.

d. What is meant by "shadow cluster" in this case – please define more clearly.

We thank the reviewer for this suggestion. In the context of our analysis, we define "shadow clusters" as low-density or peripheral cell populations that appear adjacent to major clusters in the embedding space but do not represent distinct, well-separated biological cell types. Instead, these clusters typically exhibit transcriptional profiles highly similar to their corresponding main clusters, with subtle shifts likely reflecting technical noise, partial transcriptional states, or doublet contamination.

We have updated the explanation of shadow cluster in the method section "Data Processing and Clustering of WT scRNA-seq Atlas:

"Shadow clusters were defined as low-density or peripheral cell populations in the embedding space that appear adjacent to major clusters but do not represent distinct, well-separated biological cell types, often exhibiting transcriptional similarity to their corresponding main clusters and reflecting technical noise, incomplete lysis, partial cell states, or doublet-associated signals."

e. Please justify why metacell-level aggregation becomes necessary for NODAL score computation. As this is typically done to circumvent sparsity, I assume some issues might have been encountered in attempting this at the single-cell level.

We thank the reviewer for this question. We would like to clarify that NODAL scores were indeed first computed at the single-cell level. The use of metacell aggregation was not due to an inability to perform single-cell analysis, but rather as an additional step to improve robustness.

Specifically, metacell-level aggregation was used to reduce sparsity and technical noise inherent in single-cell expression data, thereby stabilizing downstream score estimation. In addition, metacells provide a more robust representation for visualization and facilitate downstream non-negative matrix factorization (NMF)-based analyses, where reduced noise and improved signal coherence are beneficial.

Importantly, the main biological conclusions remain consistent between single-cell and metacell-level analyses, indicating that the aggregation step does not alter the interpretation but improves the stability of the scoring and downstream decomposition.

f. Relatedly, why are cell cycle and other confounding signals removed only at this stage of the analysis? Were highly variable genes not used earlier for PCA computation and if not, then why?

We did not perform cell cycle or other confounder removal at any stage of the analysis. PCA was computed using only highly variable genes, as is standard in scRNA-seq workflows. Therefore, the premise that confounding signals were removed at a later stage does not apply to our pipeline. We have clarified this in the Methods section to avoid confusion.

Minor Points:
The authors spend a lot of effort developing a signature for NODAL output, but then rely on a fully-unbiased method for module detection (NMF). A better harmonization of these could be produced by using a method like Spectra (Kunes et al 2024). This isn't a necessary addition – just a suggestion.

We chose NMF because it provides a data-driven, parts-based decomposition that is well-suited for identifying coherent, non-overlapping activity modules. In our setting, this is particularly useful for separating putative NODAL-associated activation patterns from other signals without imposing prior structure, which helps ensure robustness and interpretability.

That said, we agree that methods such as Spectra (Kunes et al., 2024) could offer a more explicit harmonization between the NODAL-derived signature and module detection. We will look into this approach and consider it for strengthening the integration between the signature definition and downstream decomposition in future work.

Finally, I would suggest clarifying in each UMAP precisely which cell subsets are being plotted. For example, it took me a while to understand which cells are WT/Lefty1/2 mutants in Fig 2a,c.

Thank you for the suggestion. We have updated the legend to make things clearer.
