# Supplementary material for: Consecutive waves of NODAL inhibition are required for epiblast patterning
Source: Nat Commun. 2026 Oct 9;17:11484. doi: 10.1038/s41467-026-78248-8 (PMC13740313; doi:10.1038/s41467-026-78248-8)
Supplement: Supplementary file 3 [file 41467_2026_78248_MOESM3_ESM.pdf]

nature portfolio

Corresponding author(s): *Double-anonymous peer review submissions: write DAPR and your manuscript number here instead of author names.*

Last updated by author(s): *YYYY-MM-DD*

Reporting Summary

Nature Portfolio wishes to improve the reproducibility of the work that we publish. This form provides structure for consistency and transparency in reporting. For further information on Nature Portfolio policies, see our Editorial Policies and the Editorial Policy Checklist.

Statistics

For all statistical analyses, confirm that the following items are present in the figure legend, table legend, main text, or Methods section.

n/a	Confirmed	
[ ]	[x]	The exact sample size (*n*) for each experimental group/condition, given as a discrete number and unit of measurement
[ ]	[x]	A statement on whether measurements were taken from distinct samples or whether the same sample was measured repeatedly
[ ]	[x]	The statistical test(s) used AND whether they are one- or two-sided<br>*Only common tests should be described solely by name; describe more complex techniques in the Methods section.*
[x]	[ ]	A description of all covariates tested
[ ]	[x]	A description of any assumptions or corrections, such as tests of normality and adjustment for multiple comparisons
[ ]	[x]	A full description of the statistical parameters including central tendency (e.g. means) or other basic estimates (e.g. regression coefficient) AND variation (e.g. standard deviation) or associated estimates of uncertainty (e.g. confidence intervals)
[ ]	[x]	For null hypothesis testing, the test statistic (e.g. *F*, *t*, *r*) with confidence intervals, effect sizes, degrees of freedom and *P* value noted<br>*Give P values as exact values whenever suitable.*
[x]	[ ]	For Bayesian analysis, information on the choice of priors and Markov chain Monte Carlo settings
[x]	[ ]	For hierarchical and complex designs, identification of the appropriate level for tests and full reporting of outcomes
[ ]	[x]	Estimates of effect sizes (e.g. Cohen's *d*, Pearson's *r*), indicating how they were calculated

Our web collection on statistics for biologists contains articles on many of the points above.

Software and code

Policy information about availability of computer code

Data collection	No Software was used for data collection
Data analysis	The following software tools were used in this study: 10x Genomics Cell Ranger v6.0.2; CellBender v0.3.0; SeuratDisk v0.0.0.9021; Seurat v5.1.0; Scrublet v0.2.3; TrimGalore v0.6.7; STAR v2.5.3; STAR v2.7.7a; SNPsplit v0.3.4; Bowtie2 v2.4.2; SAMtools v1.16; MACS v2.2.7.1; Singlet v0.0.99; Bedtools v2.30.0; HOMER v4.11; DESeq2 v1.34.0; deepTools v3.5.5; RcppML v0.3.7; metacell 0.3.7; ggplot2 v3.4.4; clusterProfiler v4.14.6; igraph 1.0.1; Cytoscape 3.10.4

For manuscripts utilizing custom algorithms or software that are central to the research but not yet described in published literature, software must be made available to editors and reviewers. We strongly encourage code deposition in a community repository (e.g. GitHub). See the Nature Portfolio guidelines for submitting code & software for further information.

Data

Policy information about availability of data

All manuscripts must include a data availability statement. This statement should provide the following information, where applicable:

- Accession codes, unique identifiers, or web links for publicly available datasets
- A description of any restrictions on data availability
- For clinical datasets or third party data, please ensure that the statement adheres to our policy

● All datasets have been deposited in the Gene Expression Omnibus (GEO) under accessions GSE272574 (access token: ohwpiagmftkzzwn) for the wild type reference and GSE266636 (access token: uvmrkucelvottuv) for the mutant data. Previously published data used in this study include ChIP-seq data and RNA-seq

data from GSEGSE70486 and GSE125116.

Research involving human participants, their data, or biological material

Policy information about studies with human participants or human data. See also policy information about sex, gender (identity/presentation), and sexual orientation and race, ethnicity and racism.

Reporting on sex and gender	Not applicable.
Reporting on race, ethnicity, or other socially relevant groupings	Not applicable.
Population characteristics	Not applicable.
Recruitment	Not applicable.
Ethics oversight	Not applicable.

Note that full information on the approval of the study protocol must also be provided in the manuscript.

Field-specific reporting

Please select the one below that is the best fit for your research. If you are not sure, read the appropriate sections before making your selection.

- [x] Life sciences
- [ ] Behavioural & social sciences
- [ ] Ecological, evolutionary & environmental sciences

For a reference copy of the document with all sections, see nature.com/documents/nr-reporting-summary-flat.pdf

Life sciences study design

All studies must disclose on these points even when the disclosure is negative.

Sample size	We aimed to sample 10 or more embryos per time point. No statistical methods for predetermining the sample size were applied. We decided on this sample size to maximize the number of replicates, yet recover a sufficient amount of cells per embryo to capture the overall cell state proportion, under the scalability limit in the 10x Chromium platform (10000~20000 cells for 1-2 GEM wells per sample).
Data exclusions	When preparing the single-cell sequencing library, we sorted the full clutch of embryos from 3 or more fosters per target prior to selection for inclusion into a single cell cohort. In general, we applied no additional selection criteria.
Replication	Our observation is supported by a replicate power of average 11.1 embryos per condition per time point, and the transcriptional responses were consistently confirmed across Lefty mutants under the similar biochemical functions.
Randomization	We did not perform sample randomization because we sampled as many embryos as possible within the scalability limit of the 10x Chromium platform.
Blinding	Blinding was not applied to this study because sequencing analysis is not based on subjective measurement.

Reporting for specific materials, systems and methods

We require information from authors about some types of materials, experimental systems and methods used in many studies. Here, indicate whether each material, system or method listed is relevant to your study. If you are not sure if a list item applies to your research, read the appropriate section before selecting a response.

Materials & experimental systems

n/a	Involved in the study	
[ ]	[x]	Antibodies
[ ]	[x]	Eukaryotic cell lines
[x]	[ ]	Palaeontology and archaeology
[ ]	[x]	Animals and other organisms
[x]	[ ]	Clinical data
[x]	[ ]	Dual use research of concern
[x]	[ ]	Plants

Methods

n/a	Involved in the study	
[x]	[ ]	ChIP-seq
[x]	[ ]	Flow cytometry
[x]	[ ]	MRI-based neuroimaging

Antibodies

Antibodies used	Antibody and reagents used in this study: Sox2 (1:800, rat, #14-9811-82, Invitrogen); Brachyury (1:500, Rabbit, #81694S, CST); Eomes

	(1:200, Rabbit, #Ab23345, Abcam); Cer1 (1:50, Rat, MAB1986-SP, R&D); Foxa2 (1:500; Rabbit, #8186S, CST); Sox17 (1:400, Goat, #AF1924, R&D); Afp (1:100, Mouse, #MAB1368, R&D); Laminin A (1:500, Rabbit, #L9393, Millipore); Phalloidin-647 (1:40, A22287, Invitrogen); DAPI (1:1000, 1mg/mL, D1306, Invitrogen); Donkey anti-Rat IgG (1:1000, Alexa Flur 488, #A48269, Invitrogen); Donkey anti-Rabbit IgG (1:1000, Alexa Flur 555, #A31572, Invitrogen); Donkey anti-Goat IgG (1:1000, Alexa Flur 555, #A-21432, Invitrogen); Donkey anti-mouse IgG (1:1000, Alexa Flur 647, #A-31571).
Validation	Antibodies used in this study are widely used, and batch-batch consistencies are validated by the manufacturer.<br>Links:<br>hSox17, https://www.rndsystems.com/products/human-sox17-antibody_af1924<br>Laminin A: https://www.sigmaaldrich.com/US/en/product/sigma/l9393?srsltid=AfmBOoqPoZIwtdv8SwqthCr9nREUlo0Ly4YVK78CaBIzNU62j6dsfDZz<br>Foxa2: https://www.cellsignal.com/products/primary-antibodies/foxa2-hnf3b-d56d6-xp-rabbit-mab/8186?utm_term&utm_campaign=SO-Products-PrimaryAntibodies&utm_source=adwords&utm_medium=ppc&hsa_acc=8625036580&hsa_cam=21865491299&hsa_grp=167649643577&hsa_ad=730824565107&hsa_src=g&hsa_tgt=dsa-2373170399499&hsa_kw&hsa_mt&hsa_net=adwords&hsa_ver=3&gad_source=1&gad_campaignid=21865491299&gbraid=0AAAAAD_viZIOcwnM9eVhcpuvyr6o1OA4u&gclid=Cj0KCQjwnovFBhDnARIsAO4V7mD-zYHgkoaCn9DHvHxZukFjIpP1g-xPXPd0yD4MqigM_xSl3vgQpXgaAk0LEALw_wcB<br>T: https://www.cellsignal.com/products/primary-antibodies/brachyury-d2z3j-rabbit-mab/81694?utm_term&utm_campaign=SO-Products-PrimaryAntibodies&utm_source=adwords&utm_medium=ppc&hsa_acc=8625036580&hsa_cam=21865491299&hsa_grp=167649643577&hsa_ad=730824565110&hsa_src=g&hsa_tgt=dsa-2373170399499&hsa_kw&hsa_mt&hsa_net=adwords&hsa_ver=3&gad_source=1&gclid=Cj0KCQiAz6q-BhCfARIsAOezPxlRHk6weIIqZYsoYyylES5ywuT88CGk3fVWGqMhR5xxkI-OwptjXL8aAnk9EALw_wcB<br>Afp: https://www.rndsystems.com/products/human-mouse-alpha-fetoprotein-afp-antibody-189502_mab1368<br>Eomes: https://www.abcam.com/en-us/products/primary-antibodies/tbr2-eomes-antibody-ab23345<br>Cer1:https://www.rndsystems.com/products/mouse-cerberus-1-antibody-225807_mab1986#product-datasheets<br>Sox2, https://www.thermofisher.com/antibody/product/SOX2-Antibody-clone-Btjce-Monoclonal/14-9811-82

Eukaryotic cell lines

Policy information about cell lines and Sex and Gender in Research

Cell line source(s)	We tested the spontaneous differentiation of mESCs under normoxic and hypoxic conditions in the presence or absence of SMAD2/3 inhibition. Mouse Embryonic Stem Cells KH2 lines (Beard et al. 2006, PMID: 16400644)
Authentication	The KH2 cell line is routinely validated for the presence of integrated transgenes, namely an RTTA and a dox inducible promoter. We further validate them by the expression of pluripotency markers and the generation of high grade midgestation chimeras after injection into host embryos.
Mycoplasma contamination	The KH2 cell line is routinely tested for mycoplasma and maintained indefinitely ias mycoplasma negative.
Commonly misidentified lines<br>(See ICLAC register)	Not applicable.

Animals and other research organisms

Policy information about studies involving animals; ARRIVE guidelines recommended for reporting animal research, and Sex and Gender in Research

Laboratory animals	We used laboratory mouse (Mus musculus) for this study. Sperms collected from B6/CAST F1 mice were injected into B6D2F1/J mouse oocytes, and transferred into CD-1 strain female surrogates. The embryos were collected at embryonic days (E)6.5 to E9.0
Wild animals	Not applicable.
Reporting on sex	Our WT and mutant embryo cohorts include both male and female embryos (46 males and 49 females for WT, and 55 males and 25 females for mutant). The sex was assigned based on chromosomal gene expression profile for each embryo. As our observations were consistent across replicate individuals, they are applied to both sexes.
Field-collected samples	Not applicable.
Ethics oversight	Interventional procedures necessary to conduct this work were approved by the Yale University Committee on the Use of Animals in Research and Teaching (IACUC, Protocol #2026-20357) or by the LaGeSo Berlin (Protocol #G0098/23).

Note that full information on the approval of the study protocol must also be provided in the manuscript.

Plants

Seed stocks	Not applicable.
Novel plant genotypes	Not applicable.
Authentication	Not applicable.
